# Structural constraint at a P–P bond: phosphinophosphination of alkenes, alkynes, and carbonyls by a concerted mechanism†

**DOI:** 10.1039/d4sc06581f

**Published:** 2024-11-05

**Authors:** Lijun You, Daniel Roth, Lutz Greb

**Affiliations:** a Anorganisch-Chemisches Institut Ruprecht-Karls-Universität Heidelberg Im Neuenheimer Feld 270 69120 Heidelberg Germany greb@uni-heidelberg.de

## Abstract

Structurally constraining p-block elements has become a powerful strategy for bond activation chemistry with main group compounds. Traditionally, this approach focuses on mononuclear centers, yet applying structural constraints to systems with element–element bonds remains underexplored. In this study, we introduce a cation featuring a structural constraint-elongated P–P bond that spontaneously adds to unactivated alkynes, alkenes, aldehydes, and ketones. Despite its positive charge, the surprisingly apolar P–P^+^ bond promotes phosphinophosphination *via* a concerted, highly regio- and diastereoselective mechanism. This unique reactivity opens pathways to novel seven-membered phosphorus heterocycles with customizable optical properties and a structurally varied array of ligands for transition metal coordination.

## Abstract 2

A structural constraint approach at a P–P bond is presented that enables the phosphinophosphination of alkynes, alkenes, and carbonyls with high regio- and diastereoselectivity. The apolar nature of the P–P bond facilitates a concerted mechanism.

## Graphical abstract

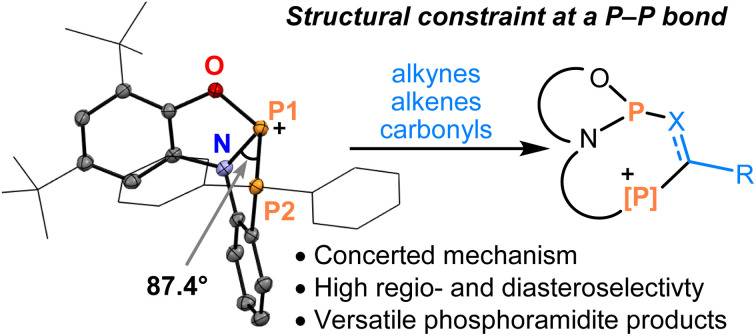

## Introduction

**Scheme 1:**
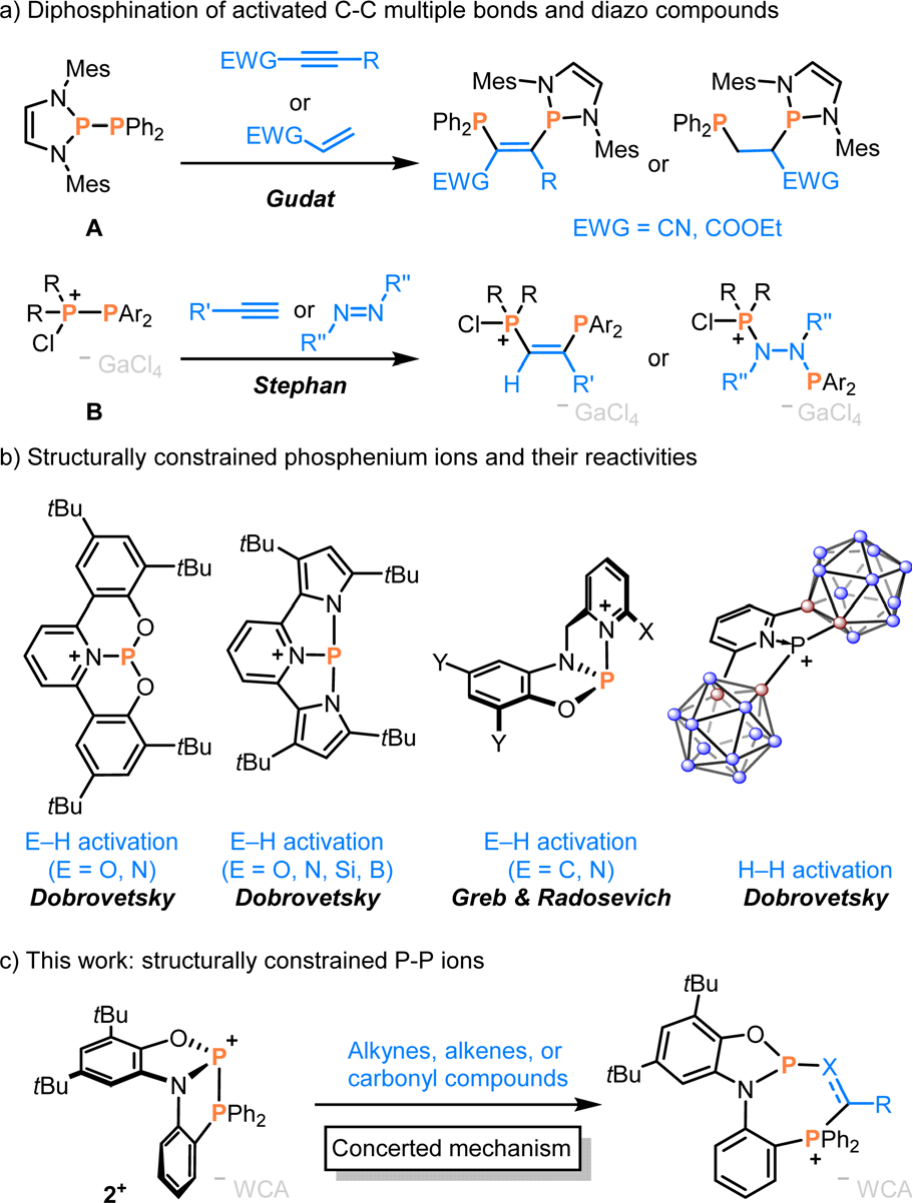
(a) Phosphinophosphination of electron deficient (with A) or electron-rich terminal alkynes and diazo compounds (with B). (b) Structurally constrained phosphenium ions applied for E–H, C–H, and H–H bond activation (E = N, O, Si, B). (c) This work: phosphinophosphination of unsaturated compounds by a concerted mechanism.

Double functionalization of C=C or C=O groups by the addition of element–element bonds, *e.g.*, diboration, disilylation or diphosphination, is a straightforward tool for expanding molecular functionality and complexity.^1–3^ In particular, adding two phosphorus groups across alkenes and alkynes provides manifold products of interest for catalysis, materials science, pharmaceuticals and biological research.^4,5^ Usually, transition metal-, Lewis acid-, or light-induced reactivity *via* phosphinyl radicals is required for P–P to C–C multiple bond additions.^6–9^ While spontaneous diphosphination has been described with a polarized P–P bonded system A, this reactivity is limited to electron-deficient alkenes and alkynes (Scheme 1a).^10–14^ More recently, phosphino-phosphenium cations B were shown to undergo FLP-type addition to alkynes, but limited to terminal electron-rich C–C triple bonds and diazo compounds (Scheme 1a).^15,16^ In this context, P–P bond hydrogenation and its reverse phosphine dehydrocoupling in a FLP manner are to be mentioned.^17,18^ The addition of P–P bonds across carbonyls leading to P–O–C–P motifs has been reported, but only for the electron-deficient bisphosphine (CF_3_)_2_P–P(CF_3_)_2_ under Lewis base catalysis.^19^

Inspired by the continuing interest in structurally constrained compounds,^20–23^ we reasoned whether applying this approach to a P–P bond might facilitate the extension of corresponding diphosphination reactivities. Structurally constrained phosphenium ions have been utilised for the activation of E–H,^24,25^ C–H,^26^ and H–H bonds^27^ (Scheme 1b), while reactions with π-bonds remained underexplored. Moreover, neutral diazadiphosphapentalene featuring a constrained P–P bond was explored for NH_3_ activation *via* σ-bond metathesis.^28^

Here, we describe the phosphanylphosphenium ion 2^+^ and its reactivity towards non-activated alkynes, alkenes, aldehydes, and ketones (Scheme 1c). Comparisons and computations reveal that a surprisingly apolar P–P^+^ bond in 2^+^ leverages high selectivity by a concerted elementary step, while only the structural constraint unlocks the thermodynamic and kinetic feasibility.

## Results and discussion

**Fig. 1:**
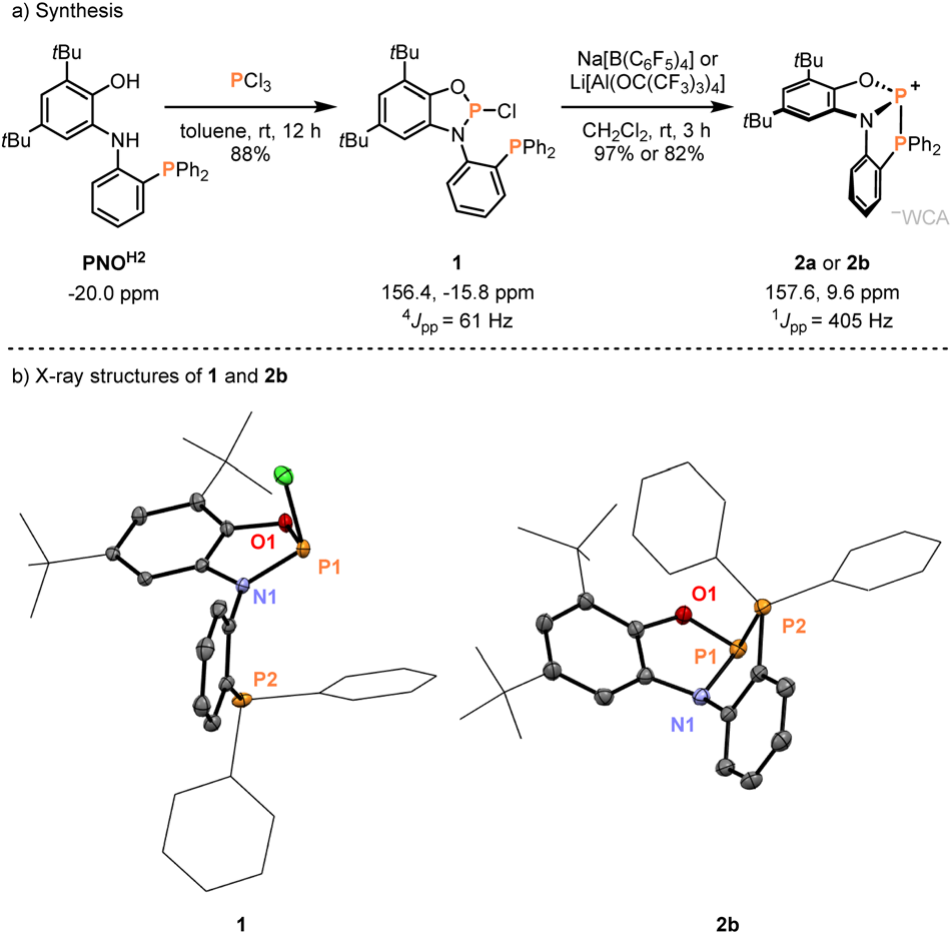
(a) Synthesis of phosphenium salts [2][WCA] with ^31^P NMR data (WCA = weakly coordinating anion). 2a: [2][B(C_6_F_5_)_4_], 97% yield; 2b: [2][AlO(C(CF_3_)_3_)_4_], 82% yield. (b) ORTEP plots of the solid-state structures of 1 and 2^+^ in 2b. Thermal ellipsoid plots are at 50% probability. The counterion [Al(OC(CF_3_)_3_)_4_]^−^ and all hydrogen atoms have been omitted for clarity. Selected bond distances (Å) and angles (deg) of 1: *d*(P1–O1) = 1.6305(2), *d*(P1–N1) = 1.6837(3), and ∠O1–P1–*N*1 = 92.14(57) (the structure of 1 contains two crystallographically independent molecules and the mean values with standard deviation are given); 2^+^: *d*(P1–O1) = 1.6316(15), *d*(P1–N1) = 1.7318(17), *d*(P1–P2) = 2.3437(15), ∠O1–P1–*N*1 = 94.18(7), ∠O1–P1–*P*2 = 104.53(5), and ∠N1–P1–*P*2 = 87.39(5).

We initiated our work by synthesizing the phosphine-appended phosphenium salts 2a/b (Fig. 1). The required diphenylphosphanylaminophenol (PNO^H2^) ligand was obtained at a multi-gram scale according to the reported procedure (Fig. 1a).^29–31^ Treatment of a solution of PNO^H2^ in toluene with PCl_3_ furnished the phosphorus chloride 1 in 88% yield (Fig. 1a). Analysis by ^31^P NMR spectroscopy showed two doublets at 156.4 and −15.8 ppm with equal coupling constants of ^4^*J*_PP_ = 61 Hz. Single crystals suitable for X-ray diffraction grown from a concentrated solution in dichloromethane at −40 °C confirmed the installation of a P–Cl unit at the amidophenolate (Fig. 1b). The *N*-phenyl group is nearly perpendicular to the NPO plane with no direct P–P interaction (see the ESI† for more scXRD information). Chloride abstraction using Na[B(C_6_F_5_)_4_] or Li[Al(OC(CF_3_)_3_)_4_] gave respective phosphinophosphenium salts 2a/b in excellent yields (Fig. 1a). An increased coupling constant of ^1^*J*_PP_ = 405 Hz in the corresponding ^31^P NMR spectra indicated the formation of a P–P bond. Single crystals of [2][Al(OC(CF_3_)_3_)_4_] (2b) analyzed by X-ray diffraction revealed a structure where the cation adopts *C*_*1*_ symmetry in the solid state (Fig. 1b). The distance between the two phosphorus atoms of 2.3437(15) Å is substantially longer compared to untethered phosphinophosphenium ions (*e.g.* 2.230 Å in [Ph_3_P–PPh_2_]^+^)^32^ and similar to that of the polarized systems A (2.334 Å).^10^ While the ∠O1–P1–N1 (94.2°) and the ∠O1–P1–P2 angle (104.5°) are less distorted, the acute ∠N1–P1–P2 angle of 87.39° shows significant deviations compared to unconstrained derivatives (see ESI, Section 9.1†).^32^ This deformation encompassing the P–P unit in 2^+^ indicated potential effects on its reactivity, which were investigated next.

**Scheme 2:**
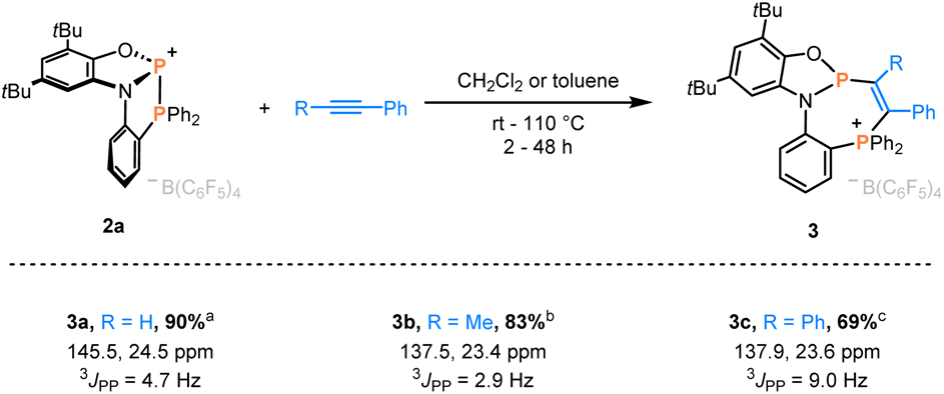
Phosphinophosphination of alkynes affording 3a–c. Isolated yields and ^31^P NMR data are given. Reaction conditions: ^a^ CH_2_Cl_2_, rt, 2 h. ^b^ CH_2_Cl_2_, 60 °C, 18 h. ^c^ toluene, 110 °C, 48 h.

Indeed, swift addition reactions towards broad scope of unsaturated functional groups were observed with high selectivity. Upon exposure of 2a to phenylacetylene, a new species with two doublets in the ^31^P NMR spectra at 145.5 and 24.5 ppm formed selectively (Scheme 2). Combined multinuclear NMR data were consistent with the phosphinophosphination product 3a, as was further confirmed by scXRD (Fig. 3). Notably, 3a represents only one out of four possible diastereomers (see Section 3.1 in the ESI†). Similarly, the reaction with internal alkynes, such as 1-phenyl-1-propyne or tolane afforded the products 3b/c as orange solids in good isolated yields (Scheme 2). This behavior contrasts that of the reported structurally unconstrained A or B (Scheme 1a), which added only to either electron-poor or electron-rich activated terminal alkynes,^10–13,15^ and encouraged the investigation of more challenging substrates.

**Scheme 3:**
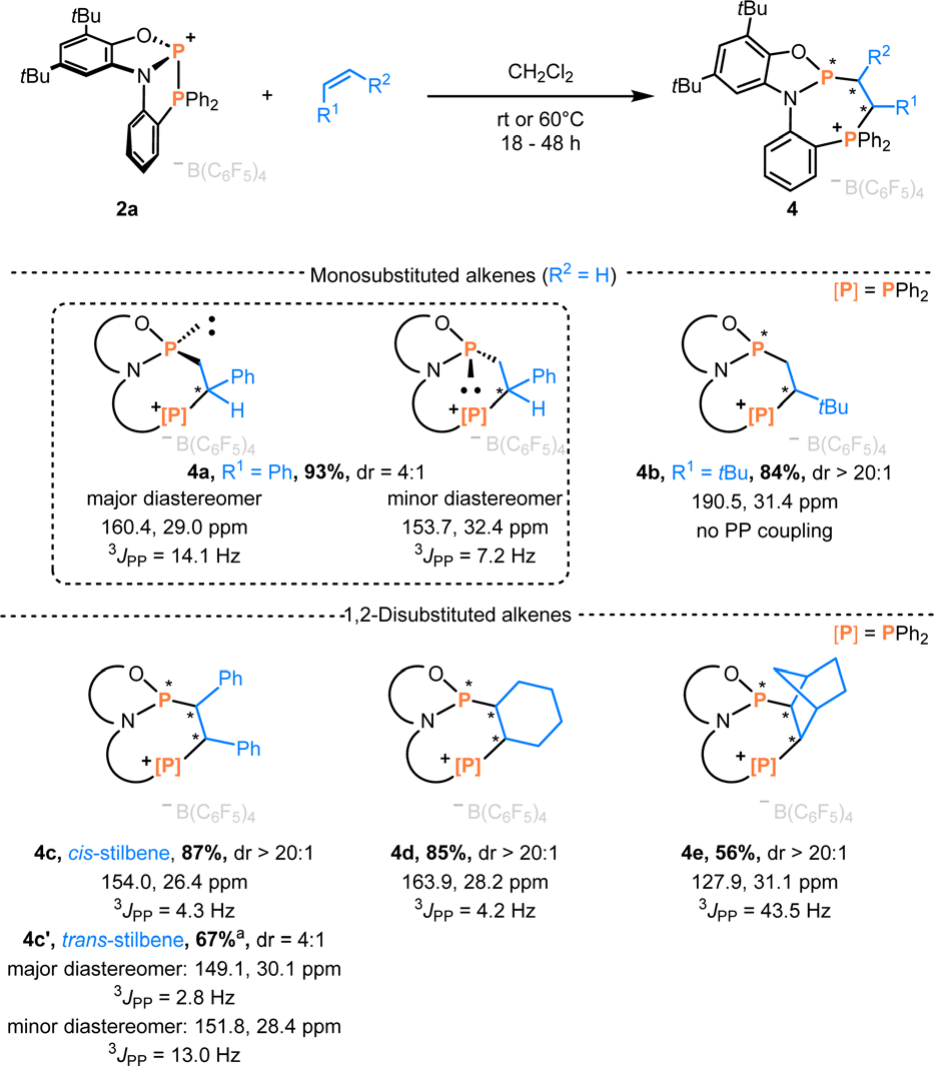
Phosphinophosphination of alkenes affording 4a–e. Isolated yields, ^a^ NMR conversion and ^31^P NMR data are given. The dr values are determined from the reaction mixtures by ^1^H NMR spectroscopy.

On adding styrene to the solution of 2a in CH_2_Cl_2_, multinuclear NMR data revealed two diastereomeric products in a 4 : 1 ratio. In the ^31^P NMR spectra, the two doublets of the major product appeared at 160.4 and 29.0 ppm (^3^*J*_PP_ = 14.1 Hz), while the minor one showed two doublets at 153.7 and 32.4 ppm (^3^*J*_PP_ = 7.2 Hz). Single crystals suitable for X-ray diffraction confirmed the major diastereomer as the racemate of (*Sa*,*S*)-4a and (*Ra*,*R*)-4a (Scheme 3), while the minor diastereomer corresponded to the racemic mixture of (*Sa*,*R*)-4a and (*Ra*,*S*)-4a. Increasing the steric demand of the alkene substituent (R^1^ = *t*Bu) improved diastereoselectivity, and formation of a single racemate ((*Ra*,*R*)-4b)/((*Sa*,*S*)-4b) was observed (Scheme 3), as confirmed by scXRD (Fig. 3). The larger distance between two phosphorus atoms (*d* = 3.813 Å) or an unfavorable conformation of 4b could explain the absence of PP coupling in the ^31^P NMR. Next, the internal alkenes *cis*-stilbene and *trans*-stilbene were examined. The reaction of 2a with *cis*-stilbene at 60 °C for 48 h afforded a single diastereomer with two doublets at 154.0 and 26.4 ppm (^3^*J*_PP_ = 4.3 Hz). Single crystal XRD confirmed the structure as the racemate (*Sa*,*R*,*S*)-4c and (*Ra*,*S*,*R*)-4c. Reacting 2a with *trans*-stilbene under analogous conditions provided two products in a 4 : 1 ratio, identified as the racemates (*Sa*,*R*,*R*)-4c′/(*Ra*,*S*,*S*)-4c′ (major) and (*Sa*,*S*,*S*)-4c′/(*Ra*,*R*,*R*)-4c′ (minor, see Section 4.1 in the ESI†). Hence, full stereoconservation of *cis*- and *trans*-configurations occurred, indicating the absence of long-lived rotatable intermediates but a concerted mechanism. Aliphatic internal alkenes cyclohexene and norbornene provided the phosphinophosphination products 4d/e in good yields with high diastereoselectivities (Scheme 3). To the best of our knowledge, this represents the first spontaneous diphosphination of non-activated C=C double bonds. Surprisingly, with the more activated 1,1-diphenylethylene, no reaction occurred at room temperature or 60 °C in CH_2_Cl_2_ solution, showcasing a behavior generally different from that of conventional P-cationic Lewis acids that exhibit high local positive charge at phosphorus.^33–35^

**Fig. 2:**
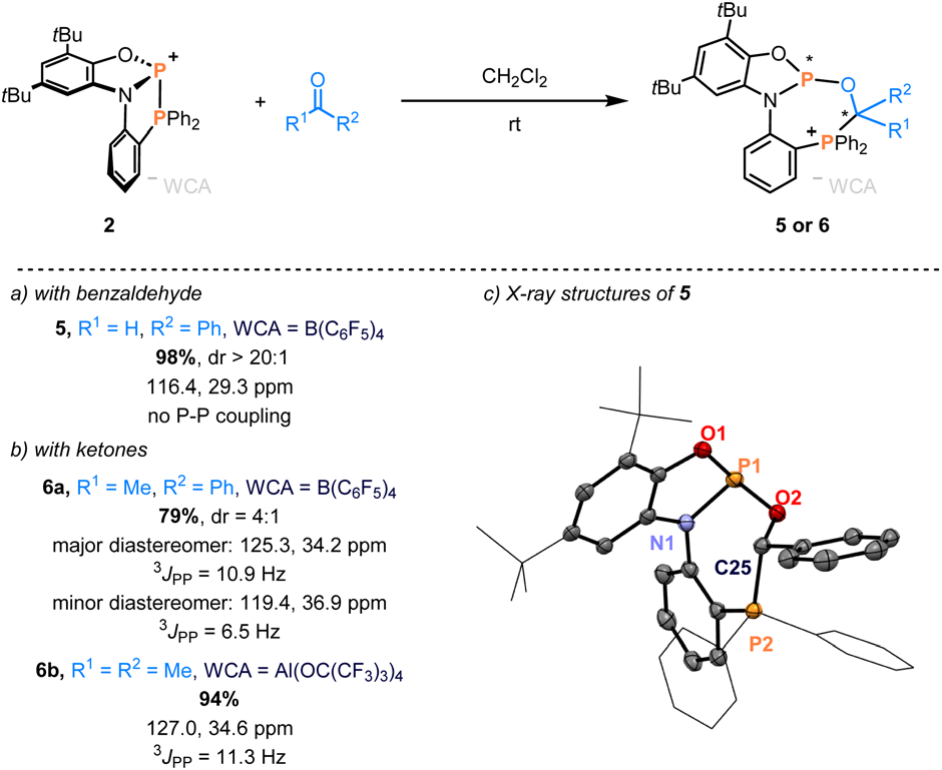
Phosphinophosphination of (a) benzaldehyde and (b) ketones. Isolated yields and ^31^P NMR data are given. The dr values are determined from the reaction mixtures by ^1^H NMR spectroscopy. (c) ORTEP plots of the solid-state structure of 5. Thermal ellipsoid plots are set to 50% probability. The counteranion [B(C_6_F_5_)_4_]^−^ and all hydrogen atoms have been omitted for clarity. Selected bond distances (Å) and angles (deg) of 5: *d*(P1–O1) = 1.6496(11), *d*(P1–N1) = 1.7228(13), *d*(P1–O2) = 1.6442(11), *d*(C25–O2) = 1.4271(17), *d*(P2–C25) = 1.8643(14), *d*(P1–P2) = 3.674, ∠O1–P1–*N*1 = 90.22(6), ∠O1–P1–O2 = 104.22(5), and ∠N1–P1–O2 = 100.76(5).

The scope was expanded further to carbonyl compounds. Benzaldehyde and phosphenium salt 2a reacted to form two diastereomeric addition products after 5 hours. The major diastereomer appeared as two doublets at 126.0 and 30.3 ppm (^3^*J*_PP_ = 5.3 Hz), while the minor diastereomer was observed as two singlets at 116.4 and 29.3 ppm in the ^31^P NMR spectra (Section S5.1 in the ESI†). Interestingly, only the diastereomer with two singlets remained after storing the sample for a week (Fig. 2), indicating the conversion to a thermodynamic product (*vide infra* for the mechanism). Single crystals analyzed by scXRD revealed the regioisomer with the oxygen binding to the central P(iii) (Fig. 2c). Acetophenone was also reactive towards 2a, giving two species in a diastereomer ratio of 4 : 1. The solid-state structure of the major diastereomer was determined through scXRD (see the ESI†). Finally, complete conversion of acetone to a single product occurred at room temperature, described by two doublets at 127.0 and 34.6 ppm and a coupling constant of ^3^*J*_PP_ = 11.3 Hz in the ^31^P NMR spectra.

**Fig. 3:**
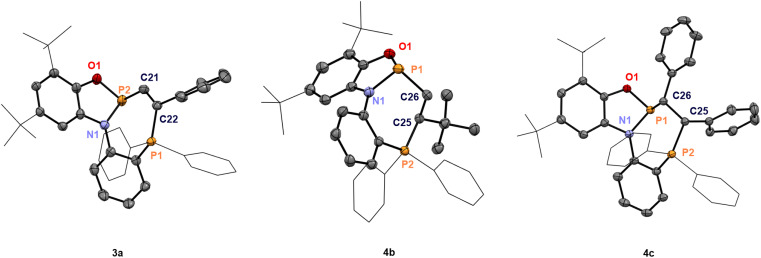
Thermal ellipsoid plots of 3a, 4b, and 4c at 50% probability. The counteranion [B(C_6_F_5_)_4_]^−^ and all hydrogen atoms have been omitted for clarity. Selected bond distances (Å) and angles (deg) of 3a: *d*(P2–O1) = 1.650(2), *d*(P2–N1) = 1.717(2), *d*(P2–C21) = 1.818(3), *d*(C21–C22) = 1.340(4), *d*(P1–C22) = 1.832(3), *d*(P1–P2) = 3.462, ∠O1–P2–*N*1 = 93.09(11), ∠O1–P2–C21 = 103.61(13), and ∠N1–P2–C21 = 99.06(13); 4b: *d*(P1–O1) = 1.666(2), *d*(P1–N1) = 1.725(3), *d*(P1–C26) = 1.831(3), *d*(C25–C26) = 1.538(4), *d*(P2–C25) = 1.852(3), *d*(P1–P2) = 3.813, ∠O1–P1–*N*1 = 91.29(12), ∠O1–P1–C26 = 104.21(13), and ∠N1–P1–C26 = 98.48(13); 4c: *d*(P1–O1) = 1.6436(15), *d*(P1–N1) = 1.7385(16), *d*(P1–C26) = 1.8652(19), *d*(C25–C26) = 1.562(2), *d*(P2–C25) = 1.8426(18), *d*(P1–P2) = 3.447, ∠O1–P1–*N*1 = 92.51(7), ∠O1–P1–C26 = 101.54(7), and ∠N1–P1–C26 = 96.42(8).

**Fig. 4:**
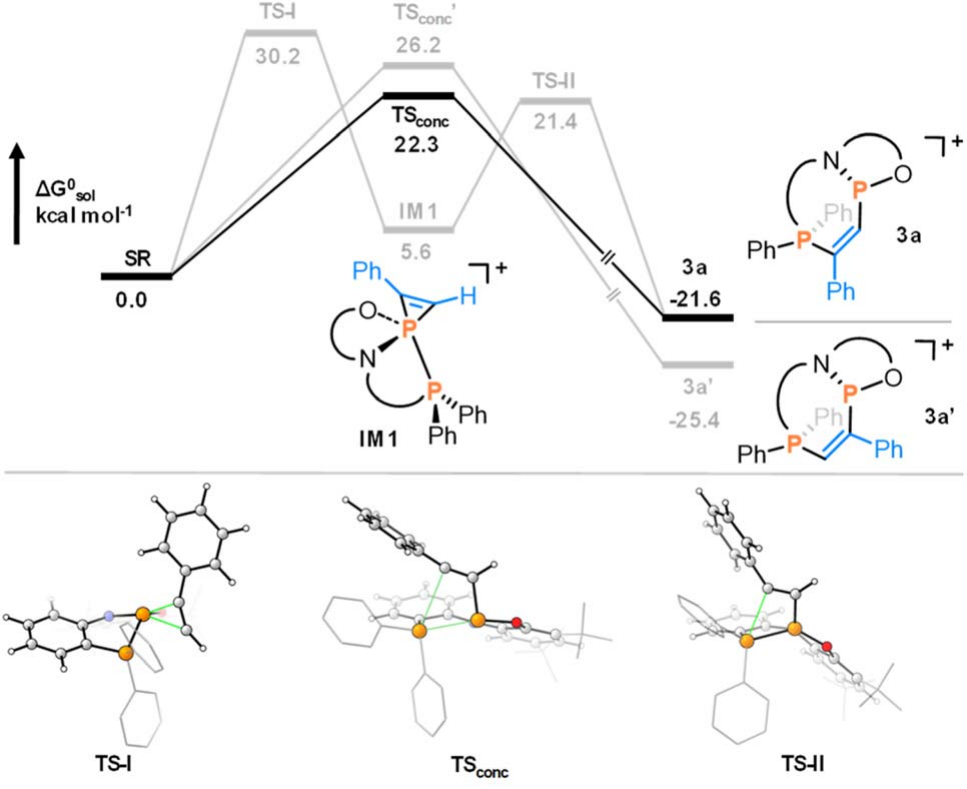
Computed mechanism at the PW6B95-D4/def2-QZVPP(SMD: CH_2_Cl_2_)//r^2^-SCAN-3c(CPCM: CH_2_Cl_2_) level of theory for the addition of phenylacetylene to 2a (black), as well as other higher energy pathways (grey) and transition state structures.

The origins of the high regio- and diastereoselectivity were investigated using DFT calculations at the PW6B95-D4/def2-QZVPP(SMD: CH_2_Cl_2_)//r^2^-SCAN-3c(CPCM: CH_2_Cl_2_) level of theory (for details, see the ESI†).^36–40^ For the reaction with phenylacetylene, the computational results suggested that insertion into the P–P bond occurs in a concerted step (Fig. 4). In the lowest transition state, the phosphenium is close to planar, taking full account of its increased electrophilicity.^21,35^ The formation of a phosphirenium ion (IM1), the proposed intermediate for reactions of B (Scheme 1a),^15^ was ruled out due to a high computed barrier (30.2 kcal mol^−1^).

Regioisomer 3a was identified as the kinetic product (22.3 kcal mol^−1^), while the thermodynamic product 3a′ is disfavored by a higher barrier (26.2 kcal mol^−1^). The analogous reaction with tolane was predicted to proceed through a rate-determining barrier of 26.8 kcal mol^−1^, consistent with the experimentally required higher temperature to drive the reaction (Section S10.1.1. in the ESI†). The reaction of styrene with 2a was similarly found to proceed *via* a concerted addition over a barrier of 20.0 kcal mol^−1^ (Section S10.1.2.†). For benzaldehyde, the intermediate formation of a phosphaoxirane could also be excluded by prohibitively high reaction barriers, but a lower transition state energy of 16.8 kcal mol^−1^ was obtained for the concerted addition to the initially observed kinetic product, in agreement with experimental observations.

The thermodynamic product is separated by a barrier of 18.6 kcal mol^−1^. The experimentally observed interconversion to this more stable diastereomer proceeds either by direct inversion at phosphorus (Δ*G*^‡^ = 22.8 kcal mol^−1^) or by return of the kinetic product back to the reactants (Δ*G*^‡^ = 23.7 kcal mol^−1^). Hence, the observed selectivity relies on the concerted mechanism, which differs from the proposed stepwise process *via* carbanions during the addition of nucleophilic polar P–P bonds in A to electron-deficient alkenes,^13^ or *via* phosphirenium ion intermediates in the case of B and electron-rich alkynes.^15^

The aptitude for such reactivity could be rationalized by NBO analysis. A surprisingly apolar P–P bond in 2^+^ (NPA charge at *P*(N,O) = 1.12 and *P*Ph_2_ = 1.22) contrasts the far more polar situation in neutral A (charge at *P*(N,N) = 1.05 and *P*Ph_2_ = 0.38). This suggests that these apolar features in the P–P bond of 2^+^ favor the concerted mechanism. At the same time, while other apolar P–P bonds might be reluctant for such reactivity with non-activated substrates, we suggest that feasibility becomes unlocked by the structural constraint imposed by the annulated five-membered rings.

**Fig. 5:**
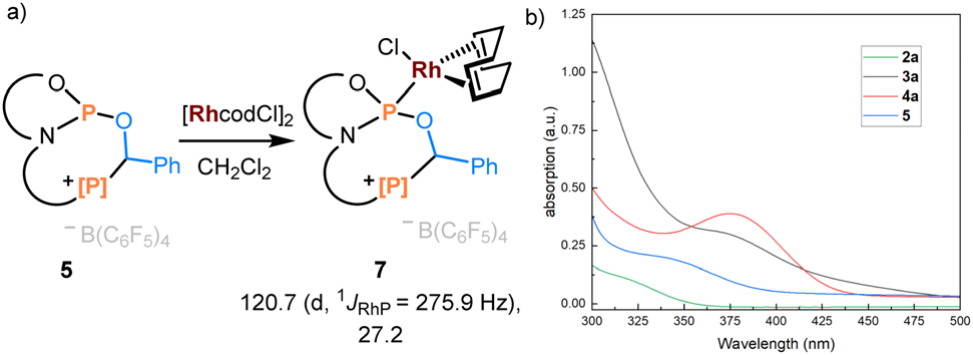
(a) Coordination chemistry of product 5 with [Rh(cod)Cl]_2_. ^31^P NMR data are given. (b) The absorption spectrometry of 2a, 3a, 4a, and 5 in CH_2_Cl_2_ (*c* = 1.0 × 10^−4^ mol L^−1^).

To showcase the value of this new accessible family of phosphorus compounds, their application and properties were considered. Product 5 was treated with [Rh(cod)Cl]_2_, leading to quantitative formation of complex 7 with ^31^P NMR resonances at 120.7 (^1^*J*_RhP_ = 275.9 Hz) and 27.2 ppm (Fig. 5a). The buried volumes of phosphines 3–6 are spanning a remarkable range (see ESI, Section S7.1†), emphasizing the diverse steric profiles of these easily modificable products. Phosphoramidites are a class of privileged ligands in asymmetric rhodium catalysis,^41^ qualifying the above-described protocol as a powerful gateway to a library of cationically charged derivatives.

Furthermore, we examined the optical properties of phosphenium salt 2a and addition products 3a, 4a, and 5 (Fig. 5b). Gradually shifted absorption wavelength maxima were consistent with the emerging color of corresponding products. In line with the extended π-conjugation in the 7-membered ring in 3a, the absorption is extending into the visible region. This represents an attractive approach for the systematic photophysical evaluation of phosphaheterocylces, which are a topical class of compounds.^42–44^

## Conclusions

Herein, we disclose that structurally constraining a P–P^+^ unit enables the phosphinophosphination of unsaturated compounds, including alkynes, olefins, and carbonyls. To our knowledge, such spontaneous reactivity towards non-activated C–C multiple bonds has not been reported previously. High regio- and diastereoselectivity originates from a concerted elementary step, contrasting previous addition reactions that occur *via* stepwise mechanisms and epimerizable intermediates.^13,15,45,46^ The combinatorial nature allows for the construction of cationic phosphoramidite ligands and P-heterocyclic chromophores. More generally, this study highlights the opportunities of imposing structural constraints on less polar structural motifs and encourages expanding this concept toward other element–element bonds.

## Data availability

Crystallographic data have been deposited at the CCDC: 2357323–2357331. Further data supporting this study are available in the ESI.†

## Author contributions

L. Y. and L. G. devised the project and designed the experiments. L. Y. performed the experimental work. D. R. carried out the quantum chemical simulations. L. Y. and D. R. wrote the initial manuscript. All authors contributed to the finalization of the manuscript, and all listed authors agreed to the submitted content.

## Conflicts of interest

There are no conflicts to declare.

## Supplementary Material

SC-OLF-D4SC06581F-s001

SC-OLF-D4SC06581F-s002
