# Supplementary material for: Structural constraint at a P–P bond: phosphinophosphination of alkenes, alkynes, and carbonyls by a concerted mechanism
Source: Chem Sci. 2024 Nov 5;16(4):1716–21. doi: 10.1039/d4sc06581f (PMC11575562; doi:10.1039/d4sc06581f)
Supplement: SC-016-D4SC06581F-s001 [file SC-016-D4SC06581F-s001.pdf]

Supplementary Information (SI) for Chemical Science.
This journal is © The Royal Society of Chemistry 2024

Contents

1. General Information 3

2. Syntheses of Phosphenium Salts 4

2.1 Preparation of 1 (PNOPCl) 4

2.2 Preparation of 2a ([PNOP][B(C_6F_5)$_4$]) 7

2.3 Preparation of 2b ([PNOP][Al(OC(CF_3)$_3$)$_4$]) 11

3. Phosphinophosphination of Alkynes... 14

3.1 Possible diastereomers for the reaction product 14

3.2 Preparation of 3 15

4. Phosphinophosphination of Alkenes... 27

4.1 Reaction development 27

4.2 Preparation of 4 30

5. Phosphinophosphination of Benzaldehyde 52

5.1 Reaction development 52

5.2 Preparation of 5 56

6. Phosphinophosphination of Ketones 61

7. Coordination Chemistry 69

7.1 Steric profiles 69

7.2 Reaction development 70

8. Optical Properties of Select Compounds 74

9. X-Ray Diffraction 75

9.1 Bond angles profile 76

9.2 X-ray crystallography 77

10. Computational Details 95

10.1 Free energy reaction profiles 96

10.2 xyz coordinates 99

11. References 162

1. General Information

Unless stated otherwise, manipulations were carried out under a dry argon atmosphere by using standard Schlenk techniques to prevent oxidation and hydrolysis of sensitive compounds. All solvents were rigorously dried by applying standard procedures, freshly degassed and stored over molecular sieve (3 Å resp. 4 Å) for at least two days prior to use. All glassware, syringes, magnetic stirring bars and needles were thoroughly dried. Commercially available chemicals were purchased and used as received. 2,4-di-*tert*-butyl-(2-diphenylphosphanylanilino)phenol (**PNO^{H2}**)[1], $Na[B(C_6F_5)_4]$[2] and $Li[Al(OC(CF_3)_3)_4]$[3] were prepared according to reported literature procedures. Air or moisture-sensitive compounds were stored in a glove box and handled under N_2 atmosphere. Purity and identity of the compounds were confirmed by high-resolution multinuclear NMR spectroscopy, mass spectrometry, elemental analysis, and, where possible, X-ray diffraction analysis. **^{1}H**, **^{11}B**, **^{13}C**, **^{19}F** and **^{31}P NMR** spectra were recorded at 298 K with a Bruker Avance II 400 or Bruker 2 Avance III 600 NMR spectrometer and referenced to the solvent in use. Chemical shifts are reported as dimensionless δ values in ppm, coupling constants *J* are given in Hertz (Hz). NMR standards were used as follows: (**^{1}H NMR**) CD_2Cl_2 = 5.32 ppm; (**^{13}C{^{1}H} NMR**) CD_2Cl_2= 53.84 ppm. Observed signal patterns are noted according to their multiplicities in the standard fashion (e.g. s = singlet, d = doublet, dd = doublets of doublets, etc.). Overlapping signals with indistinct shapes are described as m = multiplet. Electrospray ionization mass spectra were obtained with a Bruker ApexQe FT-ICR instrument. Absorption spectra were measured on a JASCO V-570 UV/Vis/NIR spectrophotometer.

2. Syntheses of Phosphenium Salts

2.1 Preparation of 1 (PNOPCl)

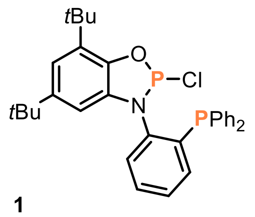

1

PNO^{H2} (2.41 g, 5.0 mmol, 1.0 equiv) was dissolved in toluene (20 mL), followed by the addition of PCl_3 (0.72 g, 5.25 mmol, 1.05 equiv). The solution was stirred at room temperature for 12 h, after which the solvent was removed under reduced pressure. The crude product was washed with pentane and dried under reduced pressure to give the product **1** as a white solid (2.41 g, 4.41 mmol, 88 %). Single crystals suitable for X-ray diffraction were grown by cooling a concentrated solution in CH_2Cl_2 to -40 °C.

^{1}H NMR (600 MHz, CD_2Cl_2): δ 7.54 – 7.46 (m, 2H), 7.44 – 7.35 (m, 4H), 7.30 (ddt, J = 7.9, 6.5, 1.8 Hz, 2H), 7.24 – 7.14 (m, 5H), 7.13 – 7.10 (m, 1H), 6.96 (d, J = 2.0 Hz, 1H), 6.34 (d, J = 1.8 Hz, 1H), 1.44 (s, 9H), 1.21 (s, 9H).

^{13}C{^{1}H} NMR (151 MHz, CD_2Cl_2): δ 146.9 (s, 1C), 144.5 (dd, J = 11.1, 0.8 Hz, 1C), 136.3 (d, J = 0.7 Hz, 1C), 136.2 (dd, J = 11.5, 2.4 Hz, 1C), 135.7 (dd, J = 10.7, 2.6 Hz, 1C), 134.6 (s, 1C), 134.5 (d, J = 1.0 Hz, 2C), 134.4 (d, J = 21.3 Hz, 2C), 134.4 (d, J = 0.9 Hz, 2C), 134.1 (d, J = 2.7 Hz, 2C), 130.7 (s, 2C), 130.2 (d, J = 0.7 Hz, 1C), 129.5 (d, J = 33.8 Hz, 2C), 129.3 (d, J = 0.9 Hz, 1C), 129.2 (d, J = 7.0 Hz, 1C), 128.9 (d, J = 7.6 Hz, 1C), 116.8 (s, 1C), 107.2 (s, 1C), 35.1 (s, 1C), 34.7 (s, 1C), 31.8 (s, 3C), 29.8 (s, 3C).

^{31}P{^{1}H} NMR (243 MHz, CD_2Cl_2): δ 156.4 (d, J = 61.2 Hz, ***P***Cl), -15.8 (d, J = 61.5 Hz, ***P***Ph$_2$).

HRMS (ESI): $[C_{32}H_{35}ClNOP_2]^+$, calculated: 546.1877, found: 546.1885.

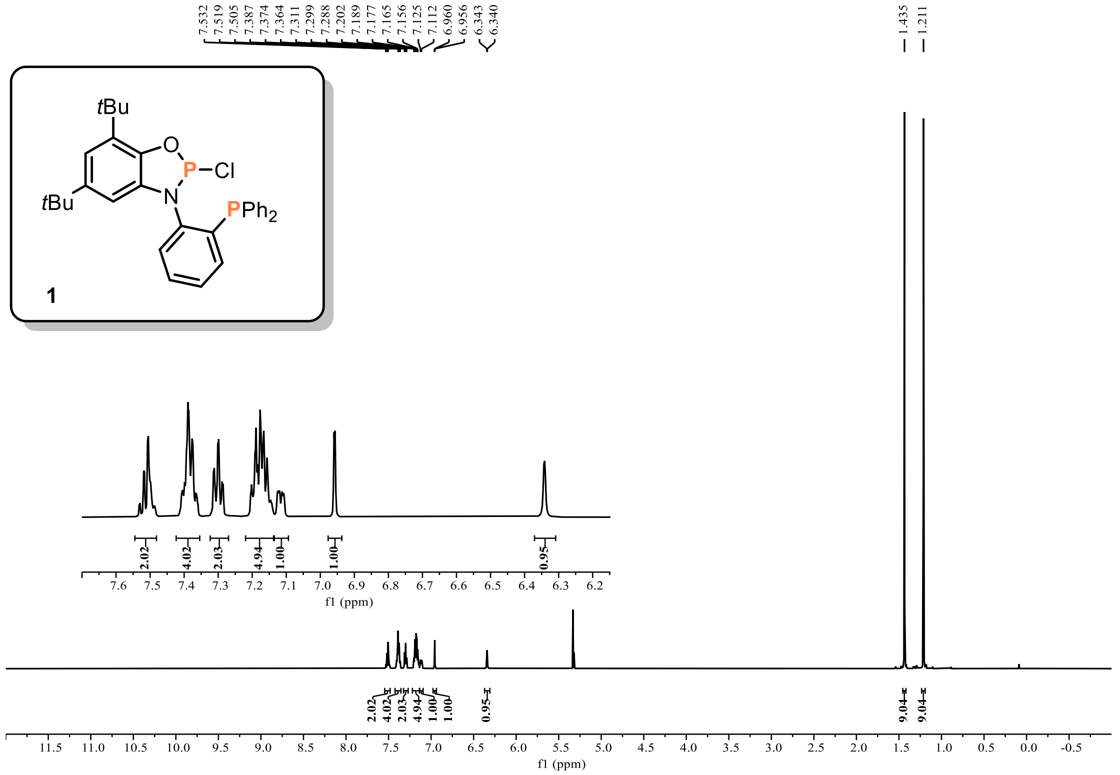

Figure S1: **^{1}H NMR** of **1** (600 MHz, CD_2Cl_2).

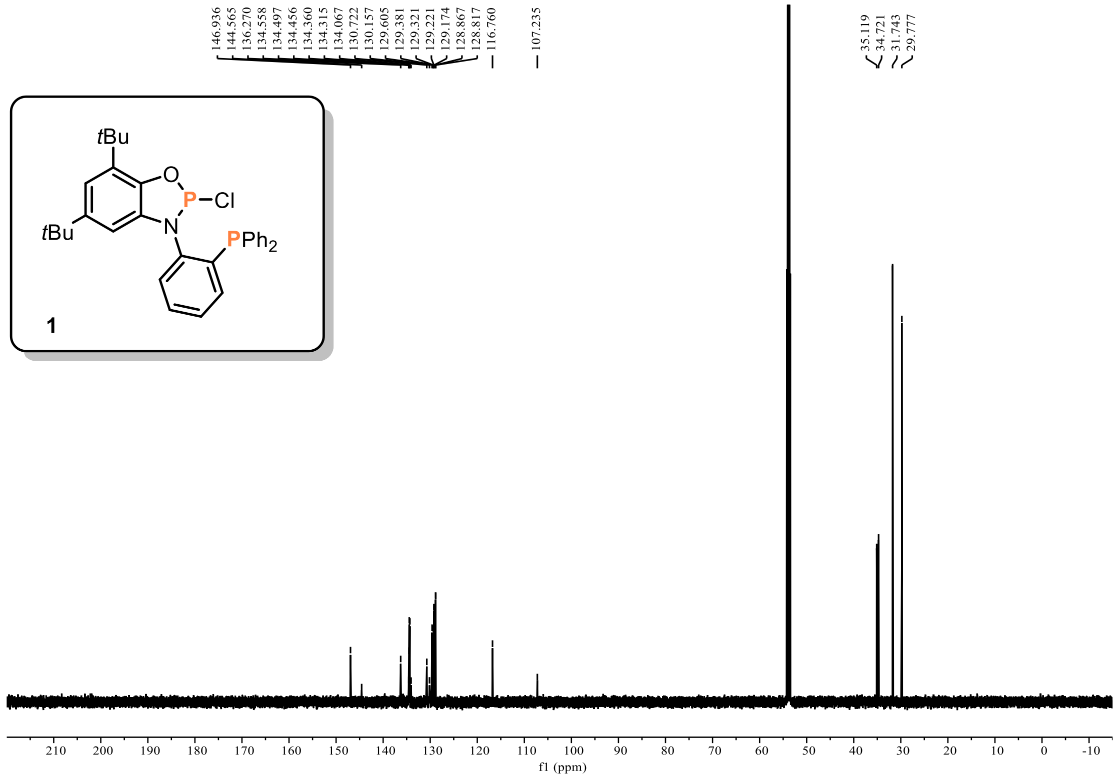

Figure S2: **^{13}C{^{1}H} NMR** of **1** (151 MHz, CD_2Cl_2).

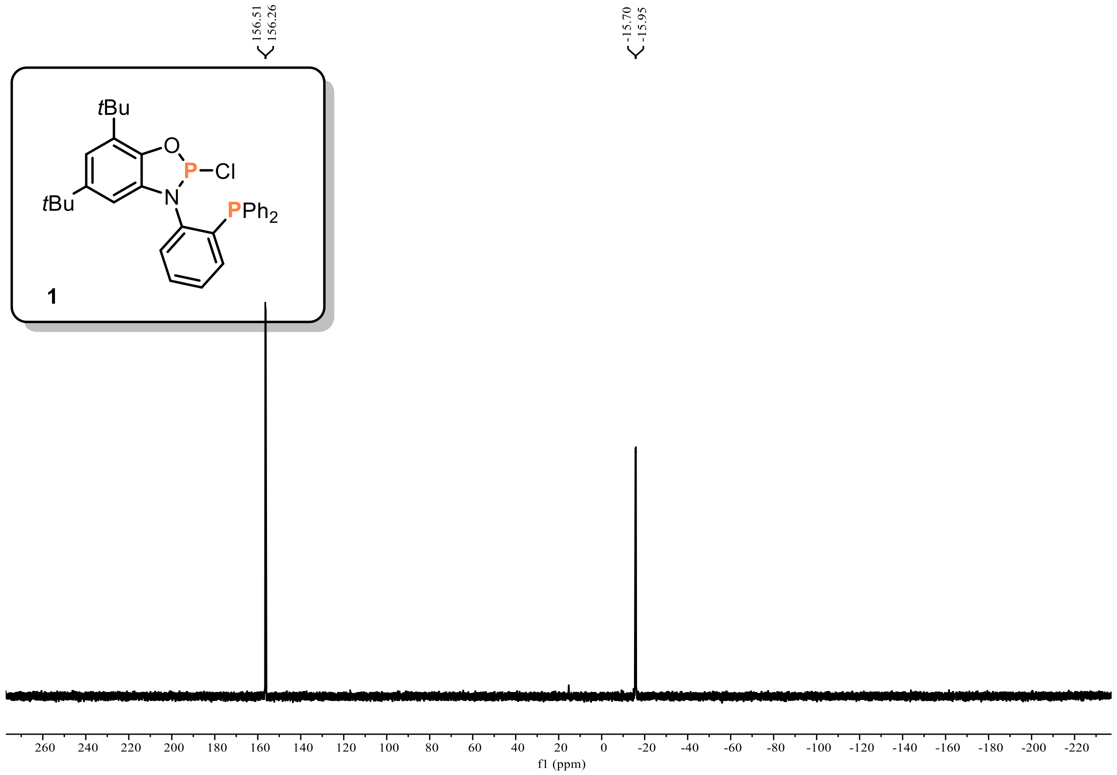

Figure S3: $^{31}P\{^1H\}$ NMR of **1** (243 MHz, CD_2Cl_2).

2.2 Preparation of 2a ([PNOP][B(C_6F_5)$_4$])

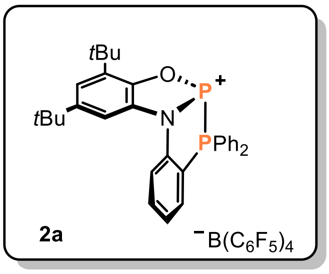

PNOPCl (546 mg, 1.0 mmol, 1.0 equiv) and NaB(C_6F_5)$_4$ (702 mg, 1.0 mmol, 1.0 equiv) were dissolved in CH_2Cl_2 (10 mL). The yellow solution was stirred at room temperature for 3 h, after which the solid was removed by filtration. Removal of all volatiles *in vacuo* furnished the product **2a** as a yellow solid (1.15 g, 0.97 mmol, 97 %).

^{1}H NMR (400 MHz, CD_2Cl_2): δ 8.13 – 7.98 (m, 2H), 7.86 – 7.66 (m, 6H), 7.60 (td, J = 7.5, 1.5 Hz, 1H), 7.51 – 7.42 (m, 1H), 7.32 – 7.21 (m, 2H), 6.97 – 6.88 (m, 3H), 6.85 (d, J = 2.0 Hz, 1H), 1.22 (s, 9H), 1.18 (s, 9H).

^{11}B NMR (128 MHz, CD_2Cl_2): δ -16.7.

^{13}C{^{1}H} NMR (101 MHz, CD_2Cl_2): δ 150.0 (s, 1C), 149.9 – 149.6 (m, $BArF_{20}$), 147.5 – 147.2 (m, $BArF_{20}$), 140.0 – 139.6 (m, $BArF_{20}$), 138.5 – 138.3 (m, $BArF_{20}$), 138.1 – 137.7 (m, $BArF_{20}$), 137.1 (s, 2C), 135.9 (d, J = 3.0 Hz, 1C), 135.6 – 135.4 (m, $BArF_{20}$), 134.9 (d, J = 3.3 Hz, 2C), 134.4 (d, J = 4.0 Hz, 2C), 133.8 (dd, J = 9.5, 2.1 Hz, 1C), 132.9 (d, J = 12.0 Hz, 2C), 131.9 (d, J = 7.5 Hz, 2C), 131.3 (d, J = 12.1 Hz, 1C), 130.6 (d, J = 12.9 Hz, 1C), 128.5 (dd, J = 6.0, 4.3 Hz, 1C), 119.9 (s, 1C), 108.5 (d, J = 3.1 Hz, 1C), 35.4 (s, 1C), 34.9 (s, 1C), 31.5 (s, 3C), 29.6 (s, 3C). 6 carbons could not be reliably assigned due to low intensity.

^{19}F NMR (376 MHz, CD_2Cl_2): δ -130.4 – -139.6 (m), -163.6 (t, J = 20.4 Hz), -167.5 (t, J = 17.9 Hz).

^{31}P{^{1}H} NMR (162 MHz, CD_2Cl_2): δ 157.6 (d, J = 404.9 Hz, O***P***N), 9.6 (d, J = 404.9 Hz, ***P***Ph_2).

HRMS (ESI): [$C_{32}H_{34}NOP_2$]$^+$, calculated: 510.2110, found: 510.2116.

Elem. Anal. for $C_{56}H_{34}BF_{20}NOP_2$ x 0.5 CH_2Cl_2 calculated: C, 55.08; H, 2.86; N,1.14; found C, 55.20; H, 3.13; N,1.29 (CH_2Cl_2 content estimated from **^{1}H NMR** measurements).

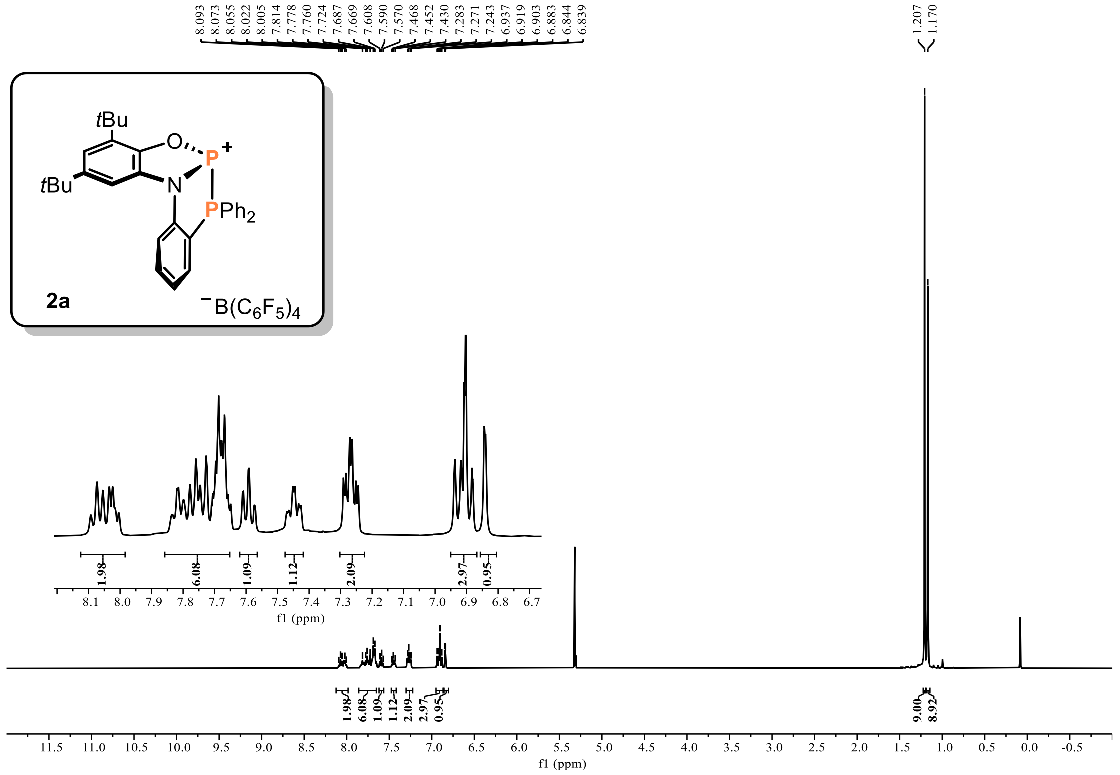

Figure S4: ^{1}H NMR of **2a** (400 MHz, CD_2Cl_2).

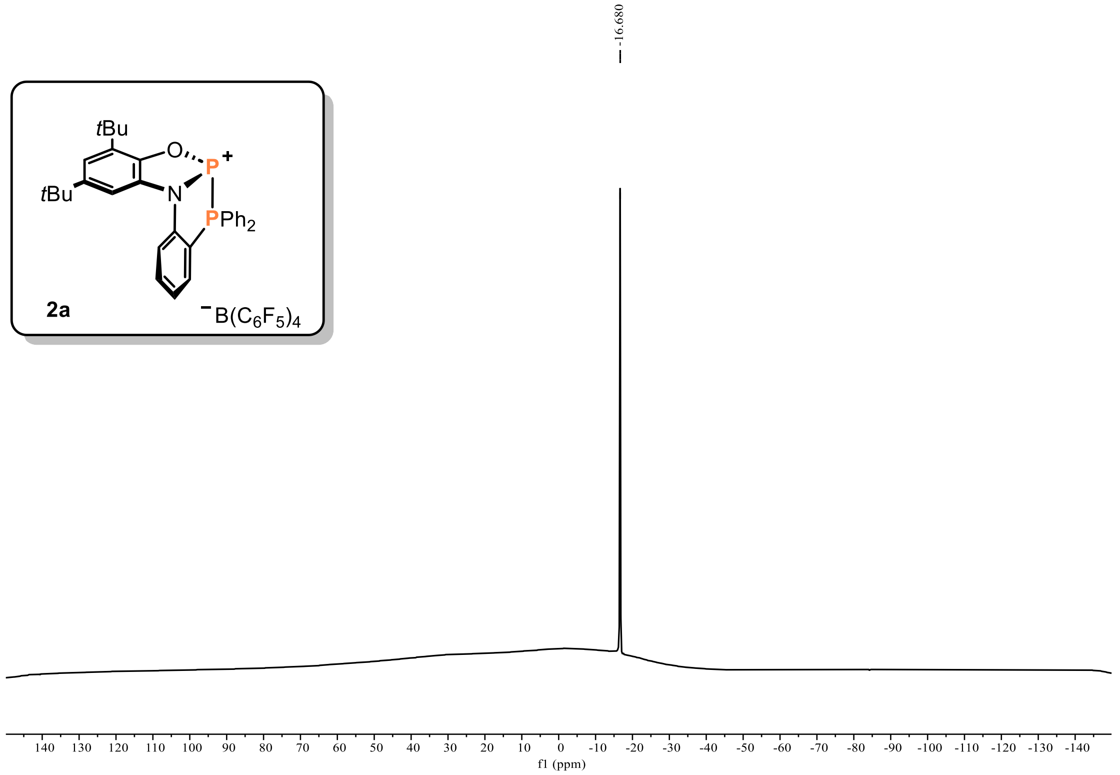

Figure S5: ^{11}B NMR of **2a** (128 MHz, CD_2Cl_2).

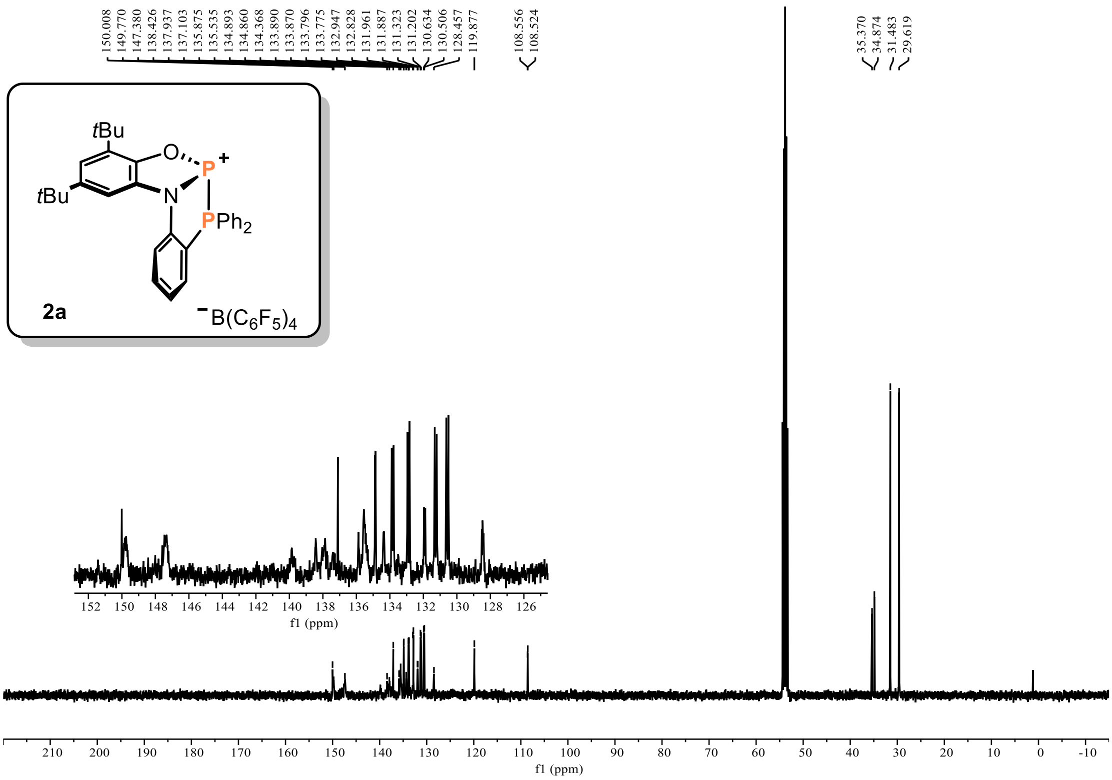

Figure S6: ^{13}**C{**^{1}**H} NMR** of **2a** (101 MHz, CD_2Cl_2).

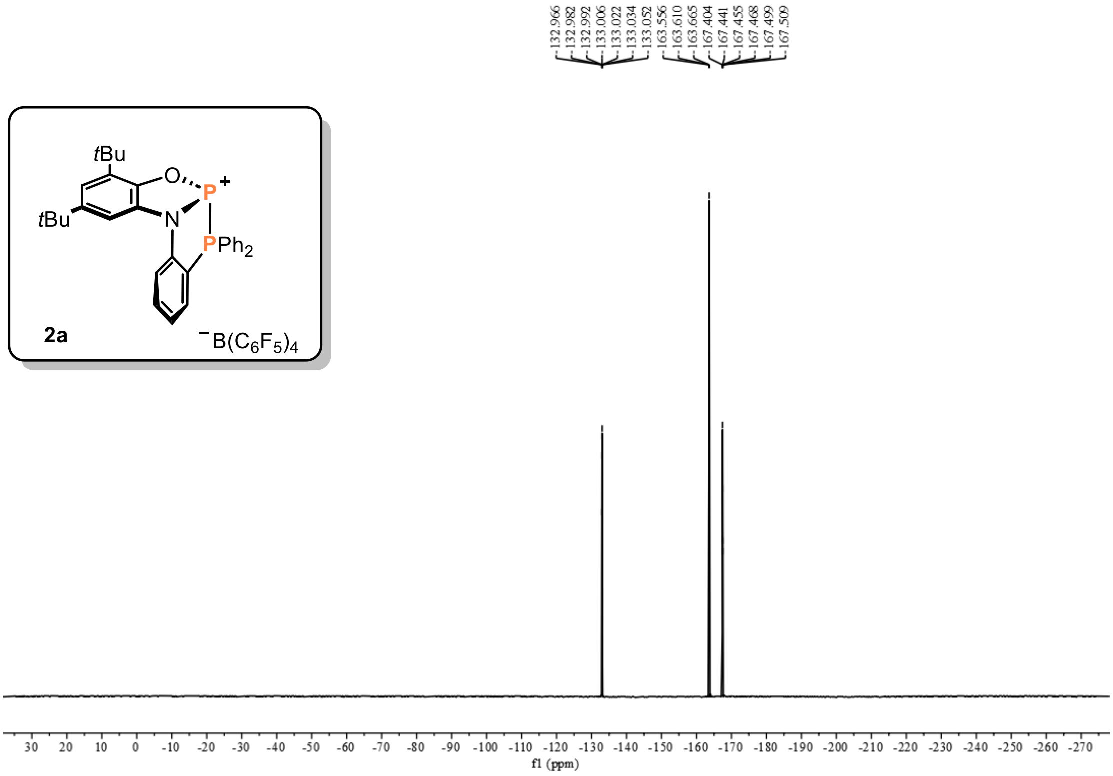

Figure S7: ^{19}**F NMR** of **2a** (376 MHz, CD_2Cl_2).

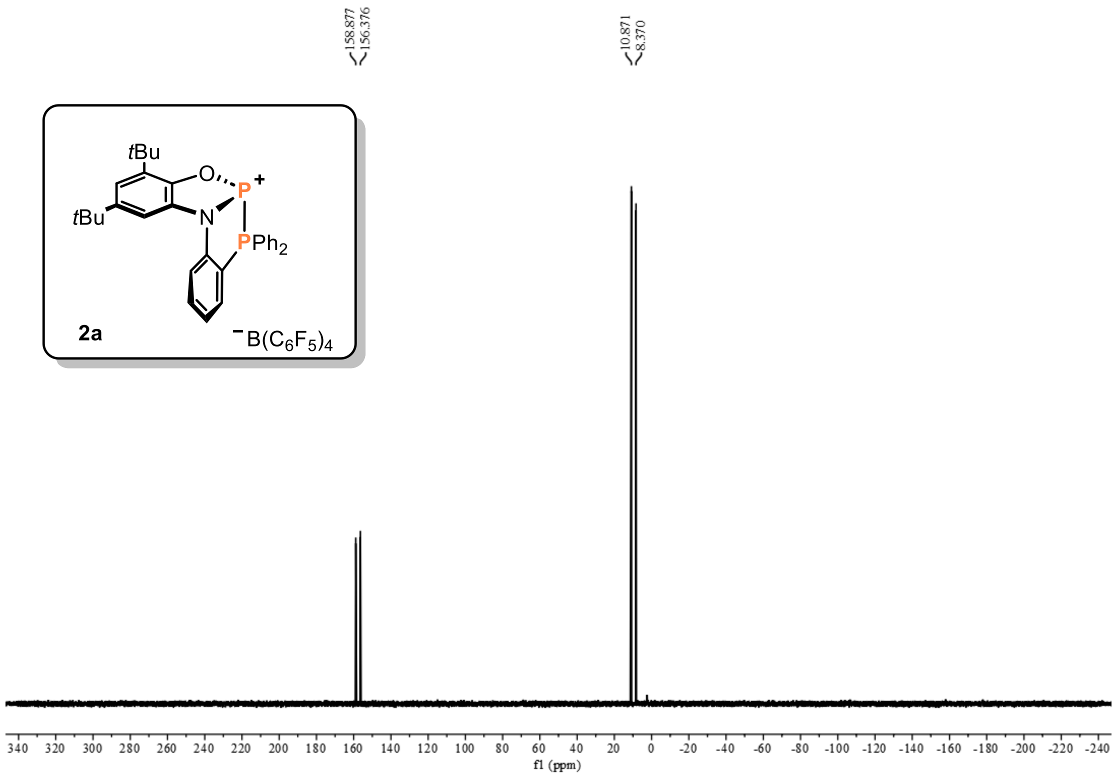

Figure S8: ^{31}P{^{1}H} NMR of **2a** (162 MHz, CD_2Cl_2).

2.3 Preparation of 2b ([PNOP][Al(OC(CF$_3$)$_3$)$_4$])

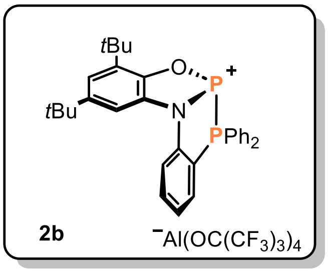

PNOPCl (273 mg, 0.5 mmol, 1.0 equiv) and LiAl(O(CF$_3$)$_3$)$_4$ (487 mg, 0.5 mmol, 1.0 equiv) were dissolved in CH_2Cl_2 (5 mL). The yellow solution was stirred at room temperature for 3 h, after which the solid was removed by filtration. Removal of all volatiles *in vacuo* furnished the product **2b** as a yellow solid (610 mg, 0.41 mmol, 82 %). Single crystals suitable for X-ray diffraction were grown by cooling a concentrated solution in CH_2Cl_2 to -40 °C.

^{1}H NMR (400 MHz, CD_2Cl_2): δ 8.12 (tt, J = 7.8, 1.3 Hz, 1H), 8.05 (dddd, J = 8.1, 4.5, 1.2, 0.5 Hz, 1H), 7.89 – 7.82 (m, 1H), 7.80 – 7.68 (m, 5H), 7.61 (tdd, J = 7.7, 1.5, 0.5 Hz, 1H), 7.52 – 7.44 (m, 1H), 7.34 – 7.27 (m, 2H), 6.94 – 6.92 (m, 2H), 6.92 – 6.89 (m, 1H), 6.86 (dt, J = 2.0, 0.5 Hz, 1H), 1.23 (s, 9H), 1.19 (s, 9H).

^{13}C{^{1}H} NMR (151 MHz, CD_2Cl_2): δ 150.1 (s, 1C), 148.1 (dd, J = 22.8, 4.1 Hz, 1C), 147.6 (d, J = 11.3 Hz, 1C), 138.5 (dd, J = 2.1, 1.0 Hz, 1C), 137.2 (s, 2C), 135.9 (dd, J = 3.1, 0.9 Hz, 1C), 135.7 (d, J = 3.5 Hz, 1C), 134.9 (d, J = 3.3 Hz, 2C), 134.5 (d, J = 4.2 Hz, 2C), 133.8 (dd, J = 9.3, 2.1 Hz, 1C), 132.9 (d, J = 11.9 Hz, 2C), 132.0 (d, J = 7.5 Hz, 2C), 131.4 (d, J = 12.3 Hz, 1C), 130.6 (d, J = 13.0 Hz, 1C), 128.5 (dd, J = 6.1, 4.2 Hz, 1C), 121.7 (q, J = 295 Hz, OC(***C***F$_3$)$_4$), 120.0 (s, 1C), 118.3 (d, J = 0.8 Hz, 1C), 117.9 (d, J = 1.0 Hz, 1C), 108.6 (d, J = 3.2 Hz, 1C), 35.4 (s, 1C), 34.9 (s, 1C), 31.5 (s, 3C), 29.6 (s, 3C).

^{19}F NMR (565 MHz, CD_2Cl_2): δ -75.7.

^{31}P{^{1}H} NMR (243 MHz, CD_2Cl_2): δ 157.6 (d, J = 405.2 Hz, O***P***N), 9.6 (d, J = 405.2 Hz, ***P***Ph$_2$).

HRMS (ESI): $[C_{32}H_{34}NOP_2]^+$, calculated: 510.2110, found: 510.2117.

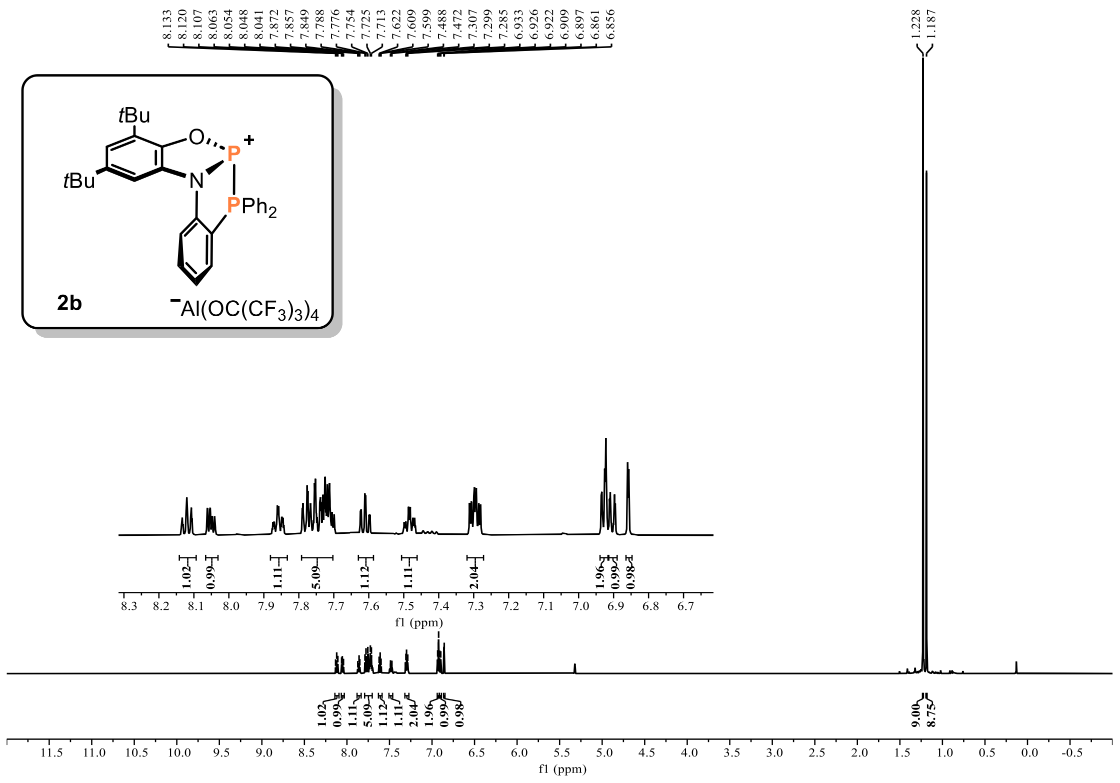

Figure S9: ^{1}H NMR of **2b** (600 MHz, CD_2Cl_2).

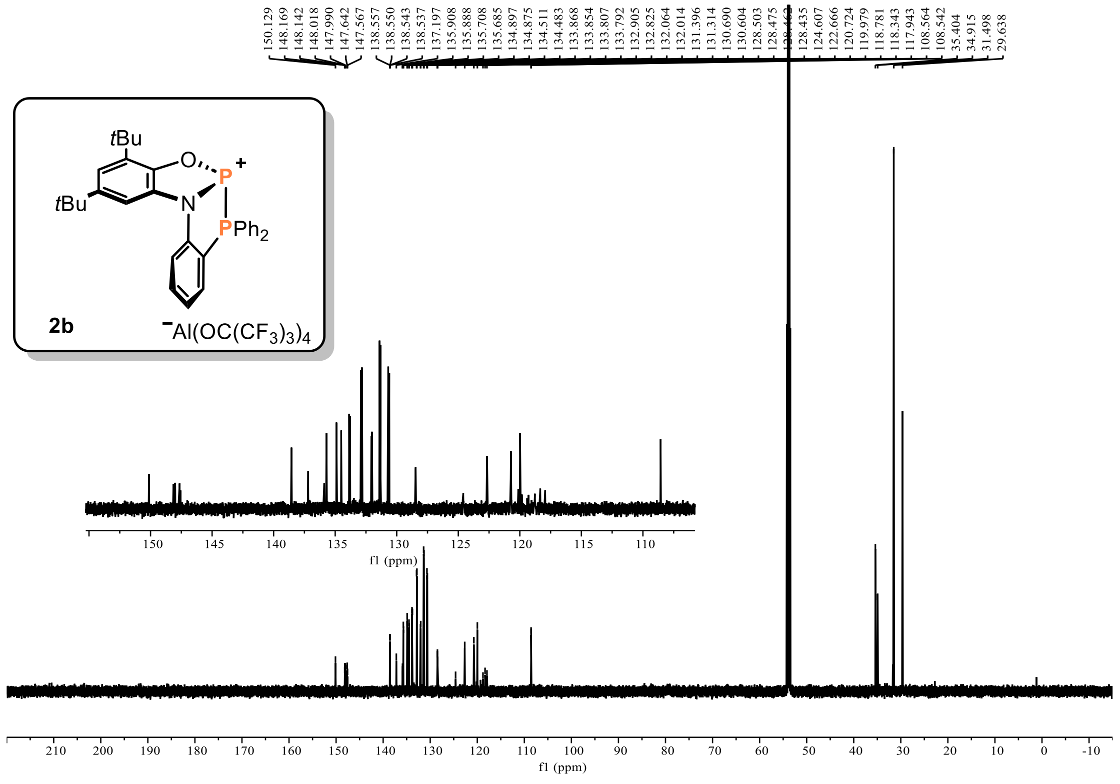

Figure S10: ^{13}C{^{1}H} NMR of **2b** (151 MHz, CD_2Cl_2).

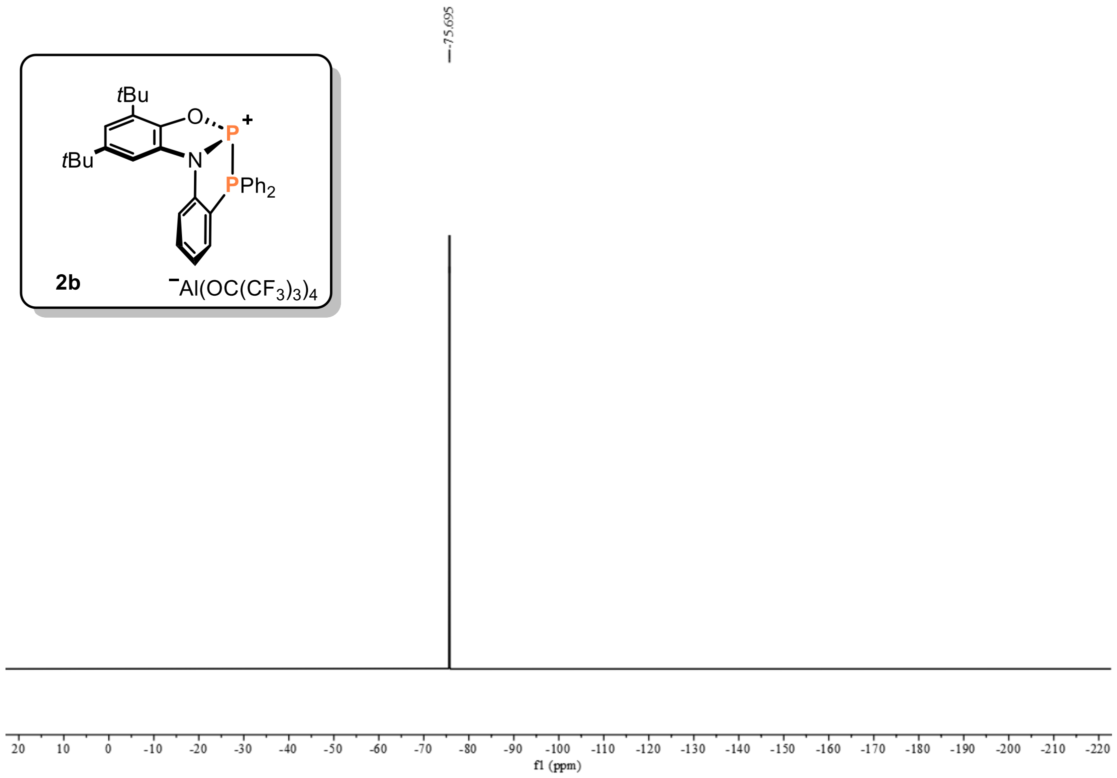

Figure S11: ^{19}**F NMR** of **2b** (565 MHz, CD_2Cl_2).

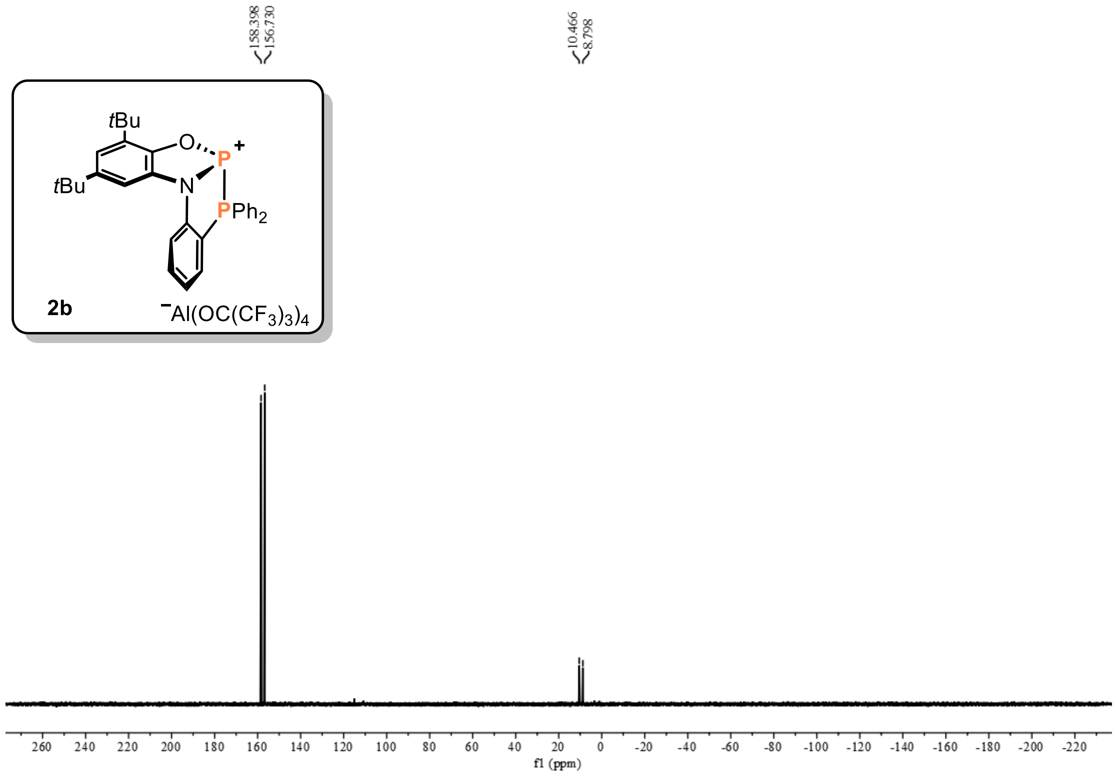

Figure S12: ^{31}**P{**^{1}**H} NMR** of **2b** (243 MHz, CD_2Cl_2).

3. Phosphinophosphination of Alkynes

3.1 Possible diastereomers for the reaction product

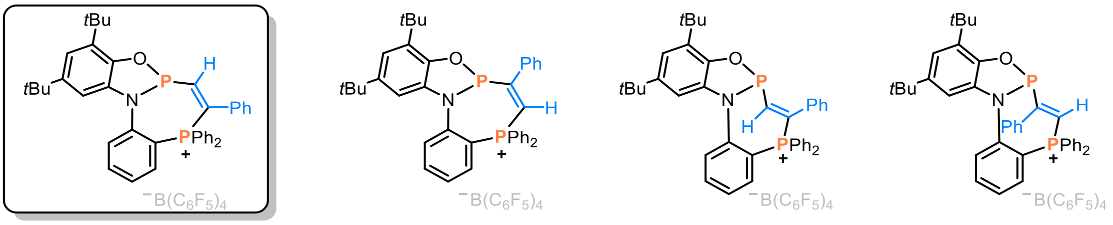

I **II** **III** **IV**

Four possible diastereomers can form for the reaction product of [**PNOP**][**B(C_6F_5)$_4$**] **2a** with phenylacetylene. Only the *cis*-addition isomer (**I**) with phenyl group and cationic P on the same side formed, which was confirmed by single crystals XRD.

3.2 Preparation of 3

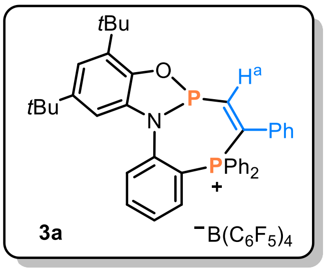

2a (300 mg, 252 µmol, 1.0 equiv) was dissolved in 5 mL CH_2Cl_2, followed by the addition of phenylacetylene (25.7 mg, 252 µmol, 1.0 equiv). The mixture was stirred at room temperature for 2 h. Removal of all volatiles *in vacuo* furnished the product **3a** as an orange solid (294 mg, 227 µmol, 90 %). Single crystals suitable for X-ray diffraction were grown by gas phase diffusion of pentane into the CH_2Cl_2 solution.

^{1}H NMR (600 MHz, CD_2Cl_2): δ 8.03 (dd, J = 47.4, 25.2 Hz, C=CP$\boldsymbol{H}^{a}$), 7.94 – 7.88 (m, 1H), 7.73 (dd, J = 8.1, 4.9 Hz, 1H), 7.68 (tdd, J = 7.5, 2.2, 1.1 Hz, 1H), 7.62 (td, J = 7.4, 1.7 Hz, 1H), 7.52 (td, J = 7.8, 3.9 Hz, 1H), 7.49 – 7.37 (m, 8H), 7.35 (ddd, J = 13.4, 8.0, 1.4 Hz, 1H), 7.16 (td, J = 7.5, 1.3 Hz, 1H), 7.07 (t, J = 7.7 Hz, 2H), 6.93 (dt, J = 8.5, 1.4 Hz, 1H), 6.87 (d, J = 2.0 Hz, 1H), 5.58 (d, J = 2.0 Hz, 1H), 1.42 (s, 9H), 1.06 (s, 9H).

^{11}B NMR (193 MHz, CD_2Cl_2): δ -16.7.

^{13}C{^{1}H} NMR (151 MHz, CD_2Cl_2): δ 165.4 (dd, J = 66.9, 14.1 Hz, 1C), 149.5 – 149.2 (m, $BArF_{20}$), 147.9 – 147.6 (m, $BArF_{20}$), 146.4 (s, 1C), 146.3 (d, J = 13.1 Hz, 1C), 143.5 (dd, J = 19.0, 4.4 Hz), 139.7 – 139.2 (m, $BArF_{20}$), 138.7 (d, J = 2.8 Hz, 1C), 138.0 – 137.7 (m, $BArF_{20}$), 137.6 – 137.4 (m, $BArF_{20}$), 137.2 (d, J = 13.4 Hz, 1C), 136.7 (d, J = 1.8 Hz, 1C), 136.3 (d, J = 9.5 Hz, 1C), 136.0 – 135.8 (m, $BArF_{20}$), 135.7 (d, J = 3.1 Hz, 2C), 135.2 (d, J = 2.9 Hz, 2C), 134.2 (d, J = 9.3 Hz, 2C), 134.0 (d, J = 2.9 Hz, 1C), 133.9 (d, J = 11.2 Hz, 2C), 130.6 (dd, J = 20.4, 13.1 Hz, 1C), 130.1 (d, J = 1.8 Hz, 1C), 129.3 (s, 1C), 129.2 (s, 2C), 129.1 (dd, J = 4.5, 2.3 Hz, 1C), 127.7 (d, J = 3.0 Hz, 2C), 127.2 (d, J = 2.8 Hz, 2C), 120.0 (dd, J = 88.6, 4.3 Hz, 1C), 118.0 (s, 1C), 117.9 (d, J = 1.8 Hz, 1C), 117.3 (d, J = 1.4 Hz, 1C), 105.2 (s, 1C), 35.0 (s, 1C), 34.9 (s, 1C), 31.6 (s, 3C), 29.6 (s, 3C).

^{19}F NMR (565 MHz, CD_2Cl_2): δ -133.0 – -133.1 (m), -163.6 (t, J = 20.3 Hz), -167.5 (t, J = 19.4 Hz).

^{31}P{^{1}H} NMR (243 MHz, CD_2Cl_2): δ 145.5 (d, J = 4.7 Hz, O***P***N), 24.5 (d, J = 4.7 Hz, ***P***Ph_2).

HRMS (ESI): $[C_{40}H_{40}NOP_2]^+$, calculated: 612.2580, found: 612.2585.

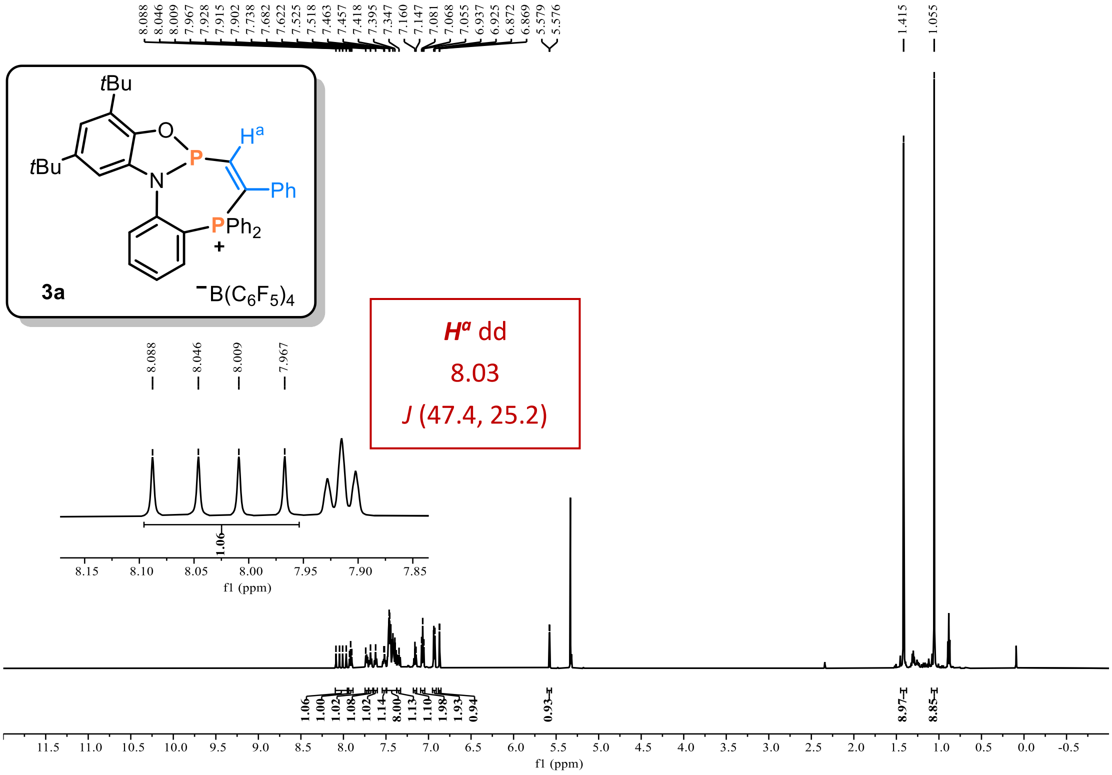

Figure S13: **¹H NMR** of **3a** (600 MHz, CD_2Cl_2).

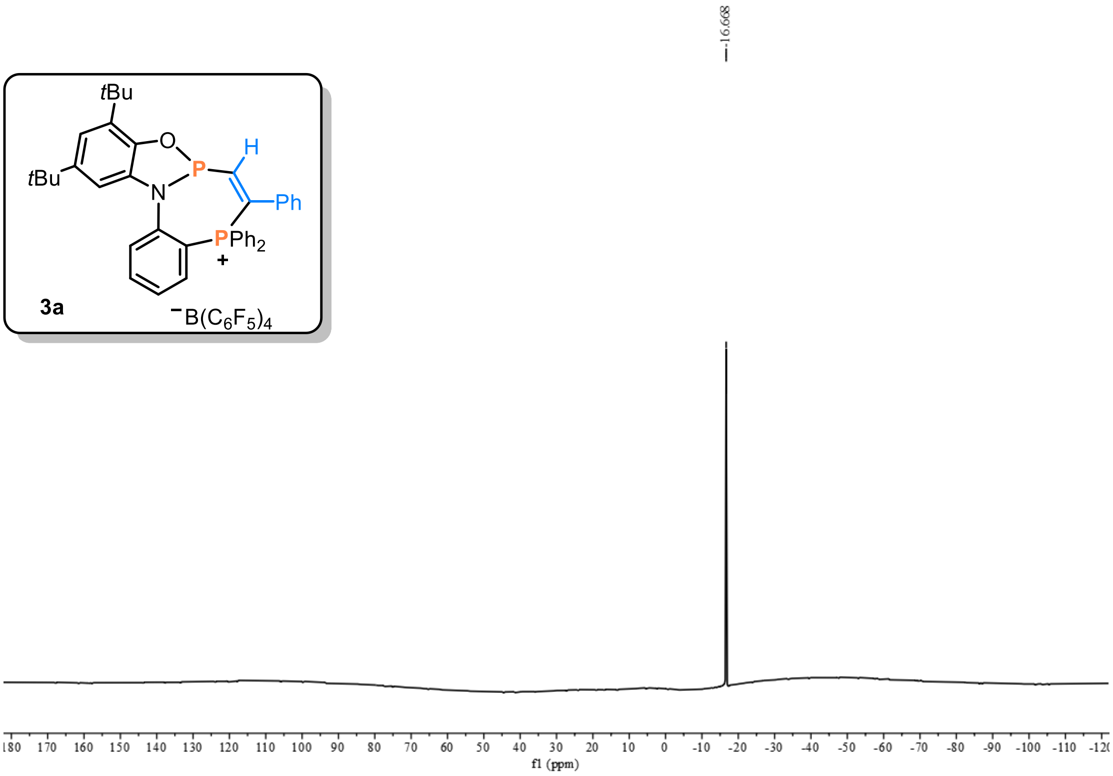

Figure S14: **¹¹B NMR** of **3a** (193 MHz, CD_2Cl_2).

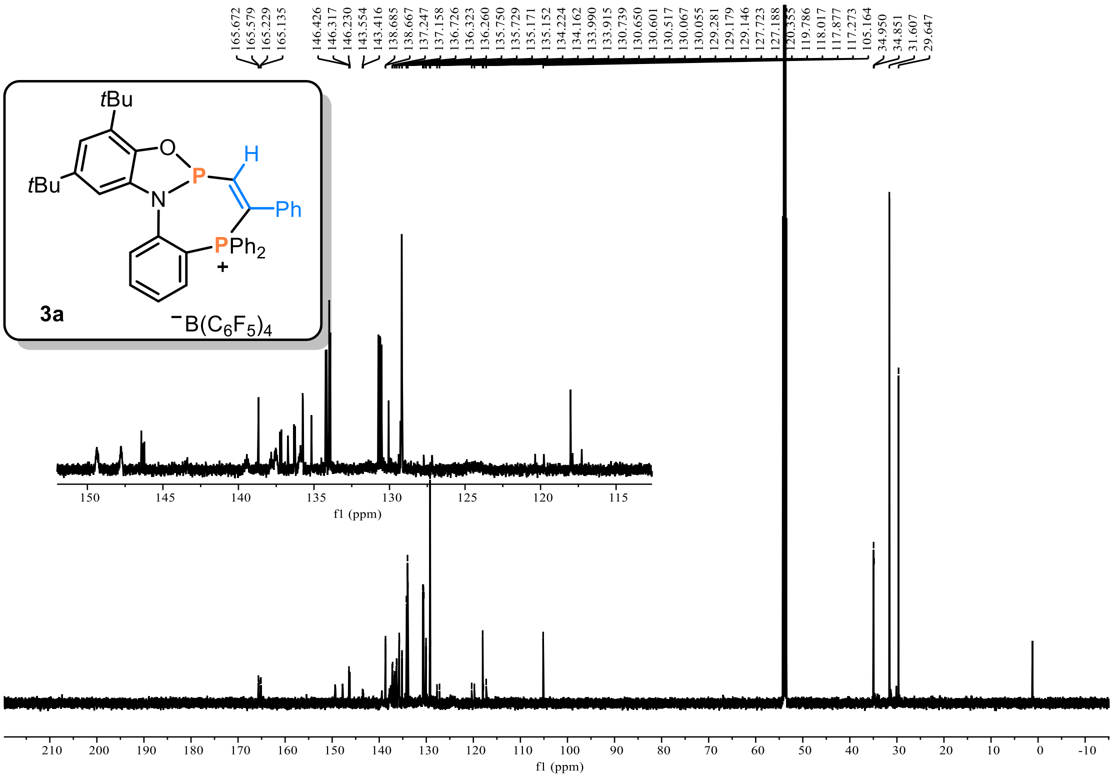

Figure S15: ^{13}**C NMR** of **3a** (151 MHz, CD_2Cl_2).

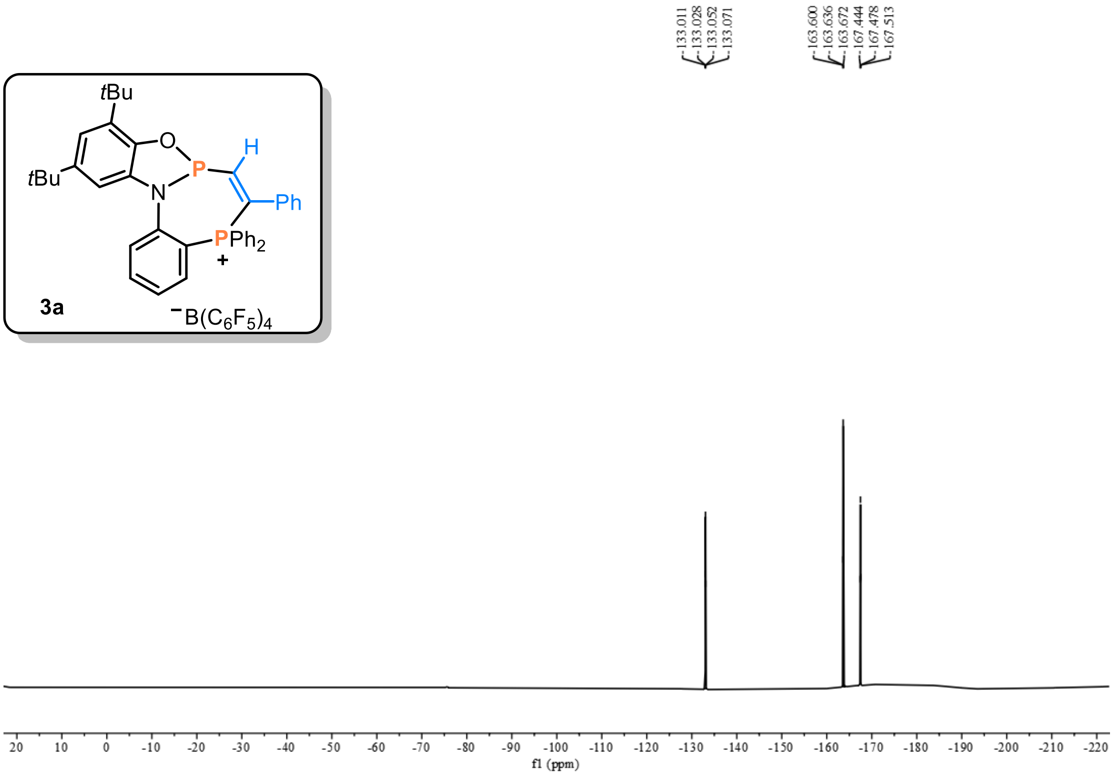

Figure S16: ^{19}**F NMR** of **3a** (565 MHz, CD_2Cl_2).

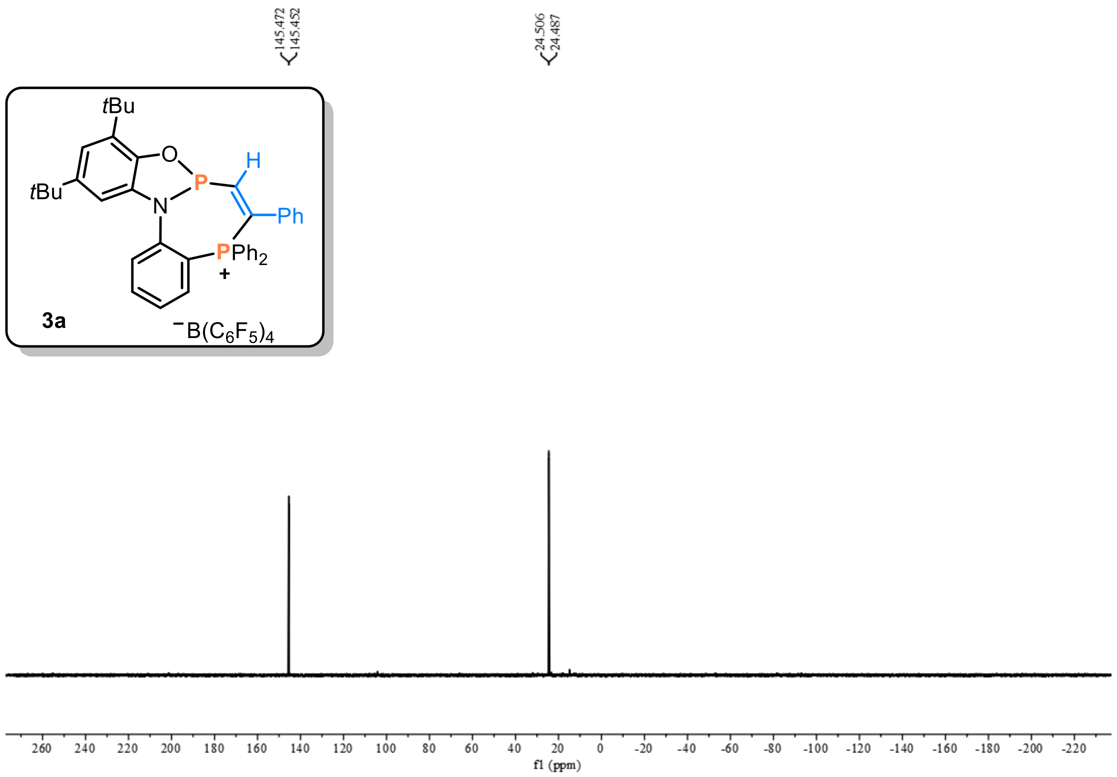

Figure S17: ^{31}**P{**^{1}**H} NMR** of **3a** (243 MHz, CD_2Cl_2).

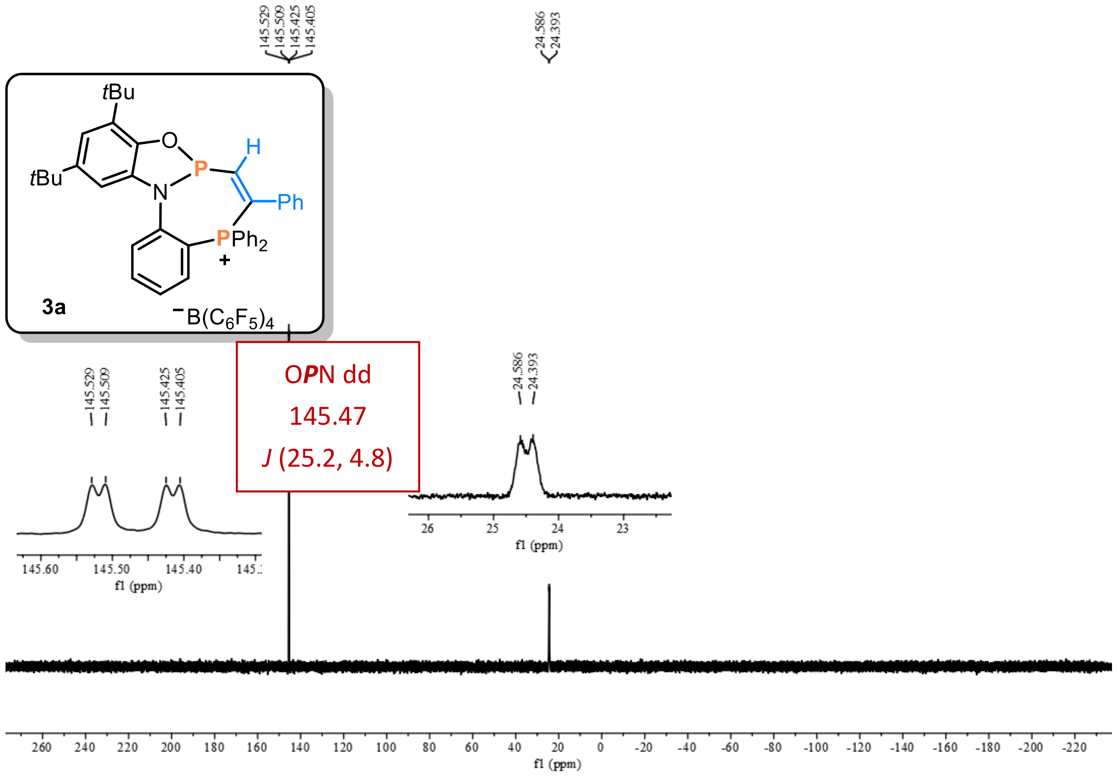

Figure S18: ^{31}**P NMR** of **3a** (243 MHz, CD_2Cl_2).

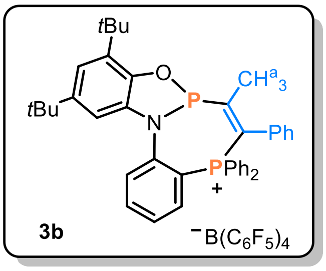

2a (100 mg, 84 µmol, 1.0 equiv) was dissolved in 2 mL CH_2Cl_2, followed by the addition of 1-phenyl-1-propyne (9.8 mg, 84 µmol, 1.0 equiv). The mixture was stirred at 60 °C for 16 h. The solvent was then removed under reduced pressure and the solid was washed with pentane and dried *in vacuo* to give the product **3b** as an orange solid (91.5 mg, 70 µmol, 83 %). Single crystals suitable for X-ray diffraction were grown by gas phase diffusion of pentane into the CH_2Cl_2 solution.

^{1}H NMR (400 MHz, CD_2Cl_2): δ 7.91 – 7.81 (m, 1H), 7.75 – 7.61 (m, 3H), 7.60 – 7.50 (m, 2H), 7.49 – 7.37 (m, 6H), 7.37 – 7.28 (m, 2H), 7.14 – 7.06 (m, 1H), 7.03 (td, *J* = 7.5, 1.5 Hz, 2H), 6.97 (d, *J* = 2.0 Hz, 1H), 6.64 (s, 2H), 5.76 (d, *J* = 2.0 Hz, 1H), 2.18 (dd, *J* = 6.8, 3.2 Hz, 3H, C=CPC$\boldsymbol{H}^a_3$), 1.46 (s, 9H), 1.10 (s, 9H).

^{11}B NMR (128 MHz, CD_2Cl_2): δ -16.7.

^{13}C{^{1}H} NMR (101 MHz, CD_2Cl_2): δ 176.3 (dd, *J* = 61.2, 14.1 Hz, 1C), 149.9 – 149.4 (m, $BArF_{20}$), 147.5 – 147.1 (m, $BArF_{20}$), 146.6 (s, 1C), 146.2 (d, *J* = 13.0 Hz, 1C), 143.9 (dd, *J* =19.7, 6.4 Hz, 1C), 140.0 – 139.6 (m, $BArF_{20}$), 138.1 (d, *J* = 2.6 Hz, 1C), 138.1 – 137.7 (m, $BArF_{20}$), 137.6 – 137.2 (m, $BArF_{20}$), 136.7 (s, 1C), 135.7 (d, *J* = 8.3 Hz, 2C), 135.6 – 135.3 (m, $BArF_{20}$), 135.2 (d, *J* =3.1 Hz, 2C), 135.0 (d, *J* =2.9 Hz, 2C), 134.4 (d, *J* = 9.1 Hz, 2C), 134.1 (d, *J* = 13.5 Hz, 1C), 133.9 (d, *J* = 11.0 Hz, 2C), 133.8 (d, *J* = 3.0 Hz, 1C), 130.4 (dd, *J* = 15.2, 13.0 Hz, 1C), 129.5 (s, 1C), 129.4 (d, *J* = 2.7 Hz, 1C), 129.2 (d, *J* = 12.1 Hz, 1C), 127.8 (dd, *J* = 8.3, 2.5 Hz, 1C), 127.3 (d, *J* = 4.4 Hz, 1C), 126.5 (d, *J* = 4.4 Hz, 1C), 123.0 (dd, *J* = 77.3, 16.1 Hz, 1C), 120.8 (d, *J* = 3.3 Hz, 1C), 119.9 (d, *J* = 3.0 Hz, 1C), 118.5 (d, *J* = 2.1 Hz, 1C), 118.5 (s, 1C), 117.6 (d, *J* = 2.1 Hz, 1C), 106.3 (s, 1C), 35.0 (s, 1C), 35.0 (s, 1C), 31.6 (s, 3C), 29.8 (s, 3C), 18.7 (dd, *J* = 18.7, 14.5 Hz, 1C).

^{19}F NMR (376 MHz, CD_2Cl_2): δ -132.9 – -133.1 (m), -163.6 (t, *J* = 20.4 Hz), -167.5 (t, *J* = 19.6 Hz).

^{31}P{^{1}H} NMR (162 MHz, CD_2Cl_2): δ 137.5 (d, *J* = 2.9 Hz, O***P***N), 23.4 (d, *J* = 2.9 Hz, ***P***Ph_2).

HRMS (ESI): $[C_{41}H_{42}NOP_2]^+$, calculated: 626.2736, found: 626.2741.

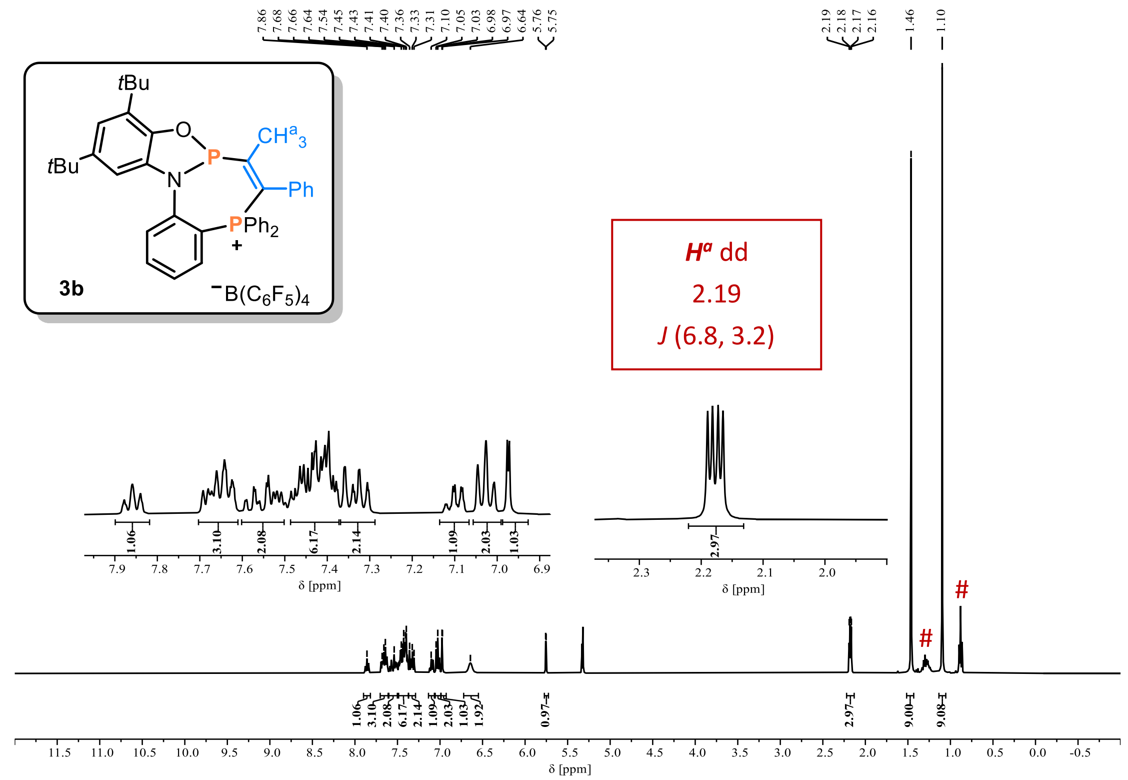

Figure S19: **^{1}H NMR** of **3b** (400 MHz, CD_2Cl_2). # denotes pentane.

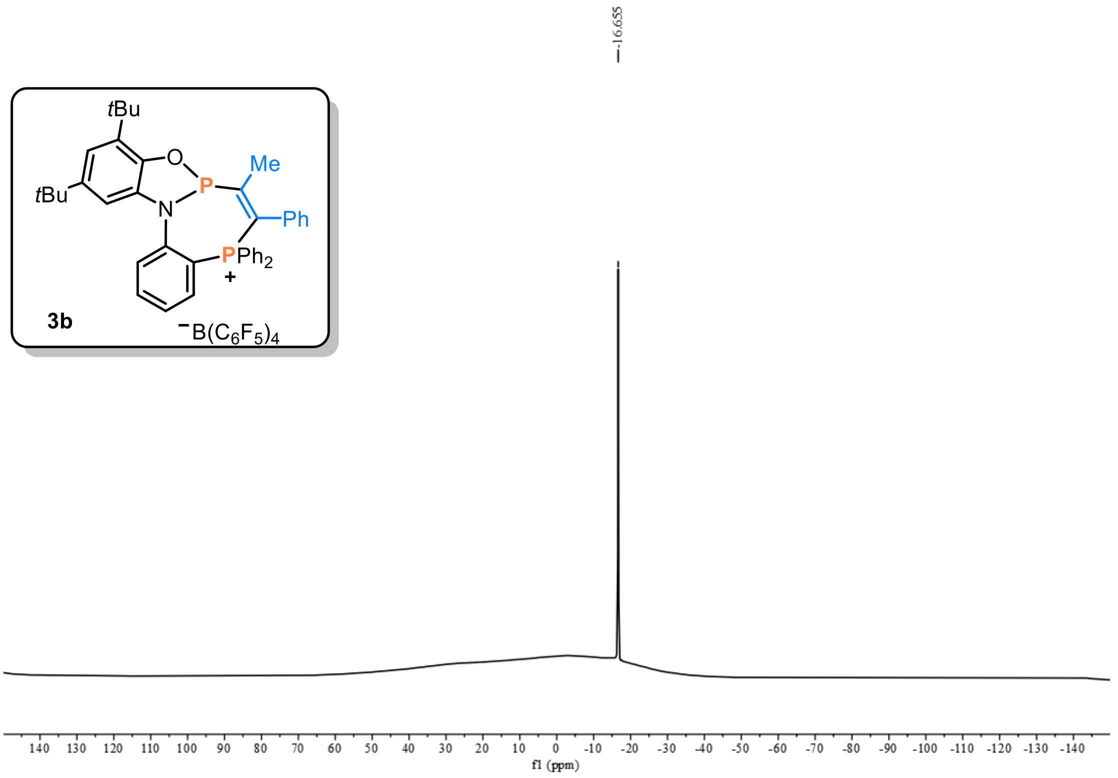

Figure S20: **^{11}B NMR** of **3b** (128 MHz, CD_2Cl_2).

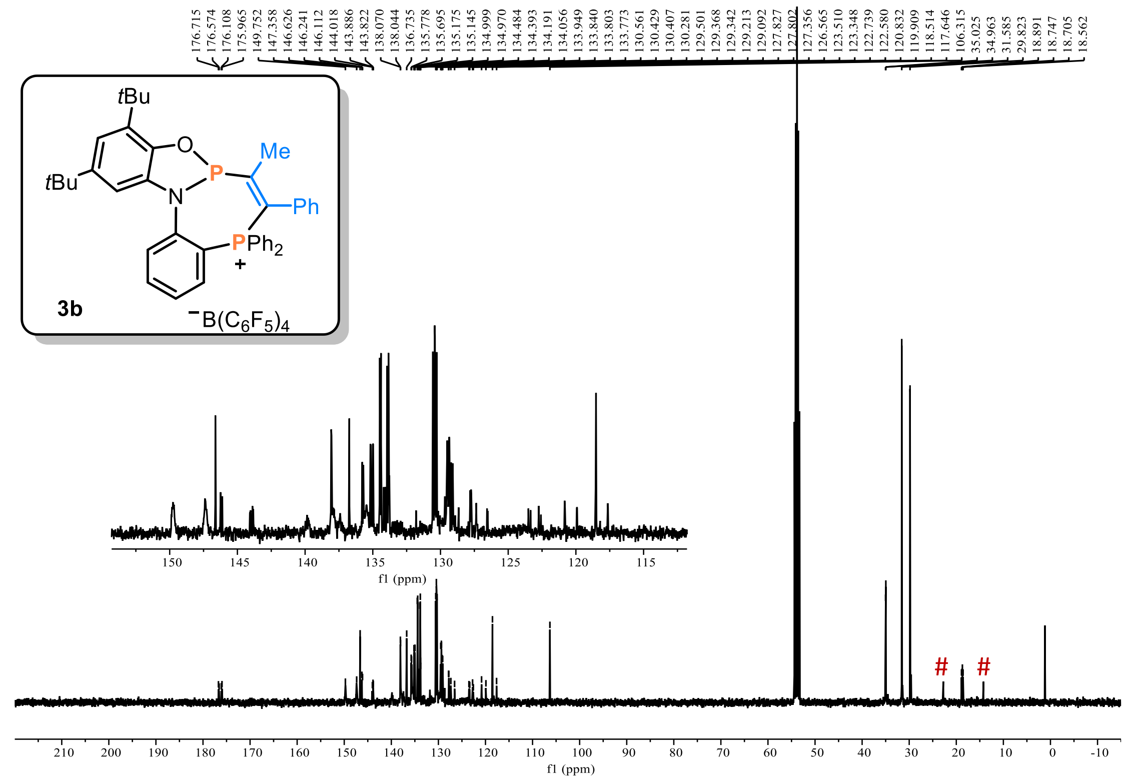

Figure S21: ^{13}**C{**^{1}**H} NMR** of **3b** (101 MHz, CD_2Cl_2). # denotes pentane.

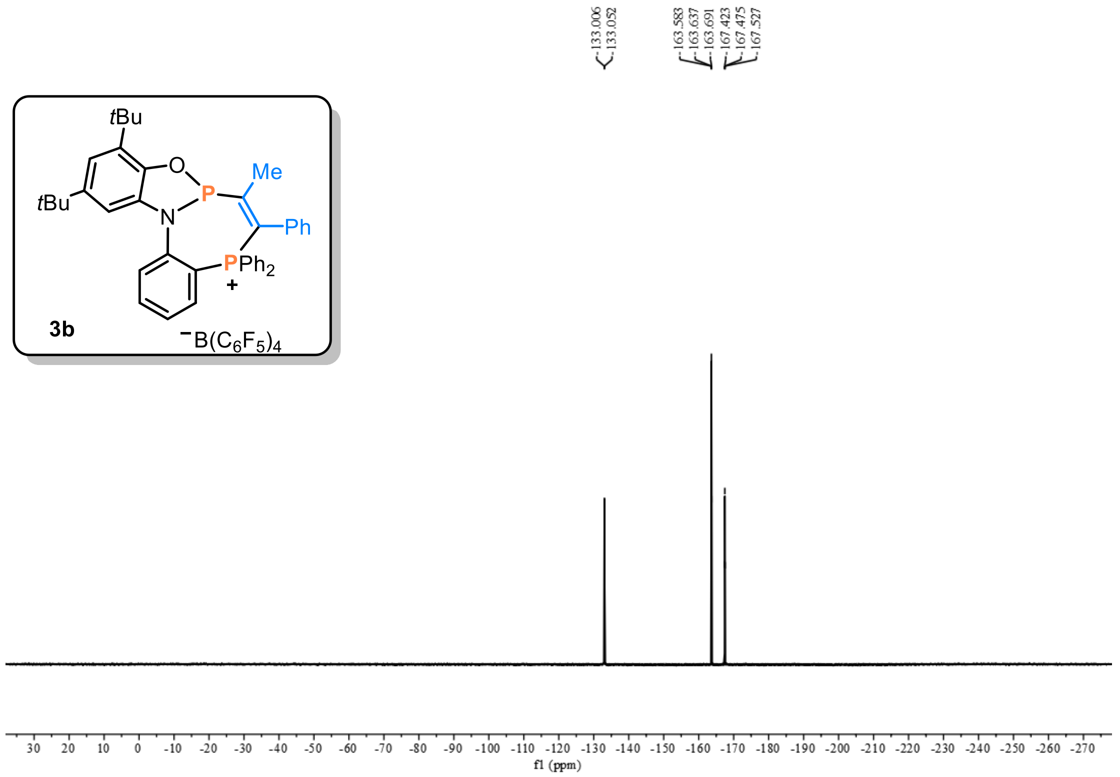

Figure S22: ^{19}**F NMR** of **3b** (376 MHz, CD_2Cl_2).

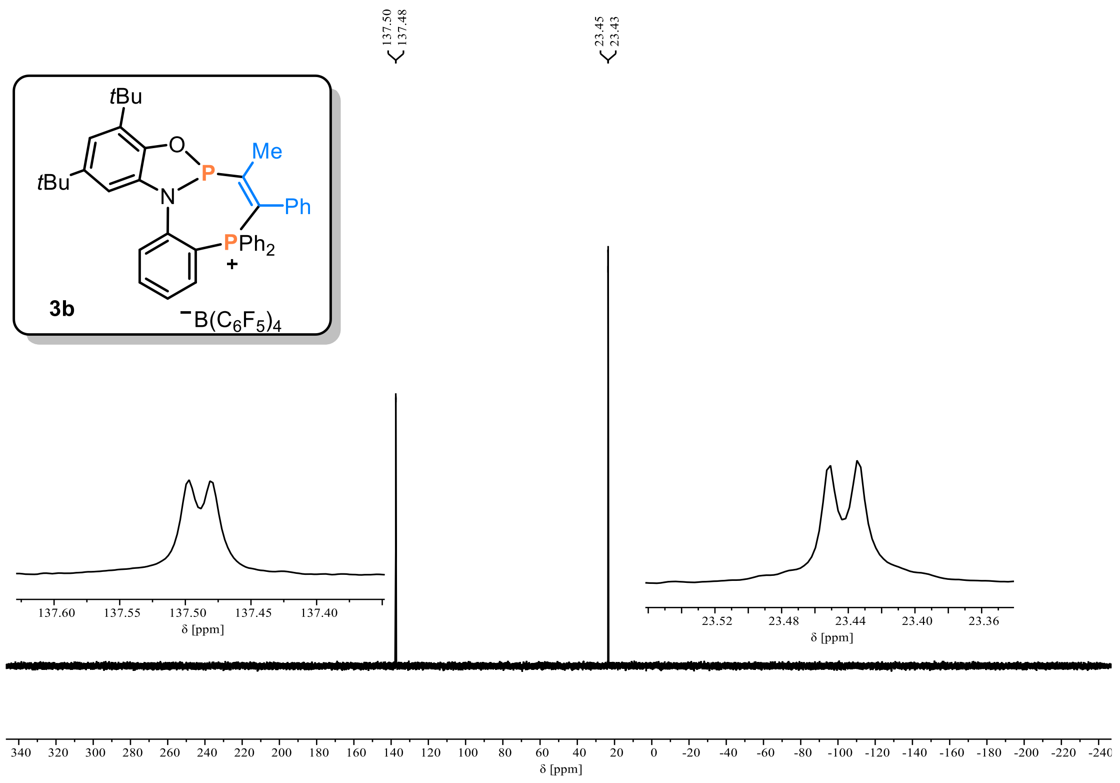

Figure S23: ^{31}**P{**^{1}**H} NMR** of **3b** (162 MHz, CD_2Cl_2).

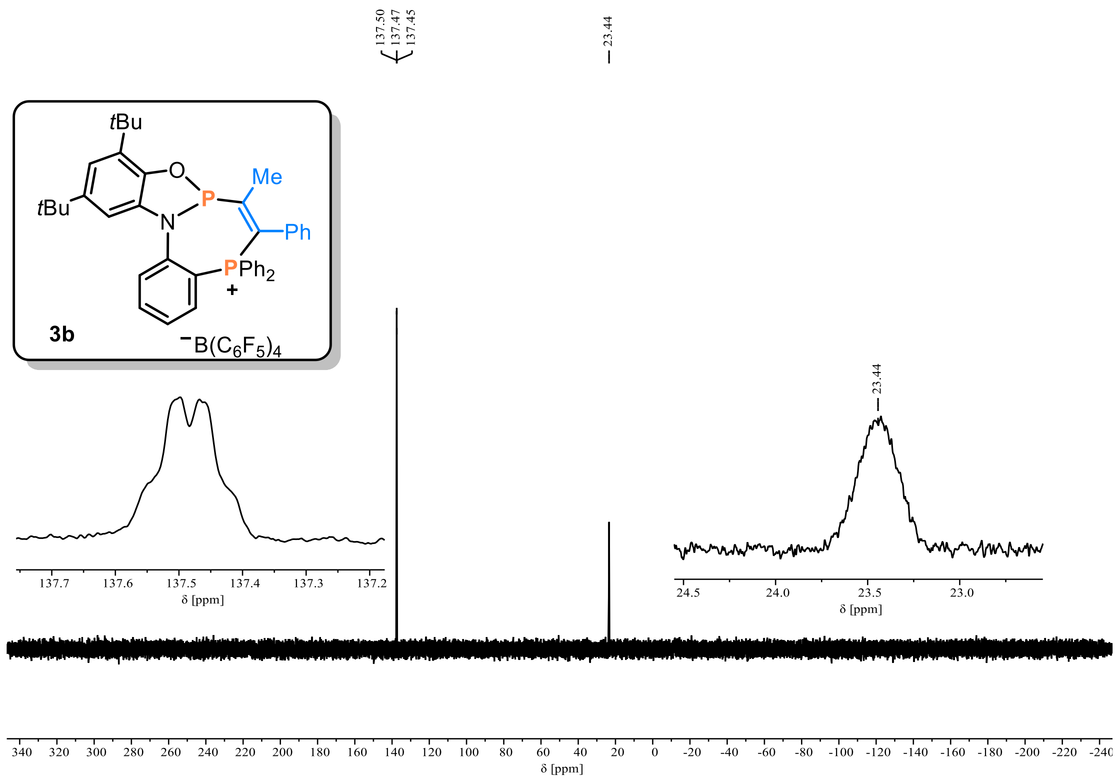

Figure S24: ^{31}**P NMR** of **3b** (162 MHz, CD_2Cl_2).

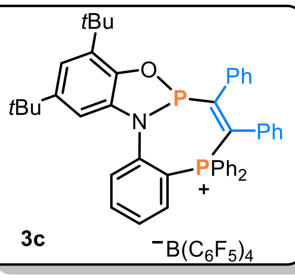

3c

2a (100 mg, 84 µmol, 1.0 equiv) was dissolved in 2 mL toluene, followed by the addition of diphenylacetylene (15 mg, 84 µmol, 1.0 equiv). The mixture was stirred at 110 °C for 48 h. The solvent was then removed under reduced pressure and the solid was washed with pentane and dried *in vacuo* to give the product **3c** as an orange solid (78.8 mg, 58 µmol, 69 %).

^{1}H NMR (400 MHz, CD_2Cl_2): δ 7.97 – 7.85 (m, 1H), 7.75 – 7.65 (m, 2H), 7.59 – 7.48 (m, 8H), 7.45 – 7.36 (m, 5H), 7.31 (ddd, J = 4.5, 3.1, 1.9 Hz, 3H), 7.23 (dd, J = 7.5, 2.2 Hz, 2H), 6.92 – 6.85 (m, 2H), 6.84 – 6.77 (m, 2H), 5.60 (d, J = 2.0 Hz, 1H), 1.10 (s, 9H), 1.07 (s, 9H).

^{11}B NMR (128 MHz, CD_2Cl_2): δ -16.6.

^{13}C{^{1}H} NMR (101 MHz, CD_2Cl_2): δ 177.9 (dd, J = 69.1, 15.2 Hz, 1C), 149.9 – 149.5 (m, $BArF_{20}$), 147.4 – 147.1 (m, $BArF_{20}$), 146.4 (s, 1C), 145.5 (d, J = 13.7 Hz, 1C), 143.5 (dd, J = 19.3, 6.2 Hz, 1C), 140.0 – 139.7 (m, $BArF_{20}$), 138.4 (d, J = 2.8 Hz, 1C), 138.1 – 137.7 (m, $BArF_{20}$), 137.6 – 137.2 (m, $BArF_{20}$), 136.7 (s, 1C), 136.0 (d, J = 8.4 Hz, 1C), 135.8 (s, 1C), 135.6 (d, J = 4.3 Hz, 2C), 135.5 (d, J = 3.1 Hz, 2C), 135.5 – 135.2 (m, $BArF_{20}$), 134.9 (d, J = 13.4 Hz, 2C), 134.8 (d, J = 3.4 Hz, 2C), 134.2 (dd, J = 44.4, 9.0 Hz, 1C), 134.1 (d, J = 3.0 Hz, 2C), 131.9 (s, 1C), 130.5 (dd, J = 49.9, 13.1 Hz, 1C), 129.6 (s, 2C), 129.5 (s, 2C), 129.3 (s, 1C), 128.9 (d, J = 2.4 Hz, 2C), 128.8 (dd, J = 5.3, 2.2 Hz, 1C), 128.4 (dd, J = 8.4, 2.5 Hz, 1C), 128.3 (d, J = 6.3 Hz, 1C), 123.6 (dd, J = 78.1, 17.7 Hz, 1C), 121.3 (d, J = 3.8 Hz, 1C), 120.4 (d, J = 4.0 Hz, 1C), 118.5 (s, 1C), 118.1 (d, J = 1.7 Hz, 1C), 117.2 (d, J = 1.7 Hz, 1C), 105.7 (s, 1C), 35.0 (s, 1C), 34.6 (s, 1C), 31.6 (s, 3C), 29.3 (s, 3C).

^{19}F NMR (376 MHz, CD_2Cl_2): δ -132.9 – -133.1 (m), -163.6 (t, J = 20.4 Hz), -167.5 (t, J = 19.5 Hz).

^{31}P{^{1}H} NMR (162 MHz, CD_2Cl_2): δ 137.9 (d, J = 9.0 Hz, O***P***N), 23.6 (d, J = 9.0 Hz, ***P***Ph_2).

HRMS (ESI): $[C_{46}H_{44}NOP_2]^+$, calculated: 688.2893, found: 688.2894.

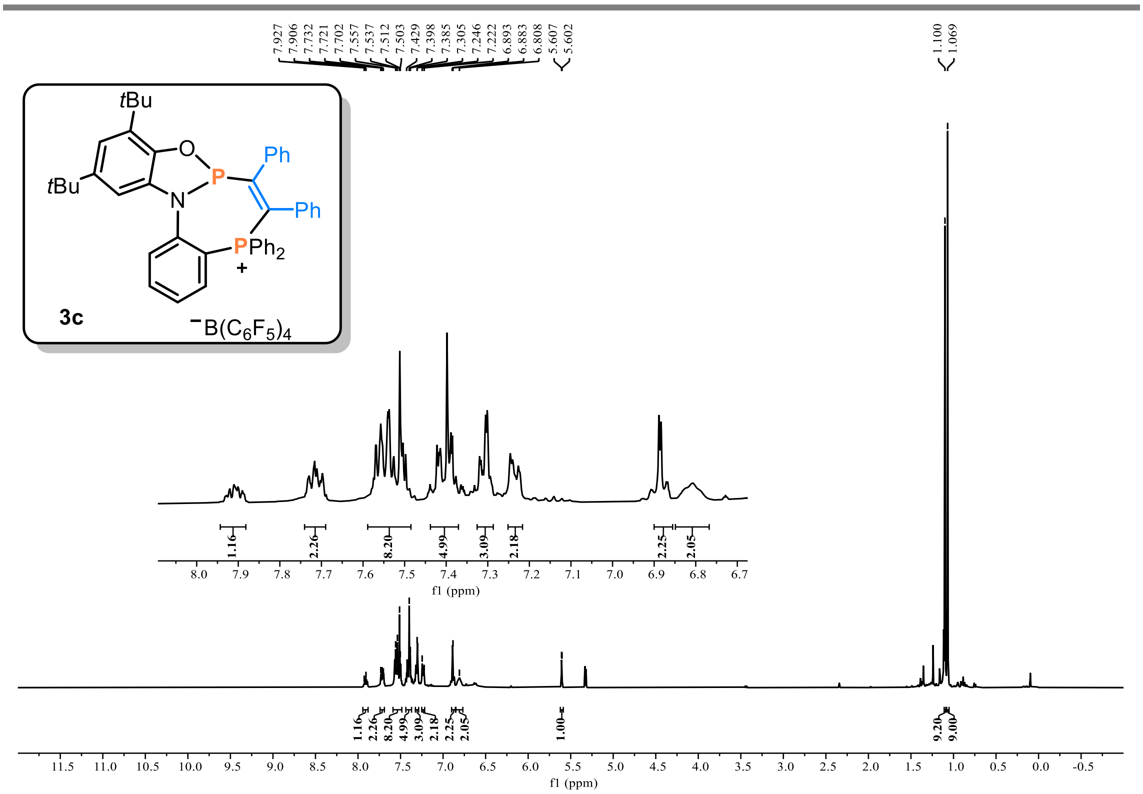

Figure S25: ^{1}**H NMR** of **3c** (400 MHz, CD_2Cl_2).

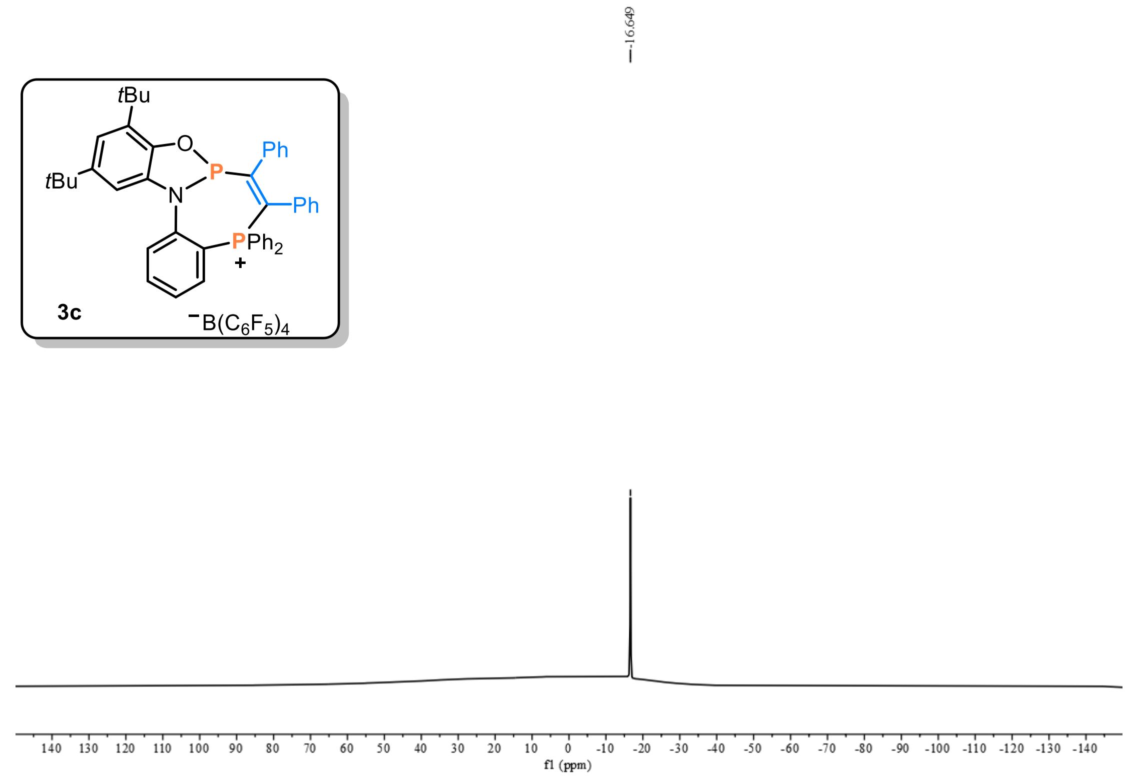

Figure S26: ^{11}**B NMR** of **3c** (128 MHz, CD_2Cl_2).

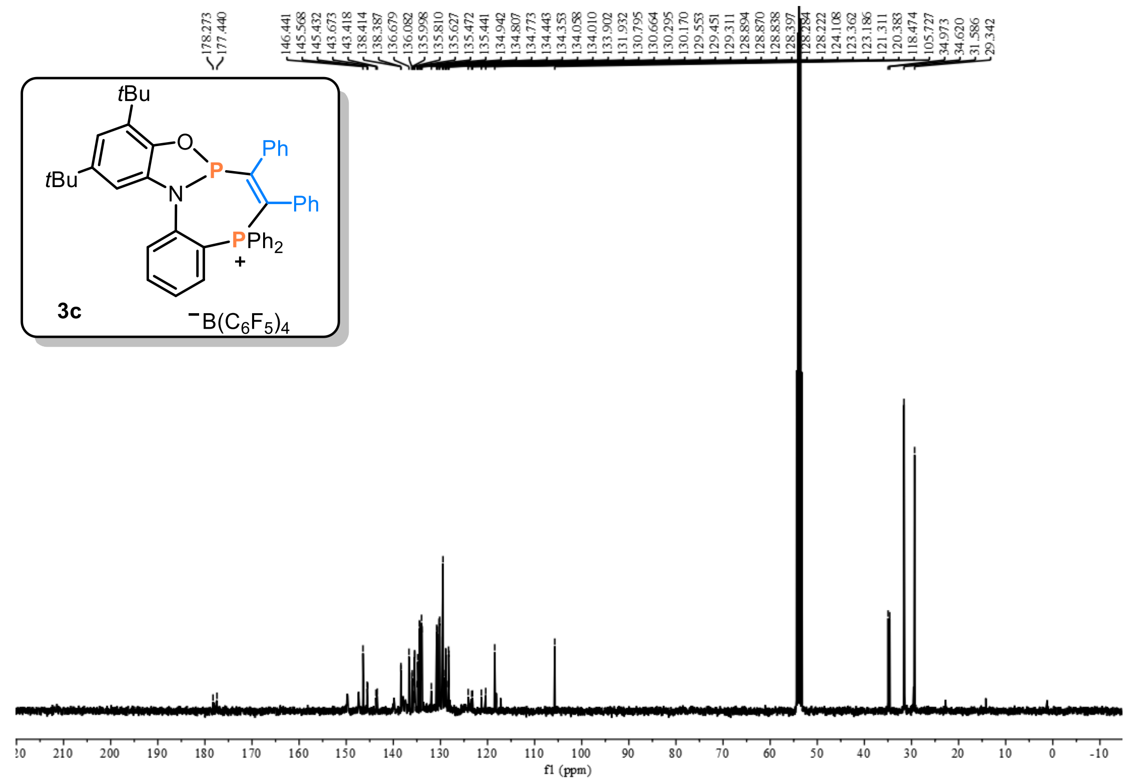

Figure S27: ^{13}**C{**^{1}**H} NMR** of **3c** (101 MHz, CD_2Cl_2).

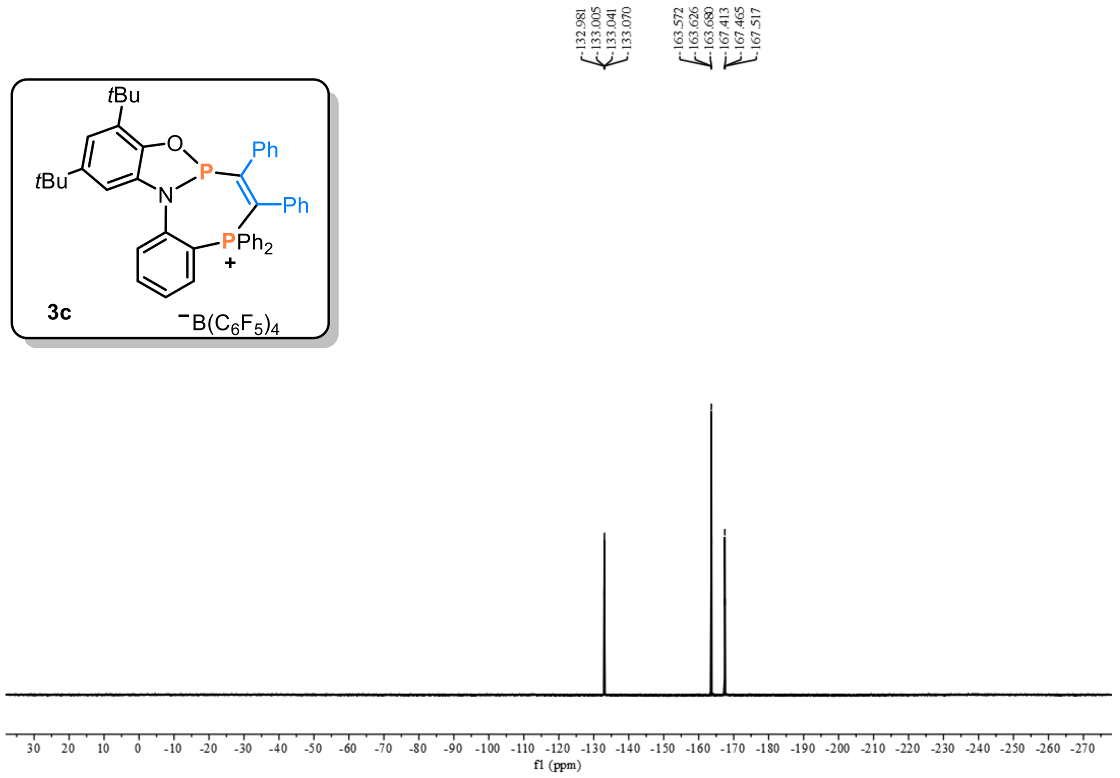

Figure S28: ^{19}**F NMR** of **3c** (376 MHz, CD_2Cl_2).

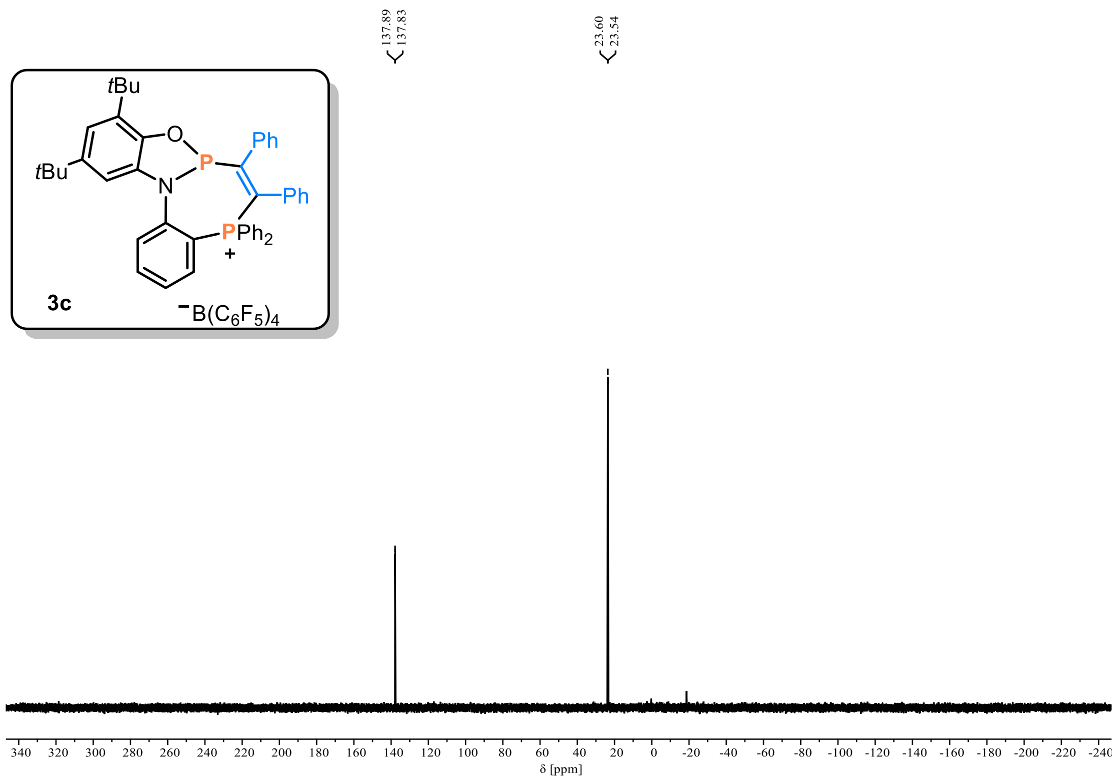

Figure S29: ^{31}**P{^{1}H} NMR** of **3c** (162 MHz, CD_2Cl_2).

4. Phosphinophosphination of Alkenes

4.1 Reaction development

4.1.1 Styrene

The yellow phosphenium salt **2a** (15 mg, 12.6 µmol, 1.0 equiv) and styrene (2.6 mg, 25.2 µmol, 2.0 equiv) were weighed into a J. Young type NMR tube, followed by the addition of CD_2Cl_2 (0.5 mL). The multinuclear NMR data revealed four doublets corresponding to a set of diastereomers in a 4:1 ratio. The major one has two doublets at 160.4 and 29.0 ppm ($^3J_{PP}$ = 14.1 Hz) while the minor one has two doublets at 153.7 and 32.4 ppm (Scheme S1). Single crystals XRD confirmed the structure of the major diastereomer.

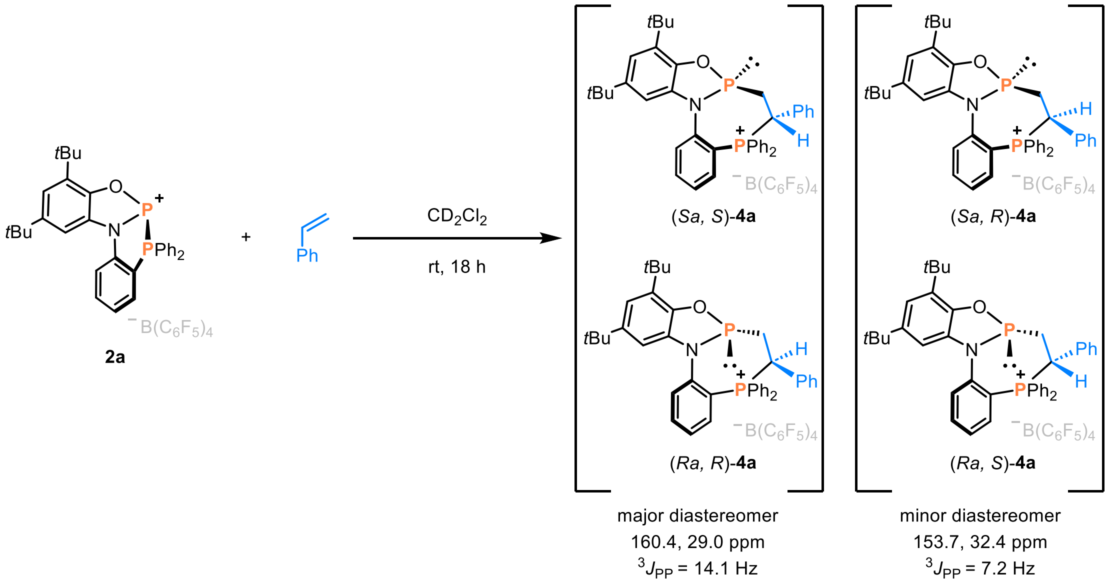

Scheme S1: 4 stereoisomers for the likely product of **2a** with styrene.

4.1.2 *cis*-Stilbene and *trans*-stilbene

Further, *cis*-stilbene and *trans*-stilbene were investigated. In such case, there should be 8 stereoisomers in total for the product (Scheme S2).

General procedure: The yellow phosphenium salt **2a** (15 mg, 12.6 µmol, 1.0 equiv) and stilbene (2.3 mg, 12.6 µmol, 1.0 equiv) were weighed into a J. Young type NMR tube. Then 0.5 mL CD_2Cl_2 was added and the tube was subjected into heating plate at 60 °C for 48 h. The solutions probed by **^{1}H** and **^{31}P NMR**.

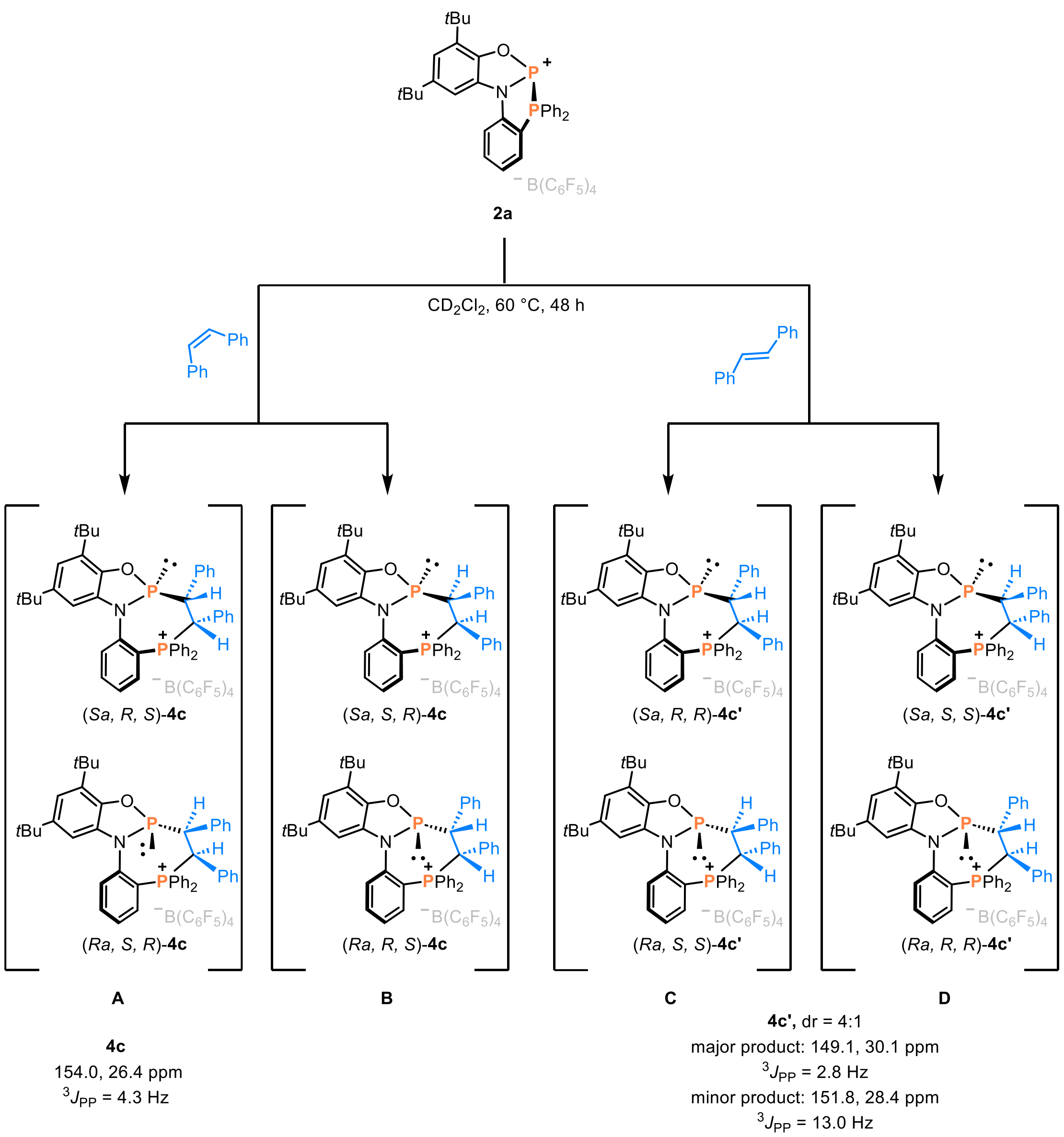

Scheme S2: 8 stereoisomers for the likely product of **2a** with stilbene.

For the reaction of **2a** with *cis*-stilbene, no starting materials remained and in **^{31}P NMR**, two doublets appeared at 154.0 and 26.4 ppm ($^3J_{PP}$ = 4.3 Hz). Single crystals XRD confirmed the structure as racemate **A**. Under the analogous conditions the recovery yield of **2a** was 33% while the yields of the two products were 53% and 14%, in an approximate ratio of 4:1 (Figure S30). In the **^{31}P NMR**, the major product has two resonances at 149.1 and 30.1 ppm ($^3J_{PP}$ = 2.8 Hz) and the minor product has two doublets at 151.8 and 28.4 ppm ($^3J_{PP}$ = 13.0 Hz), which are the mixture of racemates **C** and **D** (Scheme S2 and Figure S31).

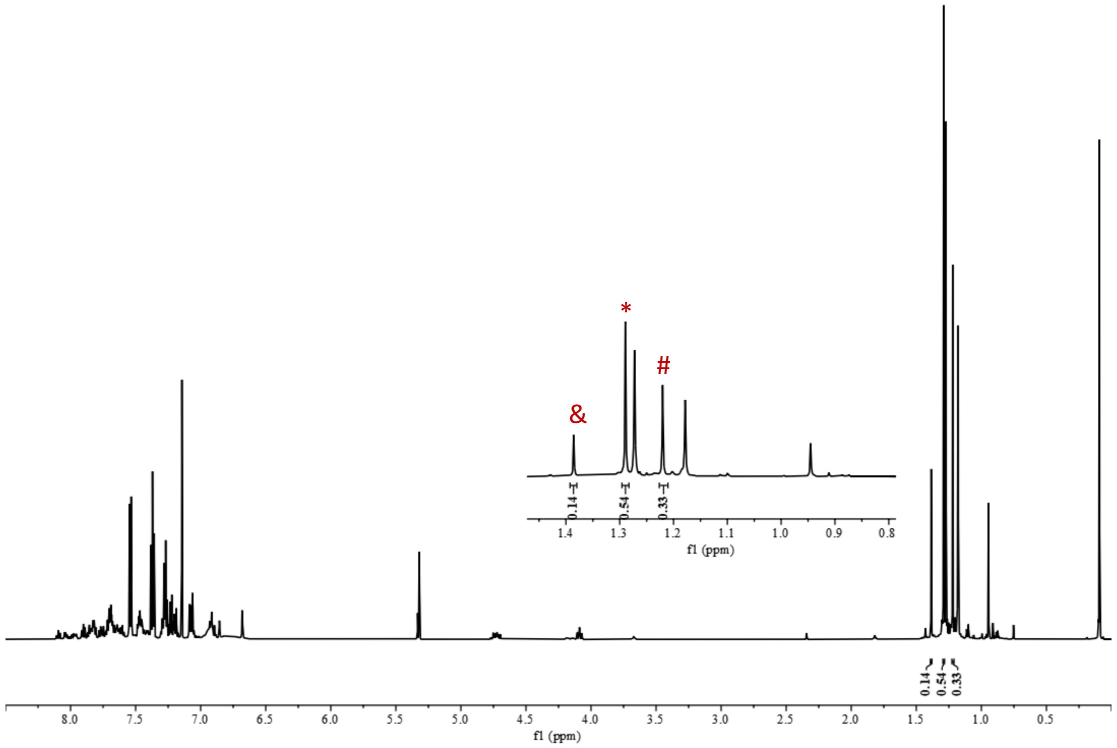

Figure S30: **^{1}H NMR** of **2a** with *trans*-stilbene. & denotes signals of minor diastereomer. * denotes signals of major diastereomer. # denotes signals of phosphenium cation **2a**.

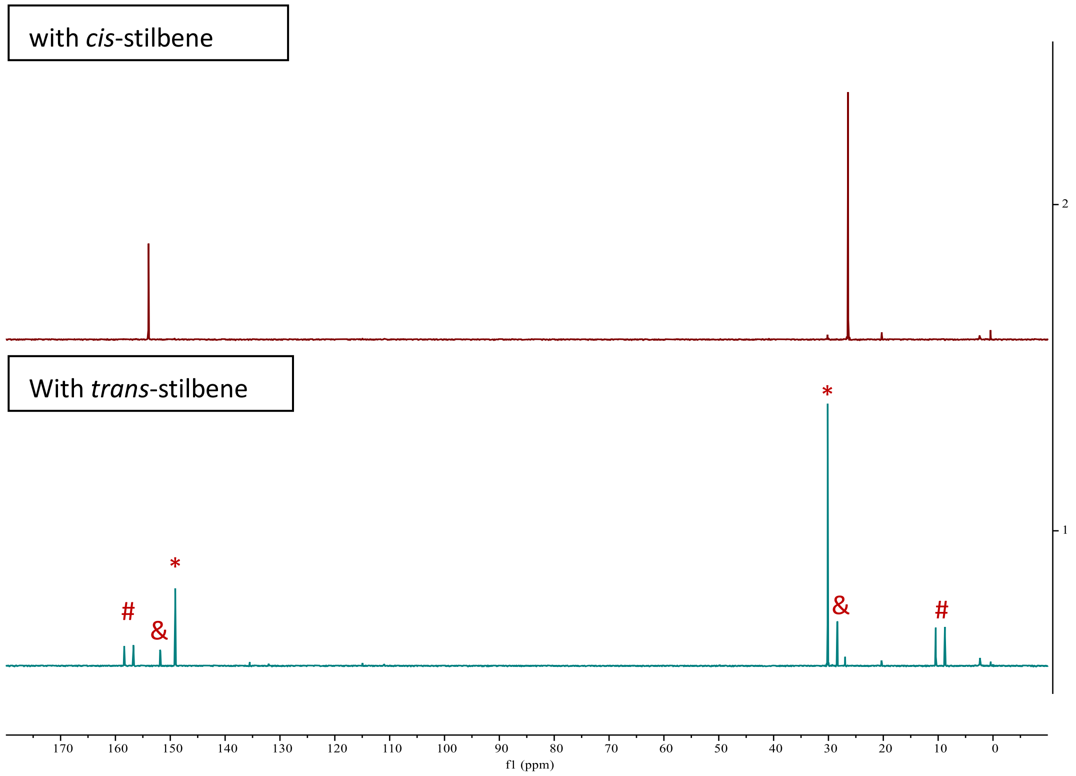

Figure S31: Stacked **^{31}P{^{1}H} NMR** of **2a** with *cis*-stilbene (top) and *trans*-stilbene (bottom). # denotes signals of phosphenium cation **2a**. & denotes signals of minor diastereomer. * denotes signals of major diastereomer.

4.2 Preparation of 4

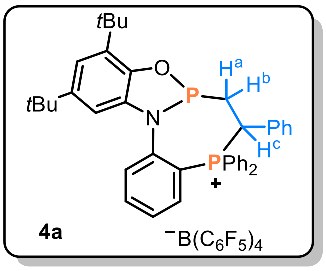

2a (100 mg, 84 µmol, 1.0 equiv) was dissolved in 2 mL CH_2Cl_2, followed by the addition of styrene (15 mg, 168 µmol, 2.0 equiv). The mixture was stirred at room temperature for 18 h. The solvent was then removed under reduced pressure and the solid was washed with pentane and dried *in vacuo* to give a set of diastereomers of product **4a** in a 4:1 ratio as a yellow solid (101.6 mg, 93% yield). Single crystals for major diastereomer suitable for X-ray diffraction were grown by gas phase diffusion of pentane into the CH_2Cl_2 solution.

^{1}H NMR (400 MHz, CD_2Cl_2) *major diastereomer*: *δ* 7.99 (ddt, *J* = 8.7, 7.5, 1.6 Hz, 1H), 7.86 (dd, *J* = 8.2, 5.1 Hz, 1H), 7.75 – 7.63 (m, 2H), 7.57 (dtdd, *J* = 14.9, 7.4, 3.5, 1.2 Hz, 3H), 7.52 – 7.45 (m, 2H), 7.40 – 7.30 (m, 6H), 7.27 (t, *J* = 10.4 Hz, 2H), 6.88 (d, *J* = 2.0 Hz, 1H), 6.75 (ddd, *J* = 11.9, 8.4, 1.3 Hz, 2H), 6.16 (d, *J* = 2.0 Hz, 1H), 4.40 (ddt, *J* = 20.1, 14.3, 5.0 Hz, 1H, PC$\boldsymbol{H}^a$H), 2.76 (ddd, *J* = 12.8, 7.1, 5.3 Hz, 1H, PCH$\boldsymbol{H}^b$), 2.58 (dtd, *J* = 37.4, 13.8, 4.7 Hz, 1H, C$\boldsymbol{H}^c$Ph), 1.34 (s, 9H), 1.14 (s, 9H). *minor diastereomer*: *δ* 4.71 – 4.54 (m, 1H), 3.03 – 2.91 (m, 1H), 2.48 – 2.29 (m, 1H), 1.40 (s, 9H), 1.28 (s, 9H). The aromatic region protons of minor diastereomer could not be reliably assigned due to low intensity and an overlap of signals.

^{11}B NMR (128 MHz, CD_2Cl_2): *δ* -16.7.

^{13}C{^{1}H} NMR (101 MHz, CD_2Cl_2) *major diastereomer* : *δ* 150.0 – 149.5 (m, $BArF_{20}$), 147.6 – 147.1 (m, $BArF_{20}$), 146.4 (s, 1C), 146.1 (d, *J* = 12.1 Hz, 1C), 145.9 (dd, *J* = 24.4, 6.6 Hz, 1C), 140.0 – 139.6 (m, $BArF_{20}$), 138.4 (d, *J* = 2.7 Hz, 1C), 138.2 (d, *J* = 8.2 Hz, 1C), 138.1 – 137.7 (m, $BArF_{20}$), 137.6 – 137.2 (m, $BArF_{20}$), 136.9 (s, 1C), 135.6 (dd, *J* = 9.5, 3.0 Hz, 1C), 135.5 – 135.2 (m, $BArF_{20}$), 134.4 (d, *J* = 9.1 Hz, 2C), 133.8 (d, *J* = 2.3 Hz, 2C), 132.8 (d, *J* = 9.2 Hz, 2C), 131.5 (dd, *J* = 11.3, 6.7 Hz, 1C), 131.2 (d, *J* = 12.5 Hz, 1C), 130.4 (d, *J* = 2.8 Hz, 1C), 130.2 (s, 1C), 130.1 (d, *J* = 11.1 Hz, 2C), 129.5 (d, *J* = 2.9 Hz, 2C), 129.4 (dd, *J* = 2.1, 7.9 Hz, 1C), 128.7 (d, *J* = 12.0 Hz, 1C), 120.0 (d, *J* = 3.6 Hz, 1C), 119.2 (d, *J* = 3.5 Hz, 1C), 118.9 (s, 1C), 118.4 (s, 1C), 118.1 (s, 1C), 117.6 (s, 1C), 106.8 (s, 1C), 39.6 (dd, *J* = 44.8, 5.3 Hz, 1C), 39.4 (d, *J* = 45.8 Hz, 1C), 35.0 (s, 1C), 34.8 (s, 1C), 31.7 (s, 3C), 29.5 (s, 3C). The minor

diastereomer signals could not be reliably assigned due to low intensity and an overlap of signals.

^{19}F NMR (376 MHz, CD_2Cl_2): δ -133.0 – -133.2 (m), -163.7 (t, J = 20.2 Hz), -167.5 (t, J = 19.1 Hz).

^{31}P{^{1}H} NMR (162 MHz, CD_2Cl_2): δ 160.4 (d, J = 14.1 Hz, major diastereomer, O***P***N), 153.7 (d, J = 7.2 Hz, minor diastereomer, O***P***N), 32.4 (d, J = 7.2 Hz, minor diastereomer, ***P***Ph$_2$), 29.0 (d, J = 14.1 Hz, major diastereomer, ***P***Ph$_2$).

HRMS (ESI): $[C_{40}H_{42}NOP_2]^+$, calculated: 614.2736, found: 614.2738.

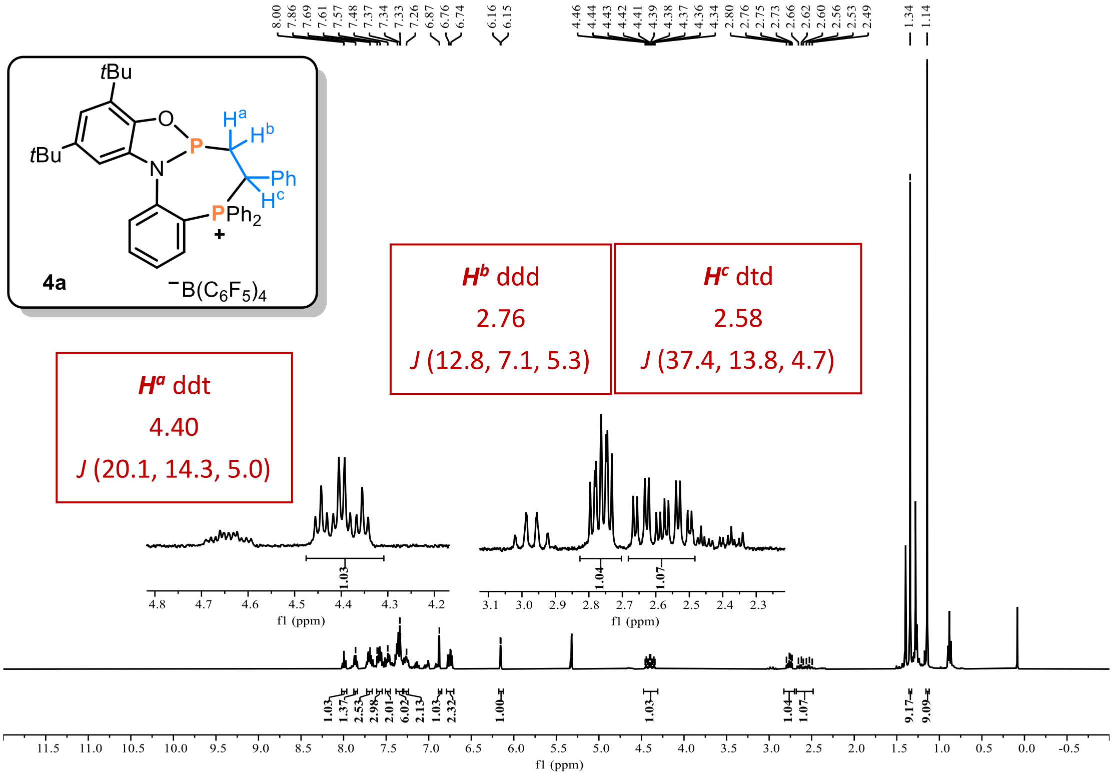

Figure S32: **^{1}H NMR** of **4a** (400 MHz, CD_2Cl_2), Only peaks for major diastereomer are labeled and integrated.

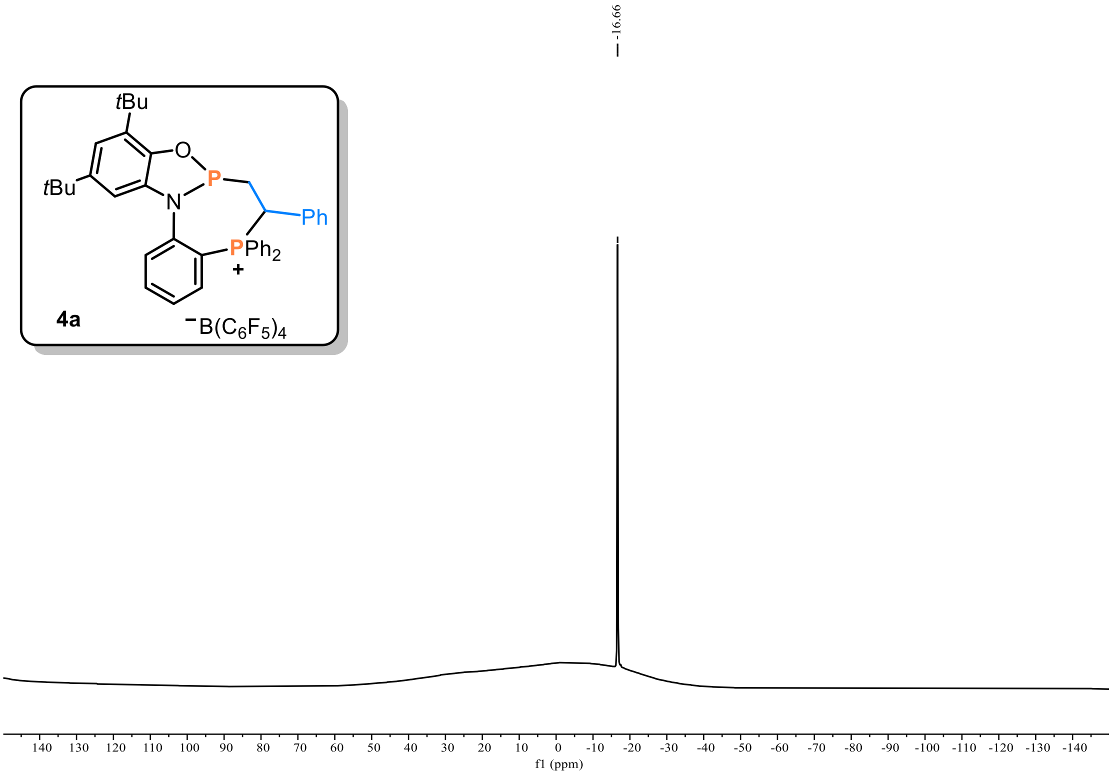

Figure S33: **^{11}B NMR** of **4a** (128 MHz, CD_2Cl_2).

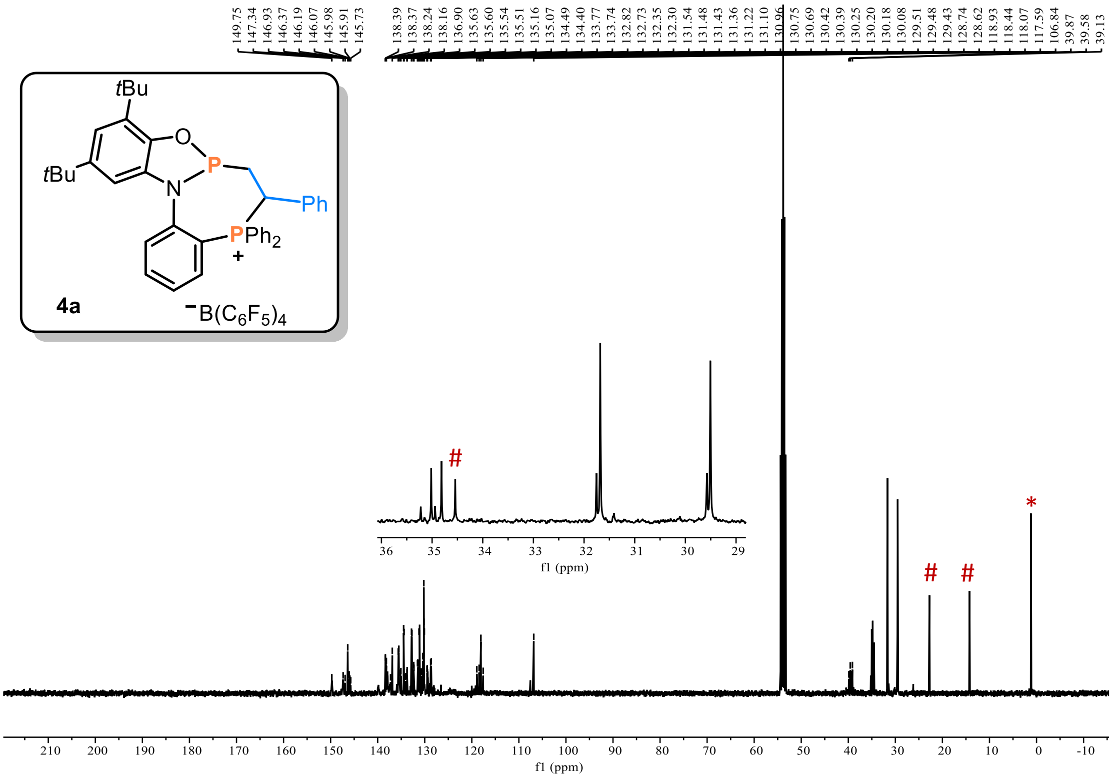

Figure S34: **^{13}C{^{1}H} NMR** of **4a** (101 MHz, CD_2Cl_2), # denotes pentane and * denotes silicone grease.

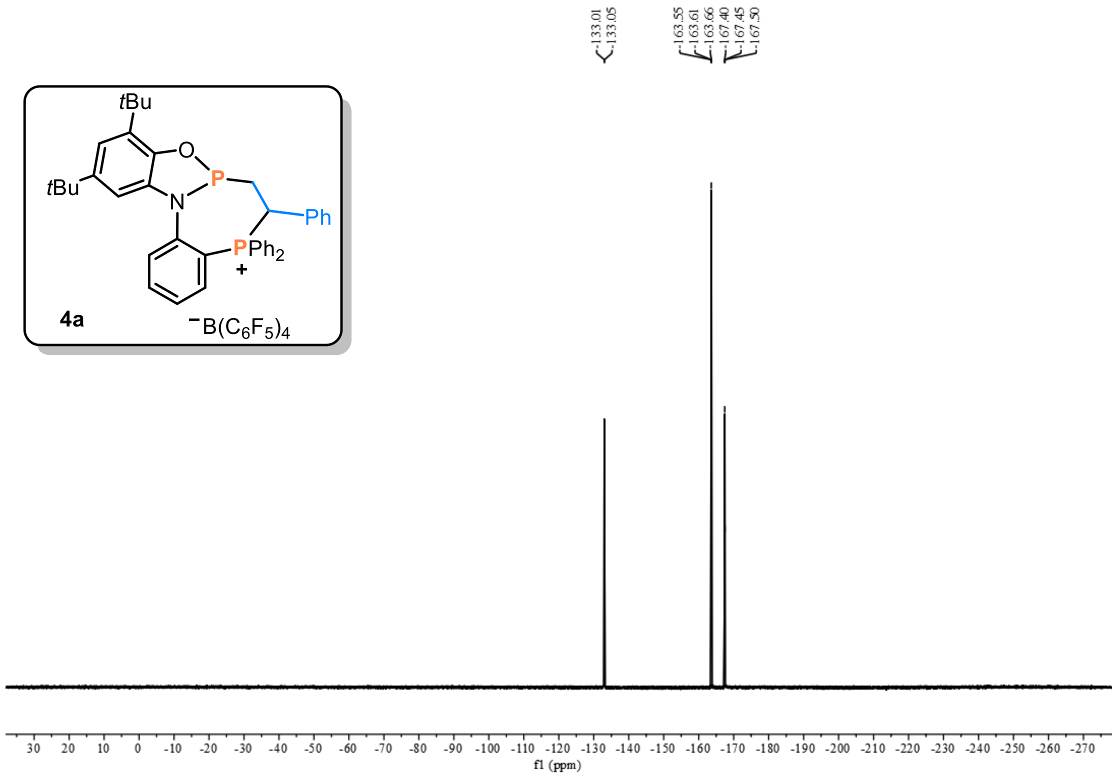

Figure S35: ^{19}F NMR of **4a** (376 MHz, CD_2Cl_2).

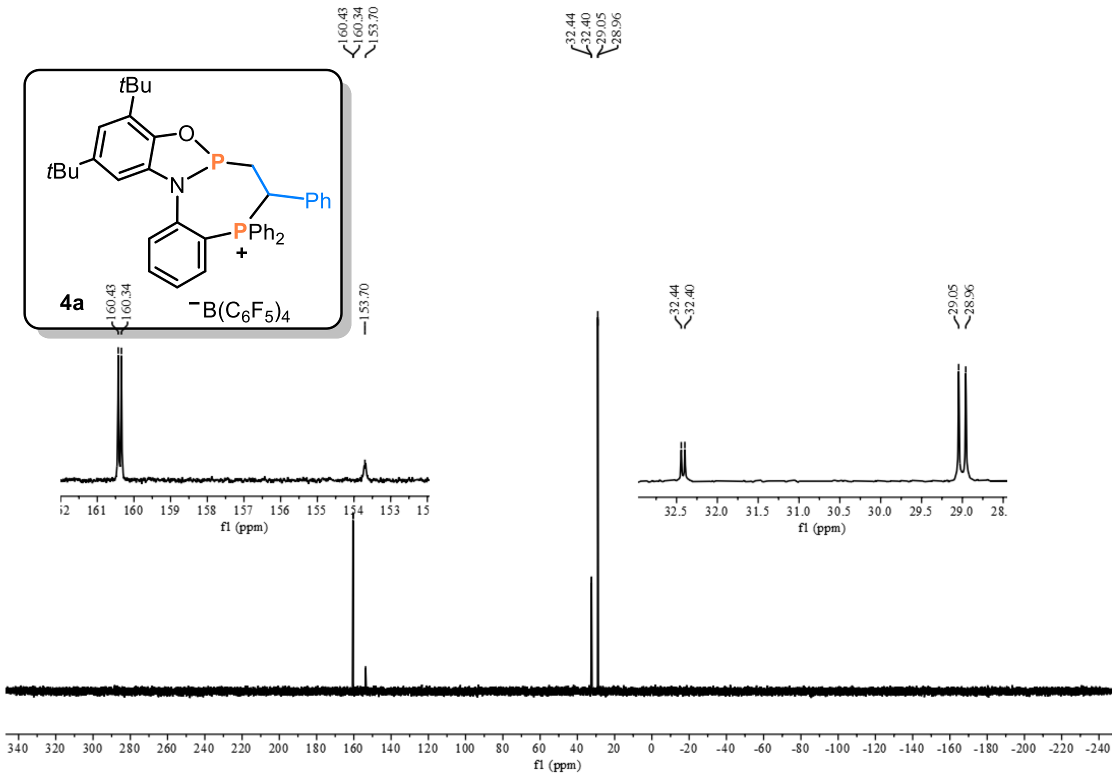

Figure S36: ^{31}P{^{1}H} NMR of **4a** (162 MHz, CD_2Cl_2).

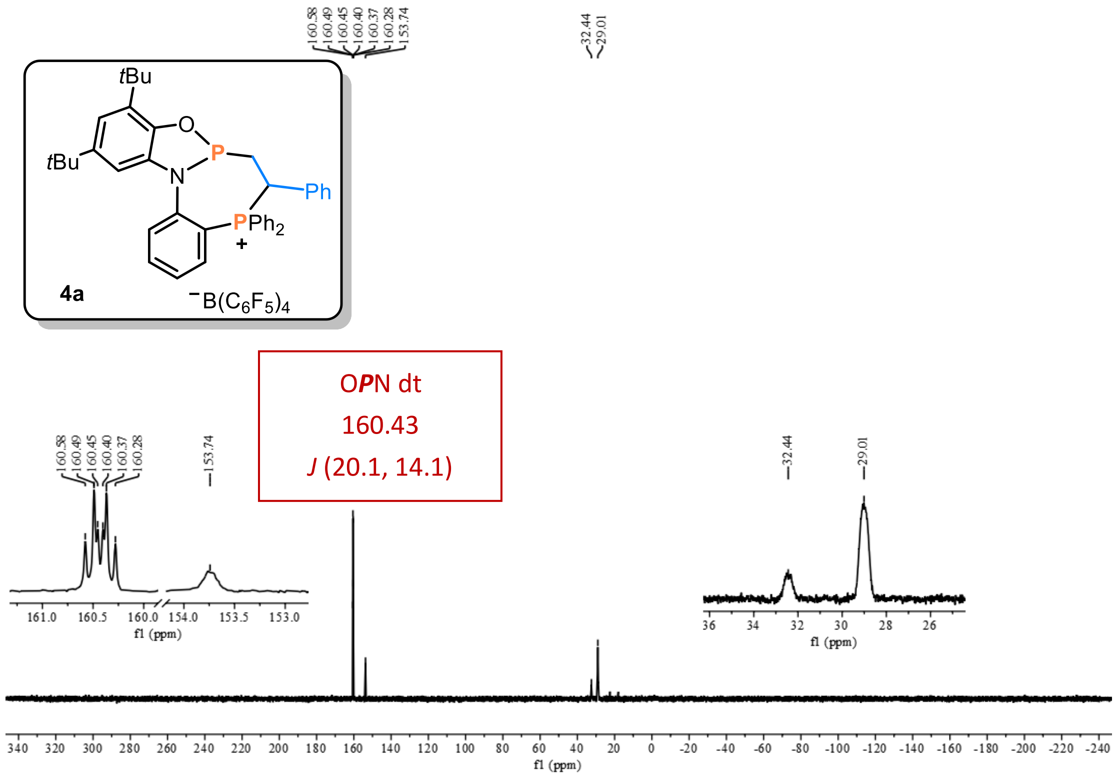

Figure S37: ^{31}**P NMR** of **4a** (162 MHz, CD_2Cl_2).

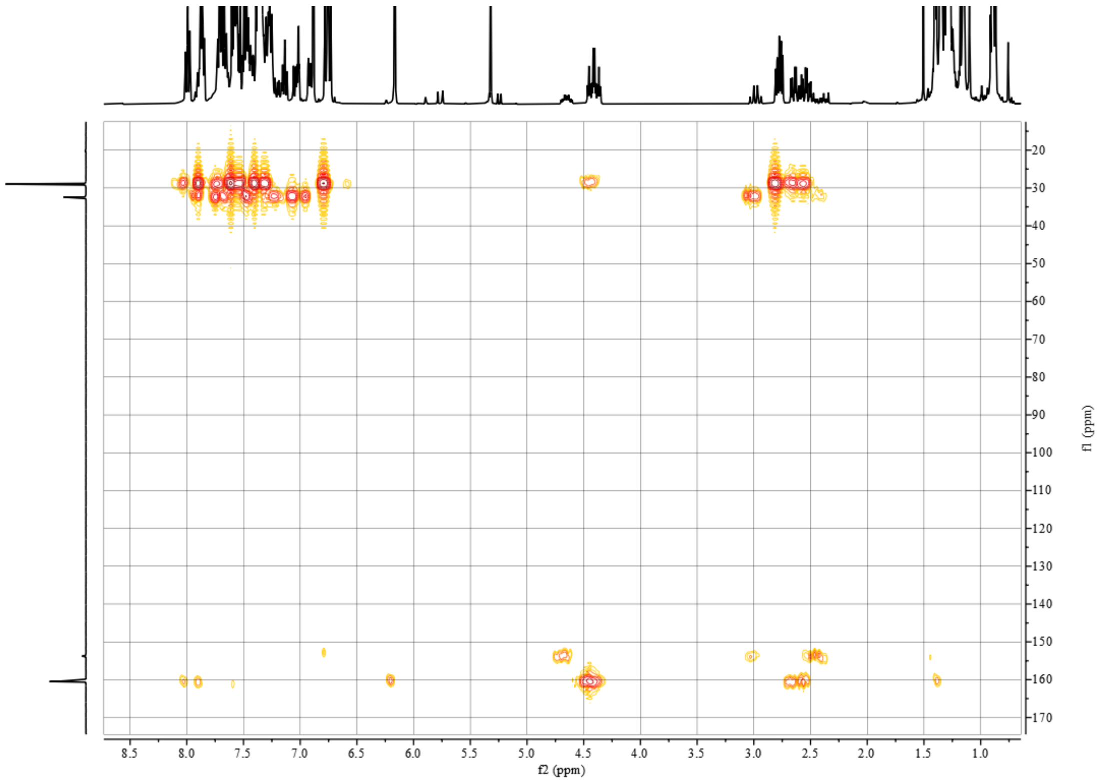

Figure S38: ^{1}**H-**^{31}**P HMBC NMR** of **4a** (162 MHz, CD_2Cl_2).

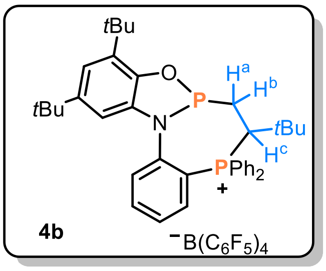

2a (200 mg, 168 µmol, 1.0 equiv) was dissolved in 4 mL CH_2Cl_2, followed by the addition of 3,3-dimethyl-1-butene (42.4 mg, 504 µmol, 3.0 equiv). The mixture was stirred at room temperature for 48 h. The solvent was then removed under reduced pressure and the solid was washed with pentane and dried *in vacuo* to give the product **4b** as a yellow solid (180.8 mg, 84% yield). Single crystals suitable for X-ray diffraction were grown by gas phase diffusion of pentane into the CH_2Cl_2 solution.

^{1}H NMR (400 MHz, CD_2Cl_2): δ 7.96 (ddq, J = 8.9, 7.2, 1.4 Hz, 1H), 7.90 – 7.77 (m, 5H), 7.72 (dd, J = 7.8, 5.2 Hz, 1H), 7.66 (tdd, J = 7.4, 3.4, 1.6 Hz, 1H), 7.56 – 7.49 (m, 2H), 7.47 (ddd, J = 9.3, 5.1, 2.1 Hz, 3H), 7.43 – 7.39 (m, 1H), 6.87 (d, J = 2.0 Hz, 1H), 6.01 (d, J = 2.0 Hz, 1H), 3.81 – 3.55 (m, 1H, PC***H****^{a}*H), 2.92 – 2.43 (m, 2H, ***H****^{b}*&***H****^{c}*), 1.46 (s, 9H), 1.14 (s, 9H), 0.80 (s, 9H).

^{11}B NMR (128 MHz, CD_2Cl_2): δ -16.7.

^{13}C{^{1}H} NMR (151 MHz, CD_2Cl_2): δ 149.5 – 149.2 (m, $BArF_{20}$), 147.9 – 147.6 (m, $BArF_{20}$), 146.8 (d, J = 10.4 Hz, 1C), 146.4 (s, 1C), 139.6 – 139.3 (m, $BArF_{20}$), 138.0 – 137.7 (m, $BArF_{20}$), 137.6 – 137.4 (m, $BArF_{20}$), 137.5 (d, J = 2.5 Hz, 2C), 136.6 (d, J = 3.1 Hz, 1C), 136.3 (s, 1C), 136.0 – 135.9 (m, $BArF_{20}$), 135.9 (d, J = 9.7 Hz, 1C), 135.8 (d, J = 9.4 Hz, 2C), 135.1 (d, J = 3.1 Hz, 2C), 132.8 (d, J = 9.2 Hz, 2C), 132.3 (dd, J = 7.4, 1.5 Hz, 1C), 131.1 (d, J = 12.1 Hz, 2C), 130.7 (d, J = 12.3 Hz, 2C), 130.2 (d, J = 11.8 Hz, 1C), 121.1 (s, 1C), 120.6 (s, 1C), 116.3 (s, 1C), 104.2 (s, 1C), 37.1 (s, 1C), 37.1 (d, J = 2.2 Hz, 1C), 36.8 (s, 1C), 35.1 (s, 1C), 34.9 (s, 1C), 31.7 (s, 3C), 29.8 (s, 3C), 29.7 (d, J = 6.3 Hz, 3C).

^{19}F NMR (565 MHz, CD_2Cl_2): δ -130.3 – -136.2 (m), -163.6 (t, J = 20.4 Hz), -167.5 (t, J = 19.2 Hz).

^{31}P{^{1}H} NMR (162 MHz, CD_2Cl_2): δ 190.5 (br, O***P***N), 31.4 (s, ***P***Ph_2).

HRMS (ESI): $[C_{38}H_{46}NOP_2]^+$, calculated: 594.3049, found: 594.3053.

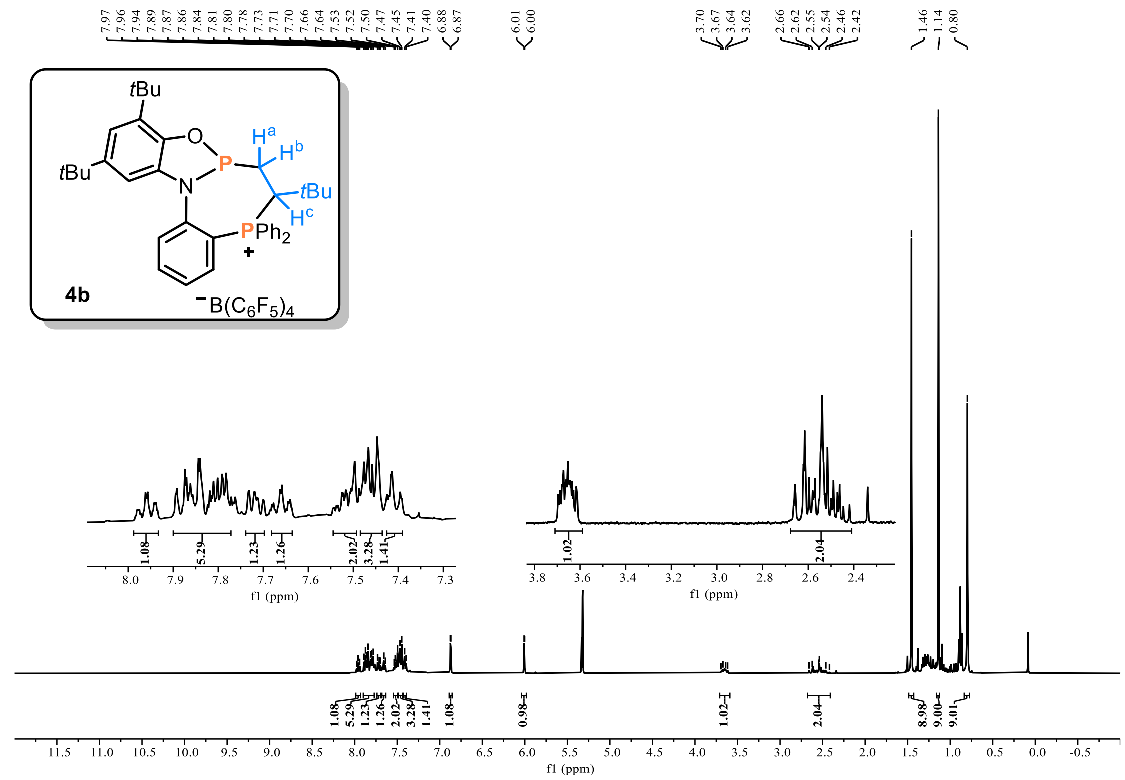

Figure S39: ^{1}H NMR of **4b** (400 MHz, CD_2Cl_2).

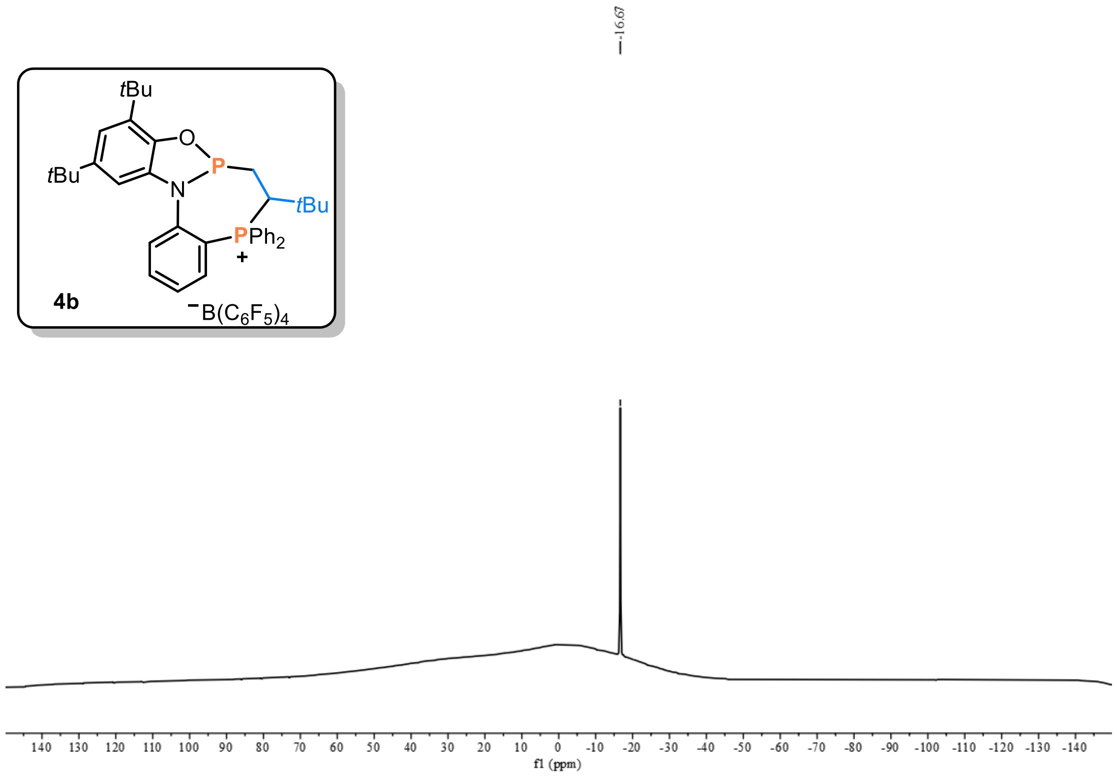

Figure S40: ^{11}B NMR of **4b** (128 MHz, CD_2Cl_2).

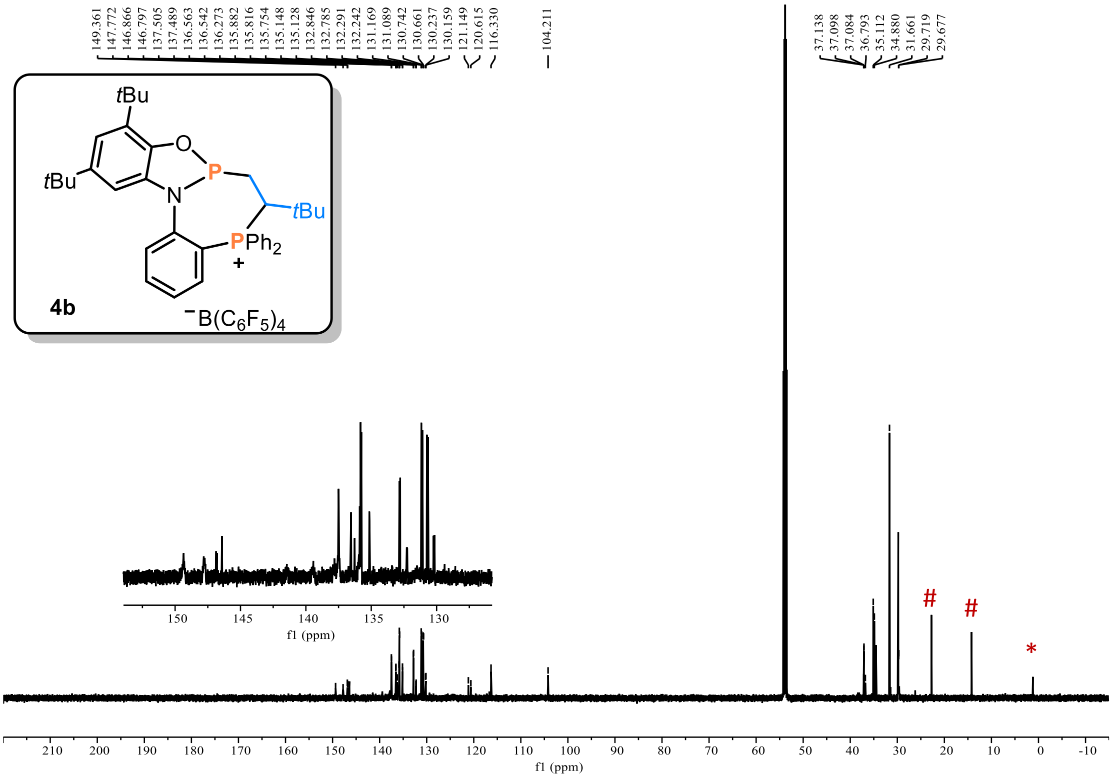

Figure S41: **$^{13}C\{^1H\}$ NMR** of **4b** (151 MHz, CD_2Cl_2). # denotes pentane and * denotes silicone grease.

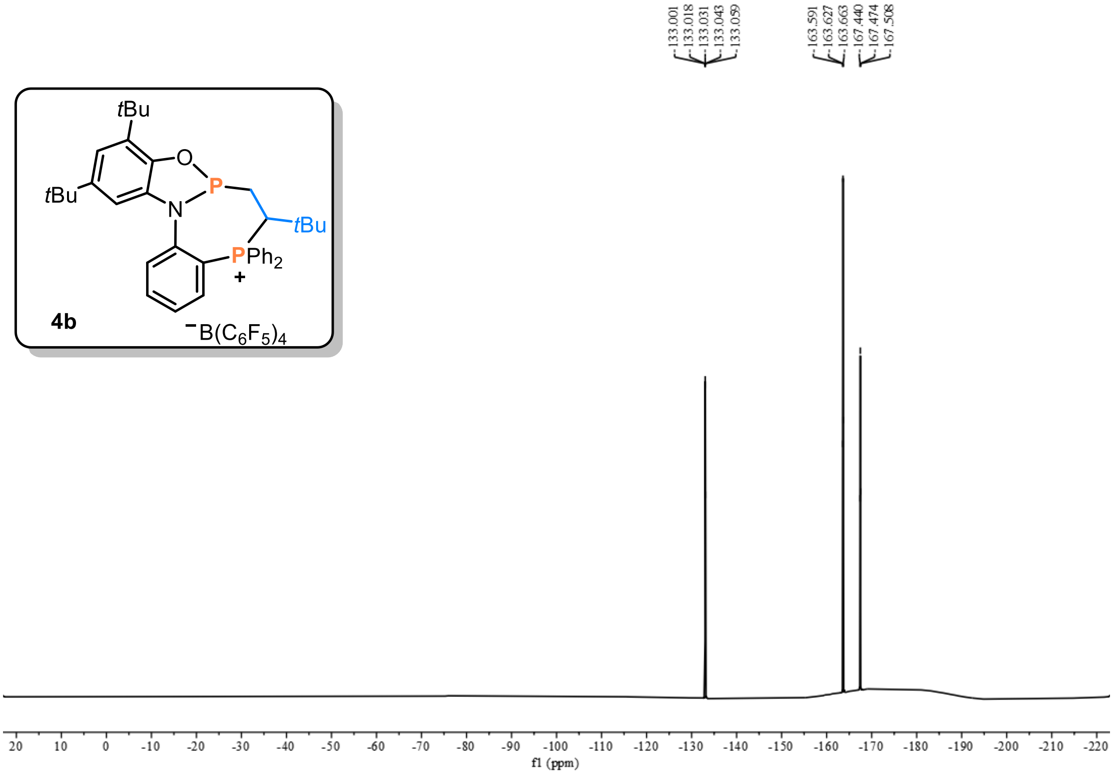

Figure S42: **^{19}F NMR** of **4b** (565 MHz, CD_2Cl_2).

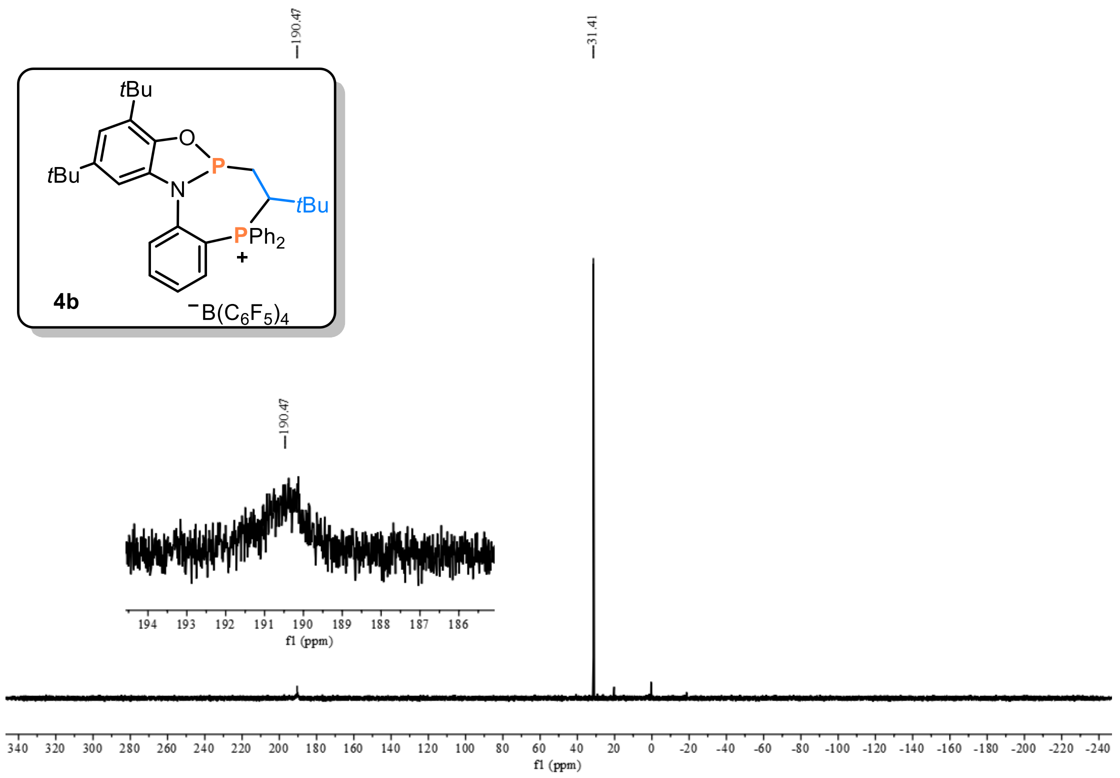

Figure S43: ^{31}**P{**^{1}**H} NMR** of **4b** (162 MHz, CD_2Cl_2).

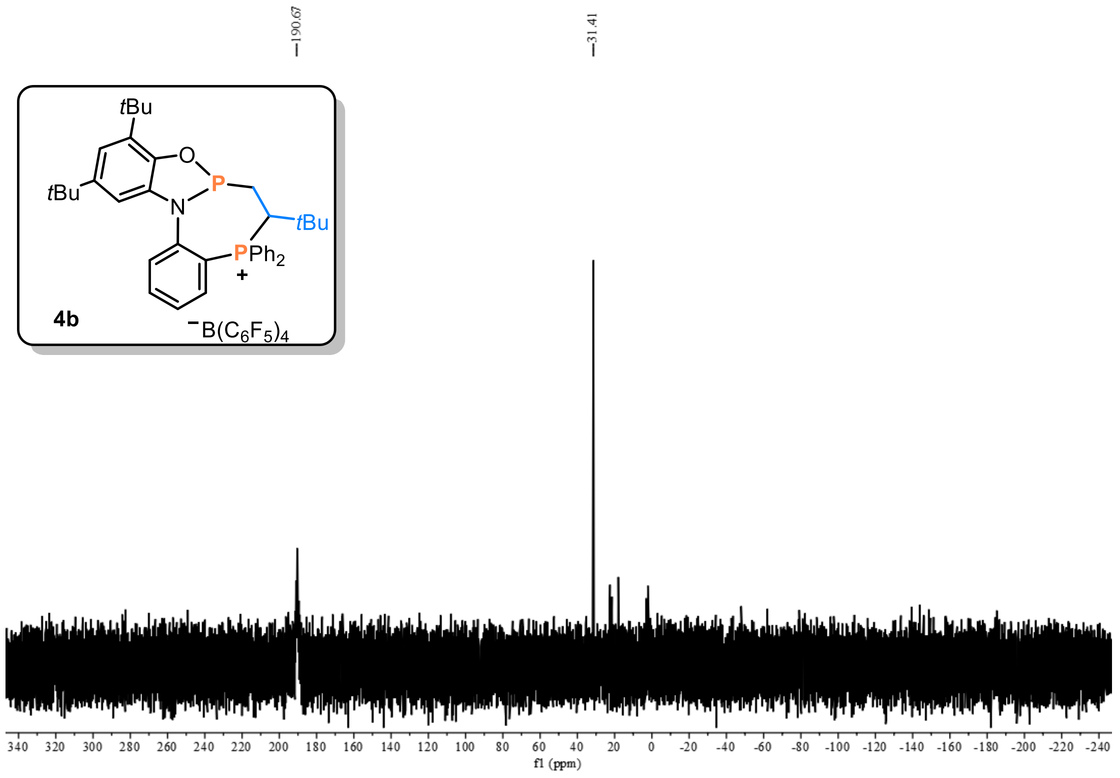

Figure S44: ^{31}**P NMR** of **4b** (162 MHz, CD_2Cl_2).

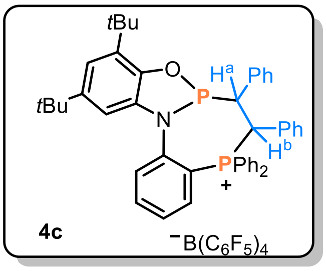

2a (300 mg, 252 μmol, 1.0 equiv) was dissolved in 5 mL CH_2Cl_2, followed by the addition of *cis*-stilbene (45.5 mg, 252 μmol, 1.0 equiv). The mixture was stirred at 60 °C for 48 h. The solvent was then removed under reduced pressure and the solid was washed with pentane and dried *in vacuo* to give product **4c** as a yellow solid (301 mg, 87% yield). Single crystals suitable for X-ray diffraction were grown by gas phase diffusion of pentane into the solution of toluene and CH_2Cl_2 (1:1).

^{1}H NMR (400 MHz, CD_2Cl_2): δ 8.13 – 8.02 (m, 1H), 7.98 – 7.86 (m, 1H), 7.82 – 7.74 (m, 1H), 7.72 – 7.49 (m, 8H), 7.47 – 7.35 (m, 3H), 7.30 – 7.23 (m, 3H), 7.23 – 7.11 (m, 3H), 6.88 – 6.75 (m, 3H), 6.54 (ddd, J = 11.8, 8.6, 1.3 Hz, 2H), 6.11 (d, J = 2.0 Hz, 1H), 4.36 (ddd, J = 19.8, 16.0, 4.2 Hz, 1H, PC$\boldsymbol{H}^a$Ph), 3.90 (t, J = 4.2 Hz, 1H, PPh_2C$\boldsymbol{H}^b$Ph), 1.13 (s, 9H), 1.08 (s, 9H).

^{11}B NMR (128 MHz, CD_2Cl_2): δ -16.7.

^{13}C{^{1}H} NMR (101 MHz, CD_2Cl_2): δ 150.0 – 149.5 (m, $BArF_{20}$), 147.5 – 147.2 (m, $BArF_{20}$), 146.2 (s, 1C), 145.5 (dd, J = 16.5, 6.0 Hz, 1C), 144.9 (d, J = 12.4 Hz, 1C), 140.0 – 139.6 (m, $BArF_{20}$), 138.8 (d, J = 2.6 Hz, 1C), 138.5 (d, J = 8.3 Hz, 1C), 138.1 – 137.7 (m, $BArF_{20}$), 137.6 – 137.1 (m, $BArF_{20}$), 136.8 (s, 1C), 135.6 (dd, J = 43.3, 3.1 Hz, 1C), 135.6 – 135.2 (m, $BArF_{20}$), 134.3 (d, J = 2.6 Hz, 1C), 134.2 (d, J = 8.9 Hz, 2C), 133.5 (dd, J = 16.2, 9.3 Hz, 1C), 132.7 (d, J = 9.0 Hz, 2C), 131.5 (d, J = 12.3 Hz, 2C), 130.8 (d, J = 2.5 Hz, 1C), 130.7 (dd, J = 8.0, 1.8 Hz, 1C), 130.2 (d, J = 7.2 Hz, 2C), 130.0 (d, J = 12.2 Hz, 2C), 129.4 (s, 2C), 129.3 (dd, J = 11.3, 3.4 Hz, 1C), 129.2 (d, J = 11.9 Hz, 1C), 128.8 (s, 2C), 120.6 (d, J = 2.8 Hz, 1C), 119.8 (d, J = 2.8 Hz, 1C), 118.6 (s, 1C), 118.1 (d, J = 5.5 Hz, 1C), 118.1 (s, 2C), 117.8 (s, 1C), 117.3 (s, 1C), 106.6 (s, 1C), 47.0 (d, J = 14.5 Hz, 1C), 46.6 (d, J = 14.5 Hz, 1C), 35.0 (s, 1C), 34.6 (s, 1C), 31.7 (s, 3C), 29.4 (s, 3C).

^{19}F NMR (376 MHz, CD_2Cl_2): δ -129.3 – -136.9 (m), -163.6 (t, J = 20.4 Hz), -167.4 (t, J = 19.4 Hz).

^{31}P{^{1}H} NMR (162 MHz, CD_2Cl_2): δ 154.0 (d, J = 4.4 Hz, O$\boldsymbol{P}$N), 26.4 (d, J = 4.3 Hz, $\boldsymbol{P}Ph_2$).

HRMS (ESI): $[C_{46}H_{46}ClNOP_2]^+$, calculated: 690.3049, found: 690.3054.

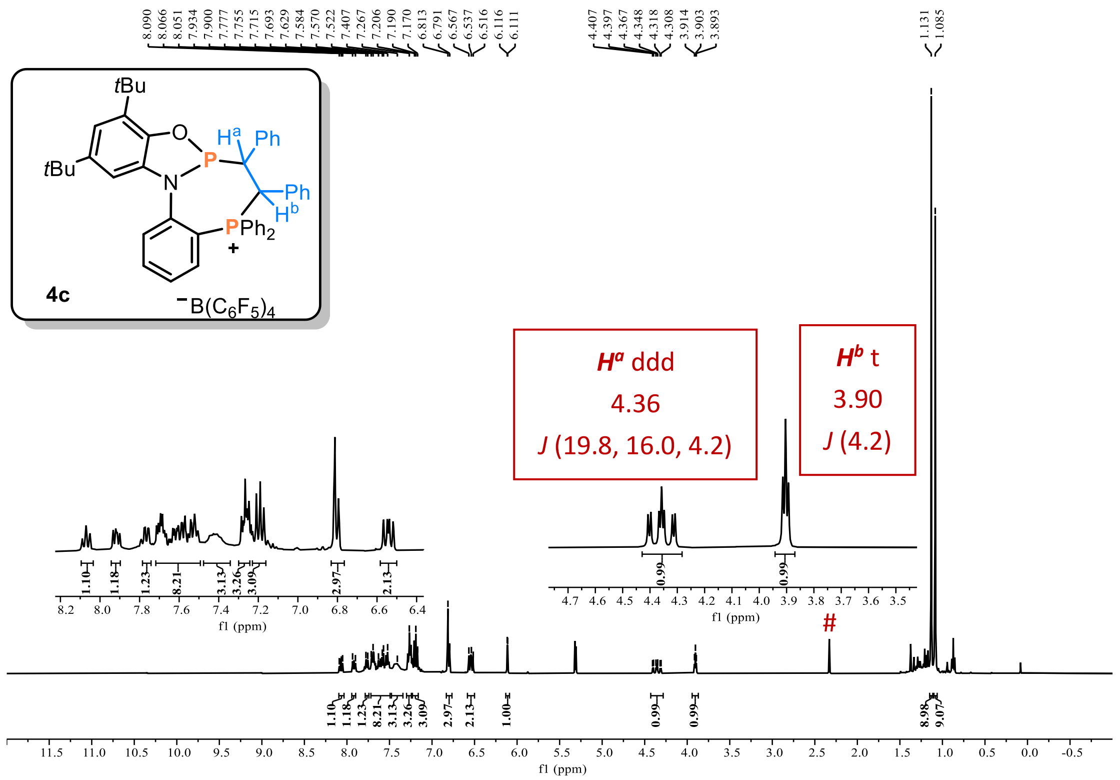

Figure S45: ^{1}**H NMR** of **4c** (400 MHz, CD_2Cl_2), # denotes toluene.

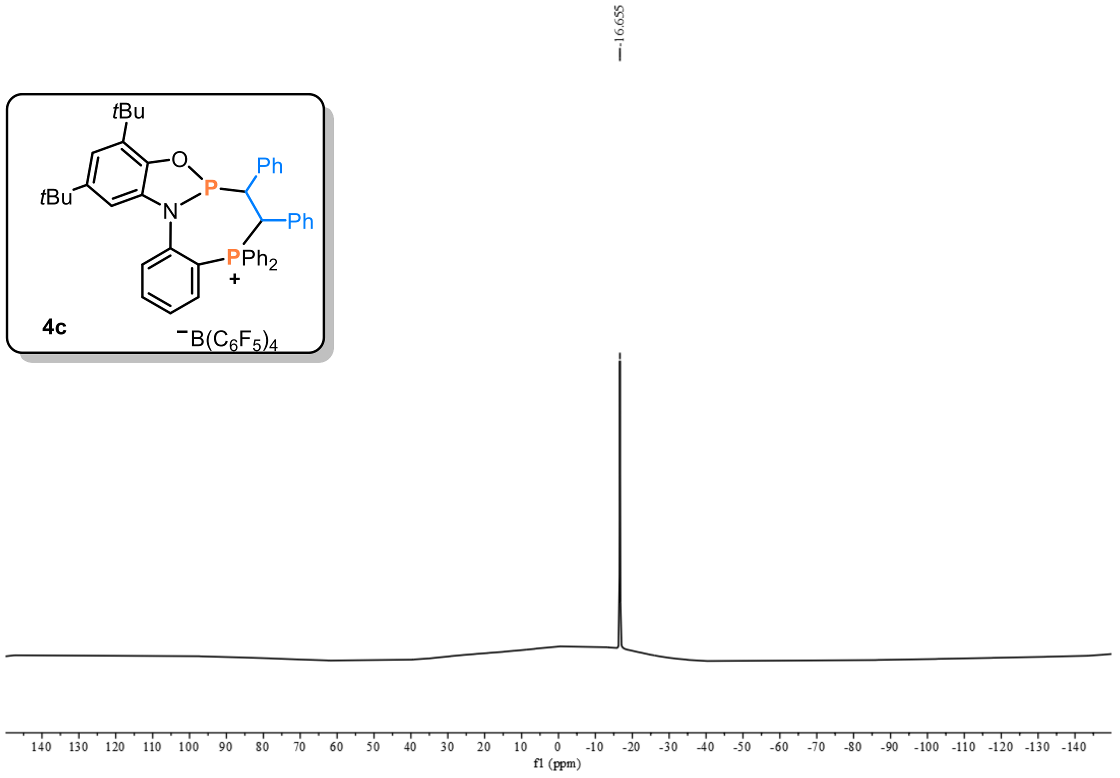

Figure S46: ^{11}**B NMR** of **4c** (128 MHz, CD_2Cl_2).

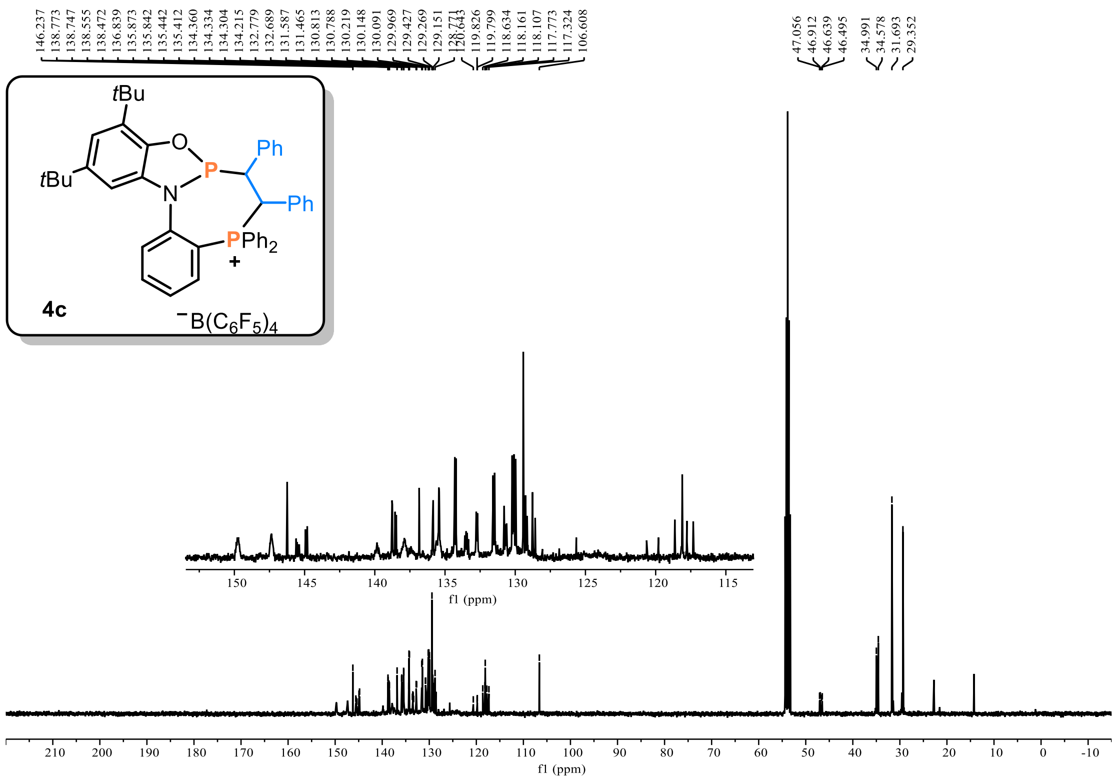

Figure S47: **^{13}C{^{1}H} NMR** of **4c** (101 MHz, CD_2Cl_2).

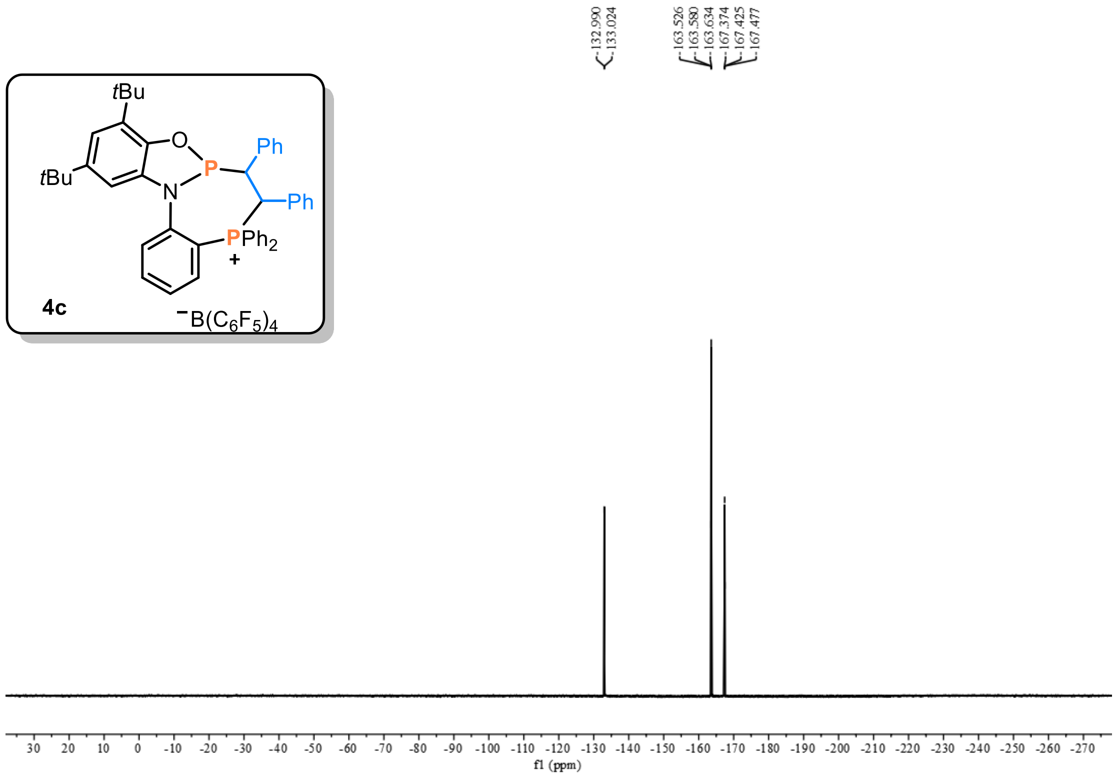

Figure S48: **^{19}F NMR** of **4c** (376 MHz, CD_2Cl_2).

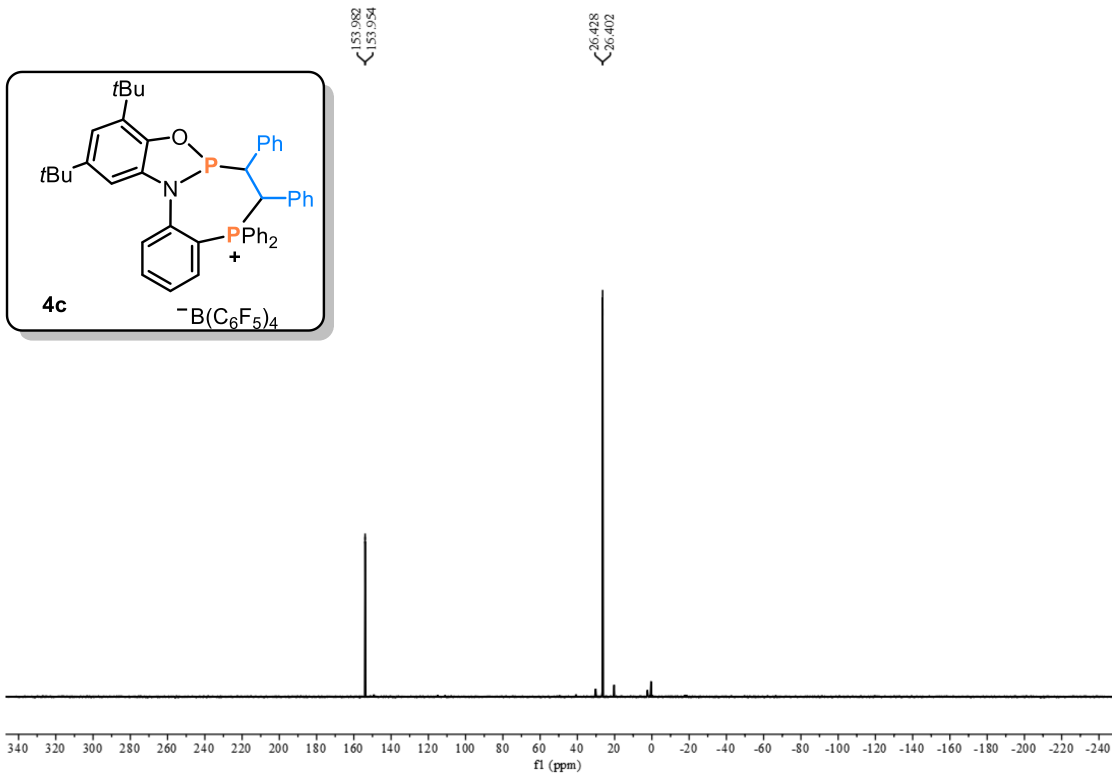

Figure S49: ^{31}**P{**^{1}**H} NMR** of **4c** (162 MHz, CD_2Cl_2).

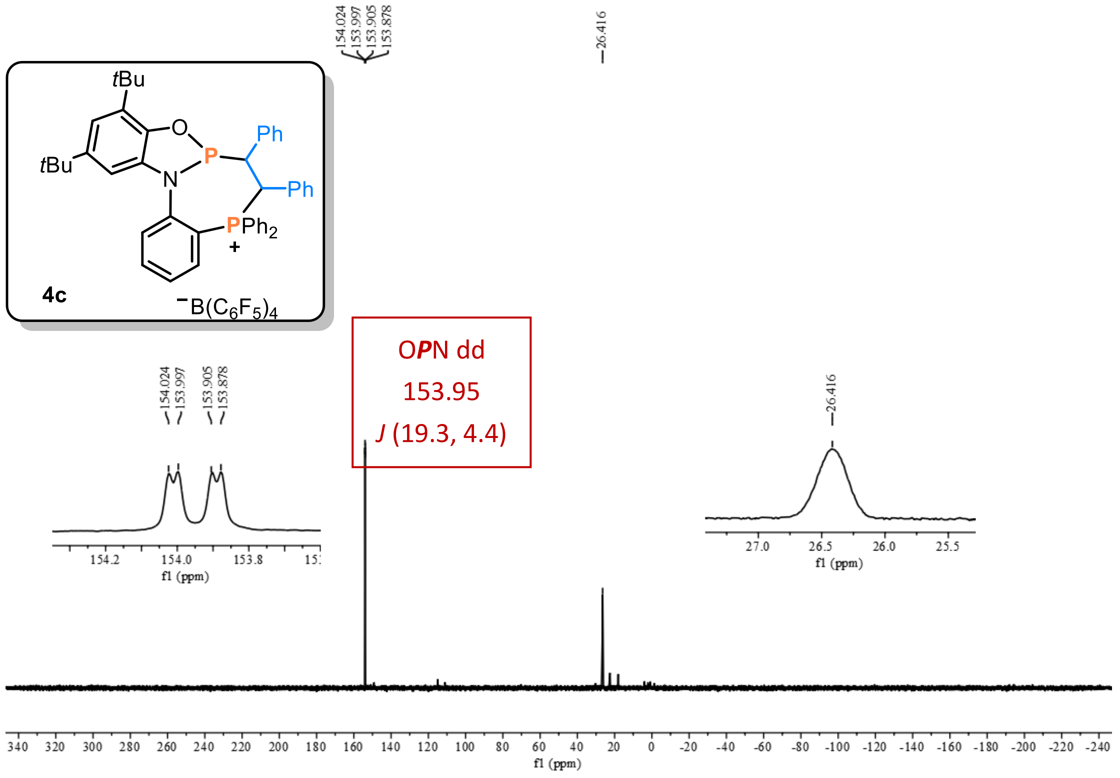

Figure S50: ^{31}**P NMR** of **4c** (162 MHz, CD_2Cl_2).

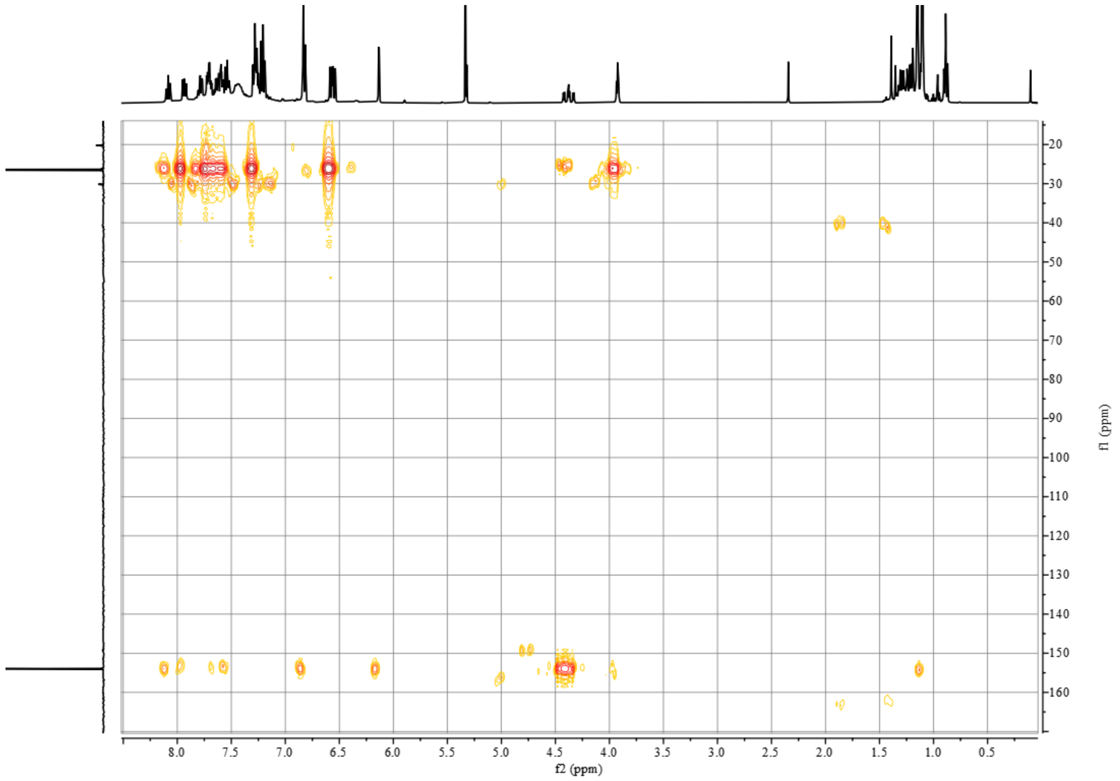

Figure S51: ^{1}H-^{31}P HMBC NMR of **4c** (162 MHz, CD_2Cl_2).

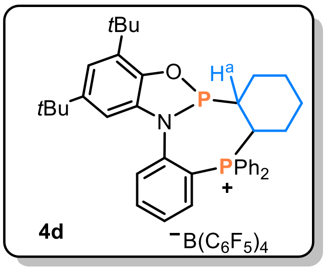

2a (100 mg, 84 μmol, 1.0 equiv) was dissolved in 2 mL CH_2Cl_2, followed by the addition of cyclohexene (8.3 mg, 101 μmol, 1.2 equiv). The mixture was stirred at room temperature for 18 h. The solvent was then removed under reduced pressure and the solid was washed with pentane and dried *in vacuo* to give product **4d** as a yellow solid (90.7 mg, 85% yield).

^{1}H NMR (400 MHz, CD_2Cl_2): δ 7.98 – 7.89 (m, 1H), 7.86 – 7.78 (m, 1H), 7.78 – 7.63 (m, 5H), 7.57 (tdd, J = 7.6, 3.3, 1.7 Hz, 3H), 7.42 – 7.35 (m, 2H), 7.23 – 7.07 (m, 2H), 6.86 (d, J = 2.0 Hz, 1H), 6.03 (d, J = 1.9 Hz, 1H), 3.17 (dtt, J = 19.4, 12.9, 3.4 Hz, 1H, ONPC$\boldsymbol{H^a}$), 2.67 – 2.43 (m, 2H), 2.41 – 2.18 (m, 3H), 2.17 – 2.06 (m, 1H), 1.94 – 1.72 (m, 2H), 1.56 – 1.47 (m, 1H), 1.38 (s, 9H), 1.09 (s, 9H).

^{11}B NMR (128 MHz, CD_2Cl_2): δ -16.7.

^{13}C{^{1}H} NMR (101 MHz, CD_2Cl_2): δ 149.9 – 149.5 (m, $BArF_{20}$), 147.5 – 147.2 (m, $BArF_{20}$), 147.0 (dd, J = 18.7, 6.3 Hz, 1C), 146.5 (d, J = 12.0 Hz, 1C), 146.3 (s, 1C), 140.0 – 139.6 (m, $BArF_{20}$), 138.4 (d, J = 7.9 Hz, 1C), 138.2 (d, J = 2.5 Hz, 1C), 138.0 – 137.7 (m, $BArF_{20}$), 137.6 – 137.2 (m, $BArF_{20}$), 136.7 (s, 1C), 135.5 (dd, J = 8.2, 2.9 Hz, 1C), 135.5 – 135.2 (m, $BArF_{20}$), 134.8 (d, J = 9.0 Hz, 2C), 133.5 (d, J = 2.2 Hz, 1C), 132.7 (d, J = 9.1 Hz, 2C), 131.2 (d, J = 12.3 Hz, 2C), 130.6 (d, J = 12.0 Hz, 2C), 128.6 (dd, J = 7.7, 2.2 Hz, 1C), 128.3 (d, J = 12.0 Hz, 1C), 118.4 (s, 1C), 118.2 (d, J = 6.7 Hz, 1C), 118.1 (s, 1C), 117.4 (d, J = 17.1 Hz, 1C), 117.3 (d, J = 4.5 Hz, 1C), 107.3 (s, 1C), 42.6 (dd, J = 37.4, 3.1 Hz, 1C), 36.6 (dd, J = 44.6, 16.0 Hz, 1C), 34.9 (s, 1C), 34.8 (s, 1C), 31.6 (s, 3C), 29.7 (s, 3C), 27.7 (d, J = 13.5 Hz, 1C), 26.0 (dd, J = 12.7, 10.1 Hz, 1C), 22.2 (dd, J = 11.8, 3.4 Hz, 1C), 21.7 (dd, J = 6.8, 1.7 Hz, 1C).

^{19}F NMR (376 MHz, CD_2Cl_2): δ -132.9 – -133.2 (m), -163.6 (t, J = 20.4 Hz), -167.5 (t, J = 19.3 Hz).

^{31}P{^{1}H} NMR (162 MHz, CD_2Cl_2): δ 163.9 (d, J = 4.3 Hz, O***P***N), 28.2 (d, J = 4.2 Hz, ***P***Ph$_2$).

HRMS (ESI): $[C_{38}H_{44}NOP_2]^+$, calculated: 592.2893, found: 592.2894.

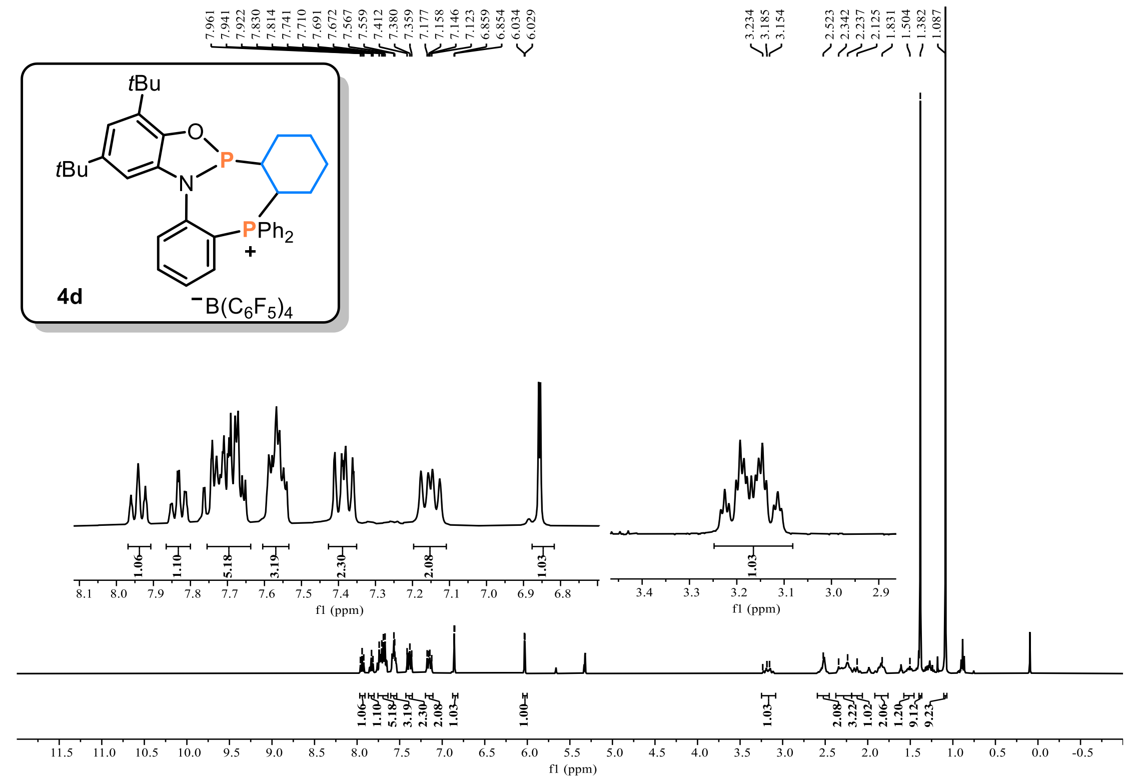

Figure S52: ^{1}H NMR of **4d** (400 MHz, CD_2Cl_2).

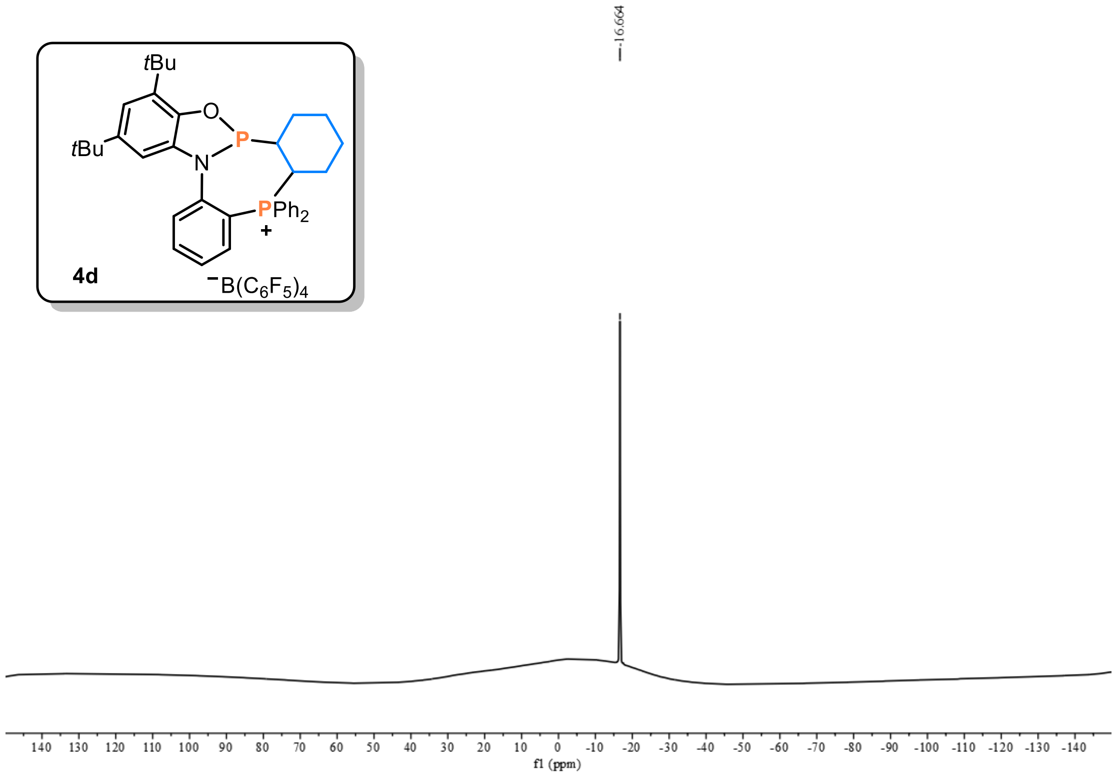

Figure S53: ^{11}B NMR of **4d** (128 MHz, CD_2Cl_2).

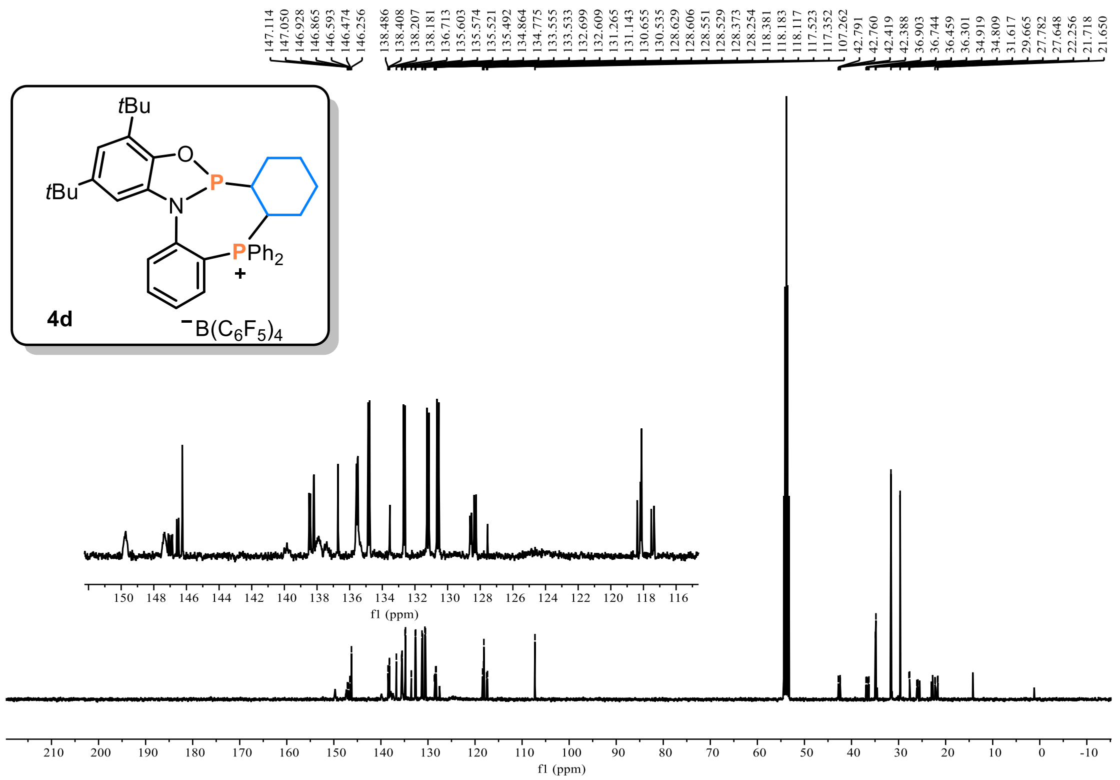

Figure S54: ^{13}**C{**^{1}**H} NMR** of **4d** (101 MHz, CD_2Cl_2).

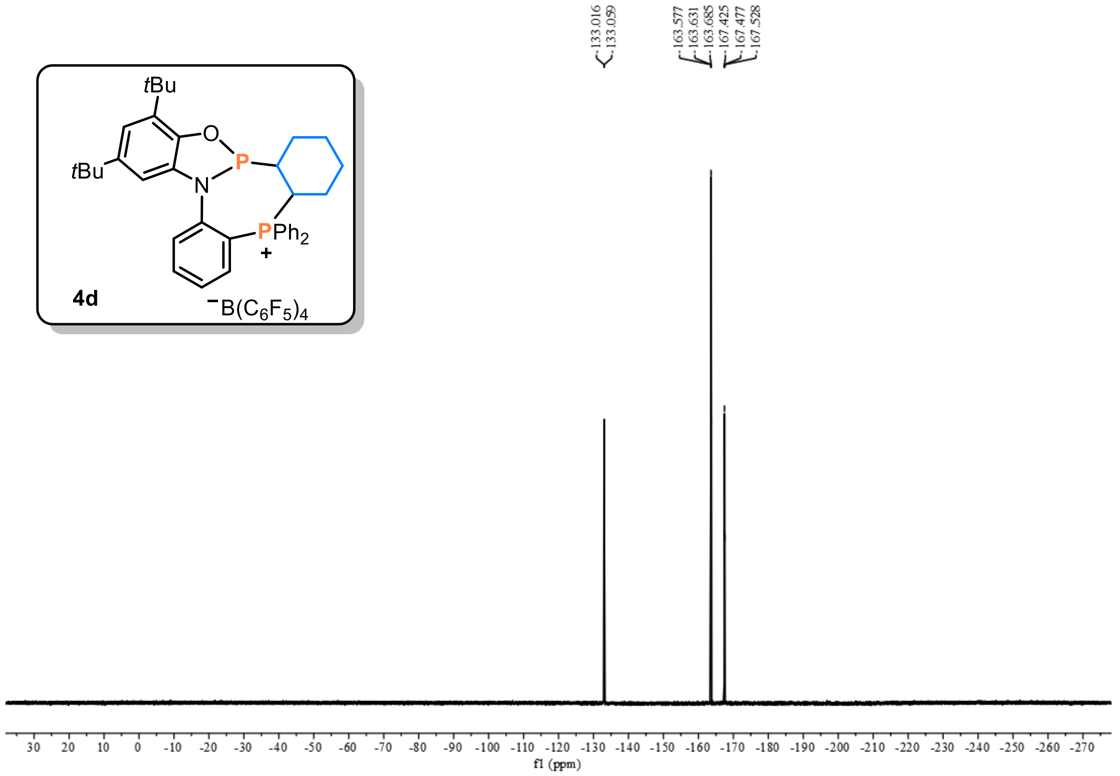

Figure S55: ^{19}**F NMR** of **4d** (376 MHz, CD_2Cl_2).

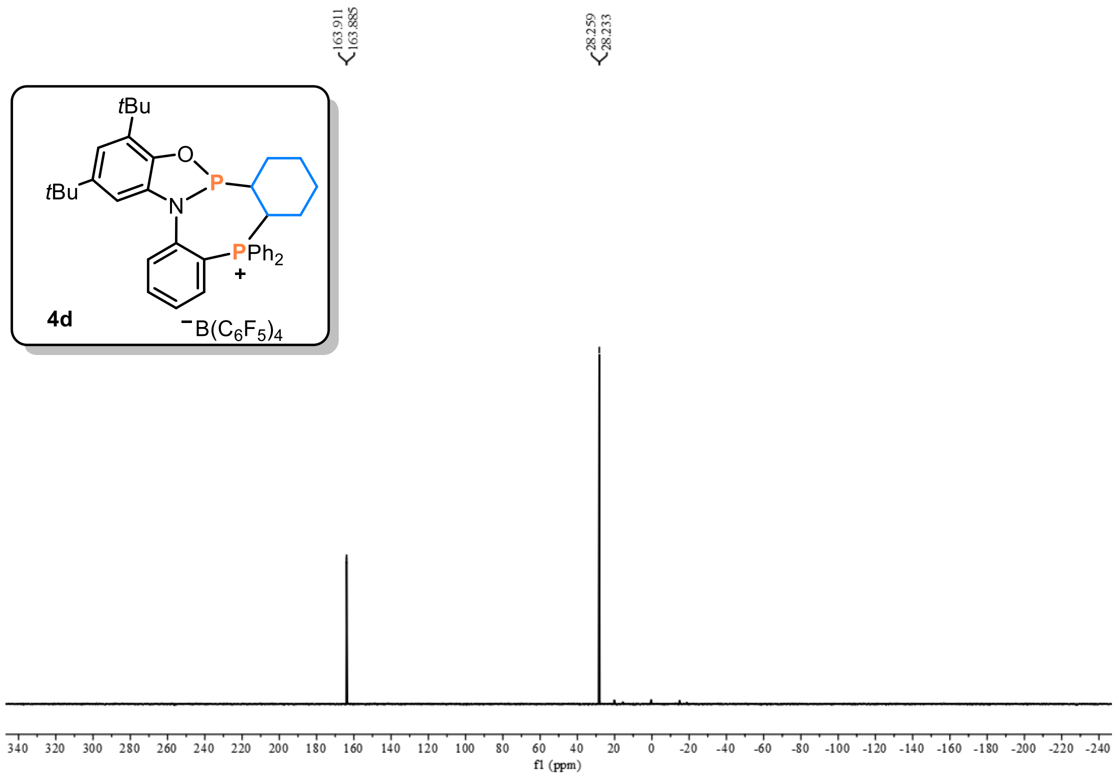

Figure S56: ^{31}P{^{1}H} NMR of **4d** (162 MHz, CD_2Cl_2).

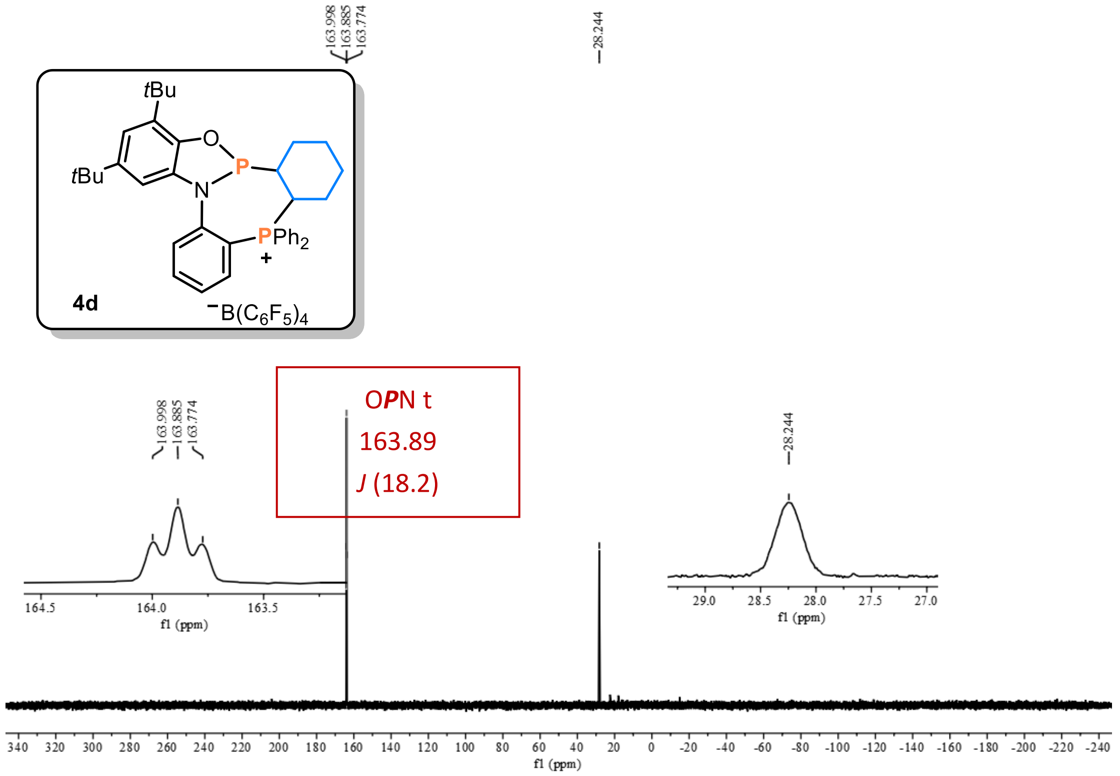

Figure S57: ^{31}P NMR of **4d** (162 MHz, CD_2Cl_2).

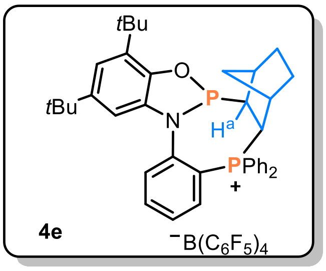

2a (100 mg, 84 µmol, 1.0 equiv) was dissolved in 2 mL CH_2Cl_2, followed by the addition of 2-norbornene (15.8 mg, 168 µmol, 2.0 equiv). The mixture was stirred at room temperature for 18 h. The solvent was then removed under reduced pressure and the solid was washed with pentane and dried *in vacuo* to give product **4e** as an off-white solid (60.6 mg, 56% yield).

^{1}H NMR (600 MHz, CD_2Cl_2): δ 7.90 – 7.81 (m, 3H), 7.79 – 7.64 (m, 9H), 7.31 – 7.25 (m, 1H), 7.17 (dddd, J = 8.2, 7.2, 2.3, 1.1 Hz, 1H), 7.10 (d, J = 2.0 Hz, 1H), 6.97 (ddd, J = 14.9, 8.3, 1.5 Hz, 1H), 3.20 (q, J = 9.9 Hz, 1H, ONPC***H****^{a}*), 2.90 – 2.67 (m, 2H), 2.47 (d, J = 5.1 Hz, 1H), 1.79 (dtd, J = 13.8, 9.0, 4.4 Hz, 1H), 1.69 (dq, J = 11.5, 3.9 Hz, 1H), 1.40 (s, 9H), 1.35 – 1.30 (m, 11H), 1.25 (d, J = 11.9 Hz, 2H).

^{11}B NMR (193 MHz, CD_2Cl_2): δ -16.7.

^{13}C{^{1}H} NMR (151 MHz, CD_2Cl_2): δ 149.5 – 149.2 (m, $BArF_{20}$), 147.9 – 147.6 (m, $BArF_{20}$), 146.9 (dd, J = 12.6, 6.8 Hz, 1C), 146.9 (s, 1C), 146.3 (d, J = 11.0 Hz, 1C), 139.6 – 139.3 (m, $BArF_{20}$), 137.9 (s, 1C), 137.9 – 137.7 (m, $BArF_{20}$), 137.7 – 137.4 (m, $BArF_{20}$), 137.0 (d, J = 8.0 Hz, 1C), 136.7 (dd, J = 10.2, 5.1 Hz, 1C), 136.1 (d, J = 2.5 Hz, 1C), 136.0 – 135.7 (m, $BArF_{20}$), 135.7 (d, J = 3.3 Hz, 1C), 135.4 (d, J = 2.9 Hz, 1C), 133.3 (d, J = 8.8 Hz, 2C), 131.3 (s, 1C), 131.1 (d, J = 12.2 Hz, 2C), 130.2 (d, J = 12.7 Hz, 2C), 125.9 (d, J = 11.7 Hz, 1C), 124.0 (d, J = 2.8 Hz, 1C), 123.4 (d, J = 2.8 Hz, 1C), 123.2 (dd, J = 9.2, 2.8 Hz, 1C), 121.7 (d, J = 4.9 Hz, 1C), 121.2 (d, J = 5.1 Hz, 1C), 118.8 (s, 1C), 109.6 (s, 1C), 42.8 (dd, J = 37.0, 13.5 Hz, 1C), 41.7 (s, 1C), 37.0 (d, J = 1.0 Hz, 1C), 36.4 (dd, J = 12.3, 1.6 Hz, 1C), 35.4 (s, 1C), 35.0 (s, 1C), 34.5 (s, 1C), 32.0 (d, J = 14.2 Hz, 1C), 31.7 (s, 3C), 30.8 (d, J = 4.8 Hz, 1C), 29.6 (s, 3C).

^{19}F NMR (565 MHz, CD_2Cl_2): δ -133.0 – -133.2 (m), -163.7 (t, J = 20.5 Hz), -167.6 (t, J = 19.3 Hz).

^{31}P{^{1}H} NMR (243 MHz, CD_2Cl_2): δ 127.9 (d, J = 43.7 Hz, O***P***N), 31.1 (d, J = 43.5 Hz, ***P***Ph_2).

HRMS (ESI): $[C_{39}H_{44}ClNOP_2]^+$, calculated: 604.2893, found: 604.2895.

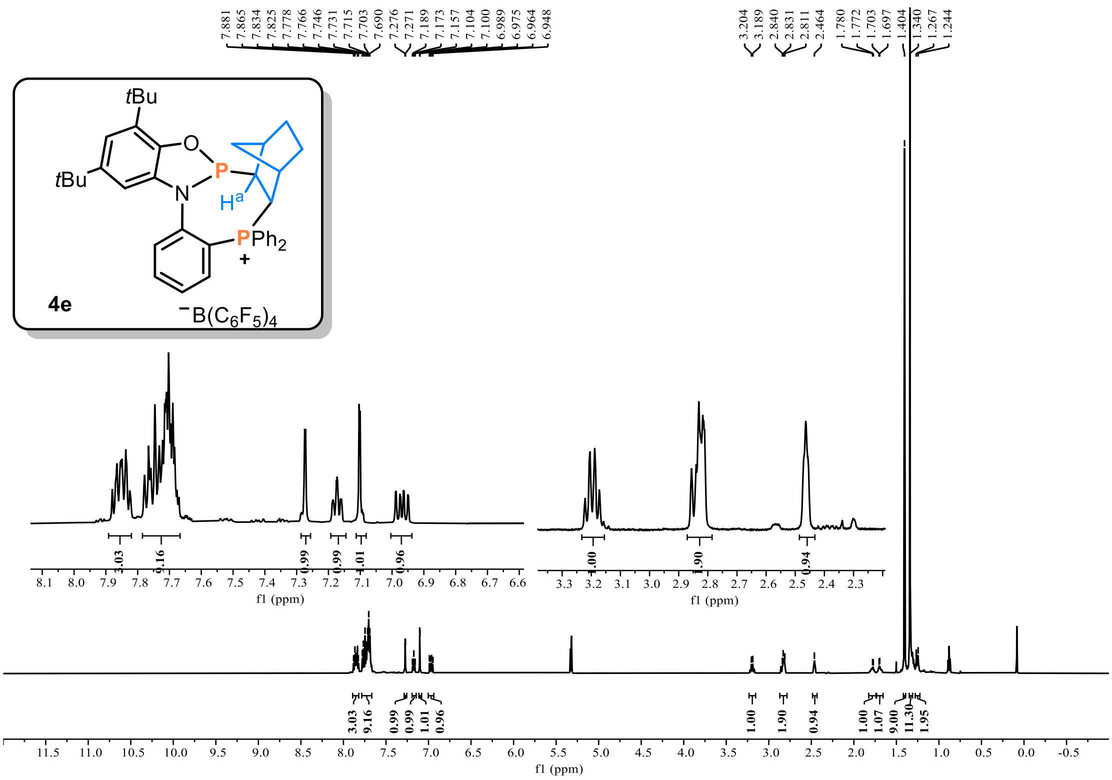

Figure S58: ^{1}H NMR of **4e** (600 MHz, CD_2Cl_2).

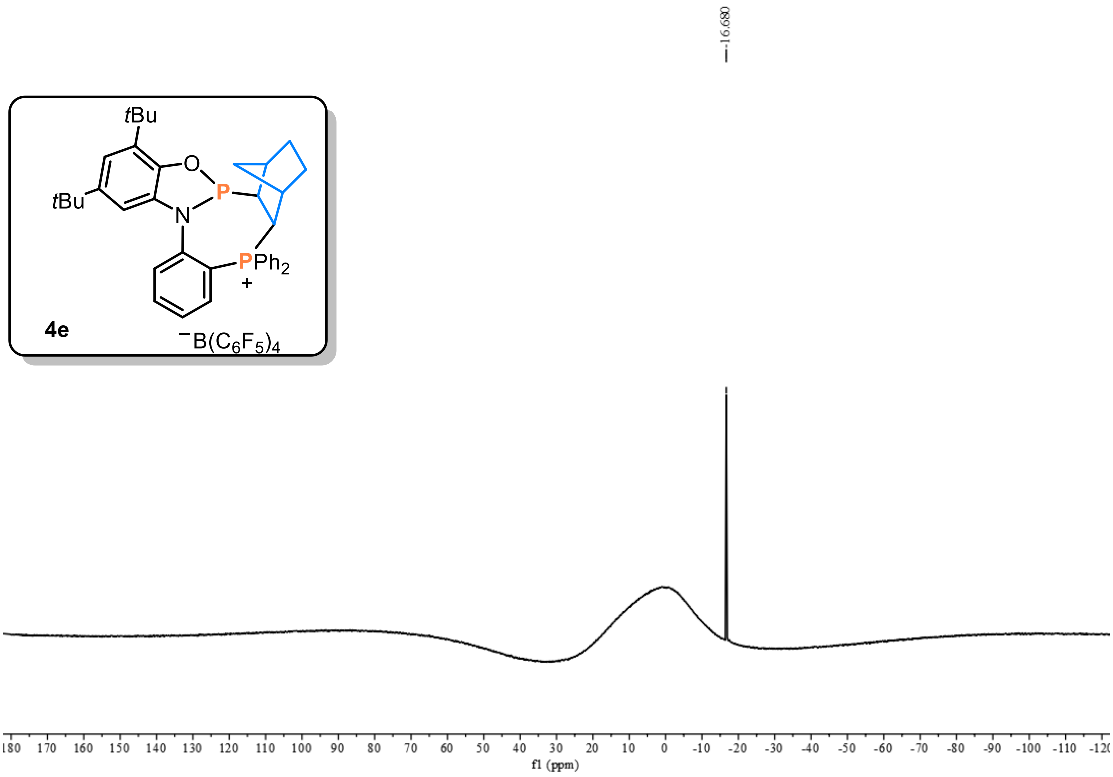

Figure S59: ^{11}B NMR of **4e** (193 MHz, CD_2Cl_2).

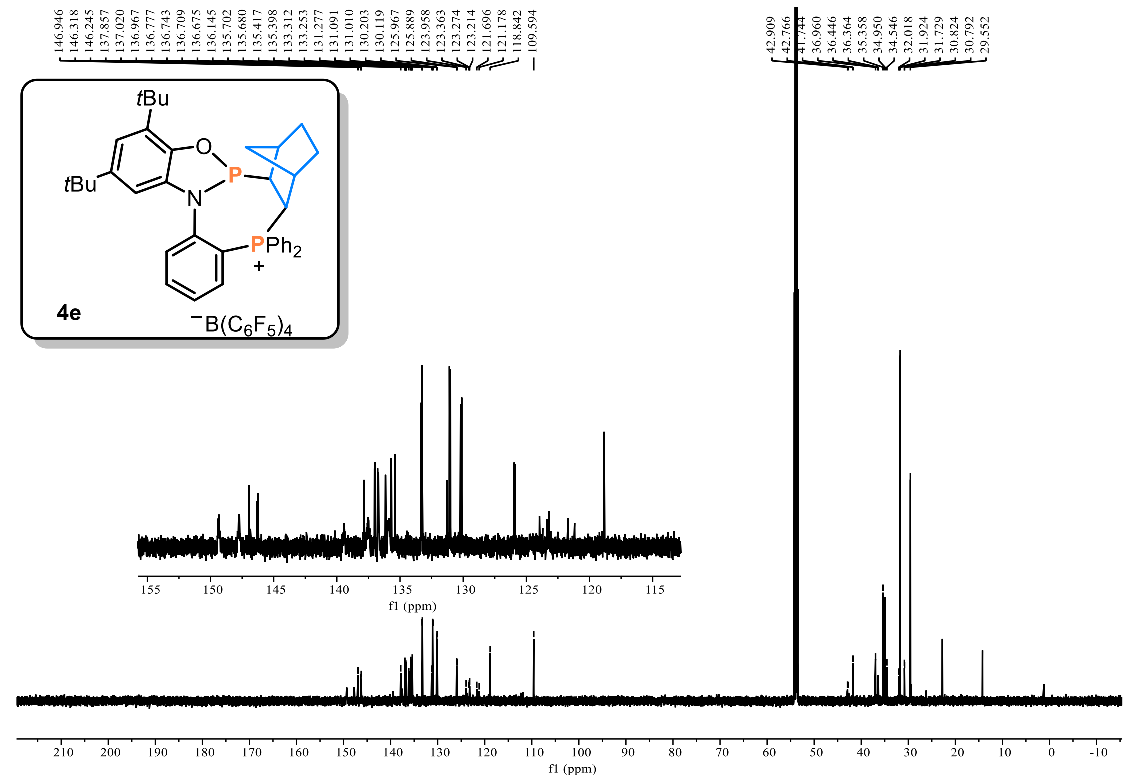

Figure S60: ^{13}**C{^{1}H} NMR** of **4e** (151 MHz, CD_2Cl_2).

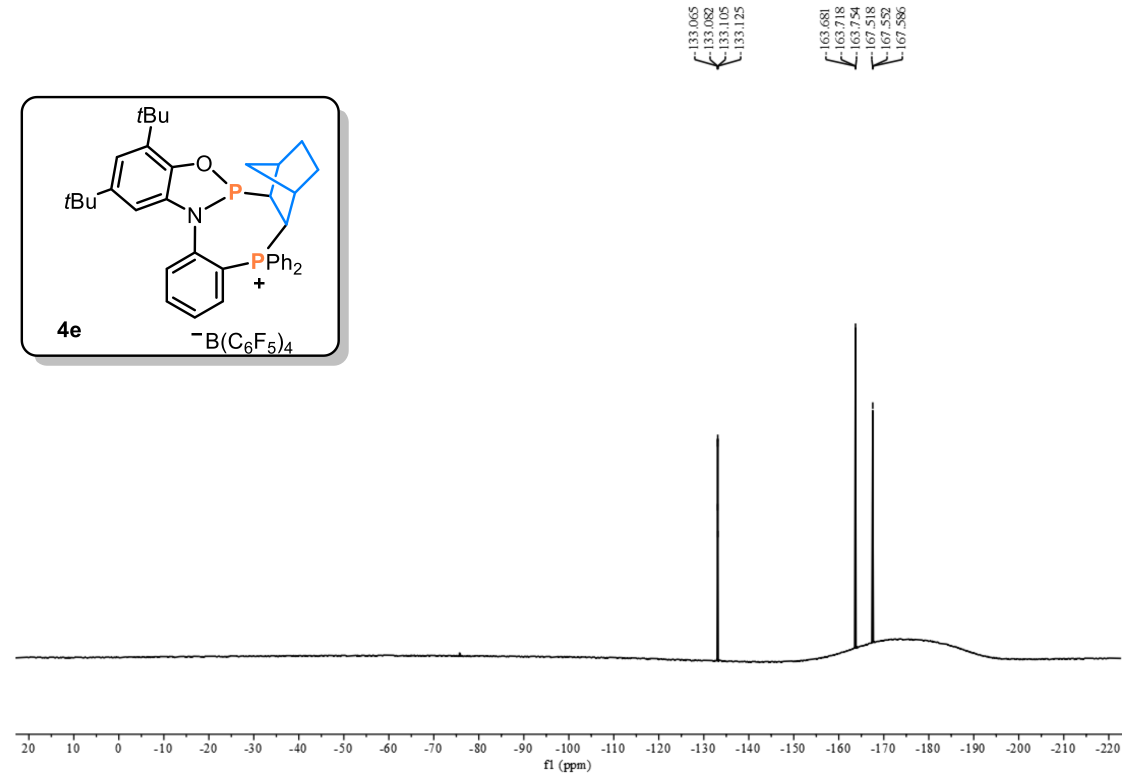

Figure S61: ^{19}**F NMR** of **4e** (565 MHz, CD_2Cl_2).

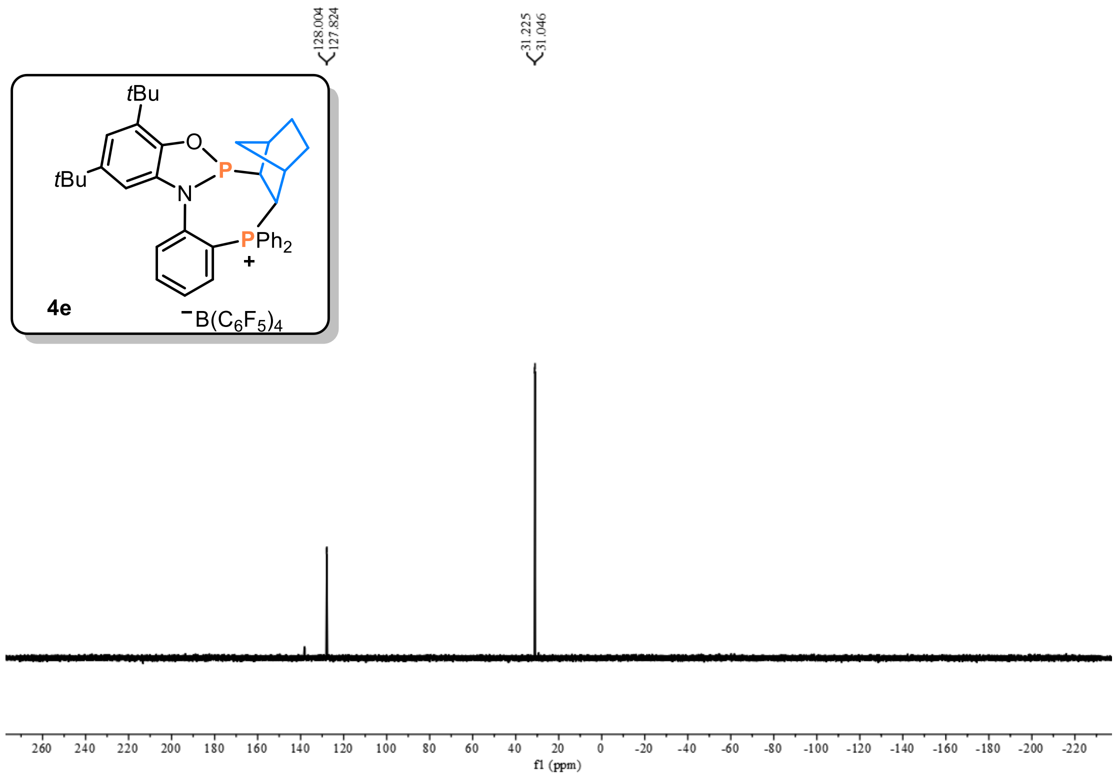

Figure S62: ^{31}**P{**^{1}**H} NMR** of **4e** (243 MHz, CD_2Cl_2).

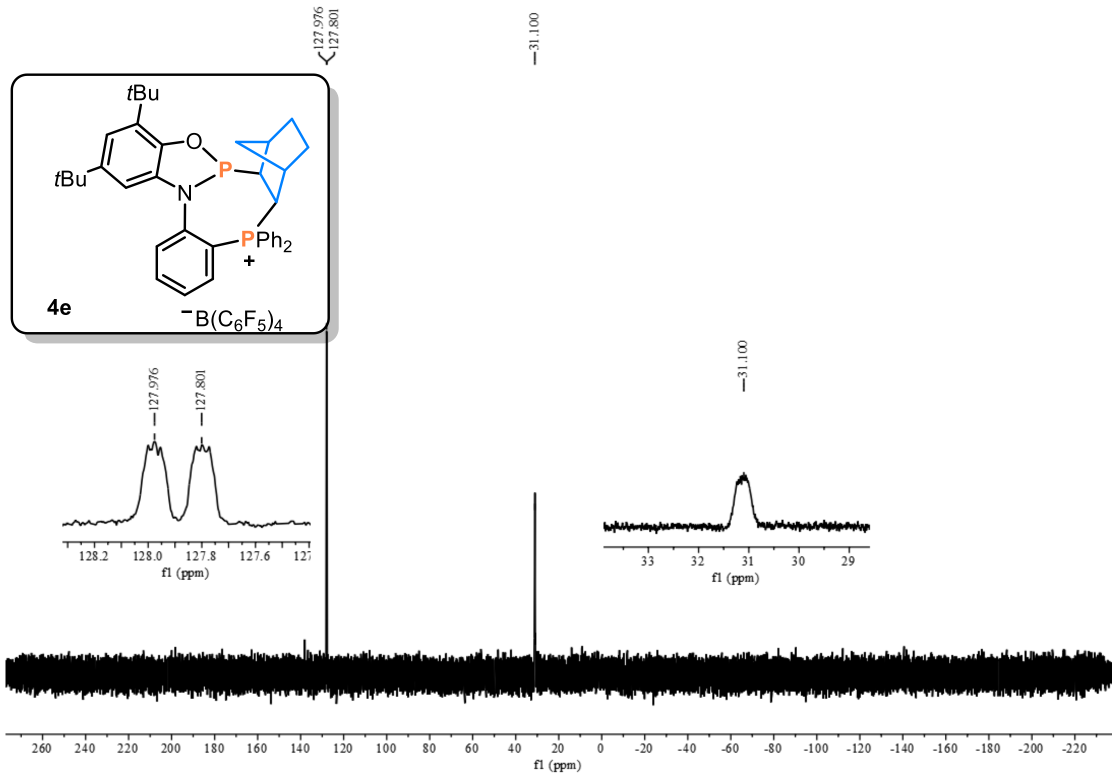

Figure S63: ^{31}**P NMR** of **4e** (243 MHz, CD_2Cl_2).

5. Phosphinophosphination of Benzaldehyde

5.1 Reaction development

The yellow phosphenium salt **2a** (15 mg, 12.6 µmol, 1.0 equiv) and benzaldehyde (2.0 mg, 18.9 µmol, 1.5 equiv) were weighed into a J. Young type NMR tube. A colorless solution was obtained after adding CD_2Cl_2 (0.5 mL), indicating full conversion of the starting material. Two diastereomeric addition products were formed after 5 hours as suggested by the multinuclear NMR data. The major one has two doublets at 126.0 and 30.3 ppm ($^3J_{PP}$ = 5.3 Hz) while the minor one has two singlets at 116.4 and 29.3 ppm (Figure S64). The **^{1}H NMR** spectrum showed resonances at 6.53 ppm (t, J = 4.2 Hz) for major diastereomer and 5.77 ppm (d, J = 9.9 Hz) for minor diastereomer (Figure S65).

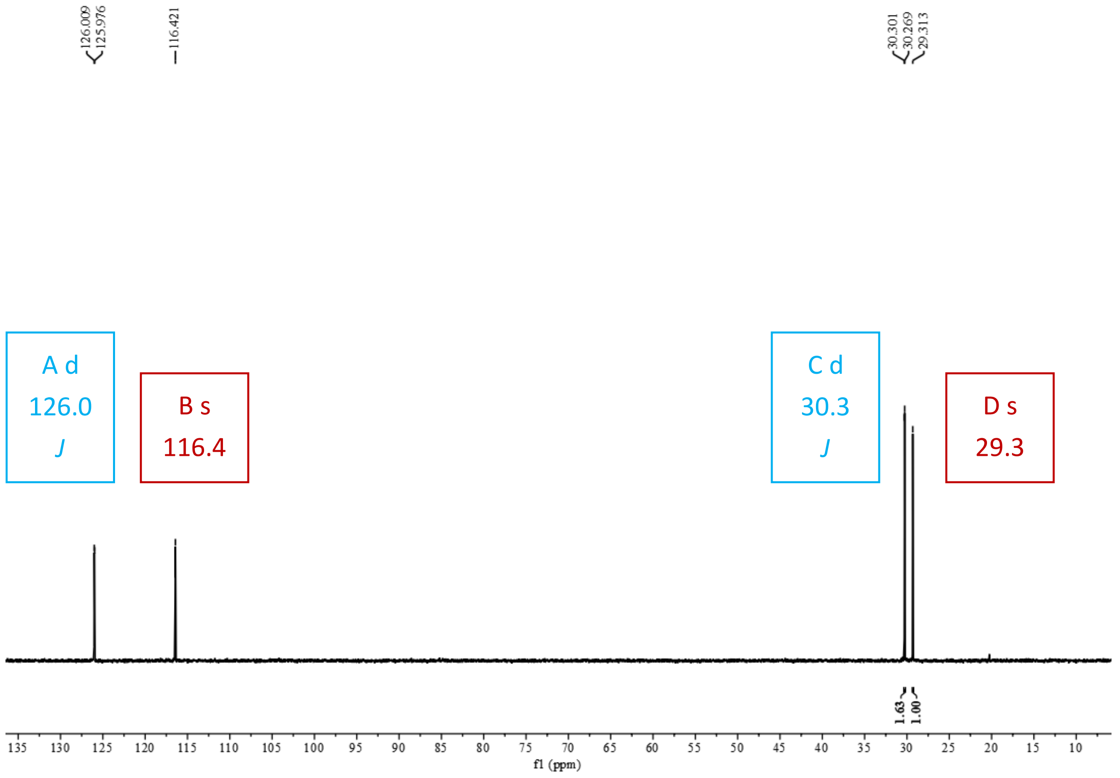

Figure S64: ^{31}P{^{1}H} NMR (400 MHz) spectra of **[PNOP][B(C_6F_5)$_4$]** with benzaldehyde in CD_2Cl_2 at room temperature after 5 h.

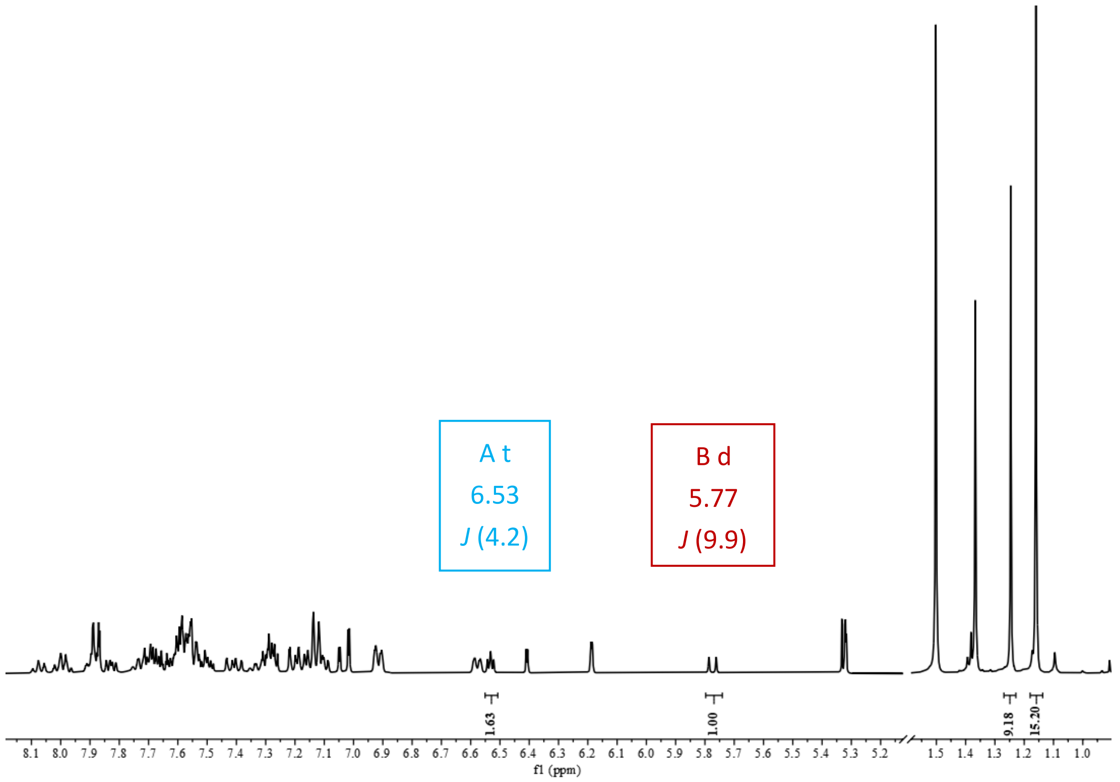

Figure S65: ^{1}**H NMR** (400 MHz) spectra of **[PNOP][B(C$_6$F$_5$)$_4$]** with benzaldehyde in CD_2Cl_2 at room temperature after 5 h.

The solution was probed by ^{1}**H** and ^{31}**P{^{1}H} NMR** over time. The combined, multinuclear spectral data indicated the conversion from one diastereomer to the other one (Scheme S3 and Figure S66-S68). Only the diastereomer with two singlets at 116.4 and 29.3 ppm was observed finally.

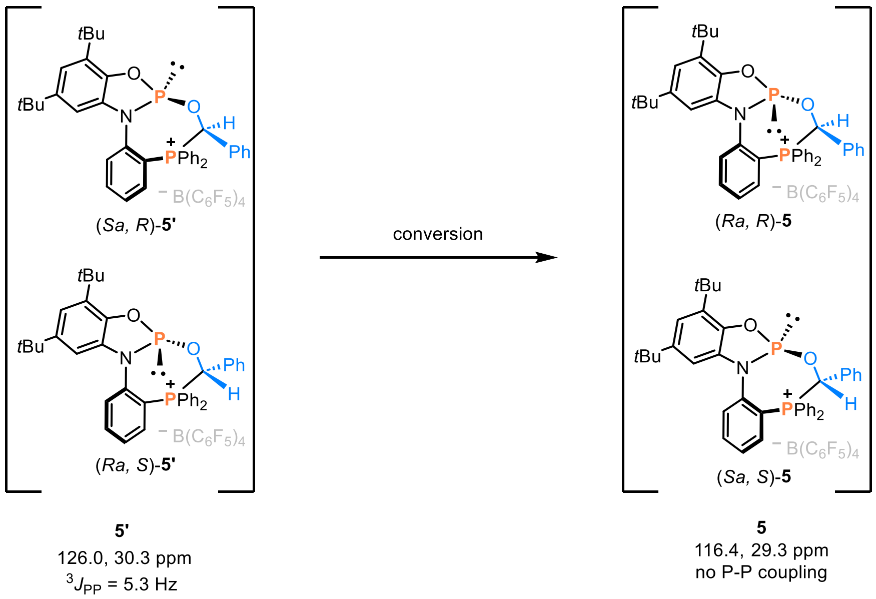

Scheme S3: The conversion from one racemate **5'** to the more stable racemate **5**.

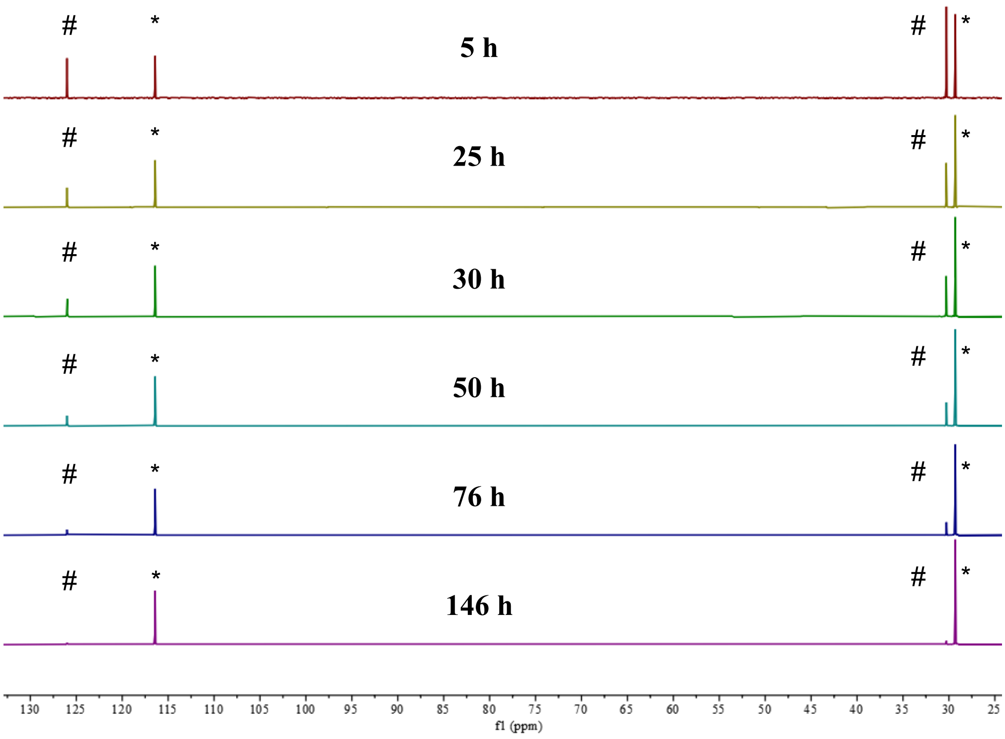

Figure S66: Stacked $^{31}P\{^1H\}$ **NMR** (162 MHz) spectra of **2a** with benzaldehyde in CD_2Cl_2 over time. # denotes signals of **5’**, * denotes signals of more stable product **5**.

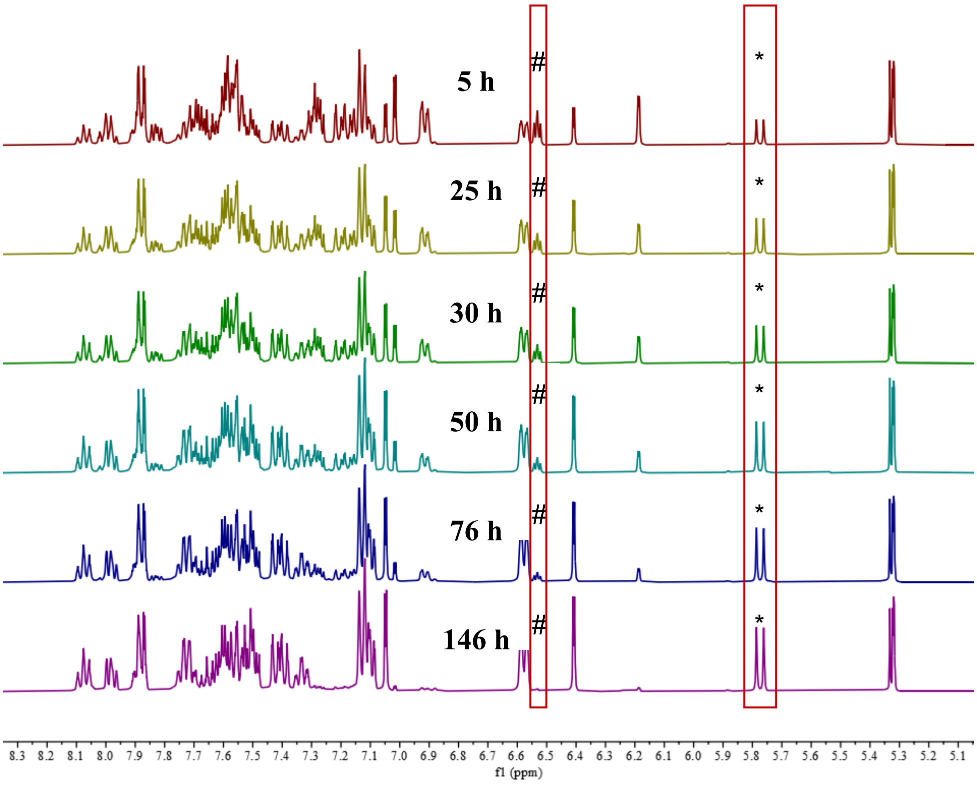

Figure S67: Stacked ^{1}**H NMR** (400 MHz) spectra of **2a** with benzaldehyde in CD_2Cl_2 at over time. # denotes protons of **5’**, * denotes protons of more stable product **5**.

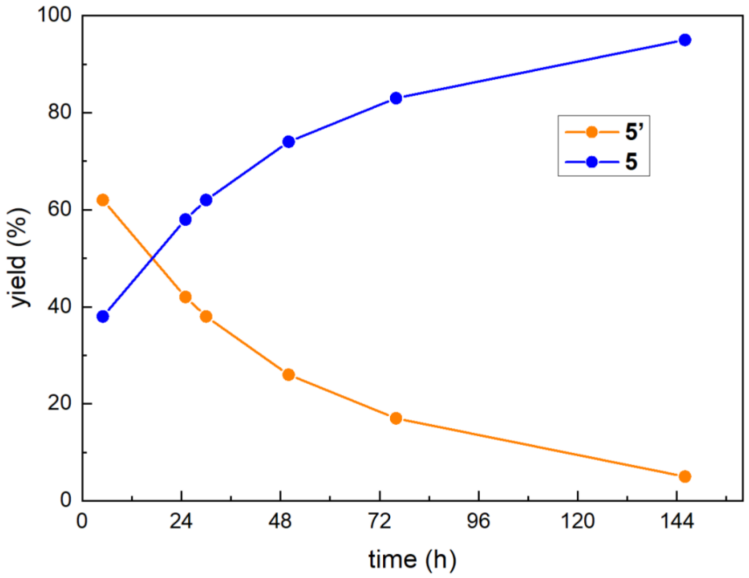

Figure S68: The yields of racemates **5'** and **5** over time. Yields were calculated based on the integration area of *tert*-butyl group protons from ^{1}**H NMR** spectra.

5.2 Preparation of 5

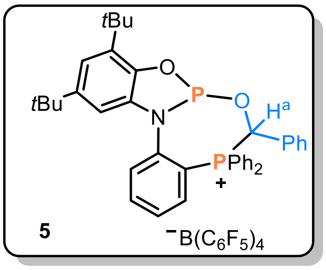

2a (100 mg, 84 µmol, 1.0 equiv) was dissolved in CH_2Cl_2 (2 mL), followed by the addition of benzaldehyde (10.7 mg, 101 µmol, 1.2 equiv). The mixture was stirred over a week, after which the volatiles were removed under reduced pressure and washed with pentane, then dried *in vacuo* to give the product **5** as a white solid (106.8 mg, 98% yield). Single crystals suitable for X-ray diffraction were grown by gas phase diffusion of pentane into the CH_2Cl_2 solution.

^{1}H NMR (600 MHz, CD_2Cl_2): δ 8.08 (tt, J = 7.8, 1.6 Hz, 1H), 8.02 – 7.95 (m, 1H), 7.89 (tdt, J = 7.6, 2.1, 1.2 Hz, 1H), 7.76 – 7.70 (m, 2H), 7.65 – 7.57 (m, 2H), 7.56 – 7.47 (m, 3H), 7.43 – 7.38 (m, 2H), 7.34 (tdt, J = 7.4, 2.4, 1.2 Hz, 1H), 7.16 – 7.08 (m, 4H), 7.04 (d, J = 2.0 Hz, 1H), 6.62 – 6.52 (m, 2H), 6.40 (d, J = 2.0 Hz, 1H), 5.77 (d, J = 9.9 Hz, 1H, OC$\boldsymbol{H}^a$Ph), 1.36 (s, 9H), 1.24 (s, 9H).

^{11}B NMR (128 MHz, CD_2Cl_2): δ -16.7.

^{13}C{^{1}H} NMR (101 MHz, CD_2Cl_2): δ 149.9 – 149.5 (m, $BArF_{20}$), 148.1 (s, 1C), 147.6 – 147.1 (m, $BArF_{20}$), 144.8 (d, J = 13.7 Hz, 1C), 140.7 (dd, J = 6.7, 5.6 Hz, 1C), 140.1 – 139.6 (m, $BArF_{20}$), 138.0 (d, J = 2.7 Hz, 1C), 138.0 – 137.7 (m, $BArF_{20}$), 137.5 (d, J = 8.3 Hz, 1C), 137.5 – 137.2 (m, $BArF_{20}$), 136.8 (d, J = 3.2 Hz, 1C), 136.3 (d, J = 3.0 Hz, 1C), 136.1 (d, J = 19.9 Hz, 1C), 135.8 (d, J = 9.1 Hz, 2C), 135.7 – 135.2 (m, $BArF_{20}$), 134.4 (dd, J = 7.8, 3.3 Hz, 1C), 133.4 (d, J = 9.1 Hz, 2C), 131.5 (dd, J = 2.1, 1.8 Hz, 1C), 131.4 (s, 1C), 131.0 (d, J = 12.3 Hz, 2C), 130.5 (d, J = 12.7 Hz, 2C), 129.3 (d, J = 2.9 Hz, 1C), 128.6 (d, J = 5.4 Hz, 1C), 128.6 (d, J = 1.0 Hz, 1C), 121.1 (dd, J = 81.6, 6.3 Hz, 1C), 117.4 (s, 1C), 116.8 (s, 2C), 116.6 (s, 1C), 114.1 (s, 1C), 113.2 (s, 1C), 104.2 (s, 1C), 73.1 (dd, J = 56.9, 5.2 Hz, 1C), 35.4 (s, 1C), 34.9 (s, 1C), 31.7 (s, 3C), 29.5 (s, 3C).

^{19}F NMR (376 MHz, CD_2Cl_2): δ -133.0 – -133.2 (m), -163.7 (t, J = 20.4 Hz), -167.6 (t, J = 19.3 Hz).

^{31}P{^{1}H} NMR (243 MHz, CD_2Cl_2): δ 116.4 (s, O***P***N), 29.3 (s, ***P***Ph_2).

HRMS (ESI): $[C_{39}H_{40}NO_2P_2]^+$, calculated: 616.2529, found: 616.2531.

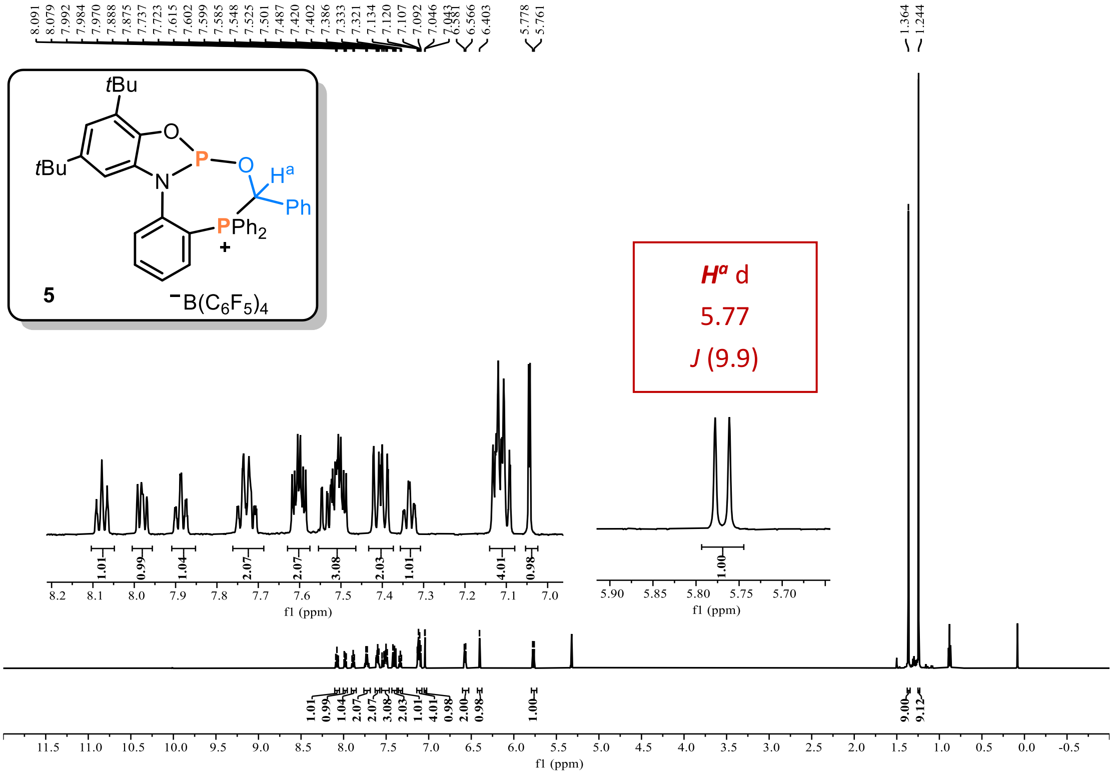

Figure S69: ^{1}**H NMR** of **5** (600 MHz, CD_2Cl_2).

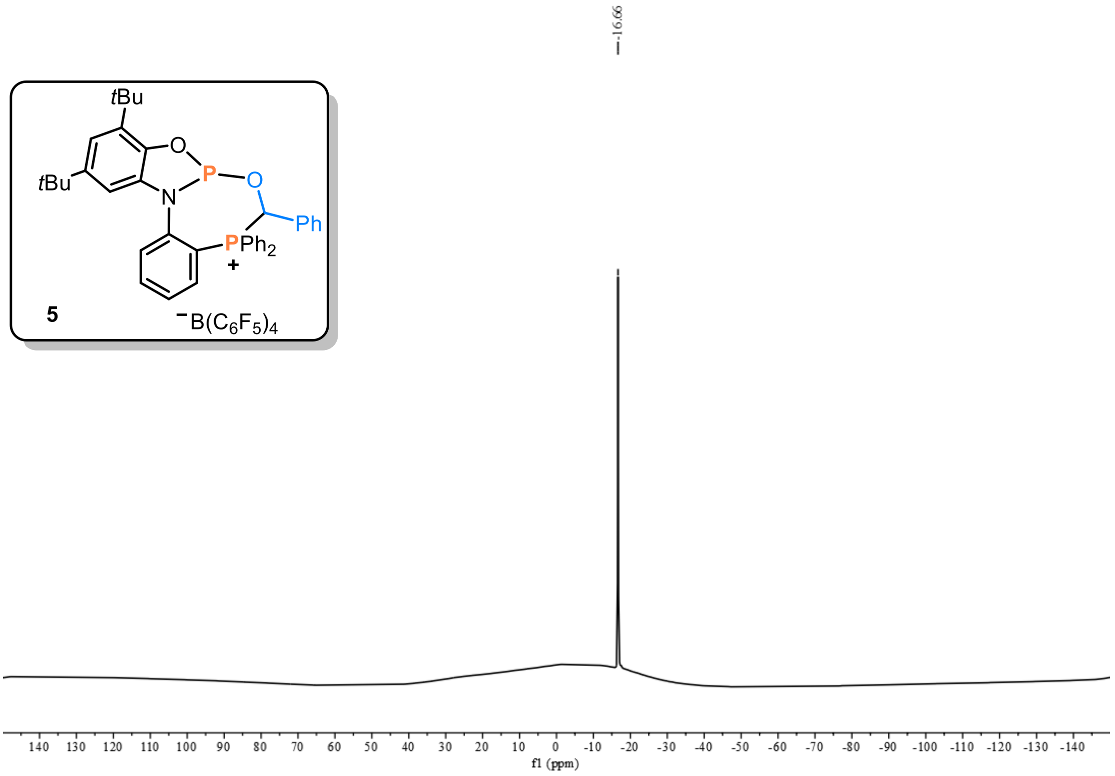

Figure S70: ^{11}**B NMR** of **5** (128 MHz, CD_2Cl_2).

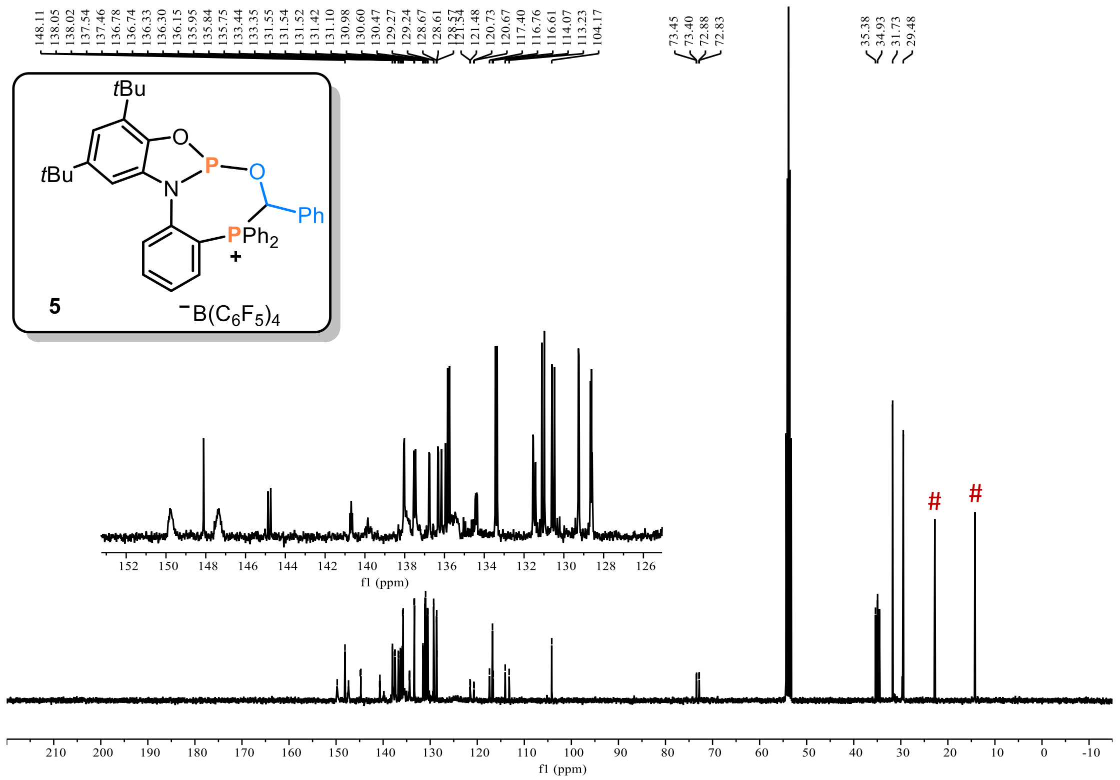

Figure S71: ^{13}**C{**^{1}**H} NMR** of **5** (101MHz, CD_2Cl_2). # denotes pentane.

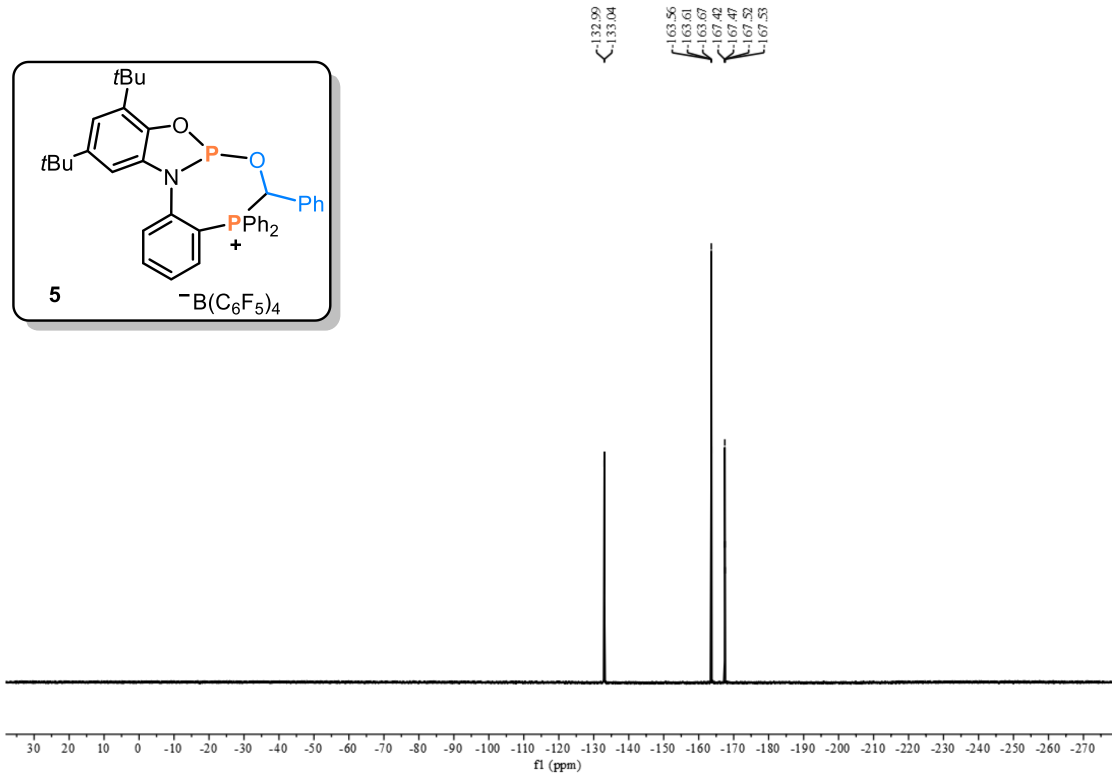

Figure S72: ^{19}**F NMR** of **5** (376 MHz, CD_2Cl_2).

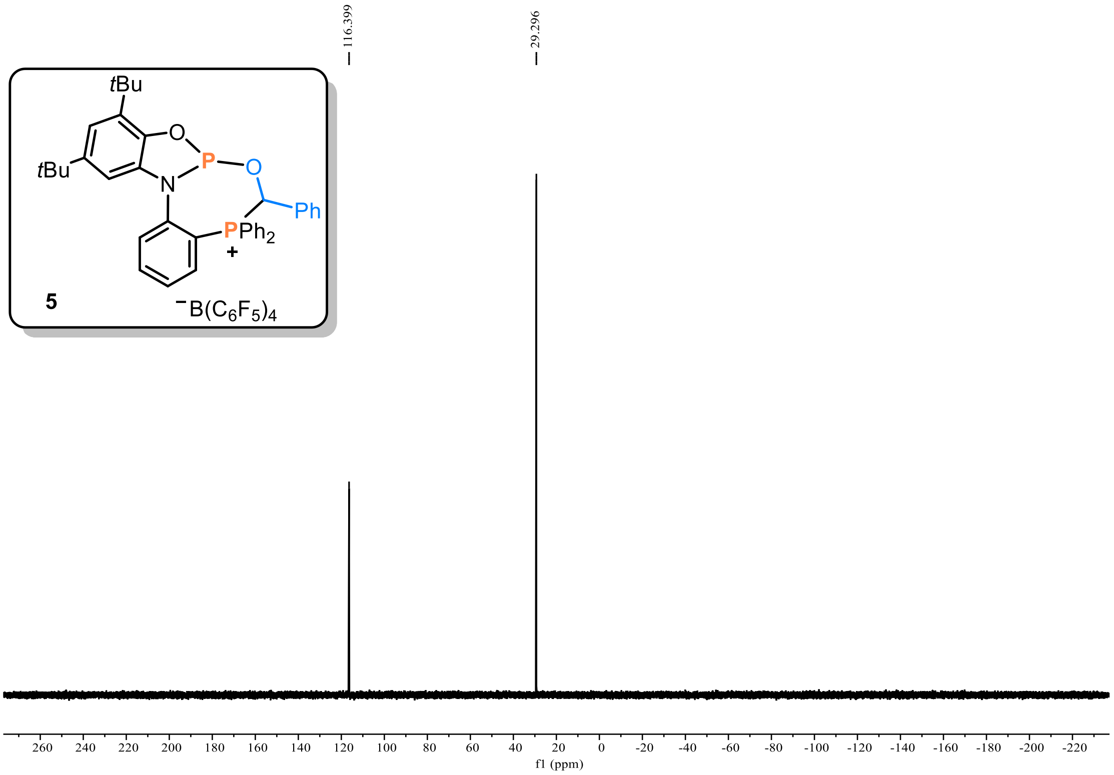

Figure S73: ^{31}**P{**^{1}**H} NMR** of **5** (243 MHz, CD_2Cl_2).

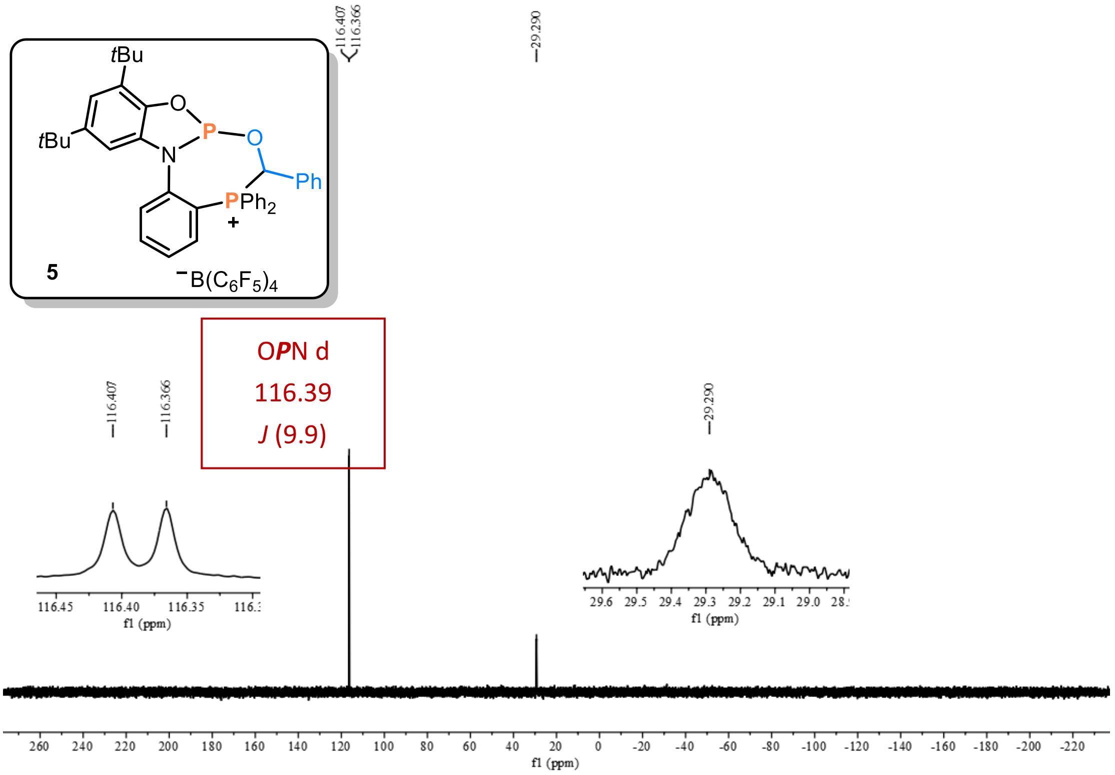

Figure S74: ^{31}**P NMR** of **5** (243 MHz, CD_2Cl_2).

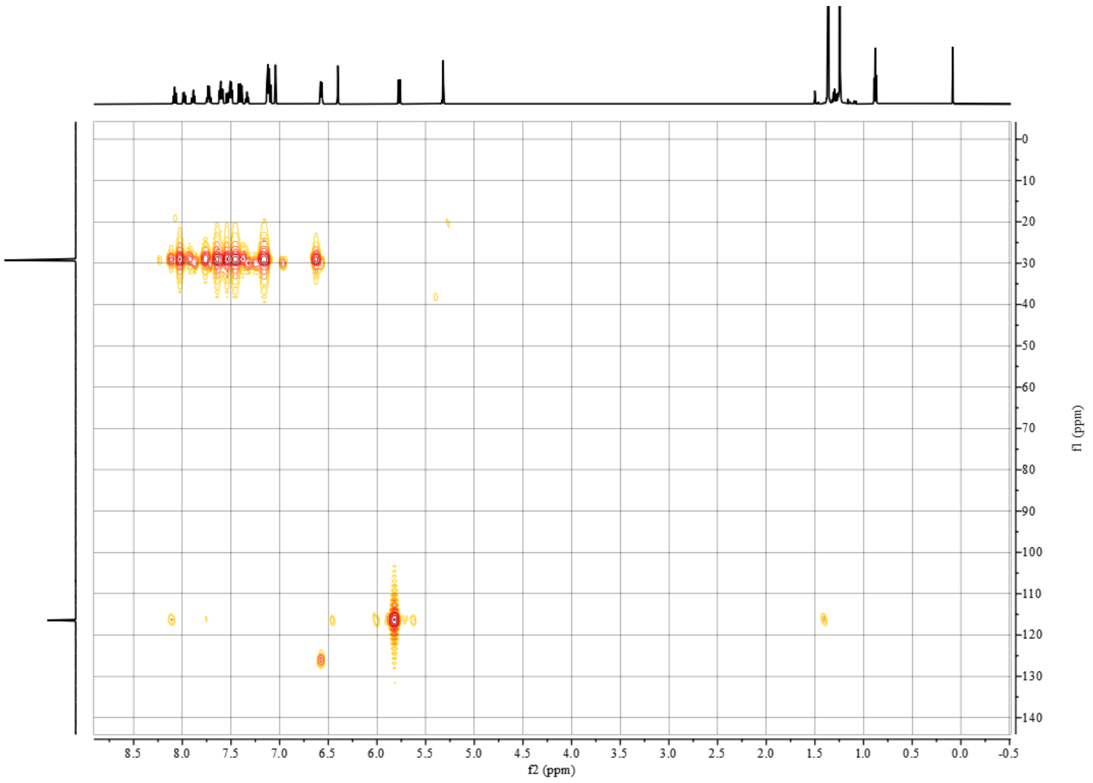

Figure S75: ^{1}H-^{31}P HMBC NMR of **5** (162 MHz, CD_2Cl_2).

6. Phosphinophosphination of Ketones

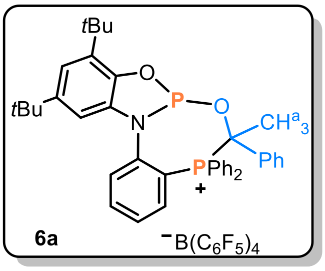

2a (100 mg, 84 μmol, 1.0 equiv) was dissolved in CH_2Cl_2 (2 mL), followed by the addition of acetophenone (40.4 mg, 336 μmol, 4.0 equiv). The mixture was stirred over a week, after which the volatiles were removed under reduced pressure and washed with pentane, then dried *in vacuo* to give a set of diastereomers of product **6a** in a 4:1 ratio as a white solid (87.5 mg, 79% yield). Single crystals of major diastereomer suitable for X-ray diffraction were grown by gas phase diffusion of pentane into the CH_2Cl_2 solution.

^{1}H NMR (600 MHz, CD_2Cl_2) *major diastereomer*: δ 8.09 (ddd, J = 12.4, 8.2, 1.4 Hz, 1H), 8.04 – 7.96 (m, 2H), 7.80 – 7.68 (m, 4H), 7.61 – 7.55 (m, 2H), 7.37 (tt, J = 6.2, 1.3 Hz, 1H), 7.29 – 7.24 (m, 1H), 7.21 (ddd, J = 11.9, 8.5, 1.4 Hz, 2H), 7.14 (t, J = 7.7 Hz, 2H), 7.09 (ddd, J = 8.1, 6.8, 3.9 Hz, 2H), 6.90 (d, J = 2.0 Hz, 1H), 6.81 – 6.75 (m, 2H), 5.65 (d, J = 2.0 Hz, 1H), 2.62 (dd, J = 16.4, 3.9 Hz, 3H, OCC$\boldsymbol{H^a_3}$Ph), 1.56 (s, 9H), 1.05 (s, 9H). *minor diastereomer*: δ 1.81 (d, J = 16.1 Hz, 3H, OCC$\boldsymbol{H^a_3}$Ph), 1.41 (s, 9H), 1.32 (s, 9H). The aromatic region protons of minor diastereomer could not be reliably assigned due to low intensity and an overlap of signals.

^{11}B NMR (193 MHz, CD_2Cl_2): δ -16.7.

^{13}C{^{1}H} NMR (151 MHz, CD_2Cl_2): The signals could not be reliably assigned due to low intensity and an overlap of signals.

^{19}F NMR (376 MHz, CD_2Cl_2): δ -132.8 – -133.2 (m), -163.5 (t, J = 20.4 Hz), -167.4 (t, J = 19.8 Hz).

^{31}P{^{1}H} NMR (243 MHz, CD_2Cl_2): δ 125.3 (d, J = 10.9 Hz, major product, O***P***N), 119.4 (d, J = 6.5 Hz, minor product, O***P***N), 36.9 (d, J = 6.3 Hz, minor product, ***P***Ph$_2$), 34.2 (d, J = 10.9 Hz, major product, ***P***Ph$_2$).

HRMS (ESI): $[C_{40}H_{42}NO_2P_2]^+$, calculated: 630.2685, found: 630.2692.

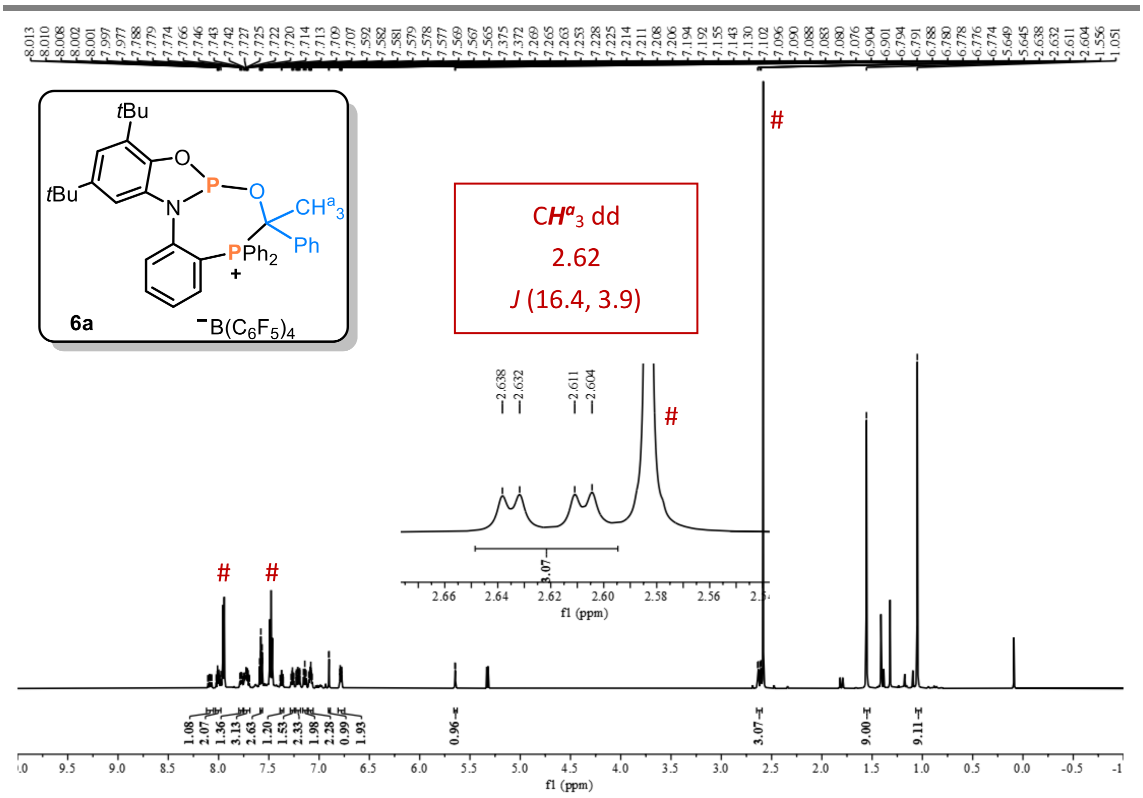

Figure S76: ^{1}**H NMR** of **6a** (600 MHz, CD_2Cl_2), Only peaks for major product are labeled and integrated. # denotes acetophenone.

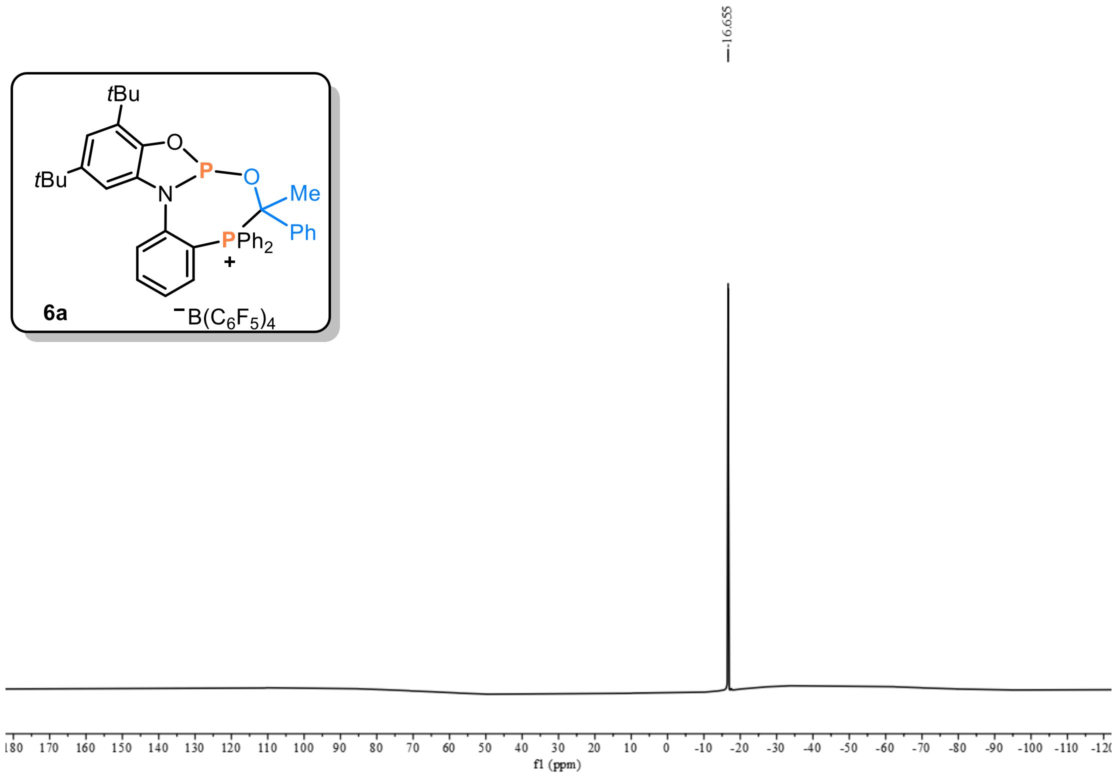

Figure S77: ^{11}**B NMR** of **6a** (193 MHz, CD_2Cl_2).

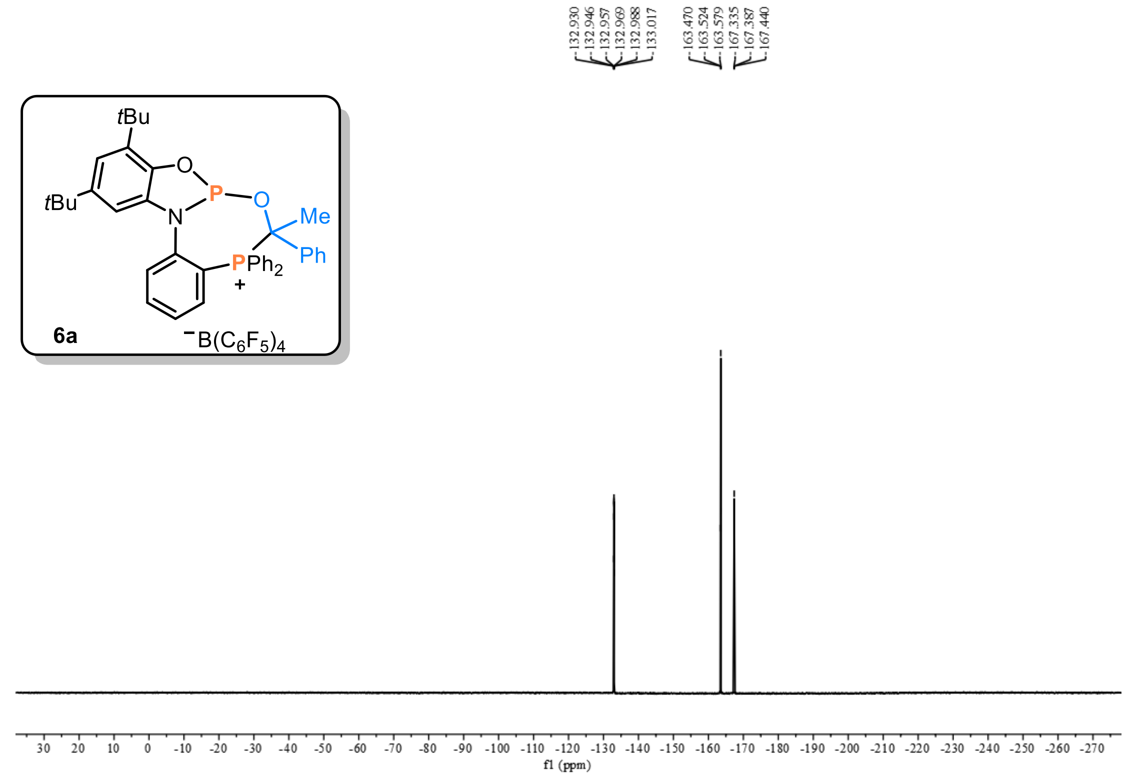

Figure S78: ^{19}**F NMR** of **6a** (376 MHz, CD_2Cl_2).

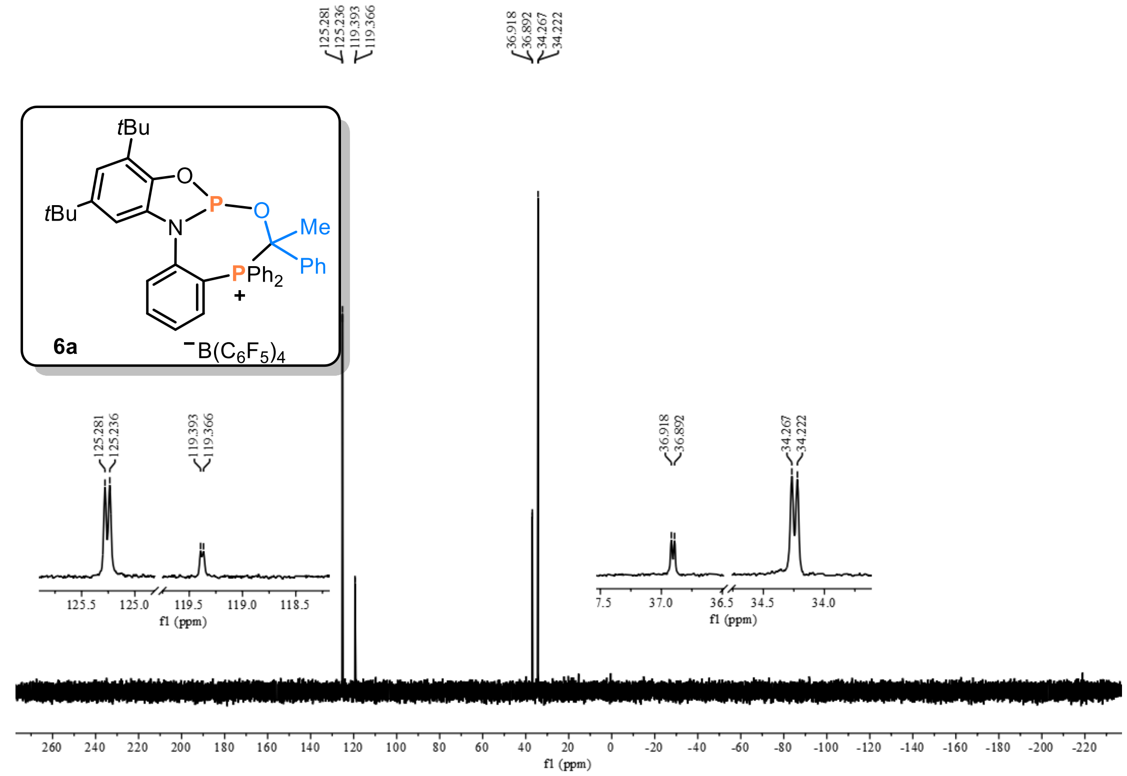

Figure S79: ^{31}**P{^{1}H} NMR** of **6a** (243 MHz, CD_2Cl_2).

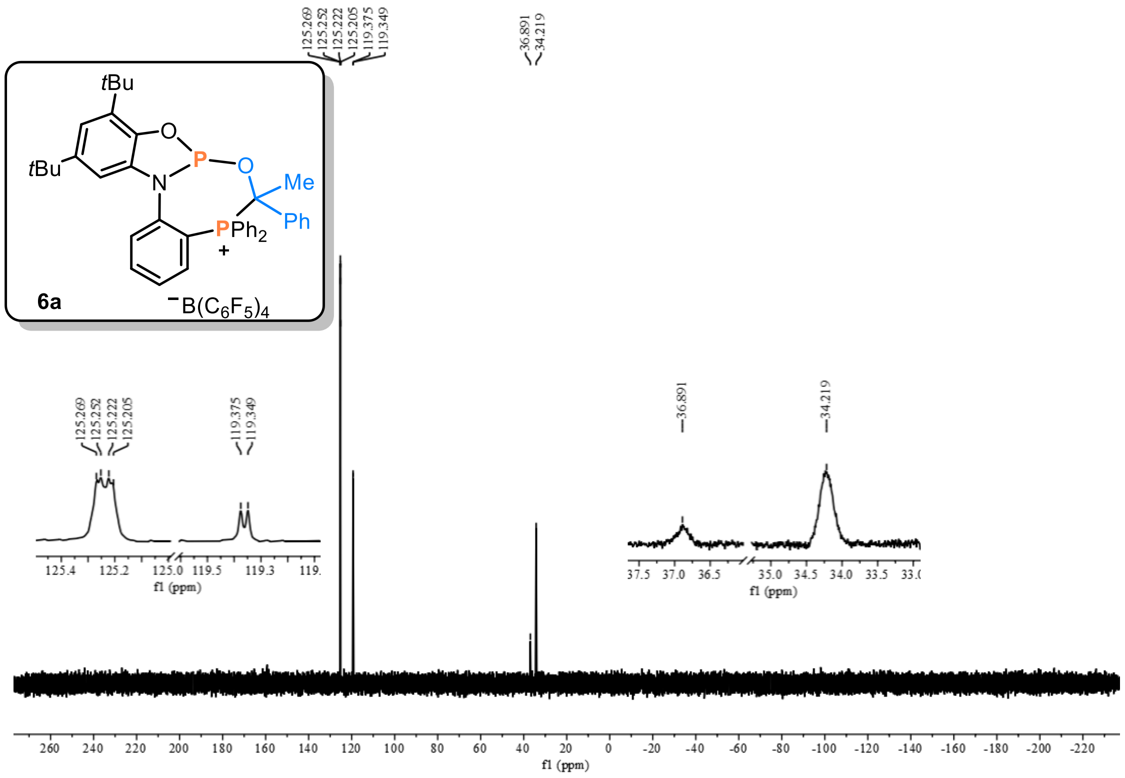

Figure S80: ^{31}**P NMR** of **6a** (243 MHz, CD_2Cl_2).

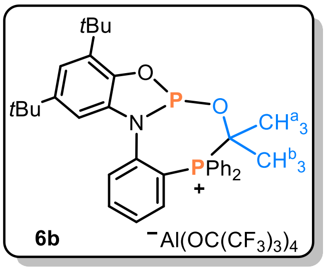

2b (50 mg, 33.8 µmol, 1.0 equiv) was dissolved in CH_2Cl_2 (2 mL), followed by the addition of acetone (7.9 mg, 135.3 µmol, 4.0 equiv). A colorless solution was obtained. The volatiles were removed under reduced pressure and washed with pentane, then dried *in vacuo* to give **6b** as a white solid with containing small amount of unknown impurities (49.1 mg, 94% yield).

^{1}**H NMR** (600 MHz, CD_2Cl_2): δ 8.01 – 7.90 (m, 3H), 7.76 (tdd, J = 7.6, 3.4, 1.1 Hz, 2H), 7.70 (ddt, J = 8.9, 7.9, 3.3 Hz, 2H), 7.62 (t, J = 9.8 Hz, 2H), 7.51 – 7.43 (m, 3H), 7.22 (ddd, J = 8.6, 7.4, 3.9 Hz, 2H), 6.86 (d, J = 1.9 Hz, 1H), 5.50 (d, J = 1.4 Hz, 1H), 2.18 (dd, J = 16.0, 3.7 Hz, 3H, OCC$\boldsymbol{H}^b_3$CH$_3$), 1.54 (d, J = 16.7 Hz, 3H, OCCH$_3$C$\boldsymbol{H}^a_3$), 1.47 (s, 9H), 1.02 (s, 9H).

^{13}**C{^{1}H} NMR** (151 MHz, CD_2Cl_2): δ 147.0 (s, 1C), 144.7 (d, J = 14.1 Hz, 1C), 142.0 (dd, J = 13.5, 6.6 Hz, 1C), 138.2 (d, J = 2.7 Hz, 1C), 136.5 (d, J = 6.9 Hz, 1C), 136.4 (d, J = 3.1 Hz, 1C), 136.2 (s, 1C), 135.4 (d, J = 3.0 Hz, 1C), 134.9 (d, J = 9.0 Hz, 2C), 134.8 (d, J = 8.8 Hz, 2C), 133.0 (s, 1C), 132.2 (dd, J = 8.9, 3.2 Hz, 1C), 131.2 (d, J = 11.8 Hz, 2C), 130.2 (d, J = 12.2 Hz, 2C), 129.9 (d, J = 11.1 Hz, 1C), 121.7 (q, J = 292.4 Hz , OC(***C***F$_3$)$_4$), 117.0 (s, 1C), 116.6 (s, 1C), 116.1 (d, J = 3.3 Hz, 1C), 115.6 (s, 1C), 104.1 (s, 1C), 75.0 (d, J = 50.1 Hz, 1C), 34.9 (s, 1C), 34.9 (s, 1C), 31.6 (s, 3C), 29.7 (s, 3C), 27.8 (dd, J = 7.3, 2.1 Hz, 1C), 26.8 (dd, J = 16.7, 8.4 Hz, 1C).

^{19}**F NMR** (376 MHz, CD_2Cl_2): δ -75.7.

^{31}**P{^{1}H} NMR** (243 MHz, CD_2Cl_2): δ 127.0 (d, J = 11.3 Hz, O***P***N), 34.6 (d, J = 11.3 Hz, ***P***Ph$_2$).

HRMS (ESI): $[C_{35}H_{40}NO_2P_2]^+$, calculated: 568.2529, found: 568.2530.

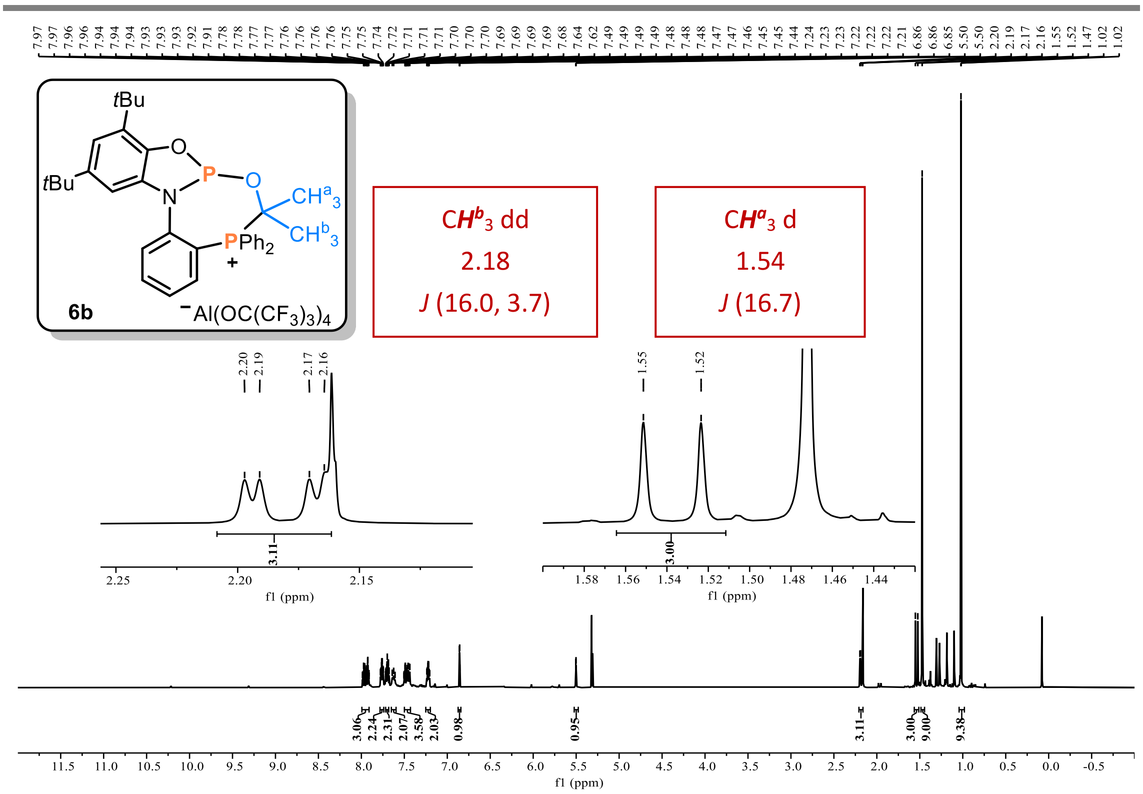

Figure S81: ^{1}**H NMR** of **6b** (600 MHz, CD_2Cl_2).

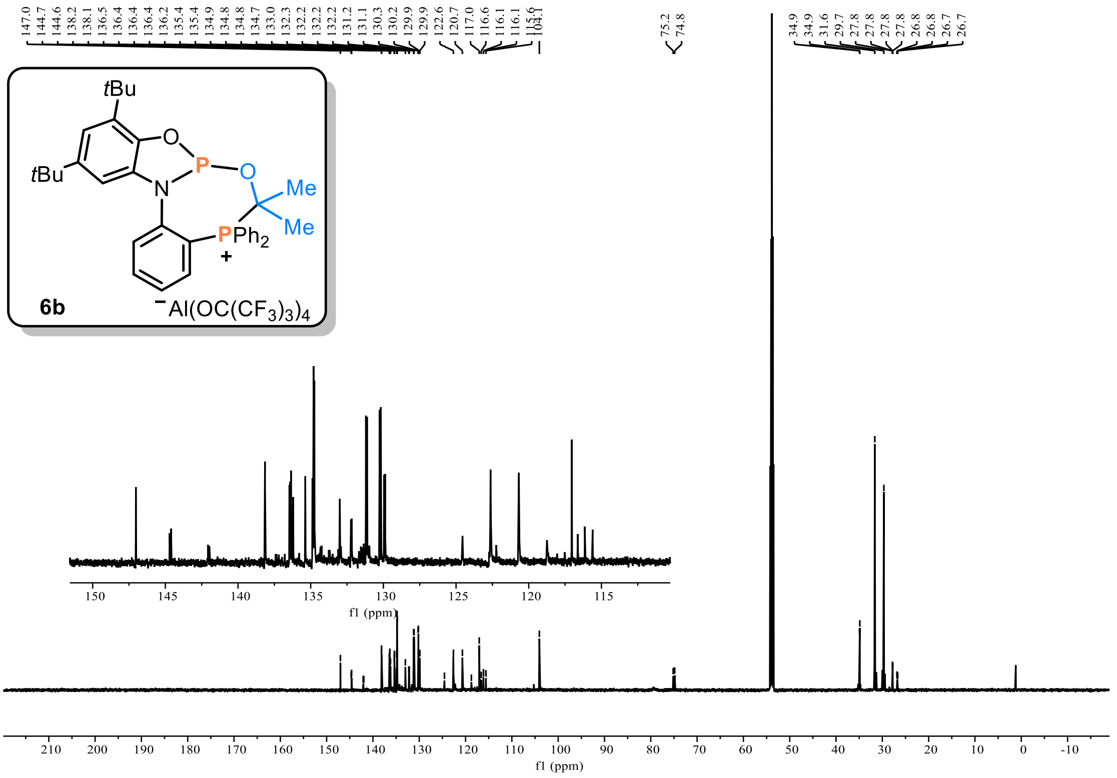

Figure S82: ^{13}**C{**^{1}**H} NMR** of **6b** (151 MHz, CD_2Cl_2).

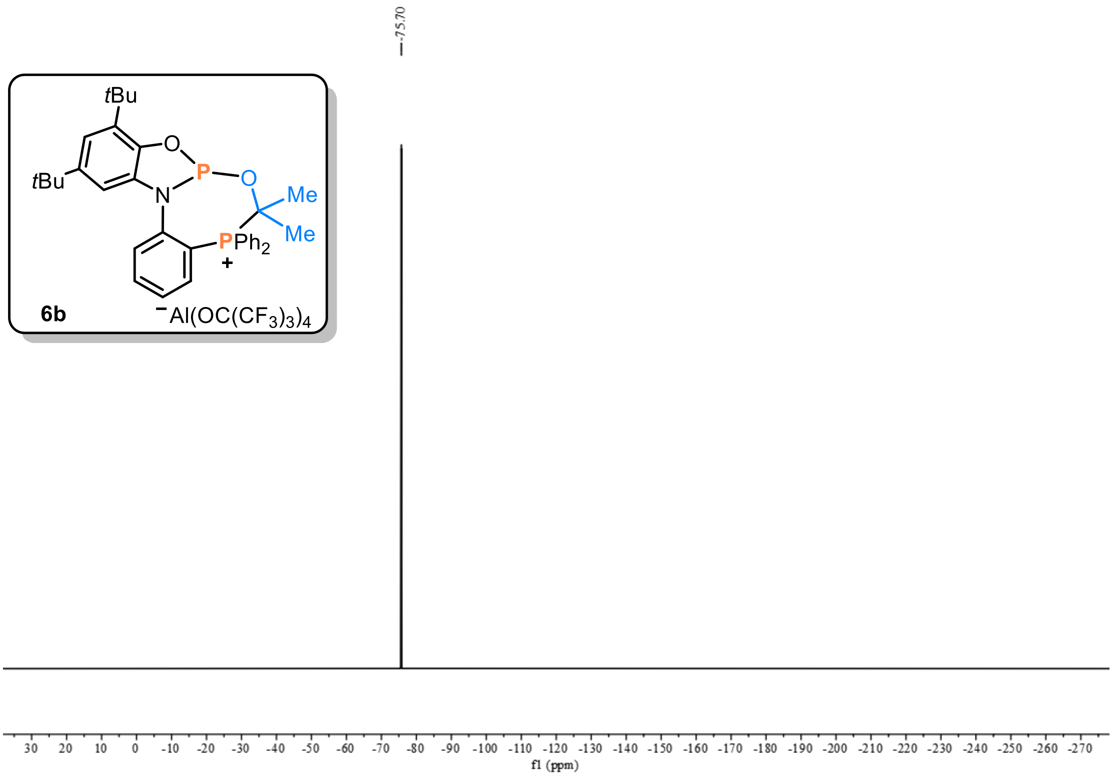

Figure S83: ^{19}**F NMR** of **6b** (376 MHz, CD_2Cl_2).

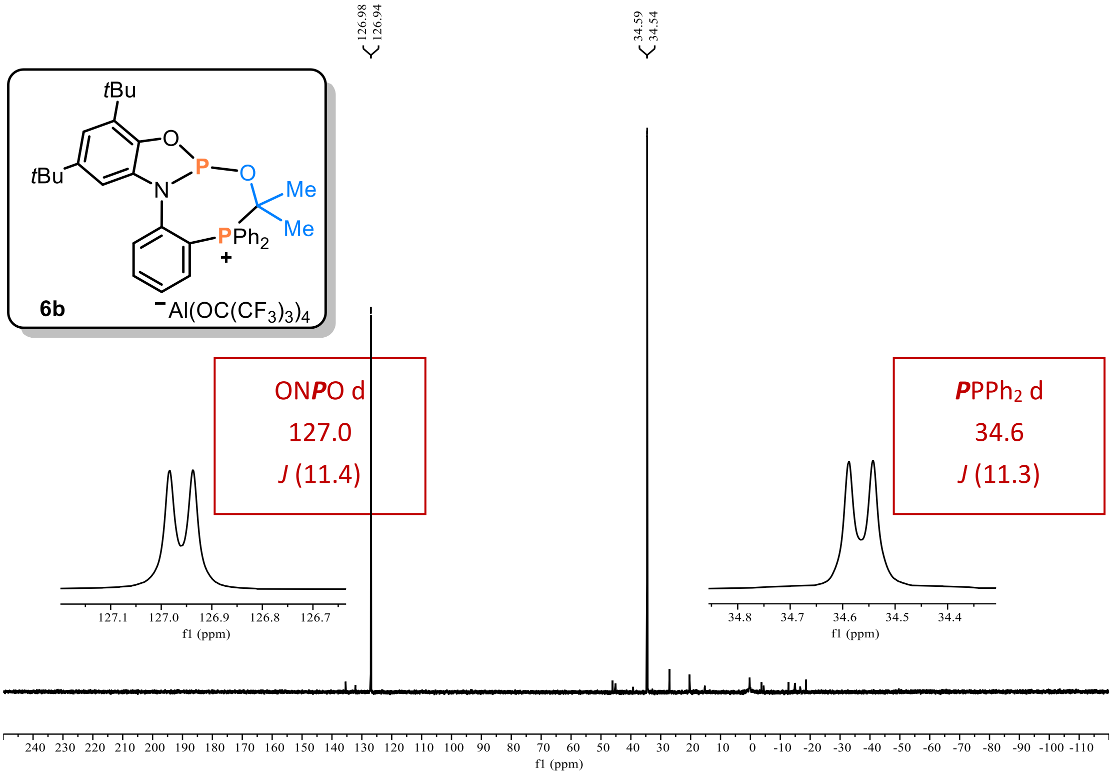

Figure S84: ^{31}**P{**^{1}**H} NMR** of **6b** (243 MHz, CD_2Cl_2).

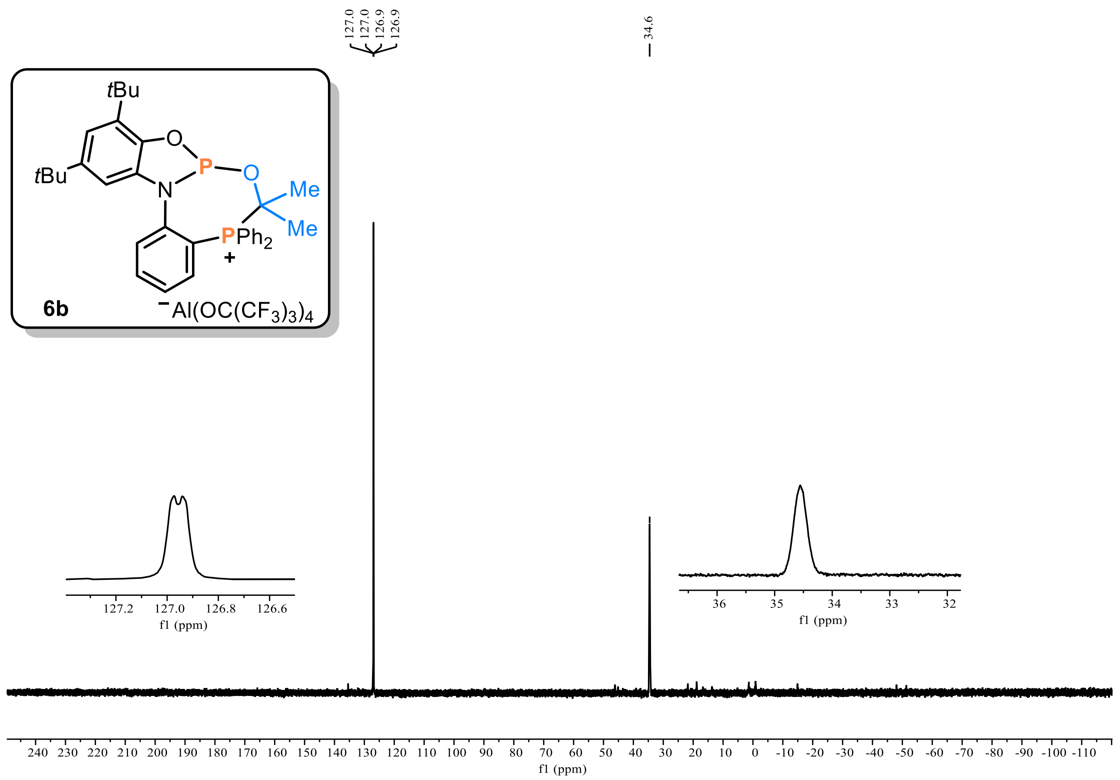

Figure S85: ^{31}**P NMR** of **6b** (243 MHz, CD_2Cl_2).

7. Coordination Chemistry

7.1 Steric profiles

Steric maps of crystalized compounds (**3a**,**3b**,**4a**,**4b**,**4c**,**5**,**6a**) are reported below, which were obtained using the SambVca 2.1 web application with a sphere radius of 3.5Å.[4]

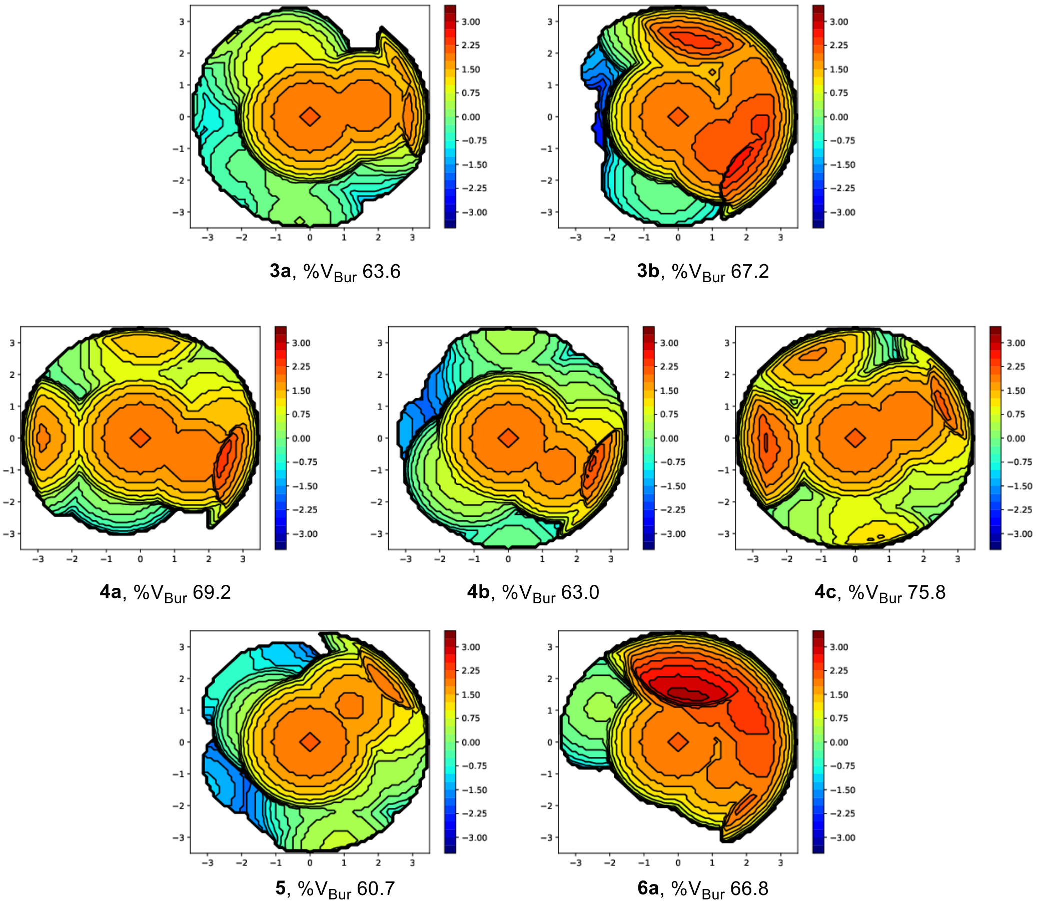

Figure S86: Steric maps of the crystalized compounds (**3a**,**3b**,**4a**,**4b**,**4c**,**5**,**6a**).

7.2 Reaction development

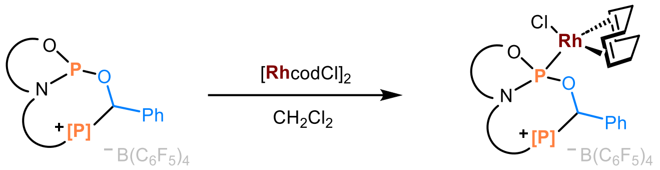

5 7

The colorless solid **5** (17 mg, 13.1 µmol, 1.0 equiv) and $[RhcodCl]_2$ (3.2 mg, 6.6 µmol, 0.5 equiv) were weighed into a J. Young type NMR tube. An orange solution was obtained after adding CD_2Cl_2 (0.5 mL), then probed by the multinuclear NMR data.

^{1}H NMR (600 MHz, CD_2Cl_2): δ 8.13 – 8.09 (m, 1H), 8.05 (dd, J = 8.1, 5.4 Hz, 1H), 7.88 (tdd, J = 7.6, 2.4, 1.3 Hz, 1H), 7.83 (ddd, J = 12.7, 8.4, 1.3 Hz, 2H), 7.75 – 7.69 (m, 2H), 7.63 – 7.57 (m, 2H), 7.51 – 7.45 (m, 3H), 7.38 (tt, J = 7.5, 1.5 Hz, 1H), 7.19 – 7.12 (m, 2H), 7.08 – 7.00 (m, 3H), 6.66 (dd, J = 7.7, 2.0 Hz, 2H), 6.45 (d, J = 2.0 Hz, 1H), 5.79 – 5.68 (m, 3H), 4.26 (d, J = 19.6 Hz, 2H), 2.63 – 2.28 (m, 8H), 1.31 (s, 9H), 1.23 (s, 9H).

^{11}B NMR (128 MHz, CD_2Cl_2): δ -16.7.

^{13}C{^{1}H} NMR (151 MHz, CD_2Cl_2): 149.5 – 149.2 (m, $BArF_{20}$), 148.3, 147.8 – 147.6 (m, $BArF_{20}$), 143.6 (d, J = 12.6 Hz), 139.6 – 139.2 (m, $BArF_{20}$), 137.9 – 137.6 (m, $BArF_{20}$), 137.8 (d, J = 2.6 Hz), 137.6 – 137.3 (m, $BArF_{20}$), 137.5 (d, J = 8.5 Hz), 136.7 (d, J = 3.2 Hz), 136.6 (d, J = 10.1 Hz), 136.3 (d, J = 3.1 Hz), 136.0 – 135.7 (m, $BArF_{20}$), 135.5 (d, J = 2.7 Hz), 134.7 (dd, J = 7.7, 3.8 Hz), 133.4 (d, J = 9.1 Hz), 131.9 (d, J = 3.4 Hz), 131.3 (d, J = 11.8 Hz), 131.0 (d, J = 12.2 Hz), 130.5 (d, J = 13.2 Hz), 129.3 (d, J = 2.6 Hz), 129.1 (d, J = 5.5 Hz), 128.0 (d, J = 3.9 Hz), 120.8 (dd, J = 82.6, 1.6 Hz), 117.7 (dd, J = 16.4, 4.5 Hz), 117.2, 117.0 (dd, J = 16.7, 5.5 Hz), 116.5, 113.7, 113.1, 104.6 (d, J = 4.3 Hz), 79.0 (d, J = 14.0 Hz), 74.5 (dd, J = 58.7, 13.1 Hz), 73.9 (d, J = 12.8 Hz), 72.5 (d, J = 12.1 Hz), 35.4, 34.9, 34.0, 33.0, 31.6, 29.5, 29.1 (d, J = 1.5 Hz), 28.2 (d, J = 1.9 Hz).

^{19}F NMR (376 MHz, CD_2Cl_2): δ -133.1 (q, J = 9.8 Hz), -163.7 (t, J = 20.4 Hz), -165.07 – -171.96 (m).

^{31}P{^{1}H} NMR (243 MHz, CD_2Cl_2): δ 120.7 (d, $^{1}J_{RhP}$= 275.9 Hz, O***P***N), 27.2 (s, ***P***Ph$_2$).

HRMS (ESI): $[C_{47}H_{52}ClNO_2P_2Rh]^+$, calculated: 862.2211, found: 862.2231.

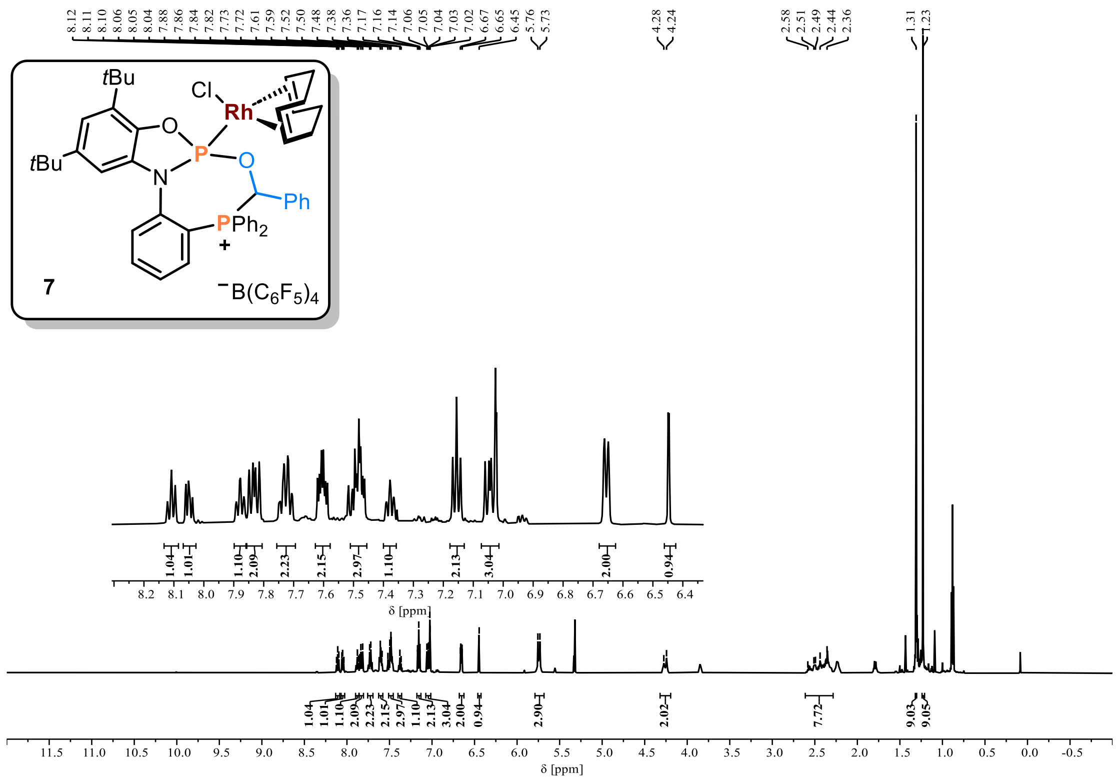

Figure S87: ^{1}**H NMR** of **7** (600 MHz, CD_2Cl_2).

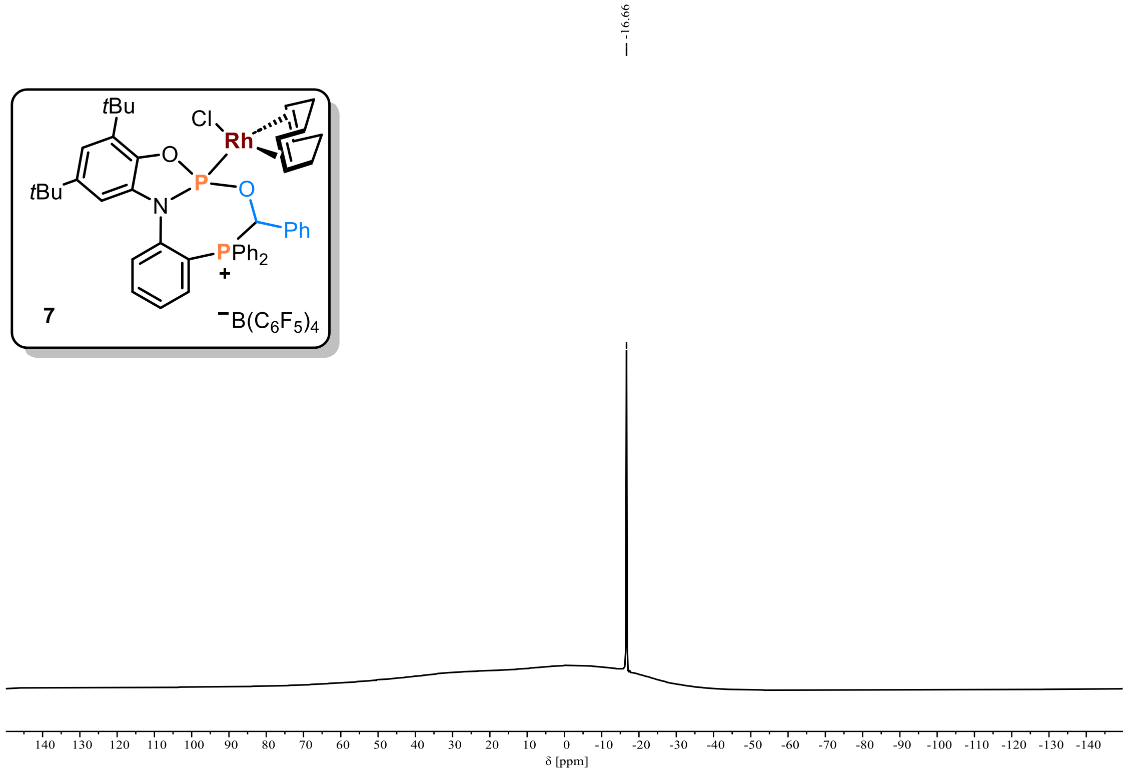

Figure S88: ^{11}**B NMR** of **7** (128 MHz, CD_2Cl_2).

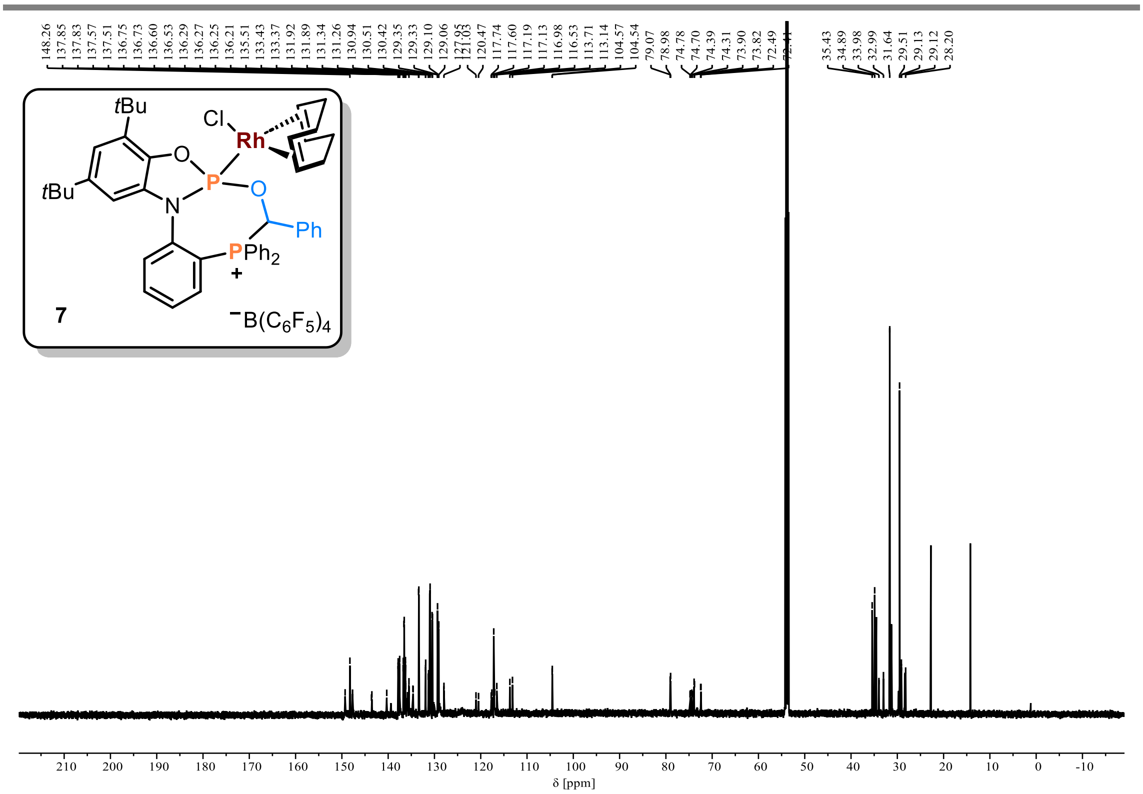

Figure S89: **^{13}C NMR** of **7** (151 MHz, CD_2Cl_2).

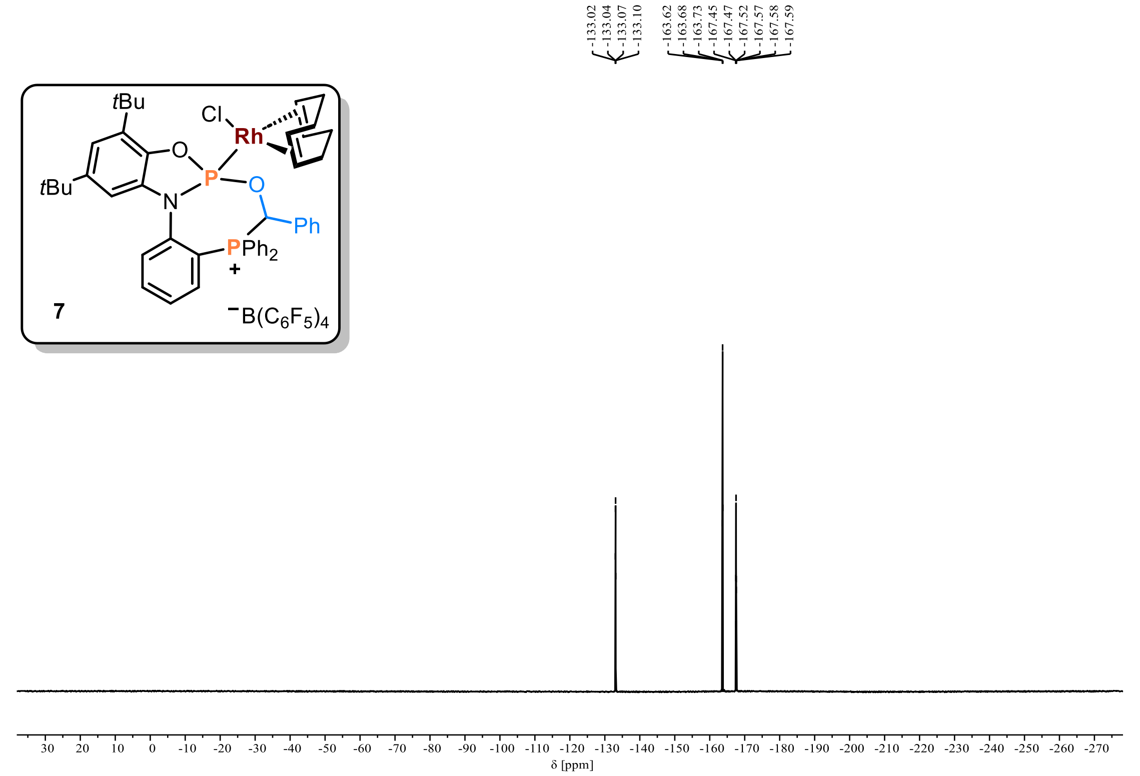

Figure S90: **^{19}F NMR** of **7** (376 MHz, CD_2Cl_2).

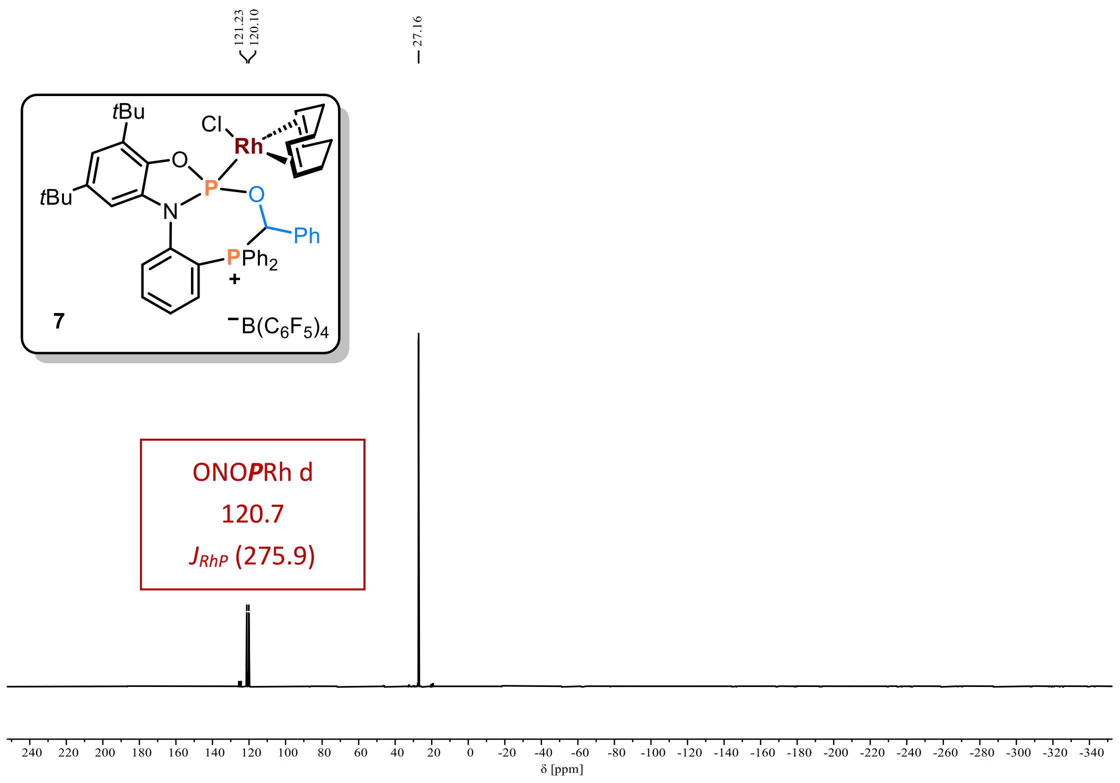

Figure S91: ^{31}**P{**^{1}**H} NMR** of **7** (243 MHz, CD_2Cl_2).

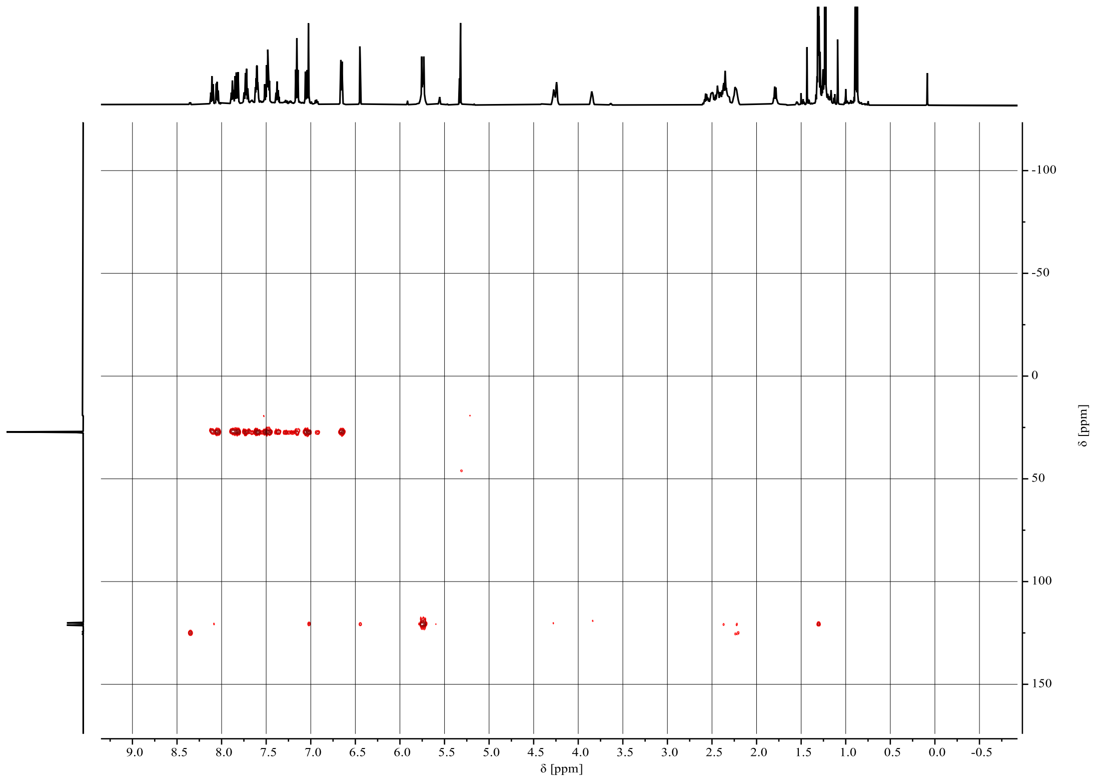

Figure S92: ^{1}**H-**^{31}**P HMBC NMR** of **7** (243MHz, CD_2Cl_2).

8. Optical Properties of Select Compounds

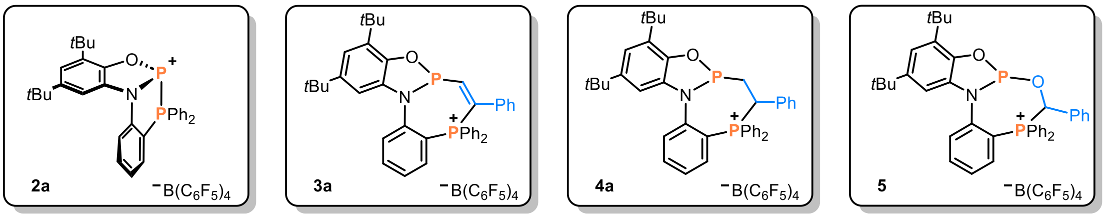

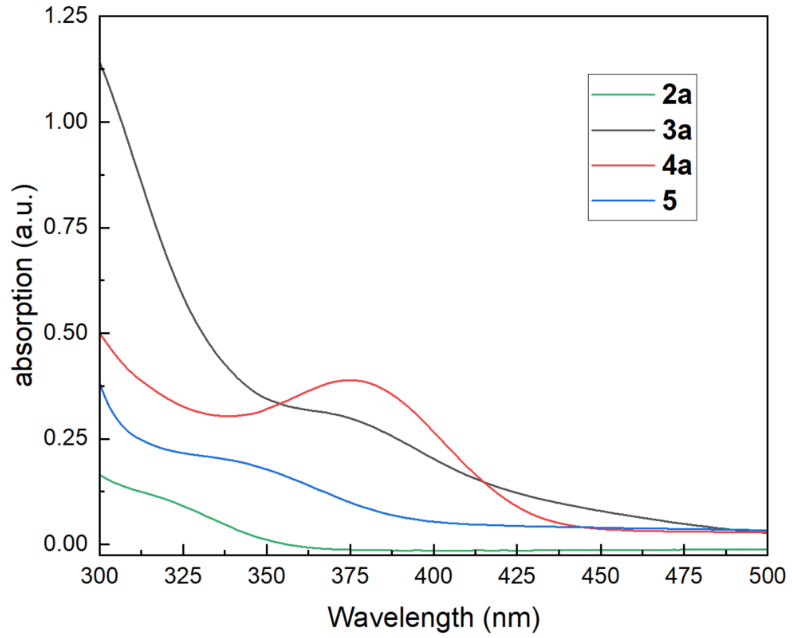

Figure S93: Absorption spectra of selected compounds (**2a**, **3a**, **4a**, and **5**) in CH_2Cl_2 (1.0×10^{-4} mol L^{-1}).

9. X-Ray Diffraction

Suitable crystals were taken directly from the mother liquor, immersed in a perfluorinated ether oil and fixed on a cryo loop. The diffraction data were collected from a shock-cooled single crystal at 100 K on a Bruker D8 VENTURE dual wavelength Mo/Cu three-circle diffractometer with a microfocus sealed X-ray tube using mirror optics as monochromator and a Bruker PHOTON III detector. The diffractometer was equipped with an Oxford Cryostream 800 low temperature device and used MoK_{α} radiation (λ = 0.71073 Å). All data were integrated with SAINT and a multi-scan absorption correction using SADABS was applied.[5] The structures were solved by direct methods using SHELXT and refined by full-matrix least-squares methods against F^2 by SHELXL-2018/3.[6,7] All non-hydrogen atoms were refined with anisotropic displacement parameters. The hydrogen atoms were refined isotropically on calculated positions using a riding model. Crystallographic data for the structures reported in this paper have been deposited with the Cambridge Crystallographic Data Centre.[8] CCDC 2357323-2357331 contain the supplementary crystallographic data for this paper. These data can be obtained free of charge from The Cambridge Crystallographic Data Centre via www.ccdc.cam.ac.uk/structures. This report and the CIF file were generated using FinalCif.[9]

9.1 Bond angles profile

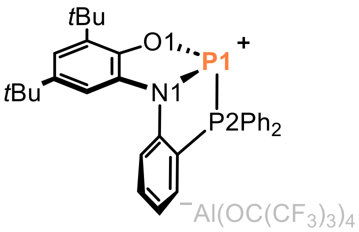

2b

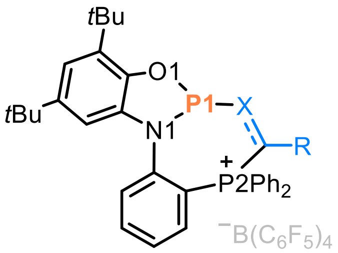

3a-b, **4a-4c**, X = C
5, **6a**, X = O

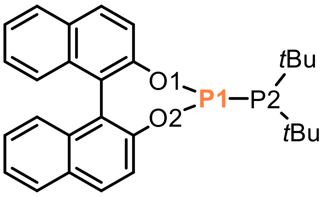

diphosphane

The bond angles around the central phosphorus (**P1**) are listed below.

Compounds	**∠O1P1N1**	**∠O1P1P2**	**∠N1P1P2**
**2b**	94.18(7)	104.53(5)	87.39(5)
	**∠O1P1N1**	**∠O1P1C**	**∠N1P1C**
**3a**	93.09(11)	103.61(13)	99.06(13)
**3b**	92.82(11)	101.54(11)	100.65(11)
**4a**	92.66(6)	101.11(7)	98.24(7)
**4b**	91.29(12)	104.21(13)	98.48(13)
**4c**	92.51(7)	101.54(7)	96.42(8)
	**∠O1P1N1**	**∠O1P1O2**	**∠N1P1O2**
**5**	90.22(6)	104.22(5)	100.76(5)
**6a**	92.17(5)	96.92(5)	99.81(5)
	**∠O1P1O2**	**∠O1P1P2**	**∠O2P1P2**
**Diphosphane**[10]	98.94(9)	98.78	101.26(6)

9.2 X-ray crystallography

The structure **1** (PNOPCl) contains two crystallographically independent molecules and selected bond distances (Å) and angles (deg): d(P1−O1) = 1.6292(13), d(P1−N1) = 1.6815(14), ∠O1−P1−N1 = 92.18(7) and d(P3−O2) = 1.6317(13), d(P3−N2) = 1.6859(14), ∠O2−P3−N2 = 92.10(7).

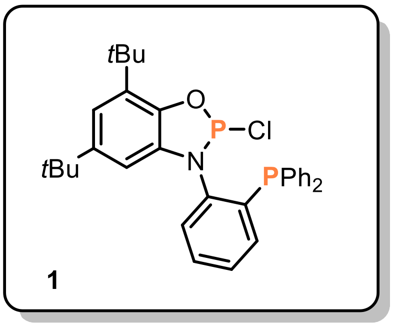

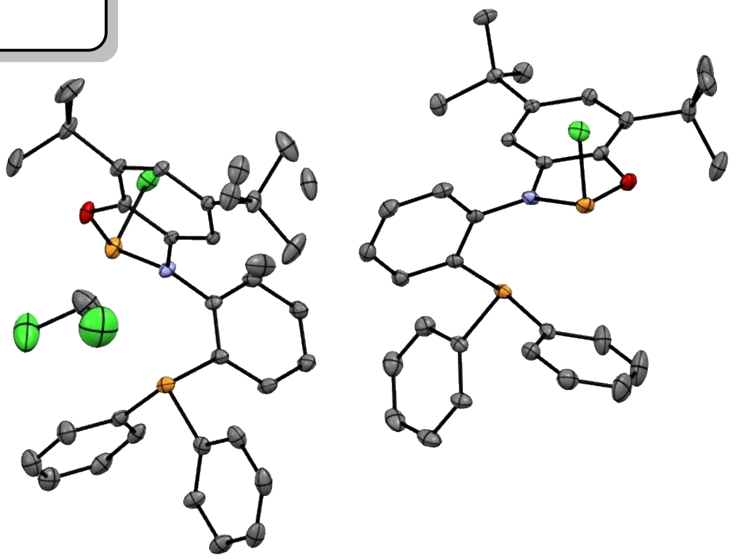

Figure S94: Thermal ellipsoid plot of **1** at 50 % probability. All carbon-based hydrogen atoms were omitted for clarity.

Table S1. Crystallographic data and structure refinement for compound **1**.

CCDC number	2357326
Empirical formula	$C_{32.50}H_{34}Cl_2NOP_2$
Formula weight	587.45
Temperature [K]	100(2)
Crystal system	triclinic
Space group (number)	$P\overline{1}$ (2)
a [Å]	12.944(5)
b [Å]	13.582(4)
c [Å]	19.223(7)
α [°]	103.948(13)
β [°]	103.841(14)
γ [°]	97.750(13)
Volume [Å^3]	3117.4(18)
Z	4
ρ_{calc} [gcm^{-3}]	1.252
μ [mm^{-1}]	0.337
F(000)	1232
Crystal size [mm^3]	0.376×0.343×0.210
Crystal colour	colourless
Crystal shape	block
Radiation	MoK_α (λ=0.71073 Å)
2θ range [°]	4.08 to 61.79 (0.69 Å)
Index ranges	$-18 \leq h \leq 18$<br>$-19 \leq k \leq 19$<br>$-27 \leq l \leq 27$
Reflections collected	151734
Independent reflections	19305<br>R_{int} = 0.0971<br>R_{sigma} = 0.0565
Completeness to<br>θ = 25.242°	99.9 %
Data / Restraints / Parameters	19305/154/738
Absorption correction<br>T_{min}/T_{max} (method)	0.6612/0.7461<br>(multi-scan)
Goodness-of-fit on F^2	1.089
Final *R* indexes<br>[$I \geq 2\sigma(I)$]	R_1 = 0.0565<br>wR_2 = 0.1423
Final *R* indexes<br>[all data]	R_1 = 0.0684<br>wR_2 = 0.1546
Largest peak/hole [eÅ^{-3}]	1.36/−1.43

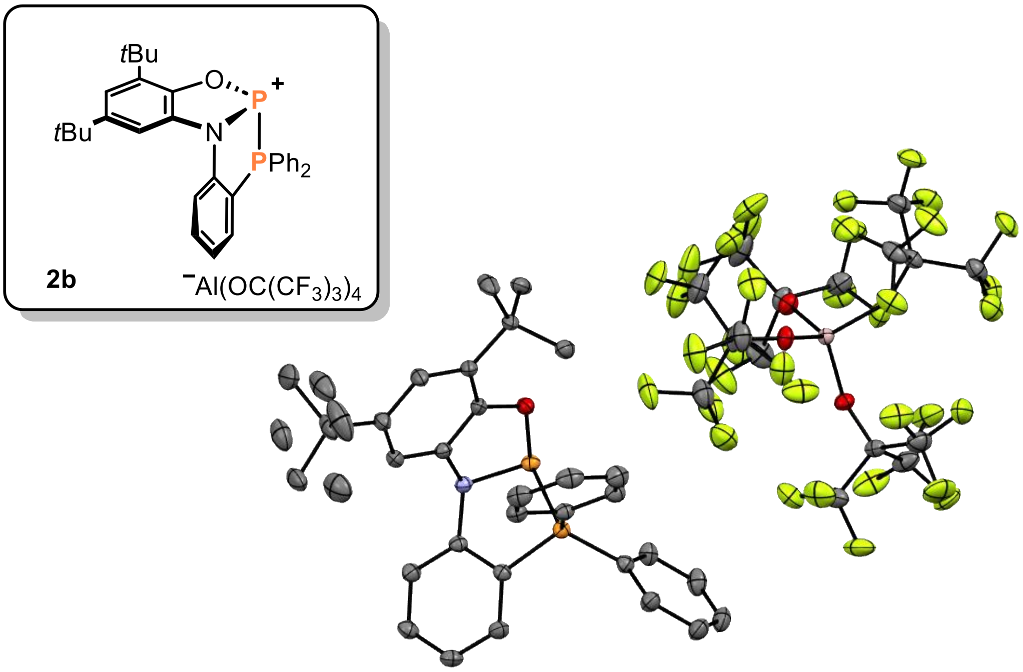

Figure S95: Thermal ellipsoid plot of **2b** at 50 % probability. All carbon-based hydrogen atoms were omitted for clarity.

Table S2. Crystallographic data and structure refinement for compound **2b**.

CCDC number	2357328
Empirical formula	$C_{48}H_{34}AlF_{36}NO_5P_2$
Formula weight	1477.68
Temperature [K]	120(2)
Crystal system	orthorhombic
Space group (number)	$Pbca$ (61)
a [Å]	20.836(13)
b [Å]	18.713(9)
c [Å]	29.740(14)
α [°]	90
β [°]	90
γ [°]	90
Volume [Å^3]	11595(10)
Z	8
ρ_{calc} [gcm^{-3}]	1.693
μ [mm^{-1}]	0.252
F(000)	5888
Crystal size [mm^3]	0.209×0.200×0.160
Crystal colour	yellow
Crystal shape	block
Radiation	MoK_α (λ=0.71073 Å)
2θ range [°]	3.91 to 59.08 (0.72 Å)
Index ranges	−28 ≤ h ≤ 28<br>−25 ≤ k ≤ 25<br>−40 ≤ l ≤ 40
Reflections collected	262857
Independent reflections	15702<br>R_{int} = 0.0831<br>R_{sigma} = 0.0343
Completeness to<br>θ = 25.242°	99.8 %
Data / Restraints / Parameters	15702/2994/875
Absorption correction<br>T_{min}/T_{max} (method)	0.6129/0.7458<br>(multi-scan)
Goodness-of-fit on F^2	1.018
Final R indexes<br>[I≥2σ(I)]	R_1 = 0.0445<br>wR_2 = 0.1137
Final R indexes<br>[all data]	R_1 = 0.0659<br>wR_2 = 0.1326
Largest peak/hole [eÅ^{-3}]	0.59/−0.40

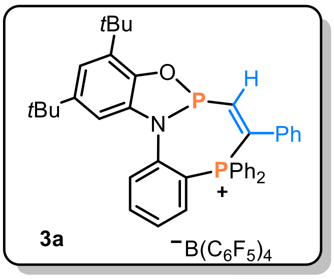

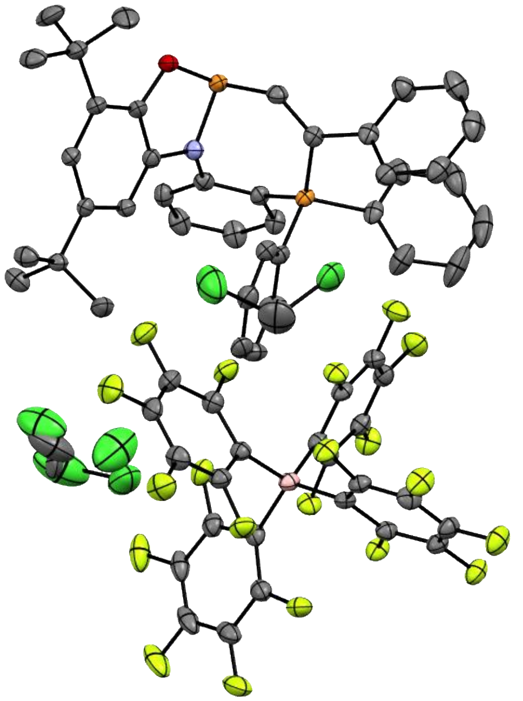

Figure S96: Thermal ellipsoid plot of **3a** at 50 % probability. All carbon-based hydrogen atoms were omitted for clarity.

Table S3. Crystallographic data and structure refinement for compound **3a**.

CCDC number	2357323
Empirical formula	$C_{66}H_{44}BCl_4F_{20}NOP_2$
Formula weight	1461.57
Temperature [K]	120(2)
Crystal system	triclinic
Space group (number)	$P\overline{1}$ (2)
a [Å]	13.370(6)
b [Å]	15.247(8)
c [Å]	16.300(6)
α [°]	87.59(2)
β [°]	75.11(2)
γ [°]	79.81(2)
Volume [Å^3]	3161(2)
Z	2
ρ_{calc} [gcm^{-3}]	1.536
μ [mm^{-1}]	0.342
F(000)	1476
Crystal size [mm^3]	0.40×0.24×0.22
Crystal colour	orange
Crystal shape	block
Radiation	MoK_α (λ=0.71073 Å)
2θ range [°]	3.74 to 52.05 (0.81 Å)
Index ranges	−15 ≤ h ≤ 16<br>−18 ≤ k ≤ 18<br>0 ≤ l ≤ 19
Reflections collected	12120
Independent reflections	12120<br>R_{int} = 0.1014<br>R_{sigma} = 0.0390
Completeness to θ = 25.242°	99.6 %
Data / Restraints / Parameters	12120/71/890
Absorption correction T_{min}/T_{max} (method)	0.593959/0.745263 (multi-scan)
Goodness-of-fit on F^2	1.023
Final *R* indexes [$I \geq 2\sigma(I)$]	R_1 = 0.0590<br>wR_2 = 0.1533
Final *R* indexes [all data]	R_1 = 0.0714<br>wR_2 = 0.1614
Largest peak/hole [eÅ^{-3}]	0.83/−0.70

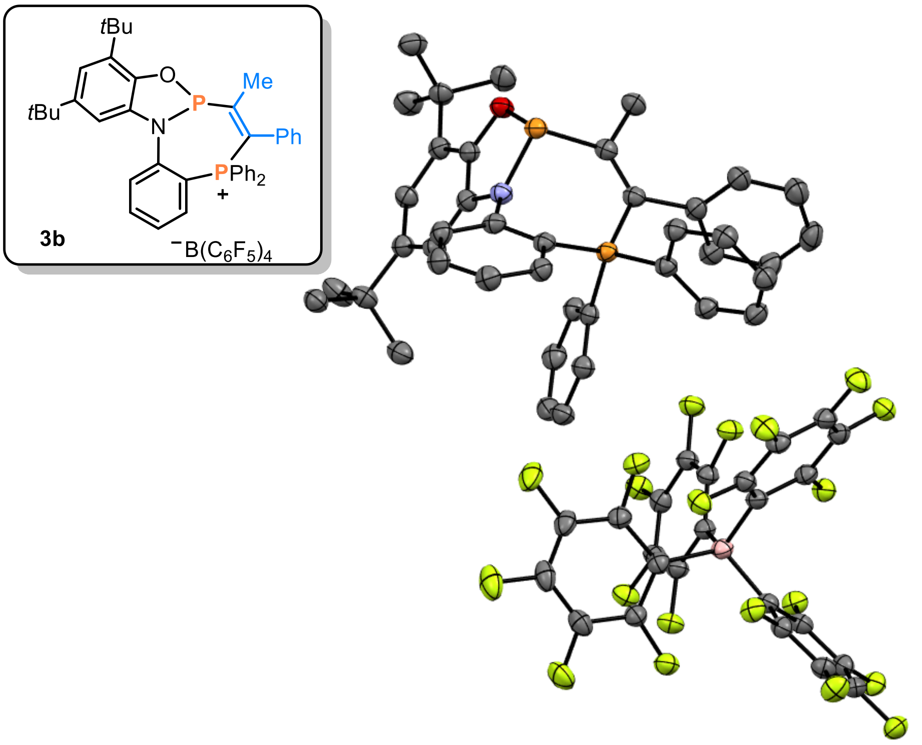

Figure S97: Thermal ellipsoid plot of **3b** at 50 % probability. All carbon-based hydrogen atoms were omitted for clarity.

Table S4. Crystallographic data and structure refinement for compound **3b**.

CCDC number	2357325
Empirical formula	$C_{65}H_{42}BF_{20}NOP_2$
Formula weight	1305.74
Temperature [K]	100(2)
Crystal system	triclinic
Space group (number)	$P\bar{1}$ (2)
a [Å]	13.124(8)
b [Å]	16.183(14)
c [Å]	17.493(13)
α [°]	110.67(3)
β [°]	95.58(2)
γ [°]	113.696(18)
Volume [Å^3]	3056(4)
Z	2
ρ_{calc} [gcm^{-3}]	1.419
μ [mm^{-1}]	0.176
F(000)	1324
Crystal size [mm^3]	0.147×0.139×0.076
Crystal colour	orange
Crystal shape	block
Radiation	MoK_α (λ=0.71073 Å)
2θ range [°]	3.75 to 53.87 (0.78 Å)
Index ranges	−16 ≤ h ≤ 16<br>−20 ≤ k ≤ 20<br>−22 ≤ l ≤ 22
Reflections collected	118125
Independent reflections	13150<br>R_{int} = 0.1178<br>R_{sigma} = 0.0718
Completeness to<br>θ = 25.242°	99.9 %
Data / Restraints / Parameters	13150/0/820
Absorption correction<br>T_{min}/T_{max} (method)	0.646/0.7454<br>(multi-scan)
Goodness-of-fit on F^2	1.015
Final *R* indexes<br>[I≥2σ(I)]	R_1 = 0.0512<br>wR_2 = 0.1253
Final *R* indexes<br>[all data]	R_1 = 0.0862<br>wR_2 = 0.1461
Largest peak/hole [eÅ^{-3}]	0.35/−0.35
Extinction coefficient	0.0152(9)

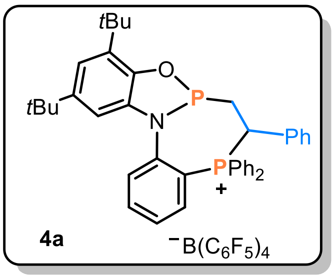

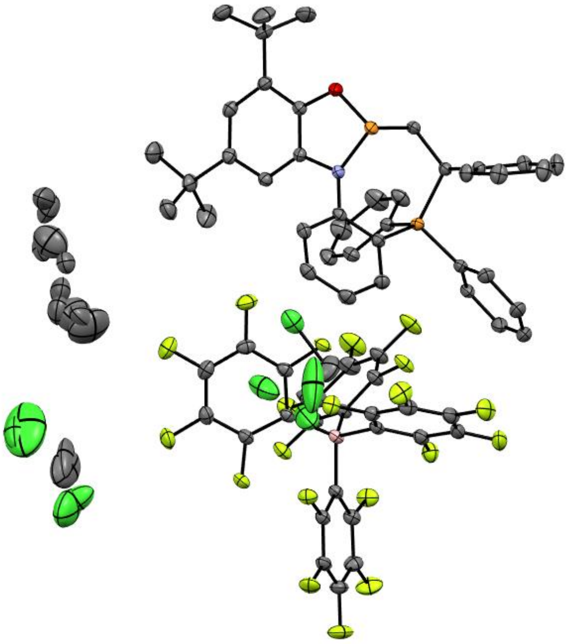

Figure S98: Thermal ellipsoid plot of **4a** at 50 % probability. All carbon-based hydrogen atoms were omitted for clarity.

Table S5. Crystallographic data and structure refinement for compound **4a**.

CCDC number	2357331
Empirical formula	$C_{71}H_{58}BCl_4F_{20}NOP_2$
Formula weight	1535.73
Temperature [K]	120(2)
Crystal system	triclinic
Space group (number)	$P\overline{1}$ (2)
a [Å]	13.127(4)
b [Å]	16.617(4)
c [Å]	17.814(6)
α [°]	114.495(14)
β [°]	98.862(18)
γ [°]	91.899(18)
Volume [Å^3]	3473.5(18)
Z	2
ρ_{calc} [gcm^{-3}]	1.468
μ [mm^{-1}]	0.315
F(000)	1564
Crystal size [mm^3]	0.343×0.329×0.319
Crystal colour	yellow
Crystal shape	block
Radiation	MoK_α (λ=0.71073 Å)
2θ range [°]	3.68 to 61.02 (0.70 Å)
Index ranges	−18 ≤ h ≤ 18<br>−23 ≤ k ≤ 23<br>−25 ≤ l ≤ 25
Reflections collected	216120
Independent reflections	20898<br>R_{int} = 0.0849<br>R_{sigma} = 0.0509
Completeness to<br>θ = 25.242°	99.8 %
Data / Restraints / Parameters	20898/291/1013
Absorption correction<br>T_{min}/T_{max} (method)	0.6779/0.7225<br>(multi-scan)
Goodness-of-fit on F^2	1.032
Final *R* indexes<br>[I≥2σ(I)]	R_1 = 0.0509<br>wR_2 = 0.1314
Final *R* indexes<br>[all data]	R_1 = 0.0624<br>wR_2 = 0.1416
Largest peak/hole [eÅ^{-3}]	0.65/−0.51

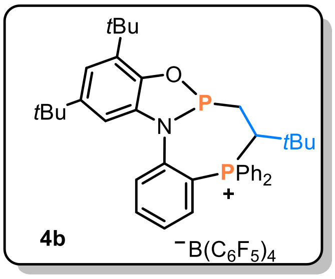

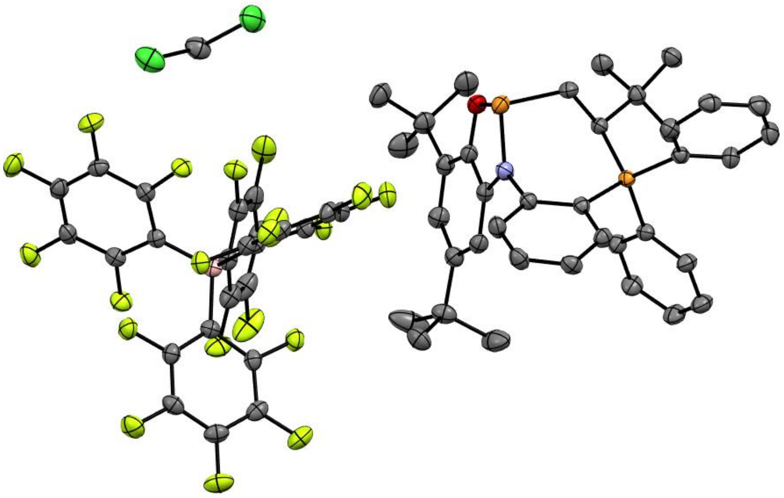

Figure S99: Thermal ellipsoid plot of **4b** at 50 % probability. All carbon-based hydrogen atoms were omitted for clarity.

Table S6. Crystallographic data and structure refinement for compound **4b**.

CCDC number	2357324
Empirical formula	$C_{63}H_{48}BCl_2F_{20}NOP_2$
Formula weight	1358.67
Temperature [K]	120(2)
Crystal system	monoclinic
Space group (number)	Cc (9)
a [Å]	10.1018(13)
b [Å]	31.870(6)
c [Å]	18.321(3)
α [°]	90
β [°]	92.637(15)
γ [°]	90
Volume [$Å^3$]	5892.2(16)
Z	4
ρ_{calc} [gcm^{-3}]	1.532
μ [mm^{-1}]	0.273
F(000)	2760
Crystal size [mm^3]	0.202×0.192×0.084
Crystal colour	yellow
Crystal shape	block
Radiation	MoK_α (λ=0.71073 Å)
2θ range [°]	4.23 to 66.49 (0.65 Å)
Index ranges	−15 ≤ h ≤ 15<br>−48 ≤ k ≤ 48<br>−28 ≤ l ≤ 26
Reflections collected	119817
Independent reflections	19847<br>R_{int} = 0.0988<br>R_{sigma} = 0.0719
Completeness to<br>θ = 25.242°	99.9 %
Data / Restraints / Parameters	19847/24/820
Absorption correction<br>T_{min}/T_{max} (method)	0.6951/0.7466<br>(multi-scan)
Goodness-of-fit on F^2	1.068
Final R indexes<br>[I≥2σ(I)]	R_1 = 0.0422<br>wR_2 = 0.0886
Final R indexes<br>[all data]	R_1 = 0.0812<br>wR_2 = 0.1055
Largest peak/hole [$eÅ^{-3}$]	0.55/−0.49
Flack X parameter	−0.01(3)

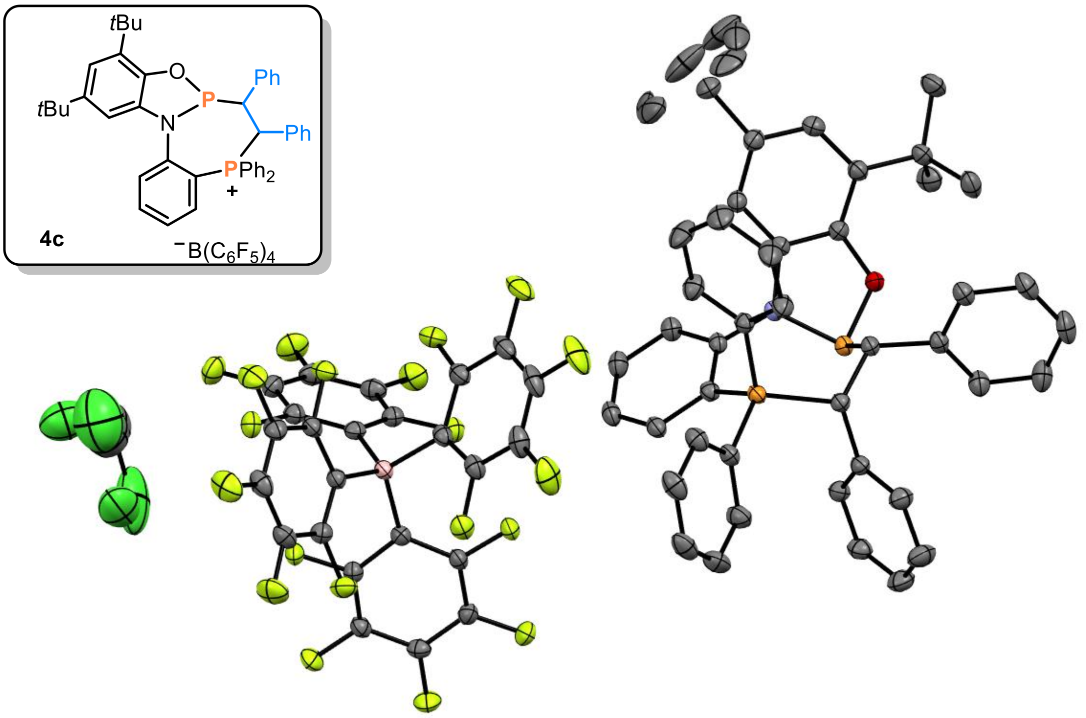

Figure S100: Thermal ellipsoid plot of **4c** at 50 % probability. All carbon-based hydrogen atoms were omitted for clarity.

Table S7. crystallographic data and structure refinement for compound **4c**.

CCDC number	2357327
Empirical formula	$C_{71}H_{48}BCl_2F_{20}NOP_2$
Formula weight	1454.75
Temperature [K]	120(2)
Crystal system	triclinic
Space group (number)	$P\overline{1}$ (2)
a [Å]	14.195(7)
b [Å]	15.574(7)
c [Å]	16.591(8)
α [°]	82.59(2)
β [°]	77.118(18)
γ [°]	64.527(16)
Volume [$Å^3$]	3226(3)
Z	2
ρ_{calc} [gcm^{-3}]	1.498
μ [mm^{-1}]	0.255
F(000)	1476
Crystal size [mm^3]	0.168×0.105×0.063
Crystal colour	yellow
Crystal shape	block
Radiation	MoK_α (λ=0.71073 Å)
2θ range [°]	3.91 to 60.09 (0.71 Å)
Index ranges	−19 ≤ h ≤ 19<br>−21 ≤ k ≤ 21<br>−23 ≤ l ≤ 23
Reflections collected	196656
Independent reflections	18332<br>R_{int} = 0.0807<br>R_{sigma} = 0.0384
Completeness to<br>θ = 25.242°	99.9 %
Data / Restraints / Parameters	18332/179/948
Absorption correction<br>T_{min}/T_{max} (method)	0.7005/0.7438<br>(multi-scan)
Goodness-of-fit on F^2	1.056
Final *R* indexes<br>[$I \geq 2\sigma(I)$]	R_1 = 0.0494<br>wR_2 = 0.1215
Final *R* indexes<br>[all data]	R_1 = 0.0700<br>wR_2 = 0.1345
Largest peak/hole [$eÅ^{-3}$]	0.76/−1.09

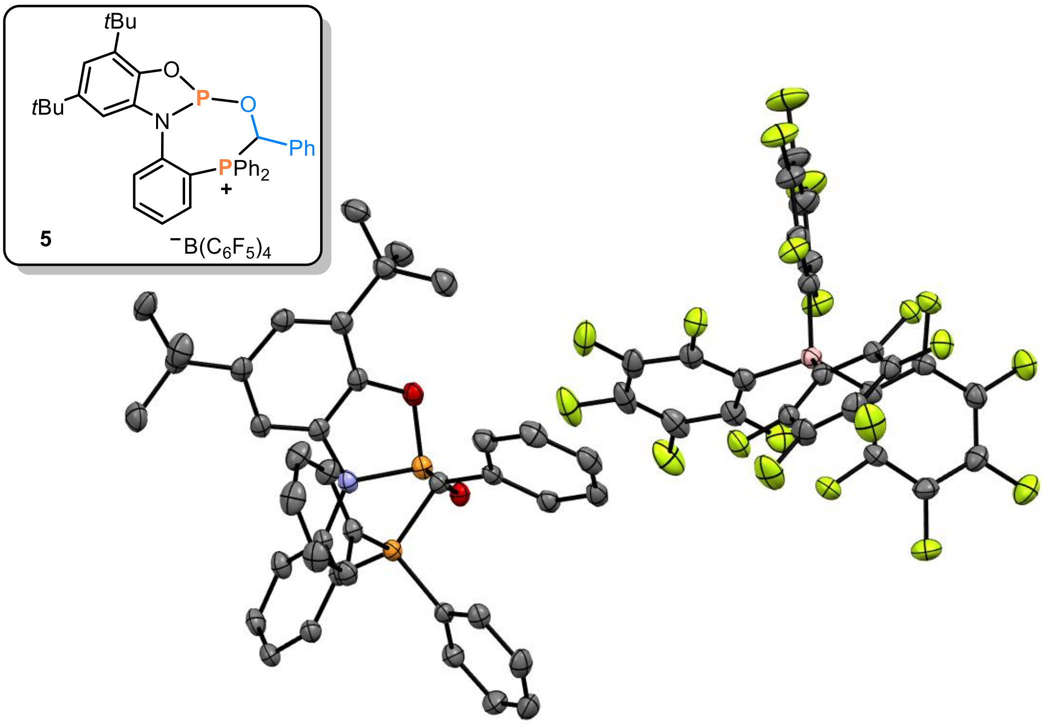

Figure S101: Thermal ellipsoid plot of **5** at 50 % probability. All carbon-based hydrogen atoms were omitted for clarity.

Table S8. Crystallographic data and structure refinement for compound **5**.

CCDC number	2357329
Empirical formula	$C_{63}H_{40}BF_{20}NO_2P_2$
Formula weight	1295.71
Temperature [K]	120(2)
Crystal system	monoclinic
Space group (number)	$P2_1/c$ (14)
a [Å]	11.884(3)
b [Å]	16.729(4)
c [Å]	31.882(7)
α [°]	90
β [°]	96.442(9)
γ [°]	90
Volume [Å^3]	6298(3)
Z	4
ρ_{calc} [gcm^{-3}]	1.366
μ [mm^{-1}]	0.171
F(000)	2624
Crystal size [mm^3]	0.178×0.144×0.129
Crystal colour	colourless
Crystal shape	block
Radiation	MoK_α (λ=0.71073 Å)
2θ range [°]	4.06 to 53.69 (0.79 Å)
Index ranges	−15 ≤ h ≤ 15<br>−21 ≤ k ≤ 21<br>−40 ≤ l ≤ 40
Reflections collected	284303
Independent reflections	13459<br>R_{int} = 0.0602<br>R_{sigma} = 0.0259
Completeness to<br>θ = 25.242°	99.9 %
Data / Restraints / Parameters	13459/2148/809
Absorption correction<br>T_{min}/T_{max} (method)	0.7072/0.7433<br>(multi-scan)
Goodness-of-fit on F^2	1.032
Final *R* indexes<br>[I≥2σ(I)]	R_1 = 0.0384<br>wR_2 = 0.1020
Final *R* indexes<br>[all data]	R_1 = 0.0432<br>wR_2 = 0.1066
Largest peak/hole [eÅ^{-3}]	0.74/−0.54

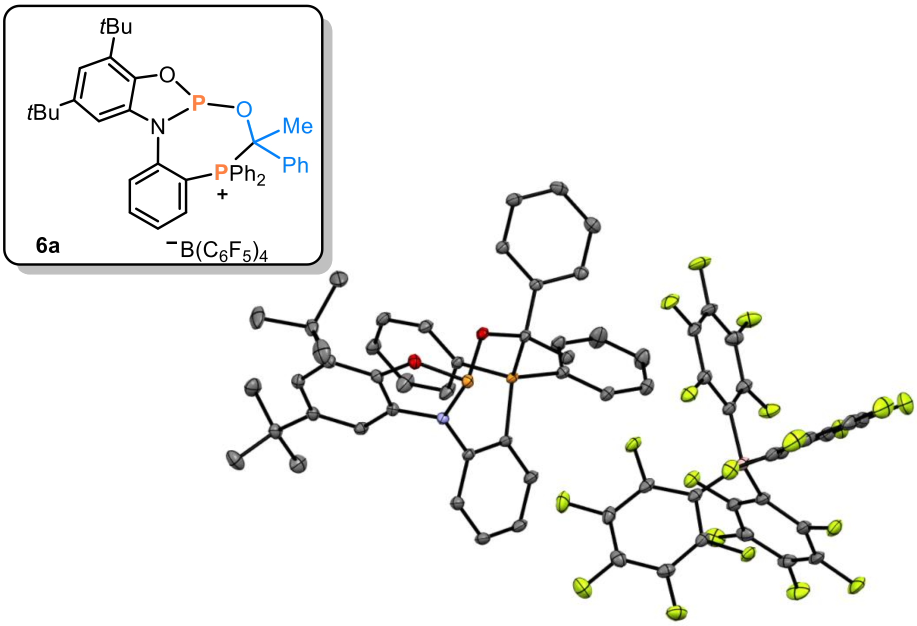

Figure S102: Thermal ellipsoid plot of **6a** at 50 % probability. All carbon-based hydrogen atoms were omitted for clarity.

Table S9. Crystallographic data and structure refinement for compound **6a**.

CCDC number	2357330
Empirical formula	$C_{64}H_{42}BF_{20}NO_2P_2$
Formula weight	1309.73
Temperature [K]	100(2)
Crystal system	monoclinic
Space group (number)	$C2/c$ (15)
a [Å]	50.046(15)
b [Å]	10.705(4)
c [Å]	27.527(8)
α [°]	90
β [°]	123.266(7)
γ [°]	90
Volume [Å^3]	12331(7)
Z	8
ρ_{calc} [gcm^{-3}]	1.411
μ [mm^{-1}]	0.175
F(000)	5312
Crystal size [mm^3]	0.352×0.342×0.194
Crystal colour	colourless
Crystal shape	block
Radiation	MoK_α (λ=0.71073 Å)
2θ range [°]	3.93 to 58.52 (0.73 Å)
Index ranges	$-68 \le h \le 68$<br>$-14 \le k \le 14$<br>$-37 \le l \le 37$
Reflections collected	418640
Independent reflections	16732<br>R_{int} = 0.0795<br>R_{sigma} = 0.0253
Completeness to<br>θ = 25.242°	99.9 %
Data / Restraints / Parameters	16732/0/818
Absorption correction<br>T_{min}/T_{max} (method)	0.6939/0.7458<br>(multi-scan)
Goodness-of-fit on F^2	1.037
Final *R* indexes<br>[$I \ge 2\sigma(I)$]	R_1 = 0.0394<br>wR_2 = 0.0989
Final *R* indexes<br>[all data]	R_1 = 0.0487<br>wR_2 = 0.1066
Largest peak/hole [eÅ^{-3}]	0.50/−0.41

10. Computational Details

General Information

All structure optimizations and single point energies were calculated using the Orca 5.0.4 program package.[11,12] For the Coulomb Integral, the RI approximation (RIJCOSX) was applied along with the corresponding auxiliary basis sets.[13-15] Computed molecular structures were visualized with CYLview20.[16]

Reaction Profiles

The composite method r^2-SCAN-3c with the implicit solvation accounted for by the CPCM model with dichloromethane as the solvent was applied for structure optimizations.[17] Frequency calculations were carried out in all cases to ensure the correct nature of the stationary points, with zero imaginary frequencies for ground states and one imaginary frequency corresponding to transition states. Thermochemistry data to obtain Gibbs free energies under standard conditions (298.15 K and 1 atm) were obtained using the rigid-rotor harmonic oscillator (RRHO) approximation and the Quasi-RRHO approach by Grimme for low frequencies as implemented in the default settings of Orca 5.0.4..[18] Additional corrections by +1.89 kcal·mol^{-1} were made to account for the higher reference solution concentration of 1 mol·L^{-1} typically used in solution. The conformational space was searched with the conformer-rotamer ensemble sampling tool (Crest) for the xtb program package and the lowest-energy conformer reoptimized as described above.[19,20] For transition state structures, IRC calculations where conducted when necessary to ensure that they connect to the correct minimum structures. Single point energies were refined at the PW6B95-D4/def2-QZVPP level of theory applying the SMD solvent model using dichloromethane as solvent.[21-23] Natural population analyses were done at the same level of theory using the NBO6 program interfaced with Orca 5.0.4.[24]

10.1 Free energy reaction profiles

10.1.1. Reaction with diphenylacetylene

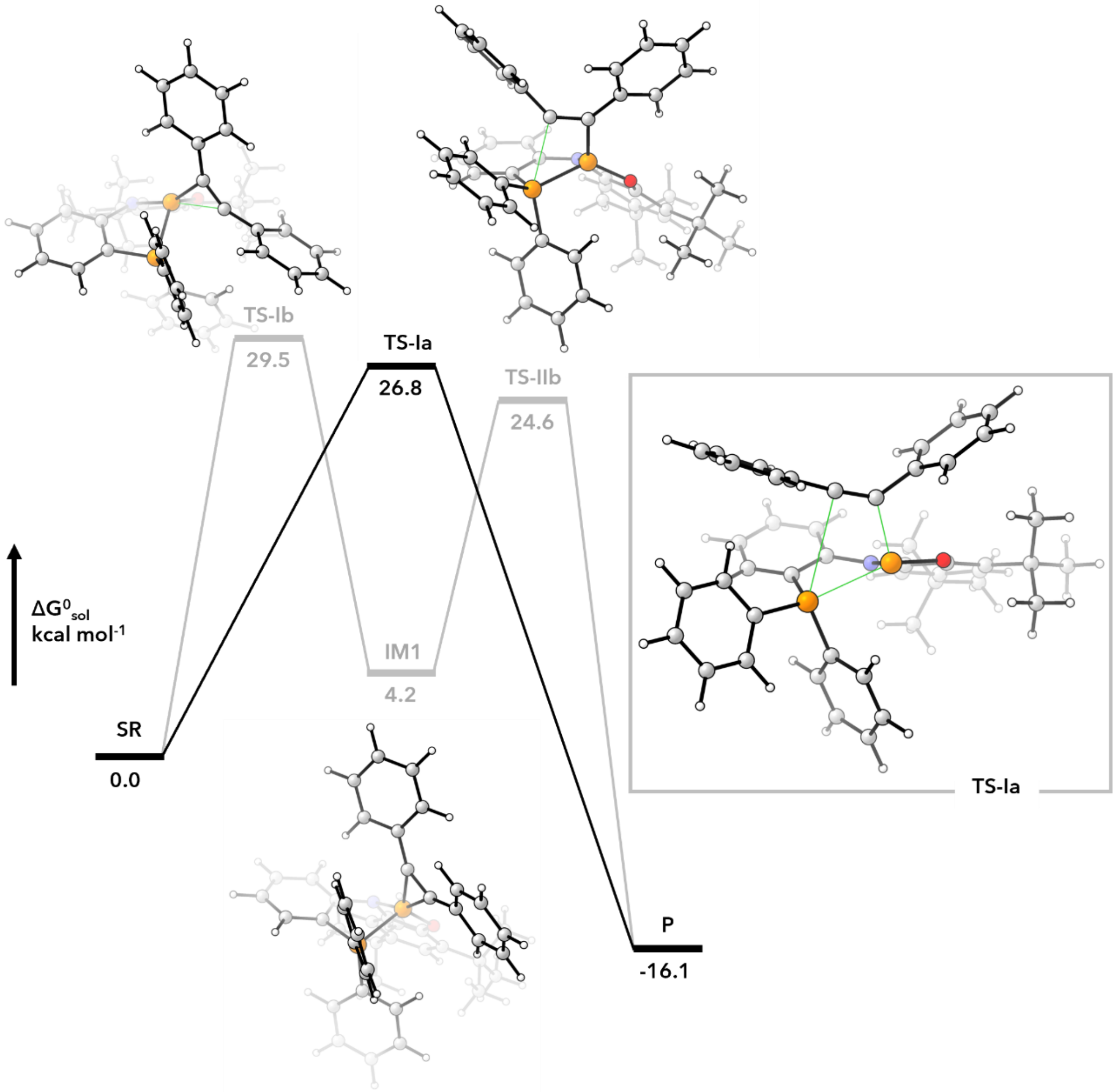

Figure S103: Computed free energy profile for the addition of diphenylacetylene to the phosphenium cation **2a** (SR = separated reactants)**.** The path proceeding through the intermediate formation of a phosphirane intermediate is shown in grey.

10.1.2. Reaction with styrene

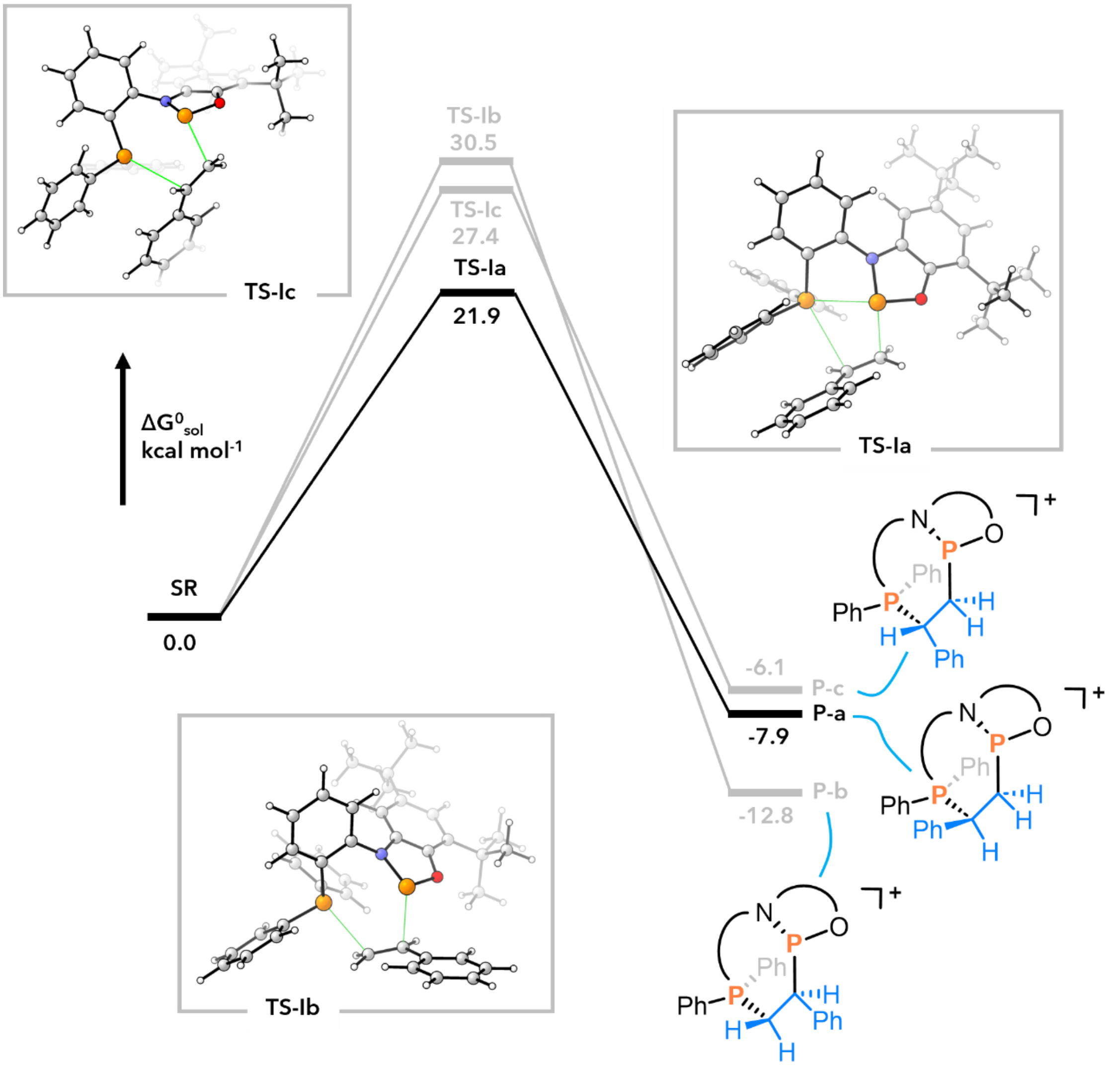

Figure S104: Computed free energy profile for the addition of styrene to the phosphenium cation **2a** (SR = separated reactants)**.** The Product P-a was also experimentally identified by SCXRD.

10.1.3. Reaction with benzaldehyde

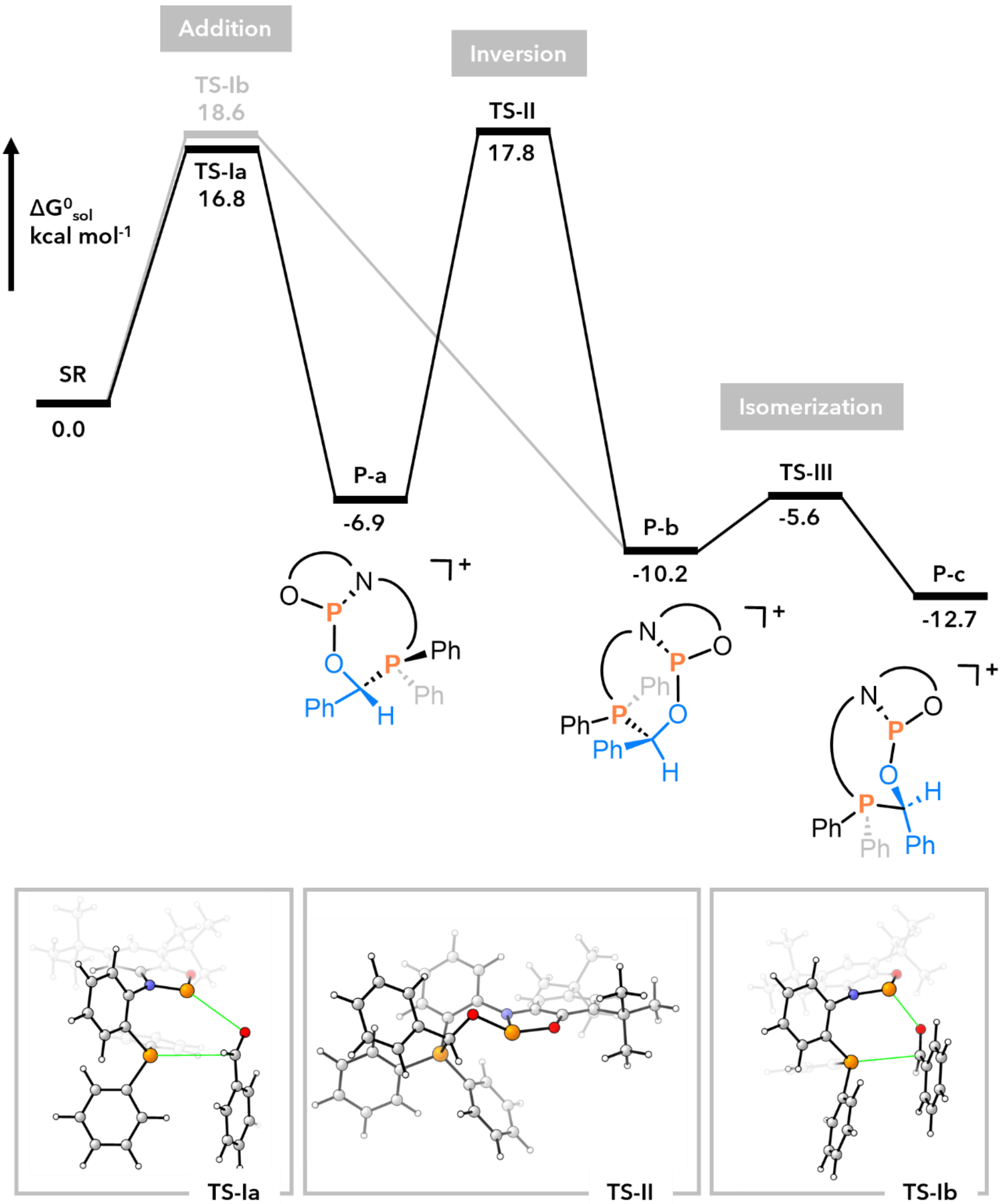

Figure S105: Computed free energy profile for the addition of benzaldehyde to the phosphenium cation **2a** (SR = separated reactants). The grey path shows the direct formation of the isomer P-b, which is also accessible by P-edge inversion of P-a. Quick isomerization finally yields the most stable conformer P-c, whose structure was confirmed by SCXRD.

10.2 xyz coordinates

Diphenylacetylene

E[a.u.] (r^2-SCAN-3c-CPCM(CH_2Cl_2): -539.3293468

Gibbs free energy correction (kcal mol^{-1}): 126.41

E[a.u.] (PW6B95-D4/def2-QZVPP-SMD(CH_2Cl_2)): -540.44628

C	-4.09268950333864	0.61400307262467	0.17028432087092
C	-3.12819157906533	1.61973487607761	0.11168915985341
C	-2.35120203538245	-1.05792716556168	0.21964180258075
C	-1.37097113259031	-0.04899623064458	0.16081720273885
C	-1.77773770048312	1.29779259230322	0.10689799741027
C	-3.69842298834044	-0.72270901379227	0.22380042277778
H	-4.44642544783722	-1.50924226936762	0.26926768464583
C	5.28132047903928	-1.67451842285117	0.08723621990764
H	-5.14799846025040	0.87103683779475	0.17392895652633
H	-3.43096239943958	2.66223023440047	0.06967996445635
H	-1.02396024636173	2.07808225991168	0.06164336696344
H	-2.04044885157962	-2.09756325914089	0.26146498594186
C	2.56057707443106	-1.00866876689509	0.12745547629314
C	4.31644440006502	-2.67931947127575	0.15493675859324
H	3.23113248232743	1.03937049250117	0.02329630968842
C	3.54119488218172	-0.00067033217044	0.05988207665040
H	2.21240436497810	-3.13550962180493	0.22767741790598
C	4.88792863438591	-0.33730707896176	0.04024491336384
H	5.63623206812493	0.44850692022135	-0.01216262368917
C	2.96647621000377	-2.35595801449733	0.17520905492068
H	4.61853803801827	-3.72219851704934	0.19201136001771
H	6.33624782947520	-1.93266388298888	0.07153323365083
C	0.00468280029799	-0.38413466507742	0.15625789985003
C	1.18535108134012	-0.67227057375580	0.14621603808145

Phenylacetylene

E[a.u.] (r^2-SCAN-3c-CPCM(CH_2Cl_2): -308.317334

Gibbs free energy correction (kcal mol^{-1}): 72.32

E[a.u.] (PW6B95-D4/def2-QZVPP-SMD(CH_2Cl_2)): -308.95787

C	-2.34683476676508	0.40524644302643	0.00363066270354
C	-3.45744254267482	-0.42888525730602	0.00075227778068
C	-3.29735290148897	-1.81384166511040	-0.00115163252167
C	-2.01686505053626	-2.36526854208992	-0.00016578908561
C	-0.89873140694828	-1.54133792939232	0.00270484007863
C	-1.05325002159324	-0.14434754411034	0.00465229899860
C	0.08827336370034	0.70374151838003	0.00764107461923

C	1.05986483936931	1.42319502893217	0.01049676704504
H	1.91812638731705	2.05588400009440	0.01310399559611
H	-2.46594424604175	1.48447620099246	0.00512157691068
H	-4.45365251238863	0.00418214975351	-0.00001405144373
H	-4.16878859498511	-2.46222261821667	-0.00340040652544
H	-1.88835724086495	-3.44393258717496	-0.00164525765426
H	0.10072469390038	-1.96557919777837	0.00348364349823

Styrene

E[a.u.] (r^2-SCAN-3c-CPCM(CH_2Cl_2): -309.5674339

Gibbs free energy correction (kcal mol^{-1}): 87.46

E[a.u.] (PW6B95-D4/def2-QZVPP-SMD(CH_2Cl_2)): -310.21135

H	5.38226055945223	0.40149853698804	-0.69125222886330
C	4.73594143698589	-0.39872597875993	-0.34071400010360
C	0.38915276539679	0.15100991738799	0.32518722432125
C	4.43426931050629	-2.68147708658597	0.36624287853493
C	3.38217832798745	-0.15478050805879	-0.15312622475328
H	6.33031869844658	-1.84842841554002	-0.23076635815222
H	4.83849098278657	-3.66914668898494	0.57064566710618
C	5.27028683687239	-1.66263466517316	-0.08237234457004
C	3.07720210254470	-2.43741765116716	0.55455791128806
H	2.42815111491711	-3.23675584171876	0.90551266749636
H	2.98819743857536	0.83609643326542	-0.36031694554886
C	1.08819128812066	-0.97340463325544	0.51562712729473
C	2.52584449938707	-1.17263323756290	0.29916007023802
H	0.84253793017468	1.07383856800895	-0.02686461138176
H	-0.67813088322014	0.17476223051643	0.52180966341505
H	0.55625759106631	-1.85660997935974	0.87098950367848

Benzaldehyde

E[a.u.] (r^2-SCAN-3c-CPCM(CH_2Cl_2): -345.5053871

Gibbs free energy correction (kcal mol^{-1}): 72.51

E[a.u.] (PW6B95-D4/def2-QZVPP-SMD(CH_2Cl_2)): -346.18817

H	5.55182150325894	0.45199990802741	-0.43930651175742
C	4.83682897752116	-0.32079362286404	-0.17207242342469
O	0.76633822947759	0.47425931234337	0.88450171367905
C	4.32426716811602	-2.66277806195476	0.17855626955343
C	3.53483945996028	0.02127776589503	0.15883328583648
H	6.25329909288419	-1.92462823580864	-0.42285135442182
H	4.63684716744587	-3.70280637665579	0.18433893492583
C	5.23165257099322	-1.66174028429012	-0.16247291104541
C	3.01758934498739	-2.32376572128344	0.51098702862629

H	2.29888417572088	-3.09531228274160	0.77901522574578
H	3.20931132269556	1.05761655722133	0.15705412572621
C	1.22949725721681	-0.65481007517618	0.85682639966983
C	2.61820037629207	-0.98234257032881	0.50216549773992
H	0.59538335342999	-1.53192631238372	1.10964471914651

Phosphenium ion 2a

E[a.u.] (r^2-SCAN-3c-CPCM(CH_2Cl_2): -2052.101666

Gibbs free energy correction (kcal mol^{-1}): 383.15

E[a.u.] (PW6B95-D4/def2-QZVPP-SMD(CH_2Cl_2)): -2055.576132

C	-3.30937728938862	-0.89423260783197	-0.11533851397494
C	-2.19221482331003	-1.52915912737892	0.42793990768086
H	3.24811886970107	-2.35403968872777	-1.18743527958990
C	-1.27763951849136	-0.75668404802678	1.12804569495094
H	-2.01723498066338	-2.59126782243696	0.30557495134342
C	-2.57243952914629	1.28939607606370	0.79305103319888
C	-2.78341923648866	2.78670768714032	1.01589007444881
C	-4.36376741185921	-1.66259357290720	-0.91556378480421
C	-3.47325416485377	0.48640975534871	0.08473116471436
H	-4.35202559445645	0.96101039768697	-0.33358629690574
N	-0.08288276356389	-1.21707382187846	1.75550830706352
C	-1.46266094917348	0.61018264077572	1.28973838753350
O	-0.42552360061693	1.20696444480675	2.02271623066834
C	1.69035278108865	-1.77131899430422	0.18651460150076
C	1.16099059346971	2.08428580816968	-1.57557435803080
P	0.83762122828980	0.17690572399614	2.24999756074055
P	1.90140576864828	0.02265521635587	0.11209121095013
C	-0.40433181006685	1.84974949427175	-3.39571142040183
C	1.02769977770276	0.71609427457828	-1.30330280458987
H	1.81589132368912	2.70742282772794	-0.97189016722532
C	4.41998031686853	0.33199288031205	1.21757000592367
C	3.65383165962689	0.44446962942816	0.04995633144494
H	3.66004485630209	0.92417946544778	-2.06119747800414
C	5.77813060259928	0.62189689597677	1.17572277314909
C	0.16814587688384	-0.08151057344534	-2.06683908945177
C	-0.54447565396832	0.49246474095981	-3.11312249071413
H	0.55413354900590	3.70060695185842	-2.84654475773826
C	0.44887084701960	2.64180065921635	-2.62962399450653
H	-0.96355438580744	2.29253843471749	-4.21478452899976
H	0.05619300886609	-1.13971121863291	-1.84838824772069
H	-1.20740540171470	-0.12488107701002	-3.71153711353431
C	4.24889835964933	0.84297335725027	-1.15265352964644
C	6.37207455256556	1.02383394164093	-0.01943340237871

H	-5.67999809348894	-1.93520520082729	0.80448038484941
C	5.60922883632933	1.13138547189760	-1.17945586572122
H	6.37268081923794	0.53789790418850	2.08033958427803
C	1.24704120911653	-4.48595116886153	0.55379319587208
C	2.23531299519456	-4.05365519653809	-0.32924623822364
C	2.46789186870719	-2.69424768660265	-0.51188700926735
H	2.83581767612462	-4.77887427907218	-0.86941110787117
C	0.69439460250380	-2.21476182555716	1.07716831287531
H	3.96544332055478	0.01730253772223	2.15310197312925
H	1.08092929629865	-5.54926819158299	0.69845047337956
C	0.47708866883468	-3.57130852023039	1.26952473387463
H	-0.28225793439608	-3.90127649443199	1.97164005022203
H	-4.09736211312438	3.12867859783717	-0.70370677315401
H	-4.96506747805036	2.77421227058019	0.81027764482333
H	-4.19407035698445	4.34958488282139	0.57346932553374
H	-3.07163191430029	-3.31049234730277	-1.55400618744037
H	-3.97745901872324	-3.64005004353821	-0.05775493649368
H	-4.80683885946728	-3.65191126335167	-1.62057957402550
C	-4.09103855102168	3.27664762647144	0.38179678558214
H	-1.91912076726334	2.81078766087003	3.03747491326000
H	-3.03725235702067	4.14039056145774	2.69118473187216
C	-2.85064657767891	3.07274302810162	2.52982074061514
H	-3.66805484218536	2.50799279374670	2.99146271776860
H	-1.78151082050084	4.64974703792628	0.54037711894909
C	-1.62342138680453	3.57701462627522	0.38122827123092
H	-0.65659298577979	3.30698565936790	0.81364637098329
H	-1.58025959978715	3.39133244467715	-0.69710735673896
C	-4.02562771853012	-3.15288881061538	-1.03809174933622
H	-4.74381718015503	-0.01393952403507	-2.31545413215931
H	-3.49314397801489	-1.15318598190732	-2.85039054059346
C	-4.45646168902741	-1.06987015111304	-2.33474610048028
H	-5.20791779018125	-1.61492455718046	-2.91735325007766
H	-6.05561001637197	-0.48897749757764	-0.15226575105199
C	-5.72924180711371	-1.53185130943514	-0.21310192859723
H	-6.48893211234183	-2.09191599327626	-0.77013556395152
H	6.07232415805809	1.43939101449521	-2.11208738116020
H	7.43335488194623	1.25357864045150	-0.04589701784933

Phosphenium ion 2a + Diphenylacetylene TS-Ia

E[a.u.] (r^2-SCAN-3c-CPCM(CH_2Cl_2): -2591.423254

Gibbs free energy correction (kcal mol^{-1}): 510.5

E[a.u.] (PW6B95-D4/def2-QZVPP-SMD(CH_2Cl_2)): -2596.001348

C	-4.38166350760615	0.43561368961320	-0.38505032962151

C	-4.91244315996142	-0.82592739530052	-0.10405753101026
H	-5.51399803799145	-3.98189477800247	-4.44535365993947
C	-4.06343144893214	-1.80961892755774	0.38387209607119
H	-6.37583584567817	-1.80789534487674	1.83479962753707
C	-2.11843924113075	-0.32308724718676	0.25606947272594
C	-0.61632317561548	-0.08038093422503	0.39694664846609
C	-5.32075177636717	1.52442266115811	-0.91275850318230
C	-3.01201026276838	0.66083875013424	-0.18845113937560
H	-5.95995018708936	-1.04169810978602	-0.27683597353209
N	-4.36722808500495	-3.14682894346983	0.68901687793615
C	-2.69736542239722	-1.55702636838865	0.53739158809868
O	-2.00452704483473	-2.68790255663882	0.90962850776888
C	-6.03124534855224	-4.92989364426140	0.50738532594081
C	-5.15835118425790	-4.86210802900521	-2.51829685043467
P	-2.92941649357091	-4.08151517377920	1.02337711351173
P	-4.63902815206715	-5.99823162488090	0.02067109948843
C	-3.47404560596552	-4.64219362870402	-4.23425462562552
C	-4.21904687143632	-5.46444571939507	-1.67684448710437
H	-1.51481002467926	-5.38951437122662	-3.73750447755405
C	-5.09952978676931	-8.42353642287508	-1.32217253522241
C	-5.28978316875006	-7.69563274670158	-0.14134385699503
H	-6.77571910540268	-10.10948088143883	1.73833518729050
C	-5.51735147857382	-9.74928348424993	-1.39723079422545
C	-2.90311961286522	-5.64603115483003	-2.11727955415205
C	-2.53582315712560	-5.24233062760714	-3.39759853036864
H	-3.18457527357745	-4.31914337496863	-5.23028445265255
C	-4.78260545228011	-4.45164134655685	-3.79389422017611
H	-6.17948110903637	-4.71196037619192	-2.17766632721927
H	-6.03944004466909	-7.77261410092761	1.88689558149680
H	-2.16746483653810	-6.10645487965850	-1.46110556845988
C	-5.88565433145091	-8.32019618905190	0.96348316905496
C	-6.12027507733882	-10.36153330588263	-0.30146285653133
H	-5.16734721299513	0.85930126576673	-2.98656749297595
C	-6.30535116024581	-9.64188541600778	0.87763852396728
H	-4.63378723604735	-7.95769663843329	-2.18539085730106
C	-7.95379211594319	-3.25653110304711	1.65135127345271
C	-8.30689609023964	-4.53448494335265	1.23276827668965
C	-7.34194545991189	-5.37049276992630	0.67496479150065
H	-8.68786823203149	-2.61089854905603	2.12395609876753
C	-5.69428052775939	-3.61551421181554	0.88422358374142
H	-8.13958496596053	-6.79373017121863	4.52778866162594
H	-7.61106520814366	-6.37864238674908	0.37485533655168
C	-6.65138354096584	-2.79600299373551	1.48248059309958
H	-9.32429136479783	-4.89251539561037	1.35699790182678

H	-0.47957280716064	1.61924489496296	-0.98163743436169
H	-0.73712692813620	2.08361576842157	0.71774726307185
H	0.84230146002398	1.48700047815544	0.18671701718454
H	-4.15000603034184	3.25205983243288	-0.24881058323828
H	-3.80993784340667	2.74816638624534	-1.92133774511068
H	-5.31841793513943	3.58768036909456	-1.53487999058493
C	-0.23808660579463	1.36656890512209	0.05659874240863
H	-0.39229144963652	-1.38531058720417	2.15236864920339
H	0.89617293222153	-0.18460593032334	1.94913779544088
C	-0.18158211336100	-0.35723412522632	1.84854764929195
H	-0.70256664603580	0.31399506410438	2.53961224609617
H	1.22003382415936	-0.81326292193002	-0.49578977795525
C	0.14467018053688	-1.01123502737221	-0.56939837664781
H	-0.02401784237357	-2.06570773028444	-0.33819753556635
H	-0.16763402795422	-0.82823007418314	-1.60356726028086
C	-4.59650813128692	2.85181228901999	-1.16581059646064
H	-7.03353986464606	0.87073503736523	0.29629112577216
H	-6.01512400294081	2.09690354215188	1.07658779506453
C	-6.43833775207261	1.77257440365251	0.11928164971673
H	-7.11309858085516	2.55644227017090	-0.24320500171787
H	-6.53276862776856	0.13903806545145	-2.11112893423533
C	-5.94604990474054	1.05404443154055	-2.24077295415925
H	-6.61342398693687	1.82987058016742	-2.63303817651740
H	-6.44522193637481	-11.39604392865868	-0.36552143869699
H	-5.37221318251919	-10.30300686491055	-2.32044414924367
C	-3.23833814702066	-4.31176737307475	3.02346214380347
C	-4.01425323099107	-5.25456194977448	3.33106645975442
C	-2.53501086673806	-3.25754133539578	3.78024477910207
C	-4.86506437359636	-6.26290934124899	3.75156525217742
C	-6.56516564621790	-8.25822347218016	4.69894578527983
C	-6.24726444627799	-5.99623617929353	3.92733140016795
C	-4.35159622724087	-7.54870577849782	4.05988871504770
H	-4.81326495779833	-9.51326416903705	4.77113619201779
H	-6.63100789999998	-5.00867350830338	3.69184176770517
C	-7.08076623483499	-6.99485488533510	4.39586646548238
C	-5.20383598336219	-8.52839054503168	4.53408860068932
H	-3.29388778532741	-7.74531520755570	3.91774472455819
H	-4.19903116849932	-1.89285200426791	3.67116775967777
H	-7.22809703482502	-9.03659378146374	5.06513954335815
C	-1.23906431715098	-1.27089190218475	5.23084245813917
C	-1.22915756920649	-3.46717090013440	4.22662624868627
H	-0.72741813747335	-4.40590454095591	4.01027251830831
C	-3.18631775315964	-2.04753893047457	4.03344735752751
H	0.42220780870179	-2.63967830916326	5.31929693103717

C	-0.58914237614465	-2.47243409830293	4.95991093086758
C	-2.53405089930271	-1.05920021279992	4.76358083236845
H	-3.04245036121719	-0.12122008641979	4.96769967259128
H	-0.73382814902283	-0.49653685501328	5.80084011821982
H	-2.61281501542738	1.63971713784836	-0.41289034799193

Phosphenium ion 2a + Diphenylacetylene TS-Ib

E[a.u.] (r^2-SCAN-3c-CPCM(CH_2Cl_2): -2591.412016

Gibbs free energy correction (kcal mol^{-1}): 510.37

E[a.u.] (PW6B95-D4/def2-QZVPP-SMD(CH_2Cl_2)): -2595.997763

C	-4.61170154294540	0.26715017702895	-0.17209584268343
C	-3.58810236751481	1.18125929326642	-0.43351077722896
H	0.51797364328639	-2.51076271715642	3.90627372496316
C	-2.31871907842723	0.68860047238950	-0.70490855833543
H	-2.34558871319484	3.76786900977714	-1.59000799583013
C	-3.04335354950202	-1.62913116238971	-0.43062649068206
C	-2.73109345039986	-3.12344944473893	-0.35267182431311
C	-6.04183308187945	0.72287077893967	0.13214003098628
C	-4.31912883897537	-1.10418113542876	-0.18520402744107
H	-3.76292654712094	2.24947935682428	-0.42072828250769
N	-1.13651592330799	1.40537704655760	-0.94911939065807
C	-2.06218412619591	-0.67912635967140	-0.69940390568636
O	-0.72531862482881	-0.98940205358956	-0.94074937360489
C	0.20421640331799	2.80850201741844	0.46012079613796
C	0.78911521473673	-0.84842870636302	2.58231947559089
P	0.28489772460768	0.32280084381428	-1.05868479130520
P	1.05040152011559	1.25162015546966	0.78142916861242
C	-1.02109106409892	-1.02253670708535	4.16717545042100
C	0.29147543748323	0.39807870936478	2.17720791294850
H	-2.41905241059004	0.61670582848196	4.20424239361400
C	3.54601113389053	1.92862673236943	-0.17217005068931
C	2.82132825323404	1.47293603533941	0.93821305996775
H	5.33834322268474	1.19038564132909	3.19097511961962
C	4.91856629010305	2.10000006309562	-0.06118098960138
C	-0.86719511264675	0.92710344940584	2.75562193224759
C	-1.51951774926125	0.20957512595154	3.75279626640500
H	-1.53553948886821	-1.58004600007456	4.94461362920047
C	0.13281502923943	-1.54721251494142	3.58612672583224
H	1.67586278377320	-1.27156700750078	2.11903667316805
H	2.89793645211362	0.86862393520037	3.01667712491212
H	-1.25881178641324	1.88715572358924	2.43150740421554
C	3.46117855874784	1.20856320903647	2.15333975517472
C	5.56395829347887	1.82887313221142	1.14598662160191

H	-8.02654681185298	0.45813011925463	-0.71425615140081
C	4.83679529352735	1.39249409796201	2.24953397740837
H	3.04406126661347	2.14424700756817	-1.11195626626441
C	-1.18537023385815	5.07157949752563	-0.31717802317262
C	-0.12353134090588	5.18219673310224	0.58125737260176
C	0.58815176766183	4.05032263016757	0.96326445830794
H	-1.72891305918363	5.96219392829910	-0.61875353489276
C	-0.86525500536592	2.69612892970073	-0.45600363776488
H	5.51805698566768	-2.61076658222065	1.71965507701445
H	1.42379847570982	4.12807458404934	1.65352512672044
C	-1.54877277083418	3.84043267850789	-0.85664693904482
H	0.15370314914523	6.15321062117987	0.97907426762971
H	-4.48389784011213	-3.70925794697703	0.82735856352744
H	-4.71967370136765	-3.83609236584954	-0.93252142980075
H	-3.71439872300084	-5.01819687738940	-0.07877398486088
H	-5.93481327187153	2.66206314427052	-0.87925869945818
H	-7.22114263923563	2.51853397481554	0.32733921180061
H	-5.54574719596717	2.72932020351820	0.85590840229990
C	-3.99524569200331	-3.96067251733505	-0.11995882863557
H	-1.12564314115634	-3.11522748518336	-1.85767107164264
H	-1.90191971004732	-4.68654599376981	-1.60519042685422
C	-2.08145308795104	-3.60708358352450	-1.66425533479849
H	-2.74668583454791	-3.41744232377404	-2.51407697961327
H	-0.85374964738119	-2.78370347360370	0.73835749665062
C	-1.77621372156940	-3.36203767025570	0.83440199076120
H	-2.25970002544470	-3.07539955114256	1.77519639877062
H	-1.51077194782632	-4.42437821379562	0.89221780326442
C	-6.18339767895545	2.24934131121631	0.10494231987025
H	-6.42537793604091	-0.86681540091291	1.59689223548722
H	-5.76845108336922	0.62603858769961	2.29570955973347
C	-6.44668076017422	0.22562111030833	1.53355567068645
H	-7.46439816910931	0.55836297457616	1.76815093703825
H	-6.72547648552922	0.46475487270473	-1.92467948150643
C	-7.00077638657607	0.13011001718306	-0.91827378009570
H	-6.98715100669641	-0.96417617163991	-0.90609488755260
H	6.63877023600928	1.96359093307475	1.22529936288257
H	5.48654111450849	2.44632231626984	-0.91916489552377
C	1.72804211182595	-0.49428384140179	-1.94653322819920
C	2.02957711709751	-1.13865602272113	-0.88207223076026
C	2.06308822721216	-0.29081523229717	-3.34874807538751
C	2.68953282166715	-2.13263068832172	-0.11500082075126
C	3.93382833547973	-4.05676390396171	1.50516269457013
C	3.94079468634651	-1.86706589390234	0.47839800564791
C	2.07633969652623	-3.38169999281625	0.11153092992145

H	2.21557167363412	-5.28638911299637	1.07807278097336
H	4.42354821613513	-0.91057029888727	0.30355979436883
C	4.55027627538719	-2.82706260640515	1.27478809583024
C	2.69940788447034	-4.32729101988326	0.91413953277088
H	1.11586858149840	-3.59469637003985	-0.34733709793975
H	0.54984207538754	1.19786201818540	-3.72673316453169
H	4.41383121665169	-4.80000839548322	2.13468990475057
C	2.78122354807459	0.05419747450765	-6.02157519793375
C	3.12559503708839	-1.02870225385847	-3.89166922443816
H	3.66439157358814	-1.73302233375858	-3.26336699243538
C	1.36812166521694	0.61842241859431	-4.14968261475823
H	4.30371963480478	-1.42318468555529	-5.63945047779341
C	3.47933857921170	-0.85192008333861	-5.22224520478407
C	1.72662945150422	0.78748894182528	-5.48264118693042
H	1.18221252884354	1.49484513074191	-6.10131527248987
H	3.06151290350637	0.18879752103696	-7.06226386819784
H	-5.12381672670936	-1.79988681276501	0.01744407387229

Phosphenium ion 2a + Diphenylacetylene IM-I

E[a.u.] (r^2-SCAN-3c-CPCM(CH_2Cl_2)): -2591.448668

Gibbs free energy correction (kcal mol^{-1}): 511.94

E[a.u.] (PW6B95-D4/def2-QZVPP-SMD(CH_2Cl_2)): -2596.040673

C	-4.61709928152018	0.04881370769184	-0.31490305880158
C	-3.51556444430700	0.85863713227319	-0.60314982851321
H	-0.62760688551789	-2.15543297883415	4.41220127025463
C	-2.25736663265922	0.27121593175491	-0.66684800429343
H	-2.07097086998051	3.05933080050807	-2.15643593477291
C	-3.15877638287567	-1.93844095760769	-0.12894813322746
C	-2.95671223873591	-3.42832577024142	0.15307174587674
C	-6.03718808906998	0.61758741739652	-0.23167359987755
C	-4.41713788893071	-1.32180985384226	-0.09782936045149
H	-3.62432329895065	1.92259406986339	-0.77161736375282
N	-1.02253590466932	0.87461489873980	-0.93316744415150
C	-2.09514104273596	-1.09014690796184	-0.41951802214840
O	-0.76220775838192	-1.49013861762814	-0.44646066620360
C	0.25108830413772	2.52457188664030	0.27922267072533
C	0.07284669461327	-0.74336894408016	2.95787559419462
P	0.35418725202870	-0.27921499643979	-0.66286755021990
P	0.93592017566728	1.05949725794146	1.04760023579381
C	-1.42247530162342	-0.18215922441912	4.76338071915989
C	0.03321224684940	0.59064704632214	2.53302225150233
H	-2.07158323920985	1.86998379437829	4.86224898068559
C	3.49103925136690	1.70004106981555	0.24308782861216

C	2.71494388756721	1.12592419756681	1.26006630104790
H	5.18503597711780	-0.06848206599968	3.25346383894508
C	4.87548335121802	1.62773908446316	0.32089654645425
C	-0.74271060810971	1.53583835691168	3.21129757517103
C	-1.46717900280265	1.14145566925326	4.33048238256829
H	-1.99512798559983	-0.48464825847058	5.63537033306493
C	-0.65373968609494	-1.12216273177596	4.07974034302727
H	0.66075549381475	-1.48065725975553	2.41604852289503
H	2.72242909196999	0.04629793603652	3.13712039747095
H	-0.78480549533209	2.56532630940910	2.86732045080182
C	3.32244035436951	0.48821662551873	2.34718384829616
C	5.48454249189300	0.98598962991116	1.39861032804429
H	-7.94281512948211	0.33594391445801	-1.24081064915414
C	4.70992208215351	0.42380096086721	2.41040984136067
H	3.01950162553488	2.19608724063930	-0.60106797466414
C	-0.96371330543189	4.58555348684850	-1.10783333312518
C	0.02622705781323	4.87282898035009	-0.16642252746794
C	0.64703489984986	3.83954271141781	0.52429609031759
H	-1.43894384127044	5.39692636124983	-1.65160162851912
C	-0.73981826257271	2.22622906448447	-0.68615159414436
H	6.12418180811311	-2.90932943869130	-0.93178309043426
H	1.42346621593499	4.04980462337897	1.25477213272888
C	-1.33776251301529	3.27492060657783	-1.38564000542563
H	0.31571379931284	5.90126790838445	0.02424350261523
H	-4.80972884305649	-3.74711944891466	1.28060892064655
H	-4.95619635973411	-4.08062791012566	-0.46147703366953
H	-4.08691922231561	-5.20747464990006	0.59130592167462
H	-5.70605338810550	2.38227399158640	-1.48523147478851
H	-7.10869772141991	2.48151679501215	-0.41110033209891
H	-5.48078108670923	2.67886439342895	0.25466472447529
C	-4.28832663113182	-4.14753867103033	0.40437061382068
H	-1.28650147411305	-3.68291910176160	-1.25298989463938
H	-2.16033029434491	-5.17406737519644	-0.86090612757074
C	-2.27625962395879	-4.10169738754832	-1.05543117664170
H	-2.88915673404149	-3.98160702579434	-1.95571961052817
H	-1.09202725208070	-3.15447002960212	1.28055427557876
C	-2.08188947231236	-3.59887327663489	1.41116599958053
H	-2.55975648281631	-3.12620256680284	2.27667193508946
H	-1.95293444366743	-4.66549126138844	1.62890053671214
C	-6.07442138258207	2.12940013192210	-0.48465096954050
H	-6.66855436096736	-0.72147271249453	1.38907799854970
H	-5.98996487593382	0.82195446879109	1.94139258422496
C	-6.61254827140883	0.34997162419231	1.17280745350686
H	-7.62518033071578	0.76325455951158	1.24709466014358

H	-6.53079159812647	0.10112502447345	-2.29443085693497
C	-6.92551975021873	-0.06994387803773	-1.28659001112176
H	-6.98499008481393	-1.15033984696066	-1.12188756321413
H	6.56787540769762	0.92811811387737	1.45146021813045
H	5.47982004230344	2.07244332405747	-0.46399597356746
C	1.57893153588029	-0.18825890104796	-1.90055093394108
C	1.83470239030250	-1.18636155672485	-1.00184859267679
C	2.07368887536274	0.41815572228147	-3.12104789365002
C	2.81552022836408	-2.17255112780002	-0.62425946512768
C	4.71848359015671	-4.01001956533284	0.27507274962632
C	4.14868761814128	-2.08278451782250	-1.06104759975591
C	2.45260376369590	-3.18831375104701	0.27605404494226
H	3.10779524381619	-4.89796983091651	1.39768322681818
H	4.44385271450550	-1.28158036653486	-1.73234593448489
C	5.09213404562958	-2.99191295056618	-0.60318281303677
C	3.39733554001526	-4.10808194895932	0.71070663622219
H	1.42330723255031	-3.24637342934842	0.62102528004061
H	1.03259561550076	2.27863101623447	-2.79025492455666
H	5.45943757839177	-4.72387154836817	0.62357820537973
C	3.01707436743018	1.61074317567253	-5.46617424094409
C	2.91966751754694	-0.29328097216679	-3.98655935750944
H	3.19352969242191	-1.31759865708280	-3.75033259462141
C	1.69369458805212	1.72714854306511	-3.45364837847905
H	4.03505547694115	-0.25559507570019	-5.81898796234824
C	3.38466829207574	0.30223172279723	-5.15138711787605
C	2.17048979313457	2.32012892200764	-4.61597821783762
H	1.87897868281018	3.33734780119716	-4.86112154850542
H	3.38639909524704	2.07412951063916	-6.37658289053742
H	-5.28185880684516	-1.93739831544130	0.11604157977559

Phosphenium ion 2a + Diphenylacetylene TS-IIb

E[a.u.] (r^2-SCAN-3c-CPCM(CH_2Cl_2): -2591.41642

Gibbs free energy correction (kcal mol^{-1}): 510.89

E[a.u.] (PW6B95-D4/def2-QZVPP-SMD(CH_2Cl_2)): -2596.008385

C	-4.11070382842564	-0.21444110587956	-0.19212748885900
C	-2.99499182945332	0.51060087206380	-0.61847451765030
H	-0.06584435176193	-1.16009105814361	4.96053802214023
C	-1.73800286789016	-0.04846365287433	-0.44005599069047
H	-1.61586217944719	2.29326269315764	-2.47318440683156
C	-2.66937958178812	-2.05364095312304	0.61088660170553
C	-2.48377282141535	-3.40962448353200	1.29306109816881
C	-5.53221910433633	0.33266506878265	-0.35124560792539
C	-3.92498035128932	-1.47262272440357	0.39672827352195
H	-3.10161423448370	1.48923144923392	-1.06496574017378
N	-0.47582783973280	0.50909815859017	-0.78319685378535
C	-1.59311027600641	-1.29107510381385	0.16991698967824
O	-0.25294067165227	-1.66342122762643	0.30444018463840
C	0.73787736914139	2.46184481796735	-0.01547572837532
C	0.65217615011525	-0.10527297478645	3.23348780531636
P	0.79824737085040	-0.47136060459418	-0.15241709879354
P	1.60661511615298	1.22601073209364	0.97786959091284
C	-0.52326782441833	0.94419434978042	5.05671792933561
C	0.75894515753610	1.13418495427538	2.59442069211427
H	-0.84749293342092	3.06667297560761	4.87604474383348
C	4.06892079247235	2.47490670413662	0.54144059021518
C	3.34054379762032	1.56634670190599	1.31929622846354
H	5.74921967607113	0.65319650141963	3.52886901045803
C	5.39538995568678	2.73468625354031	0.86300174733041
C	0.21652063576327	2.27941127005137	3.18665240503775
C	-0.42233924125062	2.17881698330321	4.41749815971720
H	-1.02723527073865	0.87126856435072	6.01618669621986
C	0.01494857754437	-0.19637511721688	4.46675715847072
H	1.06781939609485	-1.00199081922018	2.77771078795804
H	3.37818513434915	0.22170280860661	3.02003875518845
H	0.28474635249055	3.24392527250044	2.69140664662313
C	3.94329579632795	0.91447278163180	2.40408733390956
C	6.00235045090518	2.07728190637737	1.93024289290002
H	-7.36928985171401	-0.24348848437135	-1.35920511119394
C	5.27832327648946	1.16453763987706	2.69487163821645
H	3.61207552075417	2.97602533855530	-0.30428560379671
C	-0.63975597457268	4.09075041916720	-1.78702169181746
C	0.30270158730759	4.64335595364100	-0.92669845359413
C	1.00330503369399	3.82693518118948	-0.04046308907581

H	-1.16414488426946	4.72417529075729	-2.49602423512965
C	-0.24100829865740	1.90760651979457	-0.86663283741240
H	4.81887116586991	-4.04618825222912	-3.35928399664661
H	1.75032930785850	4.25546021367436	0.62117463635661
C	-0.91106879534051	2.72306394651287	-1.76950771755076
H	0.51029864796971	5.70825623329300	-0.95282486056303
H	-4.39897321477641	-3.43615086707876	2.35887798479957
H	-4.44598799418111	-4.23019957896057	0.76623441448517
H	-3.64108282339585	-5.01838856506323	2.13139888894205
H	-5.12318087264331	1.69563306797518	-2.01622404438391
H	-6.59008131664596	2.05771017524560	-1.09596406554974
H	-5.00853120528699	2.45855677005292	-0.41193610047971
C	-3.82944568277693	-4.05141277891415	1.65390627284664
H	-0.72836184255374	-4.01735821648022	0.11805965605650
H	-1.64710138465781	-5.35537798073013	0.82541215178335
C	-1.73544596395270	-4.37232932128893	0.34936808317008
H	-2.28508053601858	-4.49494841859153	-0.59058902032739
H	-0.67856255497365	-2.83208314173812	2.40369133075194
C	-1.68077786861758	-3.22261574412117	2.59600256837386
H	-2.19801695386985	-2.53070073402515	3.26996813317310
H	-1.57848621902295	-4.18757010204518	3.10548619894127
C	-5.55302056348073	1.71777734768852	-1.00842504162462
H	-6.26197097064799	-0.52615033654533	1.53292906829248
H	-5.61811434755670	1.12067918353331	1.68323228356429
C	-6.19184950022255	0.44691978780205	1.03695134807796
H	-7.20673475884916	0.84793461890099	0.93471033572735
H	-5.89472758013882	-0.73635438613039	-2.22003858385712
C	-6.35242656077304	-0.63158219963451	-1.23005150449696
H	-6.42551943110267	-1.62647545756056	-0.77974967490759
H	7.04216354769207	2.27981182787969	2.17013701309334
H	5.95677833243021	3.44905804222040	0.26843353928281
C	2.49799869958846	0.29240094270657	-1.29233154657037
C	2.09680623599993	-0.97858671673414	-1.22567144225480
C	3.34428354329372	1.05818367334121	-2.17352339531161
C	2.31084216350244	-2.24302128775096	-1.93557905153446
C	2.74419093636187	-4.62652718678366	-3.33465582595619
C	3.60257668639391	-2.59473671886575	-2.35212726369672
C	1.23963172052384	-3.09892992858089	-2.22606102930465
H	0.61976944194113	-4.93391817021174	-3.14764313356042
H	4.44103215828765	-1.94881416616691	-2.11533805101488
C	3.81298979336764	-3.77981947094727	-3.04732646107066
C	1.45844779753172	-4.28098296863965	-2.92343286155563
H	0.23119846664986	-2.83503382445766	-1.92123445821197
H	1.71527632634386	2.23553489378305	-2.96457880830608

H	2.91329445811778	-5.55348046482723	-3.87497502774125
C	4.98805308397435	2.66034233878844	-3.78919506116633
C	4.73813672668368	0.87369726061564	-2.18094358088195
H	5.18535208849962	0.12876624477535	-1.52953028437496
C	2.78918927102550	2.07136799012713	-2.97598521903078
H	6.62260743655175	1.52128132294770	-2.96908348119018
C	5.54654414361221	1.67242778601682	-2.98119860151832
C	3.60650421674890	2.85149219942635	-3.78385747377027
H	3.16062007725411	3.62046529377187	-4.40883961464941
H	5.62382976060905	3.28026352792987	-4.41434283196135
H	-4.80148922384040	-2.02264555667908	0.71537668033311

Phosphenium ion 2a + Diphenylacetylene

E[a.u.] (r^2-SCAN-3c-CPCM(CH_2Cl_2): -2591.489934

Gibbs free energy correction (kcal mol^{-1}): 511.85

E[a.u.] (PW6B95-D4/def2-QZVPP-SMD(CH_2Cl_2)): -2596.074741

C	3.94682328411693	1.30102840286164	-0.43766250360890
C	2.74630008649225	1.54514605288692	0.23413960857044
H	-2.82221952417560	3.05508255956380	2.24315722979586
C	2.08179797722183	0.46759711345014	0.80166118171490
H	2.32318856802195	2.53980762632251	0.31878307761248
C	3.75597354516494	-1.12514936192313	0.02134028956667
C	4.28379206216529	-2.55541444918713	-0.09510800625972
C	4.76864092541088	2.43100939687303	-1.06511068750446
C	4.41097730574361	-0.01863608008454	-0.53741138116518
H	5.33490925790527	-0.19519543303727	-1.07424899635352
N	0.85263520519279	0.49553069107334	1.48975468772361
C	2.58125190652183	-0.82812813670159	0.70651814223586
O	1.82225773972077	-1.76544760315672	1.40628198750169
C	-1.07645786289489	1.85128487881196	1.81330474554840
C	-0.49803204398063	3.08876885812551	-1.11395157715884
P	0.40502940130618	-1.10838728408603	2.01102679490951
P	-1.69677019368375	1.04220946426968	0.30953117051946
C	0.91715059039080	2.77070230492012	-3.04053525354007
C	-0.66590672094677	1.70087228729089	-1.02412750594224
H	-0.97782877552009	3.75222260578551	-0.39839282058487
C	-3.81292401321491	2.40140058755038	-0.93528039134417
C	-3.41075012668159	1.58419921649638	0.12130080587823
H	-4.02936869010585	0.48818815599652	1.88420995808105
C	-5.15049594740816	2.77074005231336	-1.04413096354444
C	-0.02264859500264	0.85020220653988	-1.92554941610067
C	0.76816377735526	1.39141235038497	-2.93417527791678
H	0.41576435929942	4.69340316042098	-2.20390718085525

C	0.28863507546231	3.61763348327613	-2.12881171539625
H	1.53436533463644	3.19000337717557	-3.82998202748883
H	-0.12177474657548	-0.22751851548203	-1.83663867214148
H	1.27309276068964	0.73009202149089	-3.63177279613908
C	-4.34135078426873	1.13291698660069	1.06541277922549
C	-6.07811408188044	2.32462602720995	-0.10751454892822
H	6.70677103449192	1.52158829760234	-0.57941590753273
C	-5.67356715700847	1.50631925401651	0.94666724945987
H	-5.46627745028471	3.40331902745367	-1.86815722044421
C	0.25410548297257	3.46926420595768	3.63286449824648
C	-1.10513102129004	3.66615454840989	3.38682077763802
C	-1.77762150174638	2.86692035729398	2.46874147124909
H	-1.64089311601919	4.45957454728460	3.89825787768027
C	0.27438402135280	1.62186746735469	2.10762162811127
H	-3.09719078648471	2.73647171515203	-1.67953501492709
H	0.77150330924129	4.10716429201411	4.34341341456829
C	0.95148371187698	2.44418978077056	3.00123669112978
H	2.00002140429781	2.26086029421262	3.21655127273535
H	5.39606413778477	-2.26184404749875	-1.95964236556784
H	6.37848597123466	-2.04655222297718	-0.49112736491315
H	5.88792961220407	-3.66764243523092	-1.00205912765400
H	4.74547196144429	4.57323241597382	-1.32137272022071
H	3.12743289589765	3.85499061519237	-1.32500418840853
H	4.01597865313051	4.04843802457648	0.20371920493712
C	5.56251064633909	-2.62408851867291	-0.93910305872142
H	3.72217504745042	-3.13865441673014	1.94642463278929
H	5.00978028629041	-4.11928071411310	1.22311043509016
C	4.60917334203143	-3.10268442048990	1.30930575421302
H	5.36387880046265	-2.47724460975753	1.79901461076808
H	2.29898528331386	-3.49582980545888	-0.18103371533034
C	3.21898568344080	-3.44476782876831	-0.76861820479186
H	2.97083688945115	-3.05990982062296	-1.76448434199115
H	3.60840701872833	-4.46305717784282	-0.88205235354297
C	4.11789457664751	3.80375742006645	-0.85966784321178
H	5.43064754902146	1.24323222525737	-2.78777944743861
H	3.92915365322399	2.14753292754249	-3.05936401096955
C	4.91323433079299	2.18487043102904	-2.57903708327803
H	5.49053608661708	2.99668068930429	-3.03663685246832
H	6.76229746497100	3.27240778266064	-0.84896501796067
C	6.16673565531254	2.45966374598851	-0.41711829089985
H	6.08796196184140	2.62613626889393	0.66314064903828
H	-6.39735359353051	1.15531120523948	1.67593578477844
H	-7.12170043408986	2.61168213600928	-0.19910606991771
C	-0.81540337723153	-1.61610198664134	0.69549870846196

C	-1.74058264437305	-0.79173332837054	0.15814448885879
C	-0.75898106543474	-3.06266665906033	0.41547621410918
C	-2.90320868215858	-1.30269484789713	-0.61789458610890
C	-0.55944707618977	-5.35323367398267	1.17477950290784
C	-0.52482980586477	-4.86162430487938	-1.18534132942843
H	-0.77970840687447	-3.67112562817408	2.49177794590091
C	-0.69258785856187	-3.99757195436573	1.45829951168850
C	-0.47344202644136	-5.78883409207450	-0.14486490905692
H	-0.45034898533518	-5.19476890491896	-2.21663068608695
C	-0.66687193589621	-3.50842876405971	-0.91050690329079
H	-0.52623347024482	-6.07053021203897	1.98970064290091
H	-0.36552107543375	-6.84733294262092	-0.36324491665065
H	-0.69570398176550	-2.78532210589068	-1.72056815886441
C	-5.13116336974870	-2.29172331149028	-1.99327161112379
C	-4.86850518838723	-2.71417863495524	-0.69112370903721
H	-2.52699754518515	-0.15727585653341	-2.41284157451945
C	-3.17827288801332	-0.87453668681591	-1.92049941043645
C	-4.28280354184483	-1.37213966940886	-2.60460365391599
H	-4.47983744712838	-1.03756317352422	-3.61903470867607
C	-3.76344401875040	-2.22395362785729	-0.00700634517377
H	-3.56348406854485	-2.54558147982136	1.01124951333896
H	-5.52986591108774	-3.42472169427794	-0.20353579094080
H	-5.99595104939427	-2.67491436346629	-2.52722875900945

Phosphenium ion 2a + Phenylacetylene TS_{conc}

E[a.u.] (r^2-SCAN-3c-CPCM(CH_2Cl_2)): -2360.416634

Gibbs free energy correction (kcal mol^{-1}): 456.6

E[a.u.] (PW6B95-D4/def2-QZVPP-SMD(CH_2Cl_2)): -2364.520705

C	-4.13773905001181	0.75899034083021	-0.01147459542841
C	-4.64761240977922	-0.53659373655500	0.03528778027974
H	-7.17135679021517	-6.29280727268987	-0.23421343962829
C	-3.76968165007221	-1.59186166011898	0.27787649547292
H	-5.69654347352516	-0.72927563685757	-0.14096355812018
C	-1.84202565704640	-0.07671475690142	0.35387999619284
C	-0.33704080733959	0.15354022871903	0.48997815220064
C	-5.04022425131349	1.97051098301271	-0.25955230358736
C	-2.75832649580674	0.96010498343078	0.16858455768889
H	-2.38211189752673	1.97515630981494	0.13949551440022
N	-4.05418068136863	-2.97121932433027	0.30169125079965
C	-2.39984498605508	-1.35600867012015	0.39994080683716
O	-1.67116255598517	-2.51757448309762	0.49214118425896
C	-5.63745516799708	-4.81345000753560	0.03864321396726
C	-5.08602238903531	-4.98964993752332	-3.10840768861036

P	-2.58060234455832	-3.93070786003011	0.32173352847495
P	-4.21129017618201	-5.76693303665136	-0.55051050190724
C	-3.60771953559860	-4.58923429362418	-4.97430598538514
C	-3.98247032346423	-5.28035950783247	-2.30155278586691
H	-6.09115884729302	-5.02927196044649	-2.69686470573940
C	-4.04642410724552	-8.30613408653939	-1.65178401300375
C	-4.63354861990425	-7.53998980096631	-0.63263136613957
H	-5.89648054434404	-7.61176502147663	1.12721835446660
C	-4.27849415823540	-9.67411914313785	-1.72030026852826
C	-2.69141436101927	-5.22001287728277	-2.83444759965321
C	-2.50742489234490	-4.88075371955708	-4.17211065480234
H	-5.75333884133315	-4.41128777771061	-5.06607724373880
C	-4.89485229791185	-4.64232912911852	-4.44201277418656
H	-3.46280036048173	-4.31744217813737	-6.01609019223910
H	-1.83004632010080	-5.44469756681198	-2.20963607918738
H	-1.50354719555165	-4.84040040916617	-4.58450958559749
C	-5.43550736022506	-8.17528521030857	0.32484210603508
C	-5.09402872520624	-10.29734046625334	-0.77748178294672
H	-3.54161862590023	3.07454200473559	-1.42501376167166
C	-5.67019140533169	-9.54411227852828	0.24016909975578
H	-3.82398340353080	-10.25301069435901	-2.51900853412154
C	-7.59723158036490	-3.33528422737792	1.35838812130018
C	-7.91821205203364	-4.57421745180271	0.81350558488249
C	-6.93231302693471	-5.31310223503220	0.16659403507841
H	-8.92070706198699	-4.97847831907975	0.91184623216951
C	-5.34204951304610	-3.51375853775206	0.50738933498187
H	-3.41764596467965	-7.83481280906842	-2.40111618264048
H	-8.34109453616402	-2.77185619713247	1.91387252316530
C	-6.32162212704776	-2.80404182112510	1.20767503701201
H	-6.08162507632401	-1.84879610596098	1.65911005456951
H	-0.27357699142521	2.11738888440319	-0.48182402319301
H	-0.46534174582832	2.19079465098080	1.28642235035591
H	1.09695972863695	1.75595797175712	0.57644113042348
H	-5.27739044697589	2.41262686380299	1.86560007581596
H	-3.93282057419737	3.28657852409719	1.10589764691488
H	-5.60198238504792	3.78917680087701	0.78918860797976
C	0.01336530596969	1.64662906519107	0.46479946988294
H	-0.00074760762163	-1.50646590230048	1.89075372825819
H	1.23289488863870	-0.23549353833334	1.93602698163672
C	0.15928106096158	-0.42729818223422	1.82931039407293
H	-0.35889967252748	0.04911912959079	2.66896541816676
H	1.47272339822089	-0.33870316016208	-0.60098833827115
C	0.39622207590432	-0.52684206568698	-0.68395823909118
H	0.23859902852691	-1.60793596787413	-0.69165554474826

H	0.04989154709976	-0.11820453617866	-1.63970449762007
C	-4.95272739071194	2.92101479573010	0.95081130263516
H	-6.64161280641462	0.92003064548953	-1.32229359911747
H	-6.91146225331979	1.06611492882441	0.43111168655198
C	-6.50848261638667	1.57358635721089	-0.45283616558785
H	-7.10525351759593	2.47611041882687	-0.62019580673793
H	-4.61242974048056	2.04842934218818	-2.39895139810788
C	-4.56855494946959	2.71008706846036	-1.52664835013380
H	-5.21608909287736	3.57396376619273	-1.71547590382252
H	-6.30575366561057	-10.01899624513547	0.98223753731790
H	-5.27940076574252	-11.36589973695637	-0.83781014585115
C	-2.56561969150076	-4.60855777974800	2.08755469913131
C	-3.34083282082486	-5.59352821831248	2.30912316478686
H	-1.92849136679162	-4.06494077625065	2.78196784621179
C	-4.13834811471003	-6.57940617087258	2.86683174216789
C	-5.70463993832376	-8.53936569907165	4.08294345378263
C	-3.66688620969574	-7.91306873853407	2.95740396322481
C	-5.41970324408519	-6.24916399154016	3.38078556755557
H	-2.69397341993061	-8.16169128066843	2.54589043693045
H	-4.07934747335337	-9.89047240258545	3.66132318472975
C	-4.44543525779684	-8.87148512840499	3.58053247081220
C	-6.18811521246174	-7.23100306561746	3.97640744321656
H	-6.31488401660720	-9.30219995787531	4.55727270241017
H	-5.77889757532542	-5.22804455973224	3.29577038392647
H	-7.16968682289495	-6.98079975209041	4.36757726212336

Phosphenium ion 2a + Phenylacetylene $TS_{conc'}$

E[a.u.] (r^2-SCAN-3c-CPCM(CH_2Cl_2)): -2360.41138

Gibbs free energy correction (kcal mol^{-1}): 456.66

E[a.u.] (PW6B95-D4/def2-QZVPP-SMD(CH_2Cl_2)): -2364.513905

C	2.86218428215170	-2.07116637397667	-0.67031351829469
C	1.58001639952515	-2.00577236151163	-1.20068637343569
H	-4.66023283935479	-1.98231188105762	-1.39834189486918
C	0.90840076858714	-0.78364647762381	-1.13108818014709
H	1.09802060690408	-2.86860063200095	-1.64118244592659
C	2.77325261658498	0.30224399125817	0.04665401130626
C	3.36496619059856	1.51887307991524	0.75537620934016
C	3.68614045648701	-3.35949402805464	-0.70641860637527
C	3.42742772601687	-0.92138191931066	-0.07175558089543
H	4.42963141749607	-1.00298685455109	0.33064694761030
N	-0.38097261697103	-0.46124762004055	-1.50338752919317
C	1.49780995907436	0.33133378110351	-0.52326977381070
O	0.66546825287327	1.40870924621686	-0.50320392634732

C	-2.63672584748041	-1.24816305027210	-1.38852361350523
C	-1.10083023537323	-0.43471830612427	2.14236361442577
P	-0.89632948870816	1.17330162341272	-1.07822282421995
P	-2.75406455145443	0.02551427566312	-0.07034552930592
C	-1.24518626597725	-2.38759620514736	3.54992927937680
C	-2.19413563642949	-0.89229826960250	1.40079907470044
H	-0.61903283244228	0.50776609228687	1.89766499829792
C	-5.19449433441286	0.36011988956864	1.25957680088657
C	-4.52876095413865	0.39582933820144	0.03094686635507
H	-4.68404824091633	0.85115895056229	-2.07994537718471
C	-6.53808632718718	0.71750451574073	1.32356253137636
C	-2.80682376289446	-2.10708376482919	1.73191691017562
C	-2.33905402739803	-2.84259514343065	2.81357309054223
H	0.22998394048904	-0.83402253014860	3.77948943238228
C	-0.62483881581312	-1.18897722043419	3.21162703536820
H	-0.87586467356230	-2.97218717468070	4.38767689467468
H	-3.64721313828922	-2.47698982102713	1.15016317053567
H	-2.82349057315223	-3.77812854509461	3.07721346413118
C	-5.20148386988966	0.81147213430209	-1.12479748238893
C	-7.21173109284307	1.12237825341322	0.17410296115598
H	2.00913211434713	-4.76113227071592	-0.84055023439254
C	-6.54172217199488	1.17103524879239	-1.04737286693285
H	-7.05755506966413	0.67809312602226	2.27648606926110
C	-2.18287039620641	-3.05006450437223	-3.46760372974906
C	-3.44123314548770	-2.96466334292806	-2.87718772802533
C	-3.67432957225649	-2.05356671059673	-1.84883123550725
H	-4.24736142189473	-3.60655206627517	-3.21890404216076
C	-1.36720655956319	-1.35059258711030	-1.98748217831438
H	-4.67343509778143	0.04861823671454	2.16098140598563
H	-2.01014285235774	-3.74999025918333	-4.27961061132816
C	-1.14101778621459	-2.23178168988531	-3.03934869746744
H	-0.17290590180911	-2.26237819188183	-3.52922287590382
H	2.41637877994274	3.02740038474177	-0.53540542669644
H	3.87690484065361	3.57522735231322	0.30073119063850
H	4.01384826070877	2.48394160380432	-1.09315285361131
H	4.63125984257026	-3.02080169326663	1.24833961179402
H	3.12304289683079	-3.95405807646450	1.31760553536351
H	4.61523585494546	-4.71026856826682	0.71764245509347
C	3.41369169415993	2.72230165211470	-0.20804673639915
H	1.46758182672184	2.12958274674367	1.68274456188106
H	2.44127308622880	1.01623716728881	2.66920564040034
C	2.48702898409648	1.86412194881481	1.97646174985251
H	2.91954721027777	2.71979386358406	2.50746913555293
H	4.81773759136903	0.42384697697278	1.97625665906956

C	4.78956966631035	1.24439484147286	1.25100523980309
H	5.46718972553169	1.00492140940895	0.42398292469537
H	5.16993272968290	2.14257388827168	1.74778946356197
C	4.03461861052364	-3.78104525873322	0.73469795519796
H	5.57863240107245	-4.02567057441941	-1.53752018126589
H	4.75887431131553	-2.79445693490508	-2.52251126657309
C	4.98508260043233	-3.10538296390469	-1.49654359789824
H	5.59938730679749	-2.32881136836359	-1.03031056849082
H	2.67958166627378	-4.28128151827615	-2.41828074060054
C	2.93160894803759	-4.51282248818645	-1.37728651388599
H	3.56712876360319	-5.40418123599320	-1.37825479734319
H	-7.06400776130940	1.48974849163926	-1.94463953412684
H	-8.25976979036957	1.40225538070975	0.22940609695382
C	-1.44539257221752	2.82634548190475	0.18047732349046
C	-2.30810840464841	2.24229988811599	0.88364638861636
C	-0.63888756646903	4.01697785664684	-0.01805965197897
H	-2.99780007678683	2.16744478079400	1.70665665941197
C	0.49628912041191	5.58987234510036	-1.46084838092567
C	0.62736634032193	5.84347351574090	0.93488282412244
H	-0.64868635102071	3.91212164338704	-2.17147961010020
C	-0.29280092473134	4.45699809290917	-1.30077155164617
C	0.96348213589674	6.28172683509129	-0.34503258696322
H	0.98976351299320	6.37953025833636	1.80753207843559
C	-0.17276989685187	4.72015602443132	1.10313948169419
H	0.75294236284116	5.92966945661708	-2.46002738978740
H	1.58902457846646	7.16054192696655	-0.47235397634769
H	-0.43503693583001	4.37141288555115	2.09769847680496

Phosphenium ion 2a + Phenylacetylene TS-I

E[a.u.] (r^2-SCAN-3c-CPCM(CH_2Cl_2)): -2360.393682

Gibbs free energy correction (kcal mol^{-1}): 455.64

E[a.u.] (PW6B95-D4/def2-QZVPP-SMD(CH_2Cl_2)): -2364.507276

C	-0.55601373200102	3.82592428453708	0.13357990029759
C	-1.33634122378020	2.67008493064165	0.04969011508572
H	-3.46758857764085	-2.75469306909342	-2.40644681571564
C	-0.74887740435649	1.45091978156253	0.36917667146610
H	-2.37077459628432	2.71097481362503	-0.26399985266408
C	1.41737630541956	2.50005064513003	0.81347117754129
C	2.89705121867164	2.39362735648179	1.18367232564221
C	-1.13012277216969	5.21045419954760	-0.18301438724203
C	0.78776242863025	3.71650488752924	0.52005937487951
H	1.37644043566581	4.62294442622624	0.58602390746085
N	-1.28840207966102	0.16400685371304	0.31912075093885

C	0.59526421474748	1.38017129077153	0.72981777768029
O	1.03659947160565	0.08495382152701	0.94241592315656
C	-2.39425608176207	-1.36630770313958	-1.15496475568644
C	-0.43439498157276	-1.94035254831017	-3.96777161487233
P	-0.08516415772821	-1.12241160367084	0.63370743566084
P	-0.74713575115364	-2.09886950789023	-1.22273100325790
C	1.31013988709066	-0.68794441226722	-5.06719037360722
C	0.09747617279591	-1.56180430962995	-2.72845557660915
H	-1.31497001841441	-2.57553536400904	-4.02372317372652
C	0.04151301203419	-4.68286703328618	-1.84023292270149
C	-0.84933082135986	-3.88533966302202	-1.11362179984774
H	-2.45473318637679	-3.83703605111119	0.33915068097163
C	-0.00080121262660	-6.06314268911052	-1.68511764196425
C	1.22596491304945	-0.74209042699192	-2.65792590496460
C	1.82771996466350	-0.30385263090857	-3.83352840196560
H	-0.21863714767923	-1.80635650126336	-6.09725899758230
C	0.18276658703259	-1.50630608900445	-5.13399497139585
H	1.78568183246103	-0.34854846918478	-5.98286672739095
H	1.64069764721772	-0.44762556640844	-1.69787880185569
H	2.70372335842172	0.33554078146086	-3.78121671451322
C	-1.76797856681440	-4.46039036816384	-0.22685109440609
C	-0.91305383380114	-6.64107479725620	-0.80412288731390
H	0.72491608684139	5.97348673228299	-1.07568249664990
C	-1.79268837684785	-5.84152094591188	-0.07632402989822
H	0.68280373936153	-6.68788005000373	-2.25184417623570
C	-4.83379746547215	-0.11363919325431	-0.75973518867642
C	-4.75451986094656	-1.23053071302816	-1.59215056639179
C	-3.53479877929811	-1.87138643111943	-1.77695878380384
H	-5.64384513594627	-1.60675315680030	-2.08755255014273
C	-2.47396345370719	-0.23395297434178	-0.31167052676323
H	0.76019324163916	-4.23137685175644	-2.51821139505220
H	-5.79174189938035	0.37369877228058	-0.60289746588934
C	-3.71058418750242	0.37671018632887	-0.10178741044503
H	-3.79677624374161	1.21065182488709	0.58661454329013
H	3.53949146745520	4.27144921820076	0.25371859409104
H	3.11189164915206	4.42563921284494	1.97443523443942
H	4.62463242687580	3.63869281722091	1.50027059558010
H	-1.57609773053594	5.66945466406004	1.90412032740973
H	0.02100496401466	6.23672000890461	1.37923763857256
H	-1.43678606422398	7.09657266796571	0.85416509588256
C	3.57253964916751	3.77019148962354	1.22721164124783
H	2.63795779787692	0.73376302079257	2.60146336290067
H	4.10292516702270	1.69947011099559	2.84912641046764
C	3.04250967628332	1.74879190596905	2.57660819121784

H	2.52254833749479	2.34806180942406	3.33224631469276
H	4.69875483196046	1.49555391412491	0.37038110712868
C	3.63032208993932	1.53831925563138	0.12976315693648
H	3.24803771500687	0.51466960047668	0.09880068781116
H	3.52044599549579	1.98087142081206	-0.86684849707432
C	-1.01970516653091	6.10536625041700	1.06675942926178
H	-2.74518575470989	4.54651872380919	-1.50472628752797
H	-3.23378928958784	4.73377333284953	0.19614546410227
C	-2.60444501975093	5.14802050394822	-0.59960529451334
H	-2.96091468768451	6.16101820126351	-0.81338836009589
H	-0.38531146633161	5.21786948120357	-2.23520100515614
C	-0.32980704076552	5.84404553617244	-1.33761762699094
H	-0.74140292534832	6.83082005498774	-1.57929152974499
H	-2.50279925329638	-6.29396963427922	0.60915079490696
H	-0.93918307264630	-7.72030748156892	-0.68440199142443
C	1.63098118267749	-2.49344950561093	0.25925326013276
C	1.05914895297414	-2.50747824965729	1.39227118363694
H	2.45374816677764	-2.73529781287803	-0.39197851748421
C	0.98566554988619	-2.94880728345349	2.76864009633361
C	0.91138995060099	-3.87262208064062	5.39841395134491
C	2.01519856965421	-3.77177010515167	3.25446121053516
C	-0.07558850670299	-2.59480871708519	3.60685403478237
H	2.83805020638315	-4.04265395396329	2.59919337465915
H	2.76674693925510	-4.86816039368006	4.93589467130654
C	1.97031550748524	-4.23048217437046	4.56323722018413
C	-0.10872734933203	-3.05460174649145	4.91796720289367
H	0.88272038566847	-4.23248377546585	6.42280420110525
H	-0.87547891663934	-1.96424694717730	3.22615235066903
H	-0.93460390434553	-2.77562980881955	5.56554073093915

Phosphenium ion 2a + Phenylacetylene IM-I

E[a.u.] (r^2-SCAN-3c-CPCM(CH_2Cl_2)): -2360.433425

Gibbs free energy correction (kcal mol^{-1}): 457.99

E[a.u.] (PW6B95-D4/def2-QZVPP-SMD(CH_2Cl_2)): -2364.548414

C	-4.31504043711285	0.28337353995636	0.02977048122435
C	-4.98113232712984	-0.87672931174878	0.43270742878012
H	-7.22144151322850	-6.16938093488520	-1.29025138253937
C	-4.22566194409040	-2.00335710625633	0.72978520690792
H	-6.06082097782155	-0.91642704049012	0.51000354356679
C	-2.12759620312763	-0.85346841907800	0.23541620804089
C	-0.60061428877209	-0.84006076069098	0.14835726411645
C	-5.07155907476337	1.56288083872460	-0.33899589373546
C	-2.91454797731666	0.27245566619246	-0.04300507640508

H	-2.41123774284947	1.18456394313848	-0.33852470820263
N	-4.67765338827954	-3.26551604498692	1.14937589460157
C	-2.83992651049198	-1.99172909239457	0.60473103051069
O	-2.27106530609923	-3.23474558681433	0.85327764298218
C	-5.99300334043242	-4.78031228692927	-0.18608616520431
C	-4.78305146716612	-3.35045303692708	-2.77810795641312
P	-3.35216762105755	-4.49285569301577	1.06930343133477
P	-4.36783486311741	-5.24871538269015	-0.78929300043826
C	-3.02974270297592	-2.54471796743224	-4.22621434289128
C	-3.86580089046841	-4.21993002475966	-2.18001825868260
H	-5.81621631073691	-3.32234640854937	-2.44415311378581
C	-3.70270963669078	-7.48491088340781	-2.31127944843710
C	-4.25270523682329	-7.00954786174923	-1.11592052178011
H	-5.14326388859713	-7.53529669098915	0.79201342036447
C	-3.60054685840367	-8.85672827241007	-2.51519021354814
C	-2.52710113998636	-4.24468495956300	-2.59216867363939
C	-2.11845341965370	-3.41120230580033	-3.62497884658317
H	-5.06329216691276	-1.83377404702290	-4.26954809979856
C	-4.35629876155757	-2.51271998167256	-3.80263031836920
H	-2.70203069103277	-1.88708141106515	-5.02616068308379
H	-1.81049121556709	-4.90359487338938	-2.10723619282173
H	-1.08287440503174	-3.42851826724366	-3.95047013499241
C	-4.70182409328053	-7.90174541175808	-0.13095545820561
C	-4.03893583515799	-9.74579256505944	-1.53729214709850
H	-3.69738815778344	2.10757510098944	-1.96115625612525
C	-4.59167290045468	-9.26827532690741	-0.34948153011582
H	-3.17969108863296	-9.22931607073558	-3.44409347264082
C	-8.34114560479164	-3.87547854713285	0.96041878855229
C	-8.37944535044084	-4.90249004704307	0.01636970918396
C	-7.20161431322290	-5.36808872414154	-0.55674578480395
H	-9.32923532706568	-5.34222930067334	-0.27095316849559
C	-5.94796584967813	-3.75485879040145	0.78199143113490
H	-3.36812919473900	-6.79615324885167	-3.08081936115096
H	-9.26586458223102	-3.52141818183602	1.40673011055561
C	-7.13393468633727	-3.30960241880846	1.36042126214812
H	-7.10376649250925	-2.53952479787498	2.12534315115049
H	-0.41493159499110	0.86793205040927	-1.21319679943741
H	-0.34294279334568	1.30586858126206	0.51053414165247
H	1.03162450607496	0.50194980166425	-0.26260224366657
H	-4.81475525209086	2.47260079280395	1.62829108375390
H	-3.54610913766608	2.91834893857426	0.47118071411215
H	-5.16140882637187	3.63092924743583	0.32568148278961
C	-0.06193277572982	0.54634777067250	-0.22725188141996
H	-0.29822045037180	-2.23227167389962	1.82123511876414

H	1.08808929817148	-1.18582940251317	1.46803053618913
C	-0.00612694086640	-1.22371535065040	1.51843418279909
H	-0.33806302414783	-0.52047986513409	2.29036273558433
H	0.96573931997225	-1.81976806045767	-0.98981984852597
C	-0.12853087383807	-1.83747041750682	-0.92661797308460
H	-0.44236920161411	-2.85950262341920	-0.70049452517453
H	-0.53450436841708	-1.56269304681640	-1.90617657191658
C	-4.61603380016565	2.71411018243779	0.57806019171463
H	-6.98099127408721	0.61967103238830	-0.84902792420365
H	-6.87329195319686	1.16718518273603	0.84056700352586
C	-6.58931280262369	1.40367732762569	-0.19107008666105
H	-7.07895642305128	2.34413710853320	-0.46487253114694
H	-5.07397765849803	1.11321389321860	-2.47467778301099
C	-4.76529360249325	1.92471431679945	-1.80551241352173
H	-5.30998451764385	2.83272515393350	-2.08956897876289
H	-4.94094596499859	-9.96176208160241	0.40941601376574
H	-3.95527115433282	-10.81601925165427	-1.70247512771832
C	-2.32688566143645	-5.73831695595031	1.74615333230765
C	-3.55369405952916	-5.69888861886537	2.33010815349424
H	-1.38237382024503	-6.25545375729678	1.89778923285140
C	-4.27147835012646	-6.33083192309796	3.41943524392913
C	-5.65094961231106	-7.60789079828525	5.47999160419486
C	-3.61734811582127	-7.27347105523239	4.22774788984664
C	-5.62116314960449	-6.03461407310697	3.65330525755627
H	-2.57034389126399	-7.50235387901416	4.04584623571189
H	-3.79889227414179	-8.63800016798926	5.87458512748672
C	-4.30693182815322	-7.90804130317435	5.25106884745996
C	-6.30542964206138	-6.67172974721960	4.68159714299263
H	-6.18754646250795	-8.10621279642075	6.28243248808160
H	-6.12876907498911	-5.30906583825770	3.02388763835221
H	-7.35141500786493	-6.43990069874484	4.85974449416735

Phosphenium ion 2a + Phenylacetylene TS-II

E[a.u.] (r^2-SCAN-3c-CPCM(CH_2Cl_2)): -2360.406422

Gibbs free energy correction (kcal mol^{-1}): 456.97

E[a.u.] (PW6B95-D4/def2-QZVPP-SMD(CH_2Cl_2)): -2364.523232

C	-4.09908288266914	0.69893603260193	0.27805781583712
C	-4.73219657076650	-0.49358260238735	0.63713809864217
H	-7.22732339845013	-5.86860232433900	-0.97463076461097
C	-3.95637617567028	-1.63391179074151	0.79000766545278
H	-5.80267305876930	-0.53544023631547	0.77846416111165
C	-1.90547299793143	-0.43256264278827	0.19396376952798
C	-0.40208949728320	-0.42324229731835	-0.08400695180398

C	-4.88520665702353	2.00031782326535	0.09498249365066
C	-2.71183313310761	0.70500501480266	0.07620884609349
H	-2.23809403165256	1.63808609059913	-0.20170821283969
N	-4.38285095649711	-2.94979093939923	1.12004603954250
C	-2.58237584315197	-1.58955353252620	0.56571868003090
O	-1.97818334898135	-2.84091368504367	0.71404379749053
C	-5.84600618506897	-4.56155978088214	0.03878108626814
C	-4.53770703009485	-5.56196732718590	-3.23445248978418
P	-3.07715849399863	-4.05690211601488	0.90196786275090
P	-4.28339880573354	-5.32716889072426	-0.45764344566221
C	-3.71536948532598	-3.94567915267780	-4.82722739113125
C	-3.99464984840560	-4.80427259372561	-2.18871305181059
H	-5.06665543702106	-6.48818756372769	-3.02661107440403
C	-3.06591852386901	-7.70137126822051	-1.07821398677563
C	-4.21513743910388	-7.12640838007445	-0.51996578191779
H	-6.13020390661106	-7.49219429539450	0.42306670291436
C	-2.94642554153078	-9.08518900149759	-1.11936550185358
C	-3.31497421492344	-3.61682644459309	-2.47272427993317
C	-3.17501601276489	-3.19064947066664	-3.78978623889062
H	-4.81711645021807	-5.72056591177506	-5.35600315876776
C	-4.39700733847071	-5.12876578716284	-4.54807402450980
H	-3.60166640829888	-3.61481699850823	-5.85557951191593
H	-2.87698516884915	-3.01290121277128	-1.68227819015563
H	-2.63817653003266	-2.27088093842520	-4.00192676946736
C	-5.24350939854236	-7.93123473211735	-0.01962905559897
C	-3.97354520826860	-9.89080526707776	-0.63136261412224
H	-3.63894919735578	2.72766833712654	-1.56003564360327
C	-5.12059760591901	-9.31379719055587	-0.09106091028996
H	-2.05253047652528	-9.53358997543293	-1.54226251081686
C	-8.08767577286784	-3.31081424765146	1.08816810392519
C	-8.23779037987168	-4.38801813525319	0.22023302055351
C	-7.11371396202537	-5.01927045896113	-0.30712331909971
H	-9.22786649980940	-4.75172483883150	-0.03543962213825
C	-5.69645512325696	-3.42857183064748	0.86748666694785
H	-2.27255917491323	-7.07512275039910	-1.47794681186832
H	-8.96318719937805	-2.83873282976905	1.52350588034395
C	-6.82277171611758	-2.82661118895706	1.41688267960180
H	-6.71463813474292	-2.00265913442697	2.11396124318351
H	-0.38106398897933	1.35489108084160	-1.36553298464123
H	-0.07329520407137	1.69822299393329	0.35398724929928
H	1.17518828165534	0.93097484246164	-0.63865346723353
H	-4.48067214582200	2.70238549947826	2.12231245255822
H	-3.30222657852540	3.27513455303412	0.92579552752132
H	-4.92419819537029	3.98623896001279	0.97622092805789

C	0.09710115503596	0.97899804131936	-0.45451649406254
H	0.10767684857649	-1.90688758225519	1.45688485543775
H	1.44205034135504	-0.84605524377434	0.97564791037324
C	0.36460652033491	-0.88402162150037	1.17161325832534
H	0.14699863851678	-0.22276391041744	2.01756231581938
H	0.97755563231366	-1.33185938243620	-1.49136607344794
C	-0.09447204030838	-1.36458650116053	-1.26658798632760
H	-0.36308242867107	-2.40091389316531	-1.04643406350216
H	-0.64202352427145	-1.04683832241772	-2.16114410172114
C	-4.36108997447290	3.05300255529638	1.09112633726458
H	-6.82628277168809	1.09366502948607	-0.36111663986818
H	-6.59362601830598	1.47673652817203	1.36183599332594
C	-6.38727386097275	1.81303811254726	0.33944670577853
H	-6.89625516994150	2.77152950916281	0.19514113591301
H	-5.04654806871934	1.77520630482946	-2.07062661162569
C	-4.69059409600274	2.51479134019710	-1.34460110177772
H	-5.25894703596366	3.44077262770295	-1.48839258835853
H	-5.92461307998723	-9.94035310435408	0.28333538134281
H	-3.88066906996225	-10.97208323416848	-0.67577626468457
C	-2.68618663719630	-5.17432716503902	2.17894989408092
C	-3.86019375254975	-5.77704417740226	2.01186552319158
H	-1.90253978711892	-5.13058641447603	2.93353904979682
C	-4.60213882742824	-6.72723744903509	2.80579161003829
C	-6.03483521328866	-8.66111343228331	4.23402150337160
C	-3.95318847135654	-7.87295611438535	3.29770634427377
C	-5.97671969053499	-6.56296971719743	3.03989818368221
H	-2.89276895619544	-8.01123530160868	3.10414979051082
H	-4.16619940379669	-9.72770769526942	4.35347332506470
C	-4.67282724301204	-8.83655314152241	3.99316442553945
C	-6.67924720120550	-7.51643488201313	3.76580843454061
H	-6.59231199543583	-9.41431742111186	4.78320224230990
H	-6.48474984462918	-5.68281796288123	2.65499617489127
H	-7.73943592013459	-7.37288484202796	3.95476052484432

Phosphenium ion 2a + Phenylacetylene 3a

E[a.u.] (r^2-SCAN-3c-CPCM(CH_2Cl_2): -2360.481064

Gibbs free energy correction (kcal mol^{-1}): 458.29

E[a.u.] (PW6B95-D4/def2-QZVPP-SMD(CH_2Cl_2)): -2364.593956

C	-3.82472729964831	0.91791993321708	0.45499112643273
C	-2.60996346737513	1.15218363611300	-0.19472420189522
H	3.04478860840287	2.55140341020428	-2.00516868931484
C	-1.98893271292000	0.08711849157071	-0.83106989503977
H	-2.14397234343186	2.13090203944629	-0.21221220436266

C	-3.73661024084826	-1.47767052379804	-0.16515394570762
C	-4.33006308421999	-2.88648626367982	-0.14272683956292
C	-4.60223801729241	2.03911481272226	1.15083202220925
C	-4.34711200357778	-0.38274093256563	0.46326943093171
H	-5.28297830519009	-0.55322889814058	0.98087484811544
N	-0.75606868276865	0.11049178778079	-1.51100580009016
C	-2.54361028784167	-1.19030983556235	-0.82295726885749
O	-1.81888730381565	-2.11550812125210	-1.57595591416278
C	1.23937720921722	1.39942983987103	-1.69573576512069
C	0.12171995885769	0.10218940771562	1.94869381928395
P	-0.38512578457765	-1.46837416571050	-2.16363277068249
P	1.80868847613575	0.45323085219281	-0.25526186568388
C	-0.78389538586541	1.94105487342566	3.21970450656454
C	0.78395953398693	1.01412295610389	1.12359816499472
H	0.20180061194775	-0.96633487554053	1.77171732290289
C	3.95588644401058	1.63063406808439	1.11204707068888
C	3.54044003889231	0.90153480822893	-0.00243131936803
H	4.14737314768231	-0.11008061308260	-1.82024258599447
C	5.30688035387451	1.92431631913041	1.27220405488335
C	0.64433292459516	2.39271644527825	1.33021982401548
C	-0.13478979215639	2.84937703473217	2.38508118972712
H	-1.18242271756167	-0.13460929496081	3.63587162250851
C	-0.66233882441955	0.57276877077317	2.99713084396102
H	-1.39502676878758	2.30414344538524	4.04100815192452
H	1.13953230881995	3.10460873680306	0.67434371507796
H	-0.23981479778853	3.91724002126305	2.55120434665076
C	4.47000496709718	0.46432784440333	-0.95451058127035
C	6.23384209240007	1.49072429565079	0.32903634369903
H	-5.34481979875243	0.76817767419230	2.77893861232795
C	5.81547341888048	0.76213224210468	-0.78399778601588
H	5.63339708585267	2.48744802729845	2.14116981295291
C	0.01694894587618	3.21087764719531	-3.40519748765376
C	1.37851334249541	3.32622360613535	-3.12405637692841
C	1.99703030040752	2.42929645286674	-2.25988268290191
H	1.95876382131884	4.13055736338482	-3.56498277189152
C	-0.11539843647701	1.25322187107832	-2.02803528065411
H	3.23961503440731	1.95402275101173	1.86104598220556
H	-0.45835988124302	3.92404749343209	-4.07228537694050
C	-0.73668735889306	2.17292029956979	-2.86573122717077
H	-1.78785740654111	2.05476188591254	-3.11172014556284
H	-6.40805498740685	-2.30577164723929	0.24148121612429
H	-6.00271998901888	-3.97575849852141	0.66241300267133
H	-5.47086087488902	-2.65476361340442	1.71399711500660
H	-5.88636930250202	2.41470703900027	-0.57449942534412

H	-6.57196177974860	1.25652843617472	0.58132367403474
H	-6.55226945227667	2.98546694263734	0.97141199764838
C	-5.62953676923612	-2.94640113244340	0.66992847622829
H	-5.36487648938236	-2.65317566419105	-2.04848646422854
H	-3.75238194051975	-3.37974342166081	-2.20432245027647
C	-4.64921322354239	-3.33915594591350	-1.58177402582652
H	-5.09709374613561	-4.33930158916986	-1.56089279383627
H	-3.75032054977172	-4.86518157195656	0.53808535694624
C	-3.32118285633179	-3.85730441438969	0.50342562295289
H	-2.38525474801100	-3.90443993783082	-0.05880653147669
H	-3.09445708933588	-3.54575472641951	1.52929484372483
C	-5.98809543143099	2.17782540700685	0.49057743590311
H	-4.48258245943860	4.15479464016379	1.55759237850502
H	-2.90025258757058	3.36111947301968	1.52766702803033
C	-3.88550340448864	3.39035433885402	1.04942278841608
H	-3.75767420294227	3.70361524091450	0.00707272343033
H	-3.80667231153379	1.57815218192456	3.12977424778009
C	-4.78065563815062	1.69600728861904	2.64213236740957
H	-5.32576343965393	2.50095052459005	3.14871215862002
H	6.53868286632384	0.42192452704274	-1.51887647393597
H	7.28778404221481	1.71771559312206	0.46115723404676
C	0.82641602006221	-2.09268615913491	-0.94071064393385
C	1.77661956065287	-1.39243172016597	-0.29988216474076
H	0.88473229520827	-3.18373908927136	-0.88936188551763
C	4.27478015130098	-2.38713088614445	2.37652403659517
H	3.32789723779382	-3.35317605420113	-1.24883423001380
C	2.87961865767015	-2.07086244764467	0.42365739494958
C	4.61935233742148	-3.75634504037593	0.42399515460650
H	5.78296554083163	-3.91795210126772	2.22950870044112
C	3.24153744036604	-1.71888706616355	1.73046234306907
C	4.96938171651178	-3.40446499052322	1.72542744628493
H	4.53761034872902	-2.11045832477732	3.39338391148855
C	3.58255989628268	-3.09545063103903	-0.22447135127348
H	2.70997729317824	-0.93175734187336	2.25775682877850
H	5.16148334060480	-4.54224076233209	-0.09396626151346

Phosphenium ion 2a + Phenylacetylene 3a’

E[a.u.] (r^2-SCAN-3c-CPCM(CH_2Cl_2): -2360.486624

Gibbs free energy correction (kcal mol^{-1}): 458.3

E[a.u.] (PW6B95-D4/def2-QZVPP-SMD(CH_2Cl_2)): -2364.599747

C	2.97882036111692	-2.07153578518072	0.10204043716866
C	1.71679958665325	-1.89033374639086	-0.47040097789026
H	-4.17850439080204	-1.57909865252219	-1.81460557409078

C	1.37342725608534	-0.62067191524005	-0.91300800848989
H	1.00658505441780	-2.70321172944034	-0.57230971344757
C	3.50490697987789	0.32900104616958	-0.20023601786995
C	4.44310926241789	1.52551954357336	-0.03966287270763
C	3.45845256740492	-3.44229789440899	0.58887110993614
C	3.82765696682553	-0.96331804257628	0.23705322306125
H	4.79309600232543	-1.11396344721510	0.70402487223986
N	0.14855307275411	-0.22119525941848	-1.48348449467573
C	2.25446172435998	0.45159242685055	-0.79873158466518
O	1.77744653215036	1.62209180614422	-1.39149627751724
C	-2.11917483560007	-0.95812707094412	-1.58789205734778
C	-0.61855518982431	-0.77287614653809	2.18806176949649
P	0.16628863258383	1.48808147257295	-1.82551637199030
P	-2.28975935001617	-0.17269365005456	0.03541789699604
C	-0.21894418048657	-3.01706763157351	2.97724586684381
C	-1.46256487492947	-1.28375233698288	1.19929629574935
H	-0.43571782314847	0.29479739010333	2.26100053314046
C	-4.62980541752295	-0.83871250055891	1.42639885542160
C	-4.05437413945686	-0.07475824891602	0.40971393159298
H	-4.38432121165693	1.40486521918488	-1.13806701503671
C	-5.99350688157429	-0.72069660682393	1.68103952714778
C	-1.67832513946473	-2.66282501764043	1.09036951011906
C	-1.05886720811823	-3.52404422786623	1.98705021989642
H	0.66688114979346	-1.25247880136549	3.83833543915497
C	0.00351983741616	-1.64650004844754	3.07433871540374
H	0.27037656071909	-3.69577260709924	3.67003739428668
H	-2.32253785240393	-3.06332062746933	0.31159513030964
H	-1.22551567219135	-4.59397338744024	1.90600050041947
C	-4.83496286643689	0.80773837677503	-0.34815023037661
C	-6.77317738778080	0.15161775784381	0.92730466898660
H	1.48583210697919	-4.34414787174442	0.87744032502737
C	-6.19470693589809	0.91568764674519	-0.08571595769735
H	-6.44423263718037	-1.31138536483541	2.47279111174453
C	-1.56556928776590	-2.65310982639685	-3.71053088361378
C	-2.87789682636772	-2.50313104313135	-3.26208514159705
C	-3.16348166271875	-1.66246793395419	-2.19114380791345
H	-3.68161457599139	-3.05582796196997	-3.73817971607844
C	-0.81091235884138	-1.06915393936087	-2.07767934019525
H	-4.02339218291603	-1.51572726519742	2.02036524594750
H	-1.35281112693312	-3.32036624230367	-4.54059903545710
C	-0.52570716776824	-1.93331633818786	-3.12830797451011
H	0.49197285262469	-2.01565538737413	-3.49859872491104
H	3.95180105577300	2.43105089735907	-1.97982933590090
H	5.51881057073435	2.91825183052374	-1.30933455567548

H	5.32351458906275	1.30423349282600	-2.02340851778371
H	4.52704696016108	-2.64928188221452	2.33350389641341
H	2.84101506107657	-3.09995283133018	2.65324691364959
H	4.07991007171938	-4.35989364184814	2.45998713273531
C	4.82572474223791	2.07742092865655	-1.42761959632389
H	2.83266163652972	2.98538017131475	0.29008671490867
H	3.46071597079988	2.24016464416026	1.77371693524508
C	3.73748781926789	2.62238357176797	0.78452182223053
H	4.41568121478240	3.47230086021542	0.92276912402931
H	5.53621978479207	0.75704807376118	1.69673851118533
C	5.73605724633477	1.14171533007892	0.69055522396421
H	6.30968692013978	0.39090497713545	0.13618580845238
H	6.36294145941835	2.03381030006033	0.79076149482269
C	3.74539890958135	-3.37950421624260	2.10122421643505
H	5.09397670472552	-4.81134269953706	0.16797286355616
H	4.57241730412395	-3.86197974577780	-1.24112769975966
C	4.75015544293793	-3.82366681166111	-0.16049682931846
H	5.55464290563524	-3.10606207690033	0.02806727801207
H	2.19125735623309	-4.64807127642119	-0.72694663803502
C	2.41947435574244	-4.54152808671766	0.33965468729314
H	2.81459225399165	-5.49891253010262	0.69518661346018
H	-6.80317398148945	1.59790728028666	-0.67158887811437
H	-7.83656235424900	0.24027480414475	1.13041257144942
C	-0.65235548922255	2.14017252710574	-0.27480993019739
C	-1.67607496670868	1.49712531570808	0.31195126358207
C	-0.18632341445958	3.45452561713748	0.18579306589110
H	-2.22943927134756	1.98060327220216	1.12010733598282
C	0.69711821315230	5.66215192471997	-0.27913474141235
C	0.43778052287191	4.92790650540490	2.00373394901814
H	0.10583307885338	4.27164335667378	-1.79984164891728
C	0.20778043329746	4.44259545850740	-0.73064319005454
C	0.81675772531606	5.90735685612234	1.08720666335654
H	0.53983460763709	5.11032417866499	3.06962922218855
C	-0.06075870237767	3.71003782118223	1.55974682051579
H	0.98607820150568	6.42376226086068	-0.99741364171850
H	1.20870221849626	6.85797620219376	1.43716431021537
H	-0.32601172578754	2.93714653058545	2.27594234960737

Phosphenium ion 2a + Styrene TS-Ia

E[a.u.] (r^2-SCAN-3c-CPCM(CH_2Cl_2): -2361.664328

Gibbs free energy correction (kcal mol^{-1}): 471.98

E[a.u.] (PW6B95-D4/def2-QZVPP-SMD(CH_2Cl_2)): -2365.774743

C	4.49498233868993	0.73737919416023	0.75331673078107

C	3.16394060232962	1.02392128838085	1.02661616858239
H	-2.77317183132153	2.70235944052343	2.01552648775472
C	2.21557536495776	0.03611132710874	0.75794676159653
H	2.85594609769377	1.97983275730804	1.42955870230644
C	3.90769517818049	-1.51150207784669	-0.11401955004283
C	4.29155724301053	-2.85312892604516	-0.73363245003962
C	5.61270342632643	1.74385814329033	1.03278015718378
C	4.83215915951725	-0.52033699830606	0.20518177381455
H	5.87837716930156	-0.72082675644045	0.01035355936312
N	0.83328983203274	0.08627237312679	0.92264564856835
C	2.57938007607060	-1.18351646595114	0.18291049821928
O	1.49503319887054	-1.96416394333406	-0.08067883595895
C	-1.10609325996354	1.48659355198849	1.41463024350661
C	-1.88457705082900	3.47943475108831	-1.15507483729030
P	-0.01262894546578	-1.25652522661577	0.22811065642609
P	-1.78746767999727	0.83579612692720	-0.15046967088004
C	-0.60616907577243	4.23212857445327	-3.05954938197781
C	-1.37506114444178	2.18531177460017	-1.32316652534112
H	-2.57985635691304	3.69631470712069	-0.34857029522686
C	-4.46039787863208	1.44741852767195	-0.82133162304114
C	-3.58999104318543	0.86313313804127	0.10302242378056
H	-3.44687755377078	-0.30471817365366	1.91937977904088
C	-5.83724211638743	1.36795837871044	-0.62773369839720
C	-0.46802320543310	1.93008375059057	-2.35487408924266
C	-0.08399926010765	2.95285450986314	-3.21986322159467
H	-1.91121040041909	5.49329035614384	-1.89575207827847
C	-1.50633708071477	4.49374691882466	-2.02635587637566
H	-0.31034878719287	5.02915484700175	-3.73587544808445
H	-0.04746053597811	0.93683582402087	-2.48171593494092
H	0.62366845094566	2.74611754560170	-4.01737360888032
C	-4.11375863897286	0.17011347484411	1.20325205796261
C	-6.35382840882096	0.69609361872014	0.47550608327702
H	6.08505834622000	0.89337854323183	2.98799984390214
C	-5.48849923407228	0.09356995851102	1.38799014419539
H	-6.50590103810201	1.83238006378266	-1.34706132968560
C	0.08093064088668	2.30034043205362	3.80538429305242
C	-1.18242413880212	2.77469953205416	3.46029331472689
C	-1.77642766254420	2.35672279654625	2.27479603380558
H	-1.70960696302204	3.45820176084801	4.11876918299518
C	0.17281914528010	1.03241279856598	1.76351029149286
H	-4.07025079180785	1.96545548879475	-1.69239627999405
H	0.54075823587628	2.60103972943407	4.74203590904875
C	0.75778228751142	1.42080919666394	2.96919629831922
H	1.72055393578769	1.01817957927082	3.26506949226768

H	6.19597652738985	-2.20735374671512	-1.60446179053630
H	6.34514235591647	-2.90561152049409	0.02645396131560
H	6.03024994031666	-3.95267383348514	-1.36506845390609
H	4.56215377883608	2.90225624594887	2.56523590932707
H	5.91593489274018	3.73866709361526	1.79227265458931
H	4.38833217666202	3.55551016500107	0.91838833267848
C	5.80797156352844	-2.97490205258952	-0.92590138515943
H	2.74767730111475	-4.01074545140872	0.31968623801666
H	4.13565095012692	-4.95903863345640	-0.23888356143454
C	3.83293731272788	-3.99811348772763	0.19183015071339
H	4.29798187711346	-3.90305692067095	1.17922263671349
H	3.92006432915736	-3.94183156336747	-2.57246215994493
C	3.61986894666381	-2.99256624317487	-2.11478276316782
H	2.52965231824292	-2.98147080164443	-2.04016266027737
H	3.93161670150334	-2.17800988355491	-2.77784139174637
C	5.07733901425552	3.05881202517456	1.61069486068578
H	6.81876473400326	1.16351124859488	-0.70989547842454
H	5.67281877471879	2.48282267407739	-1.02095161294442
C	6.35807500261687	2.05794562918518	-0.27906415797178
H	7.15355035785389	2.78669115736898	-0.08667703886218
H	7.06045460283155	0.21766414522834	1.66814589944788
C	6.59744260390603	1.13312770387075	2.04963547260329
H	7.39700321940803	1.85057832549380	2.26637624700640
H	-5.88555644826633	-0.44014373984393	2.24683084110802
H	-7.42837463190862	0.63549490342289	0.62319129206897
C	-0.49443665110077	-1.92345218137537	-1.98746211468034
C	-1.78540446210206	-1.49408768394343	-2.19712253507055
H	-4.02789265634461	-4.56530728956077	0.46007432625621
C	-5.39564725923806	-2.23067576131337	-1.60101789943816
H	-1.95641211915802	-3.58329448493060	-0.42215605665054
C	-2.96975744030301	-2.13116583370684	-1.68044735030902
C	-4.08196659814418	-3.75222084564666	-0.25805007136278
H	-6.23585355907272	-3.72099724065368	-0.29100751874089
C	-4.23289521247526	-1.65738231528873	-2.08835663956396
C	-5.32423547946002	-3.27674370833140	-0.68054486026819
H	-6.36181400227178	-1.85626002388122	-1.92593714310116
C	-2.91372213252027	-3.19396896176436	-0.75467689649807
H	-4.28441032050810	-0.83138972536277	-2.79259184506666
H	0.31859451200595	-1.43273517456760	-2.51708020239926
H	-0.30219062260342	-2.95153005244807	-1.68826201732408
H	-1.94794784498192	-0.59368436774903	-2.78861501837857

Phosphenium ion 2a + Styrene TS-Ib

E[a.u.] (r^2-SCAN-3c-CPCM(CH_2Cl_2)): -2361.651524

Gibbs free energy correction (kcal mol^{-1}): 471.43

E[a.u.] (PW6B95-D4/def2-QZVPP-SMD(CH_2Cl_2)): -2365.760794

C	3.12019158164592	-2.18387716994439	0.09139941743493
C	1.87891783155484	-1.90546800947745	-0.46722005641941
H	-3.86403987055513	-1.75618838294491	-1.87247184381085
C	1.53529690478527	-0.56768166835981	-0.63183251745065
H	1.18377185021002	-2.68139673070019	-0.76473396355441
C	3.63810961938537	0.23587774372140	0.32709029457610
C	4.55800366939687	1.37567090646760	0.75625020126208
C	3.60422459515399	-3.61758587037677	0.31403380051995
C	3.96218504831380	-1.11335158162394	0.46541739874394
H	4.92597420877778	-1.35745866663421	0.89488487901609
N	0.34626173284364	-0.04466817795583	-1.15140980626100
C	2.38113520861441	0.46630217023861	-0.23640819456193
O	1.82243478728960	1.70635391967765	-0.45763674651592
C	-1.91656824195554	-0.94798858187820	-1.43116068187295
C	-1.65021254280953	-2.72007387348200	1.20368076086474
P	0.28537771769972	1.65312270351773	-1.06601819727087
P	-2.25470711934349	-0.16309250871885	0.18674987102656
C	0.02585992907619	-3.15191358938094	2.88449155107454
C	-1.39456789921870	-1.34550445673720	1.29667762774798
H	-2.39923901447057	-3.08914469383432	0.50718073289762
C	-4.55599753337077	-1.03755808039106	1.57903579011200
C	-4.03942243017377	-0.36503921972789	0.46697660789937
H	-4.52216664347691	0.84861837637696	-1.25963458470841
C	-5.93160214813981	-1.06870208316735	1.79636072467174
C	-0.41060846319504	-0.88762387861130	2.17694228289357
C	0.29700422976640	-1.79017536416682	2.96827470720950
H	-1.15591230063584	-4.67984660381600	1.92710940802902
C	-0.94785040507619	-3.61613429458525	2.00039836285075
H	0.57712485999868	-3.85556540197405	3.50193008241484
H	-0.17198686145310	0.17023547248674	2.23851233145504
H	1.06544100331575	-1.42523191629265	3.64372608664667
C	-4.91451186811328	0.29803223577488	-0.40684905983739
C	-6.79800904993142	-0.43295364486770	0.91173545280366
H	5.22966167164101	-4.88166891667051	-0.37696306983979
C	-6.28626981013873	0.25078562964027	-0.19077225949240
H	-6.32499303229148	-1.59564191583266	2.66101582942628
C	-1.09487270797703	-2.20621461648610	-3.79237172843016
C	-2.42250056576131	-2.29273362319639	-3.37412467469064
C	-2.83372536337080	-1.66678647791373	-2.20326815707810
H	-3.13983799714548	-2.86134048963143	-3.95842492719031
C	-0.59594458436123	-0.84244950661425	-1.88395916212785

H	-3.88979461410913	-1.53921063783317	2.27453903058590
H	-0.77705603281969	-2.69294984270506	-4.70931533009340
C	-0.17502947630909	-1.47692176391811	-3.04916437869890
H	0.85617565157067	-1.37698423958678	-3.37557667681642
H	5.73474968986664	0.23318369225954	2.20927855572310
H	6.43812376386131	0.26766400713873	0.57374307739539
H	6.50728677030723	1.70533369952278	1.60373174298819
H	1.62648973112218	-4.53698973199577	0.48247413871807
H	2.30595325476772	-4.58230344272206	-1.16257036362092
H	2.94109238483928	-5.65704222518113	0.09080626417009
C	5.88494617051908	0.85156022545422	1.31759387034952
H	3.97255816698133	2.74088285785757	-0.86652137410004
H	5.56061332848042	3.06780811556674	-0.15503713849155
C	4.87166719922987	2.27201797571114	-0.45881766613322
H	5.35022526505948	1.68880372920096	-1.25315559504153
H	2.93264654657767	2.65453191350389	1.49957508957122
C	3.86246696148732	2.20344593906978	1.85582214129829
H	3.63351938898559	1.57452599247972	2.72324523854883
H	4.52809771301439	3.01037072178557	2.18195981621324
C	2.55009641066765	-4.65183332735081	-0.09656708164791
H	4.72816671394175	-3.15874455159290	2.14686740293584
H	3.04044143170363	-3.63366571886234	2.42240095187840
C	3.92702198656198	-3.82224313726757	1.80695644645156
H	4.25177943223295	-4.85507089706656	1.97586565642468
H	4.66788959730161	-3.71118700282173	-1.59054239343706
C	4.87543225744886	-3.85512791478487	-0.52439840912682
H	5.68285409697768	-3.17420504230406	-0.23684791147713
H	-6.95687217936316	0.75621667928373	-0.87976237187341
H	-7.87025066328451	-0.46321403266954	1.08300596449718
C	-0.63622575522828	2.89700594691017	0.88671288689728
C	-1.80292955345823	2.23631517509828	1.22147289804694
C	-0.57447317348382	4.12177884286823	0.07110753381403
H	-2.51157521336361	3.91190595985483	-0.87501741416978
C	0.70165513819292	6.06146268777962	-0.64677499568109
C	-1.49331594968469	5.66840398345709	-1.55509630728822
H	1.40339750034664	4.60796546380761	0.77603893246831
C	0.58802872572880	4.90664077779723	0.12094194280117
C	-0.33919204757721	6.44962180674831	-1.48627065413327
H	-2.30637800224512	5.95890803704232	-2.21481754069788
C	-1.61073320482114	4.51406305871458	-0.79152526197894
H	1.60717847728481	6.65888834066384	-0.58791362405858
H	-0.25200969857467	7.34991046164640	-2.08753366993519
H	-1.84073914138014	1.63299631168327	2.12173380880856
H	-2.76364305532428	2.53990804980251	0.81681414705257

H	0.23684100946312	2.70768689404619	1.51164105039891

Phosphenium ion 2a + Styrene TS-Ic

E[a.u.] (r^2-SCAN-3c-CPCM(CH_2Cl_2): -2361.65704463356

Gibbs free energy correction (kcal mol^{-1}): 471.26

E[a.u.] (PW6B95-D4/def2-QZVPP-SMD(CH_2Cl_2)): -2365.76664752264

C	-3.43892064645044	-0.73234315932534	0.10630889814263
C	-2.19381364201937	-0.87730864970218	0.70573899449566
H	3.25919875737389	-2.29420801464522	2.97169443198404
C	-1.51128669582965	0.28102822328504	1.06259815837095
H	-1.74571653484043	-1.84553467856636	0.89286902612546
C	-3.28666649174843	1.74455726543655	0.23012223521793
C	-3.85908363112114	3.13767610272094	-0.01996748473436
C	-4.26274180657530	-1.94007129701709	-0.34301222460779
C	-3.95371704387011	0.56562939107993	-0.10376501385335
H	-4.92880856316737	0.65221065592481	-0.56705585550181
N	-0.24645156054314	0.38538578361886	1.64949815399045
C	-2.03172377262765	1.54921629227205	0.81305308889134
O	-1.15883631198831	2.54689833490541	1.18052908275272
C	1.67716493207514	-1.01898119422974	2.26601297449044
C	2.26317386150493	-2.63629562692757	-0.54409446045857
P	0.33092966408076	1.99043690115700	1.68585890620245
P	2.54692447161810	-0.18408528688440	0.87851529923320
C	0.67063478200579	-2.94620067713591	-2.33337245952170
C	1.91191625426871	-1.28278658877137	-0.45888387496970
H	3.00512501975766	-3.05282047180129	0.13221591460340
C	5.09984288078798	-1.14905572611416	0.11532454168543
C	4.30468305931823	-0.60430660389453	1.12946280908037
H	4.31208560767509	0.24450073594567	3.12125264833151
C	6.46853966211172	-1.31817851841285	0.31164396191411
C	0.92484367104924	-0.77888882182629	-1.30902075075198
C	0.30403669430223	-1.60790008959953	-2.24117953338699
H	1.93362595949199	-4.50599061156124	-1.54546803204977
C	1.65251802686883	-3.45835832381671	-1.48397337122139
H	0.18819200587668	-3.59437557439993	-3.05973182307245
H	0.62330389769157	0.26100435577731	-1.23725063623683
H	-0.46662965374520	-1.20361519142830	-2.89159911393853
C	4.90964472543769	-0.20586575790424	2.33120124548810
C	7.05807448769027	-0.94362406224232	1.51535653867516
H	-2.58714635074128	-3.32626748028340	-0.59051679568032
C	6.27400845219460	-0.38574525554615	2.52475894800282
H	7.07388285393265	-1.74664317207220	-0.48236334255427
C	0.09290190219199	-2.28177057337459	4.19861706716601

C	1.43326044271794	-2.63469274650202	4.05159059623408
C	2.21908408640979	-2.00602837332737	3.09254262633220
H	1.86593726019464	-3.40730466296013	4.68025696207440
C	0.33328383692359	-0.66940574051396	2.43406988328591
H	4.65690790561940	-1.43773324158345	-0.83272094357808
H	-0.52245615597051	-2.76608227919083	4.95078542883262
C	-0.46074870046806	-1.29339252813760	3.39520390938461
H	-1.49673441369164	-0.99287987619403	3.51988206230651
H	-5.24463717747819	2.57594941110524	-1.62169101414595
H	-5.96850652286301	2.54921455699033	0.00482141311340
H	-5.62595035603472	4.09175635746939	-0.79232755131214
H	-5.09426318173970	-2.71231209161369	-2.19473119280128
H	-5.08059876963067	-0.94259333996242	-2.12237398721007
H	-3.56814416025285	-1.82719938648500	-2.40894142013328
C	-5.25763408975037	3.07152966116073	-0.64481256467289
H	-2.99145329502082	4.03276831682390	1.79143765581430
H	-4.40277218523491	4.88486138392140	1.14428642275392
C	-3.96732298754280	3.89475313143757	1.31904357769893
H	-4.61647113397925	3.35263165019437	2.01533765929260
H	-3.36929262974420	4.89341075757175	-1.19484066113403
C	-2.93681318129480	3.90854008009083	-0.98612153497998
H	-1.93975936882898	4.05901296573894	-0.56479972037559
H	-2.83643449289607	3.36948562069651	-1.93469034681125
C	-4.51674620338041	-1.84320769800620	-1.86007613399851
H	-6.20254107990159	-2.81252931713374	0.09696096348348
H	-5.44756067969398	-2.00653821982731	1.48909617198743
C	-5.60885097950052	-1.94517234680866	0.40709724415612
H	-6.19393385390808	-1.04396624097942	0.19838227276362
H	-3.36140389719857	-3.40735168440150	1.01048855555726
C	-3.54486739193345	-3.26479262145487	-0.06043384157965
H	-4.17167044899185	-4.09350841549044	-0.40508256148434
H	6.72754290783198	-0.08359834188700	3.46452311984836
H	8.12507400667941	-1.08015002112769	1.66667977844036
C	1.17764824391921	3.07601602290937	-0.19323434501159
C	2.47350697169833	2.66600804149163	-0.45232412690225
H	2.08829542157886	1.11383127201751	-4.81025249247638
C	4.83855190879191	1.00557748071600	-2.81156552815624
H	1.14115728449723	2.17840564700335	-2.80975453238273
C	2.96265093697190	2.01639058764182	-1.64221037716729
C	2.70636165863611	1.24370362875840	-3.92662346727947
H	4.45014024043525	0.35273334230957	-4.82733379933791
C	4.31059926539809	1.60388148145791	-1.67830291075496
C	4.03697098818005	0.81837865594672	-3.93719725658730
H	5.87602485247286	0.68421849641167	-2.82026228846758

C	2.17215164344410	1.83841505710946	-2.79622931431711
H	4.93286738165988	1.75204688568020	-0.79945541033817
H	1.05916704044906	3.96349139638587	0.42804710142979
H	0.39194788870373	2.91687718955443	-0.92886897047103
H	3.23472623967878	2.91922746035188	0.28534673677282

Phosphenium ion 2a + Styrene P-a

E[a.u.] (r^2-SCAN-3c-CPCM(CH_2Cl_2): -2365.829082

Gibbs free energy correction (kcal mol^{-1}): 473.31

E[a.u.] (PW6B95-D4/def2-QZVPP-SMD(CH_2Cl_2)): -2361.708847

C	3.92847269613415	0.93423979089915	0.33015638036408
C	2.60485727326774	0.87301766432481	0.77381057051622
H	-3.23193204457021	1.61552285983951	2.21308283880210
C	1.93419695713539	-0.34054585847414	0.69201785178901
H	2.09394031870069	1.73920671638765	1.17753551132193
C	3.86523786917048	-1.45847632394567	-0.29824650318862
C	4.53317099265225	-2.70578344362668	-0.87823589984161
C	4.74728901356963	2.22699388561707	0.39644407693132
C	4.51640470245850	-0.22055324117127	-0.20395254588724
H	5.53606884883209	-0.15543681365203	-0.56275675531769
N	0.59613520684307	-0.59533314593868	1.05230318124953
C	2.55793998146092	-1.47788951516567	0.18168463422812
O	1.76414137197906	-2.62169398201641	0.25127887045553
C	-1.43499665023309	0.58189956289860	1.59300359343138
C	-0.24926908370806	2.74077477089682	-0.35615238609056
P	0.18356732130721	-2.26899655600647	0.69360240007085
P	-1.82600618441618	0.48953229574044	-0.17491459531760
C	1.14873812959895	3.31905984517109	-2.23332627528670
C	-0.61245003434219	1.55111798086657	-0.99930846070815
H	-0.64820591405062	2.98195564464207	0.62558546956125
C	-3.62342167646526	2.21340319166152	-1.40370954646131
C	-3.46969173665866	1.19973929312329	-0.45191967994896
H	-4.48702276209639	-0.08547590079223	0.97002994476524
C	-4.88926130916609	2.72776665571711	-1.66659350798102
C	-0.09962726905396	1.25306367667723	-2.26518180589356
C	0.78974446235667	2.13501420560376	-2.87127493986011
H	0.89054136309862	4.55196144967225	-0.48461710254229
C	0.62121703279222	3.62475381648365	-0.98118320673760
H	1.83743006631588	4.00715335938430	-2.71492479796345
H	-0.38690156007017	0.35166920967495	-2.79593601678763
H	1.19507706367620	1.89552544068914	-3.84960288174864
C	-4.58884872176542	0.70616935644372	0.23405054315269
C	-5.99868393403165	2.23972474098188	-0.98297182534536

H	5.79861355857418	1.64840508579785	2.21990070332141
C	-5.84639613928886	1.23218275772144	-0.03245656464893
H	-5.00413275901821	3.51358336990951	-2.40697868990326
C	-0.46360826858013	1.18847888291086	4.12886829967038
C	-1.75812342051977	1.56351314924591	3.77027782462442
C	-2.24427904475297	1.27271762749398	2.50160336670693
H	-2.38532002443738	2.10217119189727	4.47338626638409
C	-0.14559740280184	0.16651539956688	1.97524569242338
H	-2.76355810733912	2.60247637481616	-1.94009697270209
H	-0.08597258936244	1.42598505379743	5.11914655092126
C	0.34371478185120	0.49100131043731	3.23788200227819
H	1.34291705725929	0.17212290835645	3.51999010578497
H	5.97771652015355	-1.65877579418709	-2.15113960796687
H	6.60855628303633	-2.08643841010557	-0.54261429541400
H	6.38601387374791	-3.33893264282110	-1.77233836051100
H	3.69584025158323	3.11302433888077	2.09932563729936
H	4.60927070366429	4.26013422883239	1.10589674733445
H	3.06783913884695	3.62389255261589	0.51545733027364
C	5.96026319769191	-2.41772417098771	-1.36117020737830
H	3.61976006138855	-4.10260241698414	0.55034451250893
H	5.11186231561202	-4.68765167537100	-0.20822816163660
C	4.61101049267427	-3.80325976345755	0.20213814974147
H	5.18960533846911	-3.45112563749289	1.06346160458122
H	4.20535655680893	-4.09429060054095	-2.51493143052737
C	3.71765931621914	-3.21182237161911	-2.08520736294057
H	2.69975137164243	-3.48924442602572	-1.80043405057786
H	3.66284582995083	-2.43925053931328	-2.86052311387508
C	3.97622122216645	3.36729286401175	1.07075055916420
H	5.71528351478454	1.91036897863701	-1.54611657017280
H	4.20909047018929	2.84502887917535	-1.62525284671951
C	5.11462893443715	2.66849295748771	-1.03336708785627
H	5.69450489687539	3.59843223085951	-1.00271631126058
H	6.66946884185443	1.21849267761012	0.73551947386038
C	6.03690160517458	1.97941648609186	1.20280326100226
H	6.61848471254478	2.90620133166948	1.26926826221003
H	-6.70956024814422	0.84921208734719	0.50353298296956
H	-6.98498012165027	2.64544016045945	-1.18936475247567
C	-0.54268006772365	-1.98042535740629	-1.00158290682309
C	-1.86442602253132	-1.18825300361067	-1.00389989559963
H	-4.45835023860530	-3.56087067239438	2.11394150571578
C	-5.28443858279071	-2.83140685826542	-1.09668394405304
H	-2.42789599595561	-2.35172512226109	1.46857848595000
C	-3.08682287640830	-1.96431138155993	-0.55841962333935
C	-4.37094867013542	-3.16747917309515	1.10535034133239

H	-6.30858653375373	-3.87437340385326	0.48566128112935
C	-4.13278966811515	-2.14506474288724	-1.46787249075315
C	-5.40821007167561	-3.34194268773505	0.19288526087646
H	-6.08788056399255	-2.96236897469676	-1.81586190897159
C	-3.21688387499414	-2.48353123422281	0.73332103087490
H	-4.04876778618540	-1.73600616878365	-2.47196814894272
H	0.22078006217677	-1.46372096884031	-1.59614955607939
H	-0.74056978013277	-2.95466980872746	-1.46652349356476
H	-2.04968620220438	-0.87913692099125	-2.04356129697708

Phosphenium ion 2a + Styrene P-b

E[a.u.] (r^2-SCAN-3c-CPCM(CH_2Cl_2)): -2361.716875

Gibbs free energy correction (kcal mol^{-1}): 473.44

E[a.u.] (PW6B95-D4/def2-QZVPP-SMD(CH_2Cl_2)): -2365.836569

C	3.19979456984208	-2.04337009380344	0.07691528421951
C	1.90495618485769	-1.87208807032582	-0.41751637294385
H	-3.99595552108273	-1.82588908387645	-1.87271995415528
C	1.51211864665292	-0.60186835321558	-0.81788453547884
H	1.20797701373991	-2.69752014977303	-0.49964158927788
C	3.67745624771255	0.36497633852805	-0.24071636222391
C	4.63249420498895	1.55740433554079	-0.16971769731426
C	3.71040646537755	-3.40798817938174	0.54851376236549
C	4.04446045861257	-0.92766739081209	0.15997290868882
H	5.04224976328740	-1.07165865036505	0.55531129513569
N	0.24959417525346	-0.22577005282791	-1.31630811831079
C	2.37839510978314	0.48614932285410	-0.72921650910756
O	1.83046125319361	1.66709342091376	-1.22976949423369
C	-2.00264800111660	-1.06145306798091	-1.53175964364509
C	-1.26034549298402	-2.43444198747910	1.19783451144653
P	0.19342853533524	1.51452072879592	-1.55774928075809
P	-2.40760477635213	-0.20749653738885	0.01044842397571
C	-0.33511724017944	-2.45787563667279	3.42479174651498
C	-1.55653862945209	-1.07528387877756	1.34438010332709
H	-1.50102588703848	-2.95615564347451	0.27570668102710
C	-4.72098995563791	-1.11136007817904	1.27381965912500
C	-4.19526912749263	-0.31524354213595	0.25316579454821
H	-4.64496576578813	1.02321119496052	-1.39473643739317
C	-6.09988210425004	-1.17624408429559	1.45084292739748
C	-1.25202621380729	-0.40981501320165	2.53784068026782
C	-0.63419114771345	-1.10496877864551	3.57090351127698
H	-0.43226585476223	-4.17860004100620	2.13261480482959
C	-0.65553866886691	-3.12207345062839	2.24399254673850
H	0.14605198947835	-2.99648289270447	4.23607500856760

H	-1.49229796766303	0.64054627862054	2.67304815872117
H	-0.38860311552123	-0.58666319438239	4.49274225638680
C	-5.04594081960054	0.41219221130087	-0.59002982088645
C	-6.94707092589804	-0.45322445501753	0.61638315103197
H	5.35773837276938	-4.75566043184368	0.10967389446619
C	-6.42052276265468	0.33863630537985	-0.40310963794679
H	-6.50979552201645	-1.79336099356593	2.24457574704620
C	-1.21602936157625	-2.77663125267296	-3.57431651793519
C	-2.56014993034812	-2.70191350456479	-3.20804400834497
C	-2.95629619449089	-1.85243090047905	-2.18327333844816
H	-3.29886937935700	-3.31794076587011	-3.71068951172684
C	-0.65649364190821	-1.10381802666204	-1.93740693091336
H	-4.06288069554022	-1.67577811689983	1.92773611116600
H	-0.90913311184746	-3.44712783490064	-4.37177462985725
C	-0.26525176359786	-1.98000557505027	-2.94817617820725
H	0.77687375017534	-2.01047580476840	-3.25236402889677
H	5.89423181027234	0.78943099675423	1.44904350081072
H	6.50025007765498	0.40978048011564	-0.18085619598920
H	6.63076215507792	2.05559605966523	0.45507271811868
H	1.75934539629888	-4.34649787891967	0.86678709112320
H	2.43917841689977	-4.62488963564919	-0.75333725582168
H	3.10309849523184	-5.47630394818275	0.65079425646977
C	5.99136845720330	1.16724524758397	0.42523098509887
H	3.95019377126479	2.45626352246523	-2.05389156898760
H	5.57744215535984	2.94447465198958	-1.54686730365926
C	4.87524225945574	2.10401686641412	-1.59118074420893
H	5.30992831300671	1.32776311304168	-2.23079633311133
H	3.08176124712928	3.03534085579057	0.31093387265174
C	4.02394307837943	2.66084353838334	0.71739787776303
H	3.83730296305881	2.28199305783577	1.72880082789600
H	4.72296166156193	3.50202712117688	0.79007620276166
C	2.68581581789163	-4.52311767842567	0.30970525196390
H	4.77410152845230	-2.58888252750495	2.28233032559132
H	3.10219647744227	-3.08076711321209	2.61821005414235
C	4.00897868636944	-3.33921994985310	2.05864184315301
H	4.36992307261492	-4.31100167502914	2.41542269004019
H	4.81321719537010	-3.80877603858390	-1.29207428739838
C	4.99972792270459	-3.77118242125437	-0.21292610551740
H	5.79881924659619	-3.04653078884966	-0.02946420187013
H	-7.08143710778751	0.90046892881825	-1.05609630284134
H	-8.02257777590230	-0.50626462119054	0.75881540483488
C	-0.53133878850746	1.98143916681238	0.11739248019946
C	-2.01592263521352	1.58548974178529	0.10440762057265
C	-0.33411847551863	3.45366906751174	0.36319796739512

H	-1.59033253226322	4.11170791536047	-1.27072034278750
C	0.73573457997388	5.23242191195239	1.61365818832126
C	-0.71045028289076	5.77171215088608	-0.23549709775804
H	0.98702920400882	3.13645079347932	2.03193667821744
C	0.50817929838008	3.87679506914446	1.39547359347468
C	0.12616143719872	6.18418609823387	0.79940948555668
H	-1.18637677758387	6.50772591791302	-0.87742352379689
C	-0.93888541008064	4.41630519870401	-0.45491998627134
H	1.38992614329380	5.54500871534715	2.42288896966725
H	0.30248649240278	7.24244689185211	0.96956543660836
H	-2.50700908808138	1.92764496732569	1.02584376406828
H	-2.55396084291014	2.05634854909266	-0.72877266600048
H	0.01095659166824	1.40651190195475	0.87913255225399

Phosphenium ion 2a + Styrene P-c

E[a.u.] (r^2-SCAN-3c-CPCM(CH_2Cl_2)): -2361.71687498628

Gibbs free energy correction (kcal mol^{-1}): 473.46

E[a.u.] (PW6B95-D4/def2-QZVPP-SMD(CH_2Cl_2)): -2365.82642605234

C	-4.10369298951983	0.98766920890144	0.51352019964386
C	-2.76359770954067	0.86185220965804	0.90163973247184
H	3.29816130882773	0.81368387543534	2.54150836042496
C	-2.08778359325548	-0.30992646139897	0.60312963284501
H	-2.25343106376932	1.66462225408748	1.42401675584742
C	-4.04272077565960	-1.25884691464109	-0.51519620748730
C	-4.71740853061508	-2.38817830377014	-1.29508759630255
C	-4.84579168472094	2.28394656746793	0.85035013591792
C	-4.70768130054060	-0.06615094671585	-0.17577795270814
H	-5.73944858925664	0.03853870457515	-0.47991123157153
N	-0.73523893448133	-0.61827490566112	0.86559409432586
C	-2.71878619393080	-1.33872020680196	-0.10354326180489
O	-1.89074244723311	-2.44075211856126	-0.32114365645045
C	1.44141299018563	0.16084069214479	1.64695720644132
C	3.48377150186311	-2.67977817228427	-0.78921931611061
P	-0.28723290648878	-2.06593036834308	-0.01933588516733
P	2.32674154574267	-0.30613696984800	0.13471371029304
C	4.15024160868570	-4.49468365170630	0.65393753326402
C	3.03748940837672	-1.96365489818144	0.32869463687670
H	3.41441878996088	-2.26561293281921	-1.79044867818474
C	3.41320673651840	2.25243571503677	0.07625197611298
C	3.67628257697389	0.88407127562751	-0.06960231116767
H	5.16924598360100	-0.61451726971490	-0.52475201151185
C	4.43089691234099	3.17219170804740	-0.13733742070759
C	3.15163004827627	-2.51754165466187	1.60861360920635

C	3.71119806849584	-3.77948967995061	1.76558944358711
H	4.37951398015632	-4.49784146780630	-1.48874163720997
C	4.03664875899791	-3.94403716095920	-0.62009059137945
H	4.58036198142913	-5.48403158314710	0.78014948814219
H	2.79256254639427	-1.97657025382379	2.47880315886516
H	3.79630535290521	-4.20727709104025	2.75985273733538
C	4.95410592992195	0.44473019277083	-0.42444489437823
C	5.70462936819602	2.73636504820316	-0.49946631431806
H	-5.35876194839203	1.64353466385660	2.87340955515028
C	5.96513520963630	1.37670385099157	-0.64070452683418
H	4.22682647351574	4.23245931312250	-0.02269004378194
C	0.27923555016254	0.92834513750207	4.06444589094292
C	1.65480010077241	1.07808539706483	3.88299237573434
C	2.22889264715512	0.69002681549759	2.68456570476655
H	2.27479692404108	1.49785755255678	4.66821959524243
C	0.05586181334252	-0.02502741922595	1.84954615042291
H	2.42259511392802	2.59911458344583	0.35643066514111
H	-0.18086466114541	1.22209492218043	5.00352638175297
C	-0.50868657514562	0.37900518709646	3.06759165853713
H	-1.56998625060733	0.22593808213832	3.23235635027789
H	-5.24007426208446	-3.52515879699966	0.49133716997837
H	-5.21178095316164	-4.48115504652477	-1.00443093146216
H	-3.69023968943973	-4.01010507402984	-0.22634330957427
H	-6.77518204817169	3.22038284405420	0.62854570834484
H	-6.35618352624498	2.16741161122898	-0.73071923671687
H	-6.87534370076701	1.46364380039202	0.81855706615343
C	-4.70655434867590	-3.67987134429366	-0.45312732422363
H	-6.25899869694285	-1.16566598078142	-2.26180962584380
H	-6.60850033674613	-2.89869896570527	-2.18660873472851
C	-6.17715479279794	-2.05860346053468	-1.63249289904338
H	-6.78002729115568	-1.90822085455936	-0.73019470560113
H	-2.92579240131988	-2.91187189954551	-2.45396012031871
C	-3.96457330068856	-2.61694868624724	-2.62148375962823
H	-4.45858972382424	-3.41179224895780	-3.19197779821822
H	-3.97261601615666	-1.70431079833478	-3.22795410156307
C	-6.29755896813560	2.27278051206923	0.35824532811691
H	-4.08631505996635	3.34453372075776	-0.90077930157115
H	-4.64672386659835	4.40519421846057	0.41108386207595
C	-4.11922082351705	3.47074219081493	0.18739267715074
H	-3.09119785657167	3.56918538559470	0.55096087396364
H	-3.83885355833323	2.55489701216671	2.78211798232659
C	-4.85414615177885	2.48106053794668	2.37881322623783
H	-5.38669432263280	3.40433658198350	2.63465348276373
H	6.95888089901477	1.03468418614962	-0.91335650427332

H	6.49743486299990	3.46005791315314	-0.66591865747942
C	0.16750211380701	-1.23356980788993	-1.62583195117047
C	1.37016533014351	-0.26225587312605	-1.48396678031160
H	-1.01413771383931	3.76629411052883	-1.19686566874055
C	1.68212161443777	3.12972851095780	-3.16033481478063
H	-0.63133845388720	1.42082901808833	-0.57572627100945
C	1.08140447015227	1.17418994047551	-1.86493978375108
C	-0.19148608962762	3.22023266141702	-1.65006010260819
H	0.46322840981461	4.87965389273620	-2.85807077432083
C	1.90531822852955	1.80407840590829	-2.80147970207287
C	0.63633279334067	3.84368061450910	-2.58112705326695
H	2.32936720718672	3.60500140097353	-3.89188507709397
C	0.02819268614382	1.89312192609088	-1.29499413043024
H	2.73184044104947	1.25534059446774	-3.24721999430436
H	0.40876514953697	-2.00908309452540	-2.36128728866072
H	-0.70566063461520	-0.67865557642501	-1.98812392556642
H	2.16468730542273	-0.60921660879111	-2.15693025127099

Phosphenium ion 2a + Benzaldehyde TS-Ia

E[a.u.] (r^2-SCAN-3c-CPCM(CH_2Cl_2)): -2397.607007

Gibbs free energy correction (kcal mol^{-1}): 456.97

E[a.u.] (PW6B95-D4/def2-QZVPP-SMD(CH_2Cl_2)): -2401.75898

C	-3.78605058717411	-1.05231878371876	0.23354105887308
C	-2.61718919033347	-1.04026671339845	0.98625307764170
H	2.61381787537384	-2.50964633452047	2.56481573079560
C	-2.00654560876840	0.19096647080861	1.19352446892508
H	-2.18608682299224	-1.93692448350515	1.41444320506592
C	-3.67127883121341	1.40520976890670	-0.12496711944614
C	-4.20096904548738	2.69869237514384	-0.73725756019225
C	-4.57924131451980	-2.33865188911164	-0.00448136837867
C	-4.27368354447735	0.15949510248520	-0.30265387370496
H	-5.18101014563886	0.12000499208438	-0.89245951110478
N	-0.85268591574879	0.46801307743051	1.93699865599938
C	-2.51809098079537	1.36919493352895	0.65849992615737
O	-1.74006292469947	2.46173759054071	0.99477013032909
C	1.05944923884407	-1.07305669739147	2.18084556209153
C	0.41617206777250	-2.44428669260591	-0.69511239735819
P	-0.42029657988914	2.09244274397250	1.88839373621062
P	1.74591525577301	-0.32552116051533	0.64661184479069
C	-1.01609401547421	-2.10258960337662	-2.60715342594362
C	0.66326144990639	-1.06780867091538	-0.63439419182449
H	0.87949031236325	-3.11304840252844	0.02677808408524
C	3.74058606443666	-1.74123789710914	-0.76674326054688

C	3.38535853752469	-1.09148902464257	0.41983597950561
H	4.12182731971328	-0.30181471911298	2.29610955677337
C	5.05725052207123	-2.14559597582968	-0.97675011484068
C	0.04780473857158	-0.21310232151208	-1.55342619547380
C	-0.79202441435780	-0.73093681739225	-2.53715167007139
H	-0.59869988435813	-4.02813856732032	-1.73278956301384
C	-0.41583169011920	-2.95832519127333	-1.68304168765818
H	-1.66238426670767	-2.50812909069096	-3.38052494446237
H	0.21614730673458	0.85914366757341	-1.49497436058231
H	-1.26886324102645	-0.06138484445556	-3.24744674275401
C	4.37436194598642	-0.83608318465342	1.38214077096787
C	6.02881763620416	-1.90949741992730	-0.00854351711830
H	-2.88433952380097	-3.71315032012330	0.14527642228759
C	5.68253365079817	-1.25585407947614	1.17377725572807
H	5.32109001531142	-2.64902410406671	-1.90269536468599
C	-0.09353448096826	-2.03482267747053	4.56013374949267
C	1.10771309997219	-2.56240218483954	4.09466257619134
C	1.67902098768234	-2.08557461027844	2.91880943464683
H	1.60428976912715	-3.35450093663409	4.64741927905677
C	-0.14883604354464	-0.55581878726091	2.67165870875025
H	2.99345938686057	-1.92855069892851	-1.53241547566879
H	-0.53732375672849	-2.40348553937769	5.47971728218231
C	-0.72827875341475	-1.02561563991740	3.84424040554563
H	-1.66692015497170	-0.59886054577724	4.18613306347345
H	-5.30461074936596	1.76847023983326	-2.38503756828268
H	-6.28751819906357	2.06112974950534	-0.92916838065105
H	-5.81304340390412	3.41185207597221	-1.96939876877769
H	-5.33142270622596	-3.49185492173769	-1.68018566428831
H	-5.30983514979592	-1.75039299091852	-1.99342499415413
H	-3.79207902927452	-2.66985253790291	-2.01136657777868
C	-5.47753213954381	2.45758877252163	-1.55118154741072
H	-3.64197784298080	3.98073851869878	0.96085488719795
H	-4.93804627738643	4.62128240313792	-0.06057867651057
C	-4.52776801252926	3.70710888821137	0.38236096246482
H	-5.27545812028377	3.29189506333604	1.06688382428668
H	-3.51633505457513	4.19931751363503	-2.14569662646267
C	-3.13099120951934	3.28486177606994	-1.68129262920307
H	-2.20899638926533	3.53670975998556	-1.15079530485251
H	-2.88942473822334	2.57087064028627	-2.47655744983827
C	-4.76331407424605	-2.56940751254801	-1.51610782016665
H	-6.53962298563845	-3.11504723943054	0.51816369863361
H	-5.85443189062303	-2.02093772098978	1.74013640746432
C	-5.96121876303141	-2.19566441058365	0.66377626563040
H	-6.53169973465221	-1.36493217243923	0.23606125207791

H	-3.76691005435128	-3.48625321066286	1.67467063889673
C	-3.87644717452102	-3.56550021353802	0.58736728126738
H	-4.47420137217118	-4.45822865458891	0.37734021779084
H	6.43658797562810	-1.06150054049548	1.93132942481646
H	7.05403273468059	-2.22793775968903	-0.17482333190894
O	0.80029435963515	2.85200566246137	0.01155071942249
C	2.00745729678682	2.58823587967058	0.17386750868103
H	3.19311563847042	2.43159683187737	-4.30626977466111
C	5.22966691839514	1.99834550910639	-1.61092218795329
H	1.52136917358408	2.75683524733157	-2.49546712032807
C	2.95932258874796	2.44783082965962	-0.91708783335630
C	3.49454260745720	2.36092476971251	-3.26544357832708
H	5.55271461214338	1.93699021442778	-3.73713481353066
C	4.29952800971458	2.18252256731911	-0.59797732758340
C	4.82619354780961	2.08421252305082	-2.94288753324216
H	6.26602283370704	1.78516819175061	-1.36677832115681
C	2.55960347148222	2.54307753898605	-2.25916971493257
H	4.59813716290797	2.10904770677853	0.44488573006338
H	2.42876167620425	2.52508489938188	1.19046010592596

Phosphenium ion 2a + Benzaldehyde TS-Ib

E[a.u.] (r^2-SCAN-3c-CPCM(CH_2Cl_2)): -2397.603354

Gibbs free energy correction (kcal mol^{-1}): 456.84

E[a.u.] (PW6B95-D4/def2-QZVPP-SMD(CH_2Cl_2)): -2401.754642

C	-3.79628317093806	-0.91794302403027	0.30468410737236
C	-2.52438163952577	-0.90339357941278	0.86297199266372
H	2.77606081523369	-2.49851254208684	1.79923499473282
C	-1.87751028211648	0.32635869103840	0.93378917662942
H	-2.03785957438318	-1.79427221114060	1.24151626582442
C	-3.72558437662398	1.53121534239998	-0.13082943430107
C	-4.34595798046436	2.81702153409076	-0.66939728214397
C	-4.63054637896900	-2.19667837643217	0.22053255761311
C	-4.35251674572312	0.28795111141716	-0.18151487346694
H	-5.34125529087900	0.24265948988794	-0.62081412771176
N	-0.62224404280376	0.60823531798715	1.48037909454283
C	-2.46137939441259	1.50042701724273	0.46029875436139
O	-1.64393590855135	2.59224775119920	0.66548104485948
C	1.22827182829568	-1.03010634579402	1.52831841118905
C	0.22728033098621	-3.00267965354057	-0.79405812337460
P	-0.21277163910912	2.22617735870741	1.36111951548485
P	1.60879150802756	-0.57743687290977	-0.22267511521184
C	-1.38979876713347	-3.20739109893875	-2.57356618020274
C	0.42456055781355	-1.65661192062463	-1.12366022234550

H	0.78186555675402	-3.44985528385435	0.02748000050229
C	3.47656440826702	-2.32145506333247	-1.47242528822247
C	3.24351222581001	-1.35717309024525	-0.48722213112156
H	4.16739703272212	-0.07991782041310	0.99434199558371
C	4.76938263644285	-2.78699148738384	-1.70576313239726
C	-0.30480020807649	-1.09109603409949	-2.17369933742031
C	-1.21050704923734	-1.86549775615451	-2.89623075498563
H	-0.81504047622501	-4.82103210691454	-1.26525235351327
C	-0.67143610920932	-3.77481992710452	-1.52099387782231
H	-2.09335485453334	-3.81321366404903	-3.13798028797376
H	-0.16895805945983	-0.04162983059979	-2.42426320914225
H	-1.77561524539460	-1.41856995190057	-3.70930076073145
C	4.32853640652154	-0.85219076876719	0.24434192207123
C	5.83817783235105	-2.29923781287608	-0.95896186425728
H	-2.95436488537235	-3.60093528224138	0.19264941483922
C	5.61333773854141	-1.33084363664218	0.01887417624673
H	4.93786834317552	-3.53787914741037	-2.47279250442991
C	0.52795999094802	-1.63191753267186	4.19129058349198
C	1.59587430754638	-2.28142359504302	3.57801064848835
C	1.94152601760225	-1.98269548669720	2.26437990151166
H	2.16364325100002	-3.02930400586217	4.12377273128046
C	0.16570041797659	-0.38493930531723	2.17397992316921
H	2.65108115262593	-2.71401866125994	-2.05911048313987
H	0.25911726911286	-1.86033495011042	5.21796677504095
C	-0.19381345072932	-0.67694415370790	3.48544695041952
H	-1.02848072337720	-0.15167168524585	3.94095007468199
H	-5.68250310242118	1.86292446895373	-2.11894816792332
H	-6.43597013702585	2.17447026064456	-0.53547773717267
H	-6.12775371491412	3.51109553459463	-1.65303451486487
H	-5.59383463117512	-3.39646503069208	-1.30852931680877
H	-5.62045566747474	-1.66399802290590	-1.66915581724648
H	-4.11476064745498	-2.58310349589087	-1.86426631645618
C	-5.73004381010667	2.56302011850136	-1.27764716427300
H	-3.54082733900560	4.11419973785523	0.91462409630842
H	-4.97813319223008	4.74329669997147	0.09497470305917
C	-4.50312916086438	3.83390899612100	0.47900125948374
H	-5.13790342943719	3.42361287483141	1.27189761531419
H	-3.89506648268600	4.30447049028447	-2.17985807141413
C	-3.43478023920223	3.39908897991501	-1.76930013007579
H	-2.44666822266039	3.66570739975437	-1.38588217124796
H	-3.30783646627776	2.67810250290145	-2.58429522489421
C	-5.01221322855739	-2.46991112976772	-1.24705793399695
H	-6.51499742336367	-2.92195111805703	1.01797402854870
H	-5.66302950068501	-1.80299213538617	2.10472934639142

C	-5.91157762927299	-2.00812949218551	1.05758257135686
H	-6.52383801602019	-1.18239959386165	0.68152196636843
H	-3.61333972725385	-3.29478754634267	1.81797217085681
C	-3.87282160827394	-3.41448795818005	0.76011209748882
H	-4.50857354818451	-4.30116812396487	0.67114983553245
H	6.44318333884643	-0.93701396525280	0.59921979534730
H	6.84398574139628	-2.66750387136149	-1.14052327822827
O	0.60664735134646	2.74291647067990	-0.93257753599762
C	1.68724505896610	2.33551439664303	-1.37802486341696
H	4.53977756230434	4.25974052570605	1.73937076344915
C	5.39646082185413	2.33275768027147	-0.93306679331578
H	2.27375000145833	3.83861129014669	0.81800814013181
C	2.99884664103345	2.60962918046457	-0.80096566106032
C	4.41682915004861	3.64451019364674	0.85289727363369
H	6.53063055312888	3.30521509981946	0.61484915272731
C	4.12906094080060	2.08108044769923	-1.44029726247482
C	5.53953445472480	3.10870029197618	0.21540239181452
H	6.27164912348575	1.91978618200262	-1.42548274653501
C	3.14931259458311	3.40340797205247	0.34626225676719
H	4.00336549595349	1.46633880251152	-2.32881319316752
H	1.69821072011097	1.77371093674416	-2.33028423269432

Phosphenium ion 2a + Benzaldehyde TS-II (Inversion)

E[a.u.] (r^2-SCAN-3c-CPCM(CH_2Cl_2): -2397.599414

Gibbs free energy correction (kcal mol^{-1}): 457.32

E[a.u.] (PW6B95-D4/def2-QZVPP-SMD(CH_2Cl_2)): -2401.758851

C	4.18297165655602	0.82494452255850	0.95631459306662
C	2.79505092213679	0.85316503490127	0.90677444197766
H	-2.98620675018963	2.17313505781671	1.18481235577358
C	2.13422866521285	-0.21434726056766	0.29794590433941
H	2.22842614310928	1.67267428042577	1.32962833608547
C	4.24114814140212	-1.34514225656615	-0.23543323293356
C	5.00341707712937	-2.51168481337811	-0.86050367666812
C	4.99327663508461	1.95570452045422	1.59382256854432
C	4.86752593232847	-0.27688890014688	0.40087568540789
H	5.94937933258825	-0.27957216568465	0.45381187400917
N	0.75313690913291	-0.42547653928083	0.17482023429273
C	2.84310415095623	-1.25341230055843	-0.30775494162386
O	2.02672330842775	-2.08890554144485	-0.99512528457287
C	-1.34449793542848	0.90614408955892	0.60796468429035
C	0.09428799234121	1.73936913444030	-2.12644662984135
P	0.32397796464785	-1.70132800080689	-0.91510831338160
P	-2.11560672965680	0.50476125893942	-0.97188037201184

C	0.74070867914098	1.43824904541044	-4.43002972229357
C	-0.98236703347950	0.86904721799864	-2.32810684276207
H	0.26397143239380	2.19171350248963	-1.15310278974653
C	-4.76399158092535	1.20711652560602	-0.40844156423817
C	-3.65200270159263	1.41348258841550	-1.23960162656880
H	-2.87338823886685	2.49331340525720	-2.94785597041580
C	-5.93886643259095	1.90730977509121	-0.64870729311016
C	-1.20801772905702	0.29665571835416	-3.58621929927595
C	-0.34331733469743	0.58631745277294	-4.63383946044718
H	1.79658791845674	2.68570234953750	-3.02475542879776
C	0.95591531081410	2.01658669184880	-3.18091661114873
H	1.41944019865783	1.65454138239625	-5.24997225814777
H	-2.05066186425621	-0.36981400551828	-3.75177413933969
H	-0.51394643616062	0.14169893156870	-5.60945292622273
C	-3.72945550653441	2.32182970555167	-2.30339093883653
C	-6.01697777637464	2.80540355485167	-1.71102425661757
H	5.32837910418412	2.98679978509957	-0.30123034963331
C	-4.91345739460444	3.01305258353877	-2.53370270883276
H	-6.79655839019317	1.74608917170626	-0.00273262040239
C	-0.52655842712883	1.16316059609830	3.26639136623028
C	-1.66411155071093	1.83194165517710	2.83082402499795
C	-2.07711216325411	1.68163587406904	1.51467405842839
H	-2.24241310880327	2.44780593718329	3.51171077945200
C	-0.14561440098447	0.28834244661359	1.03678415185030
H	-4.71825825152060	0.51199620181466	0.42360823403869
H	-0.21294572165919	1.22819741602350	4.30400039186343
C	0.22254568753889	0.40425477914933	2.37763450681906
H	1.11101693567187	-0.11214587637567	2.72506523906556
H	6.94457768491817	-1.51261353811968	-1.06022516031336
H	6.74087137496936	-2.40076137286486	0.46903646632466
H	7.00531968883551	-3.28014013327340	-1.04495121197425
H	6.55603354428135	0.63120307319542	2.38733875112933
H	6.42378416402494	2.19271052868809	3.21170630290495
H	5.21186623611400	0.92693397514198	3.50739501713331
C	6.51178656939312	-2.40881465675744	-0.60239932185164
H	4.66393402633014	-3.84320987274820	0.83395861546667
H	3.44132540895234	-3.99934891862114	-0.44469239737977
C	4.50415175931780	-3.83648348536830	-0.24999336044367
H	5.06278161288716	-4.67347979823460	-0.68374078260315
H	5.35169828061482	-3.33677539471655	-2.83668255046635
C	4.77880030004686	-2.51815536198882	-2.38631611549487
H	3.72551226154910	-2.65740908921605	-2.64115716354220
H	5.12203310641886	-1.57602948231724	-2.82809682767125
C	5.84824883010199	1.38644622797583	2.74283664638965

H	3.43284339907910	2.68044949254710	2.94785457907970
H	4.72579318850032	3.84009835858402	2.60803745118029
C	4.09734282079639	3.06140587572534	2.16378558397854
H	3.48571386510717	3.53020221968353	1.38473616288726
H	6.49733651461391	3.39674230335377	0.97327461990433
C	5.91646226174594	2.58023364794799	0.52919318308238
H	6.62061316959371	1.84718748885766	0.12324551131097
H	-4.96969400543977	3.71642860109183	-3.35884018830303
H	-6.94038219075301	3.34683663120113	-1.89576296203219
O	-1.25722497844198	-1.82248579094974	-0.01577318063867
C	-2.31803882749779	-1.42934245682195	-0.82740300716922
H	-6.83424760429295	-2.44069265763423	-1.25622460218455
C	-5.08298373703161	-2.18631035797407	1.64607020433225
H	-4.59688857639127	-1.92409233749121	-2.18782606618029
C	-3.65018374193492	-1.80374033718528	-0.25609804944774
C	-5.99243066972987	-2.28582599074696	-0.58757436702126
H	-7.15004985231912	-2.59591022856277	1.20119397990570
C	-3.82396158341300	-1.90076828357777	1.12672769471644
C	-6.16850413931689	-2.37435175566494	0.79191613085567
H	-5.21756360720218	-2.26140121884559	2.72134380881897
C	-4.73599204743552	-2.00083199922129	-1.11165874933401
H	-2.97626782919487	-1.74893824512437	1.78818372936691
H	-2.22345531703989	-1.78265319235777	-1.86927354737862

Phosphenium ion 2a + Benzaldehyde TS-III

E[a.u.] (r^2-SCAN-3c-CPCM(CH_2Cl_2): -2397.633744

Gibbs free energy correction (kcal mol^{-1}): 457.4

E[a.u.] (PW6B95-D4/def2-QZVPP-SMD(CH_2Cl_2)): -2401.799176

C	-3.80440690152163	-1.03102563314929	0.39560492777016
C	-2.54766983708636	-0.89882734239285	0.99342558149292
H	2.99519491211251	-2.05050013071992	1.98144685230435
C	-1.90714869701125	0.32708652034359	0.90353835064117
H	-2.07282157902559	-1.71668532390782	1.52258348407202
C	-3.70586664505087	1.30582866941460	-0.41682747991600
C	-4.31228135798147	2.48411810719039	-1.17873780134051
C	-4.62366332044465	-2.31996146729667	0.52100821027546
C	-4.34350292032081	0.06216057648611	-0.29717755497990
H	-5.31867872585810	-0.05305782212659	-0.75391688077835
N	-0.64006654474983	0.67534872473461	1.41219218939245
C	-2.47648941014061	1.39863140162337	0.22706495185834
O	-1.69462475632371	2.54931501380848	0.30616817974975
C	1.31954215409907	-0.71850383222501	1.66291813587506
C	0.02407432622024	-2.35705414570041	-0.96093624824781

P	-0.32361747928802	2.38000222069515	1.25547654344906
P	1.62471322681451	-0.31100928613220	-0.07726521276375
C	-1.68223677149447	-2.15319075985744	-2.64984810934132
C	0.28272435086103	-0.98510407938917	-1.07444424262224
H	0.59357085145053	-2.97058345466516	-0.26646572748403
C	3.18850346473961	-1.71755125806799	-1.88157793155561
C	3.16323298784674	-1.08491552323672	-0.63333294419832
H	4.34970925892657	-0.45442981394043	1.07158891983162
C	4.38014035697863	-2.25156277338168	-2.36120006062732
C	-0.45980248089191	-0.19381352964821	-1.95345116476863
C	-1.44303517618591	-0.78579255905768	-2.73914282850764
H	-1.15069053315332	-4.00219950770450	-1.67640265739978
C	-0.95518070074096	-2.93708506220281	-1.75568886114270
H	-2.44287169514670	-2.61009943977436	-3.27607357658048
H	-0.29325161580239	0.87645286398064	-2.03151125844985
H	-2.02268230600718	-0.17228471418012	-3.42163786073775
C	4.34038641213578	-0.97981782110842	0.12217722613555
C	5.54281589154000	-2.16191682358838	-1.60223693866416
H	-2.92337747326446	-3.68489366175436	0.70571249084756
C	5.52051259526276	-1.52680483662045	-0.36190597828855
H	4.39491706674409	-2.73899865365630	-3.33121388396136
C	0.52751162462754	-1.44270468370596	4.23103234215418
C	1.69978622352054	-1.96327498134238	3.68847772167037
C	2.09939306108716	-1.60986801344631	2.40400818514932
H	2.30503667357621	-2.65763212812557	4.26259805116070
C	0.13615361778561	-0.19979906035088	2.21279514605270
H	2.28747444894787	-1.79242260082007	-2.48181613889116
H	0.22337434204825	-1.72232743199530	5.23529980044830
C	-0.26039058822485	-0.56373171828655	3.49401576345045
H	-1.17892672524744	-0.15308881461914	3.90315023149375
H	-5.54762609897550	1.29244461036853	-2.54002713145467
H	-6.40450010299334	1.83936187350823	-1.07908626653935
H	-6.03163883063411	2.98740475626884	-2.37252294098900
H	-5.62640340570811	-3.74811581529179	-0.77081876708813
H	-5.61290490724105	-2.10486000385482	-1.42971503968881
H	-4.13270656885016	-3.08177547462122	-1.46224340459093
C	-5.65317181127050	2.11710235080851	-1.82633879502867
H	-3.62695747838442	4.01234138534459	0.24206644171914
H	-5.01445391156060	4.49334556142006	-0.75170870840833
C	-4.55656911187603	3.65803983100556	-0.20949675051057
H	-5.23861732206235	3.35539370021134	0.59285361769804
H	-3.78153422762070	3.76040461936163	-2.85362361519382
C	-3.34745790167685	2.92024570415527	-2.29953270161651
H	-2.37941236945241	3.23678319250482	-1.90320104277770

H	-3.18178241874929	2.09638251637473	-3.00281217757598
C	-5.02273747907409	-2.83887736324921	-0.87262455736085
H	-6.49244457000010	-2.93082662569392	1.45487979823247
H	-5.64145416685226	-1.63711849708520	2.32827209379516
C	-5.89873279840250	-2.01716553252873	1.33316784353692
H	-6.52265001929009	-1.26956720745028	0.83284053035075
H	-3.59268665965832	-3.15211829957000	2.26593891912630
C	-3.84782309070126	-3.43166818152303	1.23770206109965
H	-4.46779562275898	-4.33319128247218	1.28247152050051
H	6.42780584553564	-1.44567213399641	0.22895688111223
H	6.47061395230056	-2.58287013671139	-1.97894882263861
O	0.96249546011844	2.44628074778158	0.21223404321949
C	1.84720105810749	1.54075432146600	-0.44079703448747
H	5.24353037960973	2.75244598883621	2.51741324754650
C	5.52309138076783	2.36757237506742	-0.84723429433964
H	2.89679126504454	2.16335399130595	1.99476916457536
C	3.26220655853460	1.94514131804185	-0.11930923484319
C	4.95427362669403	2.54670528071980	1.49090103013024
H	6.92882795448540	2.88328494948114	0.69938154468450
C	4.20575184784534	2.03051803977704	-1.14254775364438
C	5.90030368314702	2.62021989706367	0.46933997679033
H	6.25446142977850	2.43457487074654	-1.64739385305317
C	3.63643148469880	2.21257209107241	1.19917871765965
H	3.91173517902945	1.82470256867172	-2.16912884237491
H	1.68760716173297	1.59361160258489	-1.52737664160053

Phosphenium ion 2a + Benzaldehyde P-a

E[a.u.] (r^2-SCAN-3c-CPCM(CH_2Cl_2): -2397.633359

Gibbs free energy correction (kcal mol^{-1}): 458.27

E[a.u.] (PW6B95-D4/def2-QZVPP-SMD(CH_2Cl_2)): -2401.800479

C	-3.85356797207003	-0.93095658796567	0.37531567548423
C	-2.55570601555611	-0.88425269484016	0.88973225608373
H	3.15071894135106	-2.04137389948939	1.90861824238868
C	-1.86284575319861	0.31541190605404	0.81093345386079
H	-2.08568989585003	-1.74685822570853	1.34714749987793
C	-3.70123655273950	1.43579172882462	-0.33681407323454
C	-4.29377302583929	2.66862694362886	-1.01899215586315
C	-4.72011425961372	-2.18947901626147	0.48295965427147
C	-4.38484035224013	0.21650748503333	-0.23218082695981
H	-5.38731923195709	0.16194934804281	-0.63802776887079
N	-0.55600536737840	0.58633714978324	1.26000432286465
C	-2.42892356189902	1.44235638401104	0.22572016130811
O	-1.59816633032643	2.56476579970846	0.30859264125636

C	1.42404366951137	-0.79645498591746	1.52847138530789
C	0.09873408477081	-2.62886129644798	-0.81644746879982
P	-0.08876811762772	2.21760150724720	0.88638903619736
P	1.74396033218489	-0.50822379826628	-0.23273134879532
C	-1.57948619134917	-2.64890190561855	-2.54573803864987
C	0.40490767189487	-1.30206830873849	-1.14480076837333
H	0.63454852663410	-3.13937080245284	-0.01964276469270
C	3.36665565121317	-2.06677267035248	-1.84958175638925
C	3.32024023182284	-1.24663091637654	-0.71638269681513
H	4.47965067876606	-0.29179422054838	0.85128259430353
C	4.58576675556453	-2.58383144622112	-2.27511260324751
C	-0.29262601502731	-0.64338428534753	-2.16137948398922
C	-1.28886483908783	-1.32319047898141	-2.85457523745451
H	-1.11787669764910	-4.33271262172307	-1.28131200865884
C	-0.88819497676137	-3.30021428470841	-1.52626000564139
H	-2.34921818102067	-3.17726404905210	-3.10062463064931
H	-0.07958613151911	0.38977283899668	-2.41219260244498
H	-1.83569450189982	-0.81188688482248	-3.64099773267418
C	4.49814553125150	-0.94078169481025	-0.01899462856395
C	5.75325500493773	-2.28798578416073	-1.57775445523531
H	-3.07716814396656	-3.63313253921683	0.53981114127962
C	5.70776458551380	-1.46808521504831	-0.45183647682598
H	4.61898910762692	-3.22026647244092	-3.15407527563525
C	0.60371236410746	-1.51418414574029	4.08883835846788
C	1.81467636933066	-1.98635830800058	3.58719011129073
C	2.22619088310596	-1.63673711816645	2.30635858316818
H	2.43743075075100	-2.64061049316601	4.18890254318433
C	0.21536711758262	-0.29926767437106	2.04909795488469
H	2.46049270285276	-2.30237376713014	-2.39898023231324
H	0.28533132511646	-1.79217907158407	5.08916090794554
C	-0.19669220164266	-0.67182000050095	3.32441474774577
H	-1.13267290861391	-0.28223181245324	3.71402959706353
H	-5.68341313270621	1.59256602216057	-2.32729383458600
H	-6.40386584064915	2.12434226633250	-0.78891429691187
H	-6.06464815827438	3.30132654917299	-2.06594620283471
H	-5.80873171830891	-3.52886431435953	-0.83605998002082
H	-5.78819743142284	-1.85276517778227	-1.40669275192953
H	-4.33222650984141	-2.85417338027084	-1.55766812812567
C	-5.69455030181993	2.39300966417547	-1.57934564061372
H	-3.42979802132003	4.11904110293792	0.38749200747333
H	-4.85556702497332	4.69702318835893	-0.49241816852725
C	-4.40582677460093	3.82450164200681	-0.00492137757454
H	-5.04510615674230	3.53490935764667	0.83648710151094
H	-3.81441385537033	3.95411303215740	-2.70238950225927

C	-3.38429120485065	3.08417029909049	-2.19312715991692
H	-2.37936332135932	3.34916730870348	-1.85500271207344
H	-3.30141954054941	2.26774584943652	-2.91962639690876
C	-5.19056483359639	-2.62729789829784	-0.91644337825679
H	-6.57642702942027	-2.76609931061467	1.46035436765592
H	-5.64017927990711	-1.54980487376774	2.35704360071339
C	-5.95003010241607	-1.87237146050697	1.35665913398157
H	-6.56099283944948	-1.07831707336534	0.91560533780949
H	-3.65036417639222	-3.12885199940936	2.14685627075482
C	-3.96408382742522	-3.35974974568284	1.12267684650047
H	-4.62169318109922	-4.23426841658441	1.16475796077783
H	6.61753628203002	-1.23316324577198	0.09220231975299
H	6.70274594897461	-2.69592926225178	-1.91231560693882
O	0.63984324116773	2.00509489682486	-0.63656772023991
C	1.86633143941814	1.30122304502228	-0.77386417576111
H	4.55815959460378	2.90648954994742	2.72537330841352
C	5.28590415166943	2.91068541697294	-0.59180769510173
H	2.50026193751237	1.87764564868843	1.89071591307422
C	3.09483610350546	1.96086868971583	-0.19462494616830
C	4.43636842942513	2.74949736425831	1.65779346912822
H	6.36371546826945	3.56598449436843	1.15305069778338
C	4.11418470720738	2.33608526339548	-1.07372803468733
C	5.44934095611630	3.11781072505417	0.77502783183586
H	6.07111096253326	3.19554782100773	-1.28592786593205
C	3.26651562570522	2.16965551333990	1.17853153907272
H	3.99282723061501	2.16956063223028	-2.14112915487805
H	2.01707356068487	1.18855094595971	-1.85699343244971

Phosphenium ion 2a + Benzaldehyde P-b

E[a.u.] (r^2-SCAN-3c-CPCM(CH_2Cl_2): -2397.639236

Gibbs free energy correction (kcal mol^{-1}): 458.25

E[a.u.] (PW6B95-D4/def2-QZVPP-SMD(CH_2Cl_2)): -2401.805814

C	-4.07594231192289	0.99830935045951	0.62433344426571
C	-2.75665676957014	0.82837859994217	1.06216213477160
H	3.30719403703343	0.88638324212281	2.50637454465159
C	-2.08199531977829	-0.32666989479959	0.70271255652991
H	-2.26195410995901	1.58480454385228	1.66178666292625
C	-3.99127753257925	-1.16202910852968	-0.57515889736058
C	-4.63688968216908	-2.21752636342263	-1.47332826009378
C	-4.81984063807245	2.27751379226192	1.01841878711224
C	-4.66007975547032	0.00593138808624	-0.16787501410286
H	-5.67788072421085	0.14487994939345	-0.50349799685763
N	-0.74843634887890	-0.68983622015698	0.99227904152346
C	-2.68701466382592	-1.28077167739266	-0.11703279744378

O	-1.83765356489658	-2.35367004268944	-0.40983454121830
C	1.44367204001188	0.15729545243572	1.68765977955590
C	3.62082664194314	-2.66648032726311	-0.65616166468048
P	-0.27680121202787	-2.05003747523609	0.02232818090226
P	2.31771403328624	-0.34288492677170	0.17750962301678
C	4.25261500667362	-4.45681984822755	0.83234428000216
C	3.07683994288541	-1.96752475464025	0.42978286201824
H	3.60347685675827	-2.25304686048007	-1.66015806027233
C	3.27210512160317	2.24697528046436	-0.06926320150195
C	3.60795470809766	0.88722464457139	-0.12222229299386
H	5.18447902718174	-0.55955094660510	-0.45014677987541
C	4.24473615992324	3.20149861658706	-0.33089162748681
C	3.12062711271588	-2.51856549381171	1.71522661748510
C	3.71213990553057	-3.76081729224789	1.91046261527080
H	4.62775493713814	-4.44921095056741	-1.29009475418018
C	4.20661244275970	-3.90919363668217	-0.44765198792032
H	4.70906647473298	-5.42992608620862	0.98861803669245
H	2.68690731072098	-1.99105577487055	2.55913230340873
H	3.74375302023443	-4.18718678975740	2.90840513793391
C	4.91235891920913	0.49074475395780	-0.42791858814735
C	5.54444222292663	2.80855609801584	-0.64809420711714
H	-5.39412377377750	1.52307660226271	2.98477327773120
C	5.87697597831158	1.45816781010857	-0.69369086992291
H	3.98579156945938	4.25506902040176	-0.28885221983548
C	0.34648055233040	0.89553976422624	4.14436402590137
C	1.70448625050566	1.10336002755116	3.90557966358567
C	2.24883575553741	0.72625039600410	2.68936655570521
H	2.33683257668543	1.55634222499478	4.66199911118471
C	0.07500033789829	-0.07185991487621	1.94217273729602
H	2.26079132205802	2.55955990663920	0.17557486959318
H	-0.08797806221484	1.17617478983622	5.09933882884357
C	-0.45620810934551	0.30788321560009	3.18062189333773
H	-1.50335912012039	0.11179926449460	3.38594338403376
H	-5.21618480882872	-3.50382770164081	0.19048994644888
H	-5.14053793248604	-4.32563973054534	-1.38143677472342
H	-3.64479637820563	-3.93054127289317	-0.51772661538997
H	-6.73756418912360	3.23850163323927	0.80251305621110
H	-6.28465461149150	2.27338925426800	-0.60950677626942
H	-6.85107493506816	1.47360066600893	0.87541849507123
C	-4.65295104099566	-3.57727443269799	-0.74643126401730
H	-6.14554828903379	-0.91434550990078	-2.38526595658124
H	-6.49779445290457	-2.64635810050981	-2.46464579635978
C	-6.08458499595315	-1.85623098913273	-1.82928219740452
H	-6.71641759709313	-1.77916841719479	-0.93761594444527

H	-2.80683001101339	-2.64634013871129	-2.61245110166161
C	-3.83824259711433	-2.33126282240193	-2.78768175938532
H	-4.31484329567010	-3.06941663828302	-3.44279975077747
H	-3.82237226435073	-1.36843577321083	-3.31032559161466
C	-6.25679623329510	2.30726299491105	0.48518868243497
H	-4.00865212955073	3.43289565126205	-0.64673272225495
H	-4.59039662519875	4.41903146129770	0.71287227959679
C	-4.06600081871380	3.49425430741318	0.44599176535943
H	-3.04600637770857	3.55919590309568	0.83868381130846
H	-3.86926211844163	2.43072411407100	2.99205766719619
C	-4.87193473534832	2.38507248581966	2.55474416898388
H	-5.40865316671044	3.29475382388339	2.84743371869669
H	6.89127346866572	1.15120981437081	-0.92964198760586
H	6.30159006773139	3.55984815780465	-0.85351587645732
O	0.15175053632902	-1.25329595027081	-1.41008378595790
C	1.30200244756333	-0.41953419830616	-1.43889057915783
H	-1.19452783213636	3.53432659710500	-1.51760275975998
C	1.57690877465899	2.83105922003901	-3.35322659734245
H	-0.77273493270749	1.24579473852004	-0.71672012514344
C	0.98466560156082	0.96466572580145	-1.93729754043187
C	-0.34456559113070	2.97883773041584	-1.90385092871470
H	0.30755522739648	4.56399030172336	-3.20847799438230
C	1.81962089712954	1.53917214700236	-2.89814638268507
C	0.49771453697349	3.55530249548476	-2.85306883668986
H	2.23056522011528	3.27026045854105	-4.10120524825964
C	-0.10366427107847	1.68807772525983	-1.44611890893873
H	2.66560901598443	0.97630603473782	-3.28572329600396
H	2.04382605591179	-0.87203273640963	-2.11407248916014

Phosphenium ion 2a + Benzaldehyde P-c

E[a.u.] (r^2-SCAN-3c-CPCM(CH_2Cl_2): -2397.643892

Gibbs free energy correction (kcal mol^{-1}): 458.42

E[a.u.] (PW6B95-D4/def2-QZVPP-SMD(CH_2Cl_2)): -2401.810121

C	3.76436298951770	0.36330114573464	0.16392929050757
C	2.67196897317484	0.61165254306223	1.00070272818002
H	-2.25358803149476	3.15282756003673	1.88547830526532
C	1.83979143344747	-0.45245199553122	1.32196648005095
H	2.45470226935547	1.60031005194797	1.38852198474610
C	3.13179821852214	-2.02302986761866	-0.02515366079973
C	3.33458070556969	-3.41852278292209	-0.61318636019958
C	4.73611411682145	1.47530743087068	-0.24224340675148
C	3.96920528295265	-0.93700879011060	-0.31976331167115
H	4.81895799629625	-1.11410021585243	-0.96725640769472

N	0.66713580711615	-0.42305082440646	2.09543875038371
C	2.06880655301188	-1.72976935072556	0.82243384232050
O	1.10318482510181	-2.64075423310852	1.25513801256447
C	-0.92418904870954	1.44340780058330	1.88866967056696
C	-1.94678583524494	3.19472314907052	-1.32027836461558
P	-0.17771725242239	-1.93741624491074	2.06326745146345
P	-1.58426420212844	0.93086671225490	0.27403690341445
C	-0.19964342574907	4.15178022988477	-2.68092474657140
C	-1.07535950739425	2.16871735349331	-0.93812102635611
H	-2.96074879806378	3.23515956389278	-0.93552730237901
C	-3.96555035423740	0.57859293946678	1.64278336503816
C	-3.37449809895646	0.82942261494213	0.39880316902262
H	-3.70356501750425	1.03414480485370	-1.73549541563504
C	-5.34059996263160	0.39193341237590	1.72241158244844
C	0.24463395149901	2.15058329588422	-1.40948502097169
C	0.67328753245910	3.14102283698092	-2.28398265280451
H	-2.18173610885521	4.97488989926090	-2.49210271425570
C	-1.50354637045702	4.18102777749648	-2.19450105111738
H	0.14069212696529	4.92321328452311	-3.36557283152177
H	0.94652384834446	1.38305926601393	-1.09224739239732
H	1.69413846629342	3.12389261853070	-2.65278649673641
C	-4.15805324710242	0.87077856659049	-0.76294938559160
C	-6.12265164135086	0.45415115269185	0.57163842977483
H	3.79740396492171	1.77733099929608	-2.18948763079910
C	-5.53208782884637	0.69195613338043	-0.66737637735785
H	-5.79999094650972	0.19922547816856	2.68698336953841
C	0.07414582623877	2.51513512850329	4.26579624614118
C	-0.95480972433638	3.16566427879136	3.58826983413748
C	-1.45141011024458	2.63578803932001	2.40618375428230
H	-1.37294820037590	4.08785486102973	3.97918843832265
C	0.10660143634016	0.78564607149937	2.58026670489783
H	-3.36074589777135	0.52876274209497	2.54313341025114
H	0.46391114313000	2.92782030793280	5.19146975914897
C	0.59808421754079	1.33029134620905	3.76567747370288
H	1.39252288674532	0.80457960136985	4.28707357954065
H	4.51024530245814	-2.80658460672183	-2.35893960086229
H	5.49357315400733	-3.25630677131392	-0.94503078494463
H	4.67748705791236	-4.50627429407316	-1.89627525082311
H	6.51095481093897	0.19612312363474	-0.10094309162541
H	6.84656746288547	1.93347122746364	0.01883708158087
H	6.12308929746782	1.07007184894187	1.39371437165679
C	4.58061599019987	-3.48847275144753	-1.50445667168383
H	4.36793714644331	-4.19148252265962	1.14503530314965
H	2.61905655723211	-4.49706735683922	1.16214249340455

C	3.50316771900258	-4.44698481163004	0.52258161789632
H	3.67177893120928	-5.44156770093360	0.09429431737890
H	2.24843516134539	-4.77402546644744	-1.91843498157856
C	2.10807852392548	-3.78108801102240	-1.47618560969281
H	1.18698502083255	-3.79671371626268	-0.88754601300982
H	1.98655457554187	-3.05716202599628	-2.29022356476583
C	6.13970209441953	1.14435360460820	0.30062621008224
H	4.29612541204715	2.85361731115963	1.40277035481443
H	5.03207948233737	3.60099033418806	-0.02177918228525
C	4.31374134125877	2.84297514568381	0.30731302735741
H	3.32336318327520	3.13737081751503	-0.06013743846606
H	5.46204346393800	2.38962812330256	-2.07588477728352
C	4.79306800766655	1.57375240574606	-1.77892024112931
H	5.16626718495268	0.65081767105034	-2.23298218723990
H	-6.14006887084883	0.73113152135945	-1.56598731000240
H	-7.19757743755939	0.31351767462069	0.64003899352458
O	-1.30305657920444	-1.65005769564514	0.85078910763580
C	-0.99223674527638	-0.79075768743158	-0.24840813438608
H	-1.36140953765119	-1.03891733313033	-4.87338754832615
C	-3.54609893806409	-2.31744495186680	-2.60648416084856
H	-0.19455398672962	-0.38874607097368	-2.79360472818472
C	-1.70053061375189	-1.23027416122777	-1.49794586452211
C	-1.79933594547362	-1.28480239389285	-3.91048802693181
H	-3.50892920781436	-2.28171234446694	-4.75889563097749
C	-2.90352847890743	-1.93704441917072	-1.43305780666214
C	-3.00202960243262	-1.98535203324775	-3.84499582441595
H	-4.47838120524147	-2.87210743451256	-2.55163551752900
C	-1.14508489154018	-0.91370363294433	-2.74069867181753
H	-3.33236175494611	-2.19162132136040	-0.46923721750323
H	0.08604880116489	-0.73053118190768	-0.44289647946789

Phosphenium ion 2a + Benzaldehyde (isomer)

E[a.u.] (r^2-SCAN-3c-CPCM(CH_2Cl_2): -2397.634665

Gibbs free energy correction (kcal mol^{-1}): 458.21

E[a.u.] (PW6B95-D4/def2-QZVPP-SMD(CH_2Cl_2)): -2401.800838

C	3.63425708460365	-1.40970903313197	0.07722020913705
C	2.34378032157040	-1.52542108571750	-0.44339311553100
H	-3.52308946785983	-2.59400829549364	-1.81431474448600
C	1.69141282522799	-0.37082823412046	-0.85439749185063
H	1.84728434315946	-2.48367681809228	-0.53618968923535
C	3.58073754640419	1.04576013999751	-0.23722607283692
C	4.24524514707126	2.41953400046871	-0.14130040876856
C	4.41715621573304	-2.63067861270098	0.56856187214732

C	4.21304151735245	-0.13646581796228	0.17368227052283
H	5.20862644080776	-0.05874156554532	0.59234146409027
N	0.38515728410931	-0.27303870807376	-1.37227336227407
C	2.29760222887887	0.87940328318350	-0.75139775249876
O	1.51040474708103	1.91547321735532	-1.26189146449055
C	-1.67976430652409	-1.50139287819735	-1.51779535446798
C	-0.59705325710354	-2.52194895040084	1.28212249573664
P	-0.04521956738569	1.41169349488222	-1.60961570367987
P	-2.22732174506566	-0.67513016119586	-0.01890256983895
C	0.06170203307257	-2.28540168820714	3.59002112370223
C	-1.29775574196600	-1.31554028163207	1.37378608981021
H	-0.58146869202201	-3.08401997729837	0.35292748666638
C	-4.46314837322182	-1.83864475445205	1.13029338359345
C	-3.98840417469301	-0.92682460933466	0.18359257606301
H	-4.49972545103873	0.48932236789745	-1.37477850166970
C	-5.83449230472683	-2.03517558713720	1.25691925126582
C	-1.33157671372049	-0.59796631487381	2.57823150450074
C	-0.64388698457865	-1.08586119696923	3.68097878983645
H	0.62291237050591	-3.94098058674662	2.33256583432200
C	0.07961904123925	-3.00346945448951	2.39770031352341
H	0.59748275880473	-2.66308354147280	4.45611467254900
H	-1.88831511207948	0.33317369171114	2.65142277167795
H	-0.65948968483271	-0.52970205556866	4.61321208383396
C	-4.87369829604101	-0.21552057437016	-0.63722909879240
C	-6.71882768371679	-1.32709455995083	0.44782441855305
H	2.71295333044833	-3.98254425890697	0.82434675107197
C	-6.24003088158575	-0.41923141859874	-0.49645355963521
H	-6.21018243353147	-2.74045105049454	1.99179432580825
C	-0.61632329936697	-3.15233855587231	-3.48152153347633
C	-1.95592451938642	-3.28656565283298	-3.11189664753051
C	-2.49157894346584	-2.46725974361400	-2.12815887177986
H	-2.57927927563565	-4.03942390745796	-3.58341893047539
C	-0.34609505032022	-1.33206015538585	-1.93530140156242
H	-3.77229667523570	-2.38907271587538	1.76243958699717
H	-0.20135631476127	-3.79897217867593	-4.24920022600619
C	0.18886217604819	-2.17743319019071	-2.90498827970478
H	1.21900513571834	-2.04507207745025	-3.22190662012450
H	3.88168688263955	4.32531595462846	0.83145100620694
H	2.39562843004369	3.51517303348135	0.30708728988422
H	3.26449698197351	2.92996762673906	1.74019732409844
H	5.19647979080109	-3.37192319876576	2.46041954712044
H	5.21194861914822	-1.60231698398013	2.33418806607710
H	3.68087098261379	-2.45489341769605	2.61525901513376
C	3.38697006237963	3.35196068022947	0.73614949174745

H	6.32005799144210	1.71337611661579	-0.11229678273916
H	6.06954616471705	3.34289471802243	0.52941010120898
C	5.64341806154782	2.33539007631707	0.48372416015976
H	5.61027179520982	1.93893640670904	1.50455290575662
H	4.89019317021931	3.99198515790495	-1.49111112062062
C	4.39139685781539	3.01815927653982	-1.55497490043090
H	4.99984048495921	2.36222262173677	-2.18742286370982
H	3.42176766151296	3.16272244739099	-2.03746232936430
C	4.64092003278337	-2.50335024635938	2.08786801453422
H	6.39571389447590	-1.81837677510537	0.06316502174954
H	6.33264157333768	-3.58277102766230	0.18895580480605
C	5.77988044705601	-2.69803573613045	-0.14716332722697
H	5.64320710626413	-2.76868745851965	-1.23206891142845
H	3.48845739580761	-4.08973159712553	-0.77302647413429
C	3.67261192969799	-3.94338022080697	0.29744298556729
H	4.28029535990490	-4.78246195634252	0.65180414423331
H	-6.93295046970764	0.13147836113477	-1.12519280984611
H	-7.78883169853446	-1.48213916871840	0.55239168570050
C	-0.85176316558735	1.67130411491573	0.09068265468855
O	-2.12253687086632	0.93359881236399	-0.00480569415155
C	-1.14879668715020	3.10341869875128	0.36029485734777
H	-2.64569965932096	3.27978706978970	-1.18584059397100
C	-0.63927481040752	5.13835322536289	1.56077357729831
C	-2.29970022377029	5.15549312824669	-0.19042229079480
H	0.29235319842223	3.23611074629293	1.95489963202693
C	-0.42971605494655	3.77884382657801	1.34983415529964
C	-1.57602953282893	5.82743218614372	0.79411007791562
H	-3.03153953341501	5.69086465630492	-0.78848346941466
C	-2.08339189189468	3.80075689361393	-0.41519092727682
H	-0.07730515236666	5.65718861102129	2.33206583791392
H	-1.74470019683245	6.88693056081544	0.96435079430177
H	-0.22231728211506	1.23032570355713	0.87461323963909

P-phosphanyldiazaphospholenes 1 (Burck et al.)[25]

E[a.u.] (r^2-SCAN-3c-CPCM(CH_2Cl_2): -2031.766578331837

Gibbs free energy correction (kcal mol^{-1}): 381.63

E[a.u.] (PW6B95-D4/def2-QZVPP-SMD(CH_2Cl_2)): -2035.217076104960

C	-3.56669774329898	0.85778542853530	-0.56228966956985
N	-3.39494330019076	-0.39615908116266	0.04948016542401
P	-2.07671306372284	-0.40531450360613	1.16985814287391
C	-2.54659933406831	1.69295696629900	-0.30826760942218
H	-4.41142318294507	1.03349882307138	-1.21408671344586
N	-1.56408266667624	1.11131703208682	0.51123196385982

C	-4.26190157536628	-1.49778039426422	-0.20245568937408
H	-2.39467267279532	2.68206063934700	-0.71767422811335
C	-0.32357281427226	1.75137025762910	0.79672072356175
C	-3.73951657995398	-2.66960121979898	-0.77995059861863
C	-5.95531281234716	-3.68546511926261	-0.66812603704178
C	-6.86535184656878	-4.85283614835762	-0.93791431817318
C	-5.62576387186693	-1.40323575253513	0.12950747117141
C	-6.20244870488371	-0.17180716045906	0.77276489184700
H	-7.50098677632890	-2.43918185922420	0.15368283493882
C	-6.44849925221660	-2.50382577524834	-0.11636909687771
C	-4.59901082058029	-3.74777753507012	-0.99041370479170
C	-2.29389120466657	-2.77685827115188	-1.17838840067972
H	-4.19745374911616	-4.65754456324763	-1.43318449874686
H	-1.94042788836462	-1.85033320959247	-1.64263713196290
H	-1.64943081236769	-2.96420448085310	-0.31070750972285
H	-2.15498389514130	-3.60108539370311	-1.88290515230526
H	-5.50684596986029	0.26884505510678	1.49415272460356
H	-6.42750786352895	0.60389885129877	0.03085367827699
H	-7.13469517063308	-0.41673716220053	1.28838715155421
C	2.12676422646042	3.00099946357127	1.35521587748033
C	-0.31298872987529	3.01523768192780	1.41375961493055
C	2.08448072890407	1.75645323620742	0.72702911043026
H	0.92852231801390	4.58961272579056	2.16519717562419
C	0.91835957010986	3.61726393053774	1.67541898645176
C	3.43943279945630	3.64468614492842	1.70980475397130
C	-1.57792658331136	3.73117660609808	1.79966031424481
C	0.87924396233574	1.12056922994363	0.42828006203124
C	0.89387165866697	-0.20304345392970	-0.28461977872610
H	3.01622521815264	1.26947388158011	0.44459439487783
H	1.86472020055221	-0.36629628008641	-0.76001126218973
H	0.71473458330139	-1.03634133816093	0.40635194008471
H	0.11258910449383	-0.25249078792644	-1.05048413628748
H	-2.36991259362412	3.03443133948090	2.08629498101923
H	-1.38691428001659	4.40446026406064	2.63950220777923
H	-1.96169719012762	4.33759680134379	0.96965553412331
P	-3.05623471073010	0.24432039300391	3.27967610227910
C	-3.21402808739225	-1.47668257538528	3.90814232162706
C	-1.45615443032754	0.77524234339579	4.01887382584383
C	-0.19792830282418	2.48912713198184	5.19174485195411
C	0.92117674798663	0.49538683364897	4.42908572020157
H	-2.30080385270456	2.61256243396971	4.76266664532656
C	-1.39734503624746	2.01503772113064	4.66794371902655
C	0.96533577420273	1.73291059002824	5.06959361757584
H	-0.17243557330798	3.45147315639923	5.69609159204932

C	-0.27851490295704	0.01796030843920	3.91133344777167
H	-0.30194379508398	-0.95791593709367	3.43248916051914
H	1.82465195615007	-0.10097010135210	4.33220482113673
H	1.90299834453741	2.10392548703326	5.47467748199068
C	-2.61310387826053	-1.91782341604588	5.09712245176236
C	-2.81737030732323	-3.21482896985440	5.55792924964209
H	-1.98185934433121	-1.24289087325727	5.66814844977190
H	-3.77429879863120	-5.11639743841094	5.20535302125425
C	-3.62105148700450	-4.10341247540084	4.84389683085365
C	-4.22835401971792	-3.67615723521406	3.66551096280620
C	-4.03198374582207	-2.37666528182940	3.20588314193924
H	-2.34407260114287	-3.53322642995634	6.48330171660956
H	-4.53072883910576	-2.06342607525716	2.29209121365878
H	-4.86137501061672	-4.35362902174063	3.09783681134380
H	4.23574729507859	3.32412620381997	1.03156064099522
H	3.36973484727787	4.73610218646362	1.67707015560523
H	3.74293046014274	3.36662725878034	2.72742857086279
H	-7.74652847200118	-4.82608430103522	-0.29057826898501
H	-7.21805089444395	-4.83770195833509	-1.97680386906599
H	-6.34666475713096	-5.80392382692966	-0.78393155146732

P-phosphanyldiazaphospholenes 2 (Burck et al.)[25]

E[a.u.] (r^2-SCAN-3c-CPCM(CH_2Cl_2): -2037.760036195814

Gibbs free energy correction (kcal mol^{-1}): 453.10

E[a.u.] (PW6B95-D4/def2-QZVPP-SMD(CH_2Cl_2)): -2041.237072849063

C	-3.29688521886912	0.67612727308060	-0.97949174652568
N	-3.42125603603989	-0.23615214560205	0.06559350871459
P	-2.43141403795442	0.09431087330190	1.44350429240515
C	-2.33395704220750	1.58362958648633	-0.73496207647325
H	-3.91583227669140	0.58388015903915	-1.86242271309078
N	-1.71663234023571	1.36700116227560	0.49858085686441
C	-4.27949080582873	-1.37471292320698	-0.04901674781176
H	-2.00187287929866	2.38363776284112	-1.38184194478387
C	-0.62647334780783	2.18112834785731	0.93148219699236
C	-3.71450416094591	-2.61925691258424	-0.36334378532093
C	-5.94757410175426	-3.59544559098478	-0.33045337880699
C	-6.84214275351699	-4.80045867584196	-0.42975379741349
C	-5.66331191852484	-1.21464463130836	0.10658640222298
C	-6.25894408606927	0.13267551606793	0.40324821686490
H	-7.55250298665423	-2.22754502518860	0.09100101200764
C	-6.47720644115575	-2.33829249419144	-0.03616143610373
C	-4.56775660857867	-3.71530827429469	-0.49951302258951
C	-2.23094467290852	-2.77728834300145	-0.54839272153580

H	-4.14127026331954	-4.68637430319086	-0.74352938214560
H	-1.82310811139002	-1.99426790772242	-1.19662767172612
H	-1.70315076942753	-2.70438932150810	0.41071401044739
H	-2.00132139623762	-3.75182102937676	-0.98641457993802
H	-5.70924906121100	0.64485174214075	1.19969826571538
H	-6.22184550519766	0.78359428195924	-0.47899024217565
H	-7.30461281339551	0.03256623939180	0.70498188602630
C	1.52043094193430	3.79006473428998	1.74890000813311
C	-0.79446471558283	3.57559825725133	1.01240555619675
C	1.65270143340055	2.40493931440480	1.67012128997413
H	0.15729362103420	5.43186816175498	1.49265708647487
C	0.29007564744675	4.35435222193259	1.41235582624151
C	2.67775243313936	4.65725090817198	2.16329208349446
C	-2.11946698633977	4.24017366873278	0.74656967258758
C	0.60329944998803	1.58212509965202	1.25515490640575
C	0.82802371848563	0.09805668529240	1.15726036718773
H	2.60555404184684	1.94495670901854	1.92503324728055
H	1.89861794260919	-0.11779061148578	1.10679774163308
H	0.41983391644122	-0.42670014929456	2.02999990990586
H	0.34087256965453	-0.32600801377781	0.27294897500196
H	-2.95777884680516	3.60325749555117	1.04549213492323
H	-2.18106949307842	5.18172385354104	1.29919408271009
H	-2.25339191695816	4.47451624341410	-0.31614092539694
P	-3.85671056387901	0.99146559499731	3.41689998441183
C	-2.54139813913474	-1.02977377845488	4.45538861094808
C	-0.24566040344801	-0.00563542243958	5.07509591564227
C	-1.63758682500634	0.09995332852746	4.50168572238118
C	-2.12283949028249	-2.39055387758887	4.95287750639445
C	-2.19583404917746	1.27953075309107	4.04161715611899
C	-1.60268229515650	2.65553281746694	4.13852201996131
C	-5.00560519899710	-1.62406364711420	3.86795309558011
C	-3.78790423632761	-0.73091268250167	3.93299809736712
C	-2.09912450774075	3.42314906419296	5.37490495470885
H	-0.50598656533352	2.60648294729823	4.14938396877089
H	-1.87284021114677	3.23836528845471	3.25325668450544
H	-1.85557571631195	2.89209897909241	6.30010164783383
H	-1.64414609109217	4.41960308278533	5.42030763738494
H	-3.18710509306650	3.54486362619721	5.33677200864943
C	-0.18661084916610	0.22844736019222	6.59250737657890
H	0.16829559066681	-0.99586359521477	4.85232203292474
H	0.41153320918486	0.71757465711171	4.57648880571949
H	-0.85988017341500	-0.45321891017855	7.12333993510876
H	0.82870240368581	0.06923823800774	6.97265136470319
H	-0.48530103352782	1.25078058013397	6.84152718587247

H	-1.46227990059328	-2.28045992276993	5.82172236726380
H	-3.00191044141980	-2.93944611598115	5.30831010726681
C	-1.39480273227564	-3.22067036298004	3.88303199344502
H	-0.49078352435829	-2.70680387601831	3.53853941295349
H	-1.10119981231595	-4.20064302442297	4.27680021001601
H	-2.03644300399891	-3.37555784561651	3.01005948035434
H	-5.80930708985416	-1.06536636255808	3.37040664491134
C	-4.86196742513198	-2.98108219284914	3.16826605261573
H	-5.36343337864621	-1.79642303790963	4.89536951088066
H	-4.43843489894093	-2.86148110892021	2.16807932105543
H	-4.22579255719708	-3.67079110227118	3.72768987969399
H	-5.84628834261928	-3.45111412580297	3.06040213232047
H	3.40035491981662	4.09551389332735	2.76189994494603
H	3.20650504863181	5.04466703065805	1.28344107172217
H	2.33750950730928	5.52013928930616	2.74400476688546
H	-7.83322168919080	-4.53019349456759	-0.80593894440437
H	-6.41221927459792	-5.56215057647876	-1.08653544309686
H	-6.98018966394064	-5.25752993309041	0.55836524503048

11. References

1. D. L. J. Broere, L. L. Metz, B. de Bruin, J. N. H. Reek, M. A. Siegler and J. I. van der Vlugt, *Angew. Chem. Int. Ed.*, 2015, **54**, 1516-1520.
2 D. Roth, A. T. Radosevich and L. Greb, *J. Am. Chem. Soc.*, 2023, **145**, 24184-24190.
3 I. Krossing, *Chem. – Eur. J.*, 2001, **7**, 490-502.
4 L. Falivene, Z. Cao, A. Petta, L. Serra, A. Poater, R. Oliva, V. Scarano and L. Cavallo, *Nat. Chem.*, 2019, **11**, 872-879.
5 L. Krause, R. Herbst-Irmer, G. M. Sheldrick and D. Stalke, *J. Appl. Crystallogr.*, 2015, **48**, 3-10.
6 G. M. Sheldrick, *Acta Crystallogr. A: Found. Adv*, 2015, **71**, 3-8.
7 G. M. Sheldrick, *Acta Crystallogr. C: Struct. Chem.,* 2015, **71**, 3-8.
8 C. R. Groom, I. J. Bruno, M. P. Lightfoot and S. C. Ward, *Acta Crystallogr. B B: Struct. Sci. Cryst. Eng. Mater*, 2016, **72**, 171-179.
9 D. Kratzert, *FinalCif*, https://dkratzert.de/finalcif.html, (last accessed 16 January 2024).
10 A. D. Gorman, J. A. Cross, R. A. Doyle, T. R. Leonard, P. G. Pringle and H. A. Sparkes, *Eur. J. Inorg. Chem.*, 2019, **2019**, 1633-1639.
11 F. Neese, *WIREs Computational Molecular Science*, 2012, **2**, 73-78.
12 F. Neese, *WIREs Computational Molecular Science*, 2018, **8**, e1327.
13 F. Neese, F. Wennmohs, A. Hansen and U. Becker, *Chem. Phys.*, 2009, **356**, 98-109.
14 K. Eichkorn, O. Treutler, H. Öhm, M. Häser and R. Ahlrichs, *Chem. Phys. Lett.*, 1995, **242**, 652-660.
15 K. Eichkorn, F. Weigend, O. Treutler and R. Ahlrichs, *Theor. Chem. Acc.*, 1997, **97**, 119-124.
16 C. Y. Legault, *CYLview20* 2020, *Université de Sherbrooke*.
17 S. Grimme, A. Hansen, S. Ehlert and J.-M. Mewes, *J. Chem. Phys.*, 2021, **154**, 064103.
18 S. Grimme, *Chem. Eur. J.*, 2012, **18**, 9955-9964.
19 C. Bannwarth, E. Caldeweyher, S. Ehlert, A. Hansen, P. Pracht, J. Seibert, S. Spicher and S. Grimme, *WIREs Computational Molecular Science*, 2021, **11**, e1493.
20 P. Pracht, F. Bohle and S. Grimme, *Phys. Chem. Chem. Phys.*, 2020, **22**, 7169-7192.
21 A. V. Marenich, C. J. Cramer and D. G. Truhlar, *J. Phys. Chem. B*, 2009, **113**, 6378-6396.
22 Y. Zhao and D. G. Truhlar, *J. Phys. Chem. A*, 2005, **109**, 5656-5667.
23 E. Caldeweyher, S. Ehlert, A. Hansen, H. Neugebauer, S. Spicher, C. Bannwarth and S. Grimme, *J. Chem. Phys.*, 2019, **150**, 154122.
24 E. D. Glendening, C. R. Landis and F. Weinhold, *J. Comput. Chem.*, 2013, **34**, 1429-1437.
25 S. Burck, D. Gudat and M. Nieger, *Angew. Chem. Int. Ed.*, 2004, **43**, 4801-4804.
